# Functionally relevant microsatellites in sugarcane unigenes

**DOI:** 10.1186/1471-2229-10-251

**Published:** 2010-11-17

**Authors:** Swarup K Parida, Awadhesh Pandit, Kishor Gaikwad, Tilak R Sharma, Prem Shankar Srivastava, Nagendra K Singh, Trilochan Mohapatra

**Affiliations:** 1 National Research Centre on Plant Biotechnology, Indian Agricultural Research Institute, New Delhi-110012, India; 2 Department of Biotechnology, Jamia Hamdard University, New Delhi-110062, India

## Abstract

**Background:**

Unigene sequences constitute a rich source of functionally relevant microsatellites. The present study was undertaken to mine the microsatellites in the available unigene sequences of sugarcane for understanding their constitution in the expressed genic component of its complex polyploid/aneuploid genome, assessing their functional significance *in silico*, determining the extent of allelic diversity at the microsatellite loci and for evaluating their utility in large-scale genotyping applications in sugarcane.

**Results:**

The average frequency of perfect microsatellite was 1/10.9 kb, while it was 1/44.3 kb for the long and hypervariable class I repeats. GC-rich trinucleotides coding for alanine and the GA-rich dinucleotides were the most abundant microsatellite classes. Out of 15,594 unigenes mined in the study, 767 contained microsatellite repeats and for 672 of these putative functions were determined *in silico*. The microsatellite repeats were found in the functional domains of proteins encoded by 364 unigenes. Its significance was assessed by establishing the structure-function relationship for the beta-amylase and protein kinase encoding unigenes having repeats in the catalytic domains. A total of 726 allelic variants (7.42 alleles per locus) with different repeat lengths were captured precisely for a set of 47 fluorescent dye labeled primers in 36 sugarcane genotypes and five cereal species using the automated fragment analysis system, which suggested the utility of designed primers for rapid, large-scale and high-throughput genotyping applications in sugarcane. Pair-wise similarity ranging from 0.33 to 0.84 with an average of 0.40 revealed a broad genetic base of the Indian varieties in respect of functionally relevant regions of the large and complex sugarcane genome.

**Conclusion:**

Microsatellite repeats were present in 4.92% of sugarcane unigenes, for most (87.6%) of which functions were determined *in silico*. High level of allelic diversity in repeats including those present in the functional domains of proteins encoded by the unigenes demonstrated their use in assay of useful variation in the genic component of complex polyploid sugarcane genome.

## Background

Sugarcane (*Saccharum sp*.) is a complex polyploid belonging to the family Poaceae of the tribe Andropogoneae. It is an important commercial sugar producing crop and a source of approximately 50% of the world's sugar and alcohol. The polyploid/aneuploid nature with variation in chromosome number has been largely responsible for its genetic and taxonomic complexity [1]. Characterization of such large genomes is greatly facilitated by the use of molecular markers. Microsatellite or simple sequence repeat (SSR) markers are being preferred because of their co-dominant inheritance, reproducibility, multi-allelic nature, chromosome-specific location and wide genomic distribution. These markers are amenable to high throughput genotyping due to multiplexing and efficient resolution of amplicons by automated fragment analysis [2,3].

In sugarcane, Cordeiro *et al. *(2000) [2] and Parida *et al. *(2009a) [4] developed a large number of microsatellite markers from the genomic sequences. Pinto *et al. *(2004; 2006) [5,6] and more recently Oliveira *et al. *(2009) [7] also designed such markers from the sugarcane ESTs, which were used for constructing high resolution functional genetic linkage map of *Saccharum *spp. [8]. However, information on a limited number of these genic microsatellite markers is available in the public domain. Recently, the EST sequences have been assembled into unigenes [9], which is expected to provide non-redundant, locus specific and novel gene-based functional markers for sugarcane having a large genome not amenable to complete sequencing. The unigene sequences of sugarcane have not yet been analyzed for their microsatellite constitution and compared with the other small genome members of the grass family.

In India, systematic breeding of sugarcane has resulted in the development of a number of varieties with high productivity and stress tolerance by inter-specific hybridization [10]. However, the genetic base of modern Indian sugarcane cultivars is considered narrow due to use of a limited number of parental species clones in cross hybridization and repeated intercrossing of hybrids [11]. Understanding the extent of natural variation at molecular level is essential to develop new strategies for sugarcane improvement. Earlier, molecular markers such as RAPD (Randomly Amplified Polymorphic DNA), AFLP (Amplified Fragment Length Polymorphism), and maize and sugarcane genomic microsatellites have been used for this purpose [4,12-15]. No effort has yet been made to understand the genetic diversity of Indian sugarcane cultivars based on functionally relevant genic regions of its complex genome.

The present study was undertaken to mine the available unigene sequences of sugarcane (*Saccharum *sp.) to understand the microsatellite structure and distribution in the expressed genic component of the genome, assess their functional significance *in silico*, design primers from the flanking regions of the identified microsatellites, assess the efficiency of a set of fluorescent dye labeled primers in genotyping using automated fragment analysis system and determine functional diversity among different species, related genera and Indian varieties of sugarcane.

## Results

### Frequency, distribution and organization of microsatellites in sugarcane unigenes

The type, frequency and relative distribution of the microsatellites in the unigene sequences of sugarcane are given in Table 1. The perfect microsatellite (excluding the mononucleotides) frequency in the unigenes of sugarcane was one in every 10.9 kb and the proportion of microsatellite carrying unigenes was 3.7% (584 out of 15,582). When 1,871 (12%) mononucleotide microsatellites were included, the proportion of unigenes carrying microsatellites increased to 17.4%. The mononucleotides in sugarcane showed a strong bias (84.6%) towards A/T repeat, with the majority (89%) being 9 to 30 bases long and the remaining (11%) extending up to 69 bases (T_69_). A total of 841 perfect microsatellites were identified in 584 unigene sequences of sugarcane. One hundred sixty-seven (28.6%) of these unigenes contained multiple microsatellites (that accounted for 424 microsatellites) which were interrupted by more than 100 nucleotides and the remaining 417 (71.4%) unigenes had a single microsatellite each. In addition to the perfect microsatellites, we identified 183 compound microsatellites, of which 74.9% were interrupting types and the rest being non-interrupting types (Table 1). The trinucleotide repeat motifs were the most prevalent (73.1%) class of microsatellites followed by dinucleotide (23.8%), tetranucleotide (1.8%), pentanucleotide (0.83%) and hexanucleotide (0.47%) repeat motifs (Table 1). The GC rich repeat motifs GCA/GCC/GCG/GCT (23%) coding for alanine were most abundant followed by arginine (AGA/AGG/CGA/CGC/CGG/CGT, 22%) and glycine (GGA/GGC/GGT, 11%) (see Additional file 1). Among the dinucleotide repeats, the most and least frequent motifs were GA and CG, which accounted for 21% and 1.4% of all the microsatellites, respectively (see Additional file 2).

**Table 1:** Distribution of microsatellites in the unigene sequences of sugarcane

Characters under study	Unigenes*
Number of sequences examined	15,594

Size (bp) of examined sequences	9,17,43,95

Number of identified perfect microsatellites	2,712 (17.4)

Number of perfect microsatellite containing sequences	2,230 (14.3)

Number of perfect microsatellite (excluding mononucleotides) containing sequences	584 (3.7)

Number of sequences containing more than one perfect microsatellites	167 (28.6)

Number of sequences containing single and unique perfect microsatellites	417 (71.4)

Number of mononucleotides	1,871 (12)

Number of dinucleotides	200 (23.8)

Number of trinucleotides	615 (73.1)

Number of tetranucleotides	15 (1.8)

Number of pentanucleotides	7 (0.83)

Number of hexanucleotides	4 (0.47)

Number of perfect microsatellites excluding mononucleotides	841 (5.4)

Size (kb) of sequences containing one perfect microsatellite	10.9

Number of perfect class I microsatellites	207 (24.6)

Size (kb) of sequences containing one perfect class I microsatellite	44.3

Number of primer pairs designed for perfect^a ^microsatellites	810 (96.3)

Number of compound class I microsatellite containing sequences	183

Size (kb) of sequences containing one compound class I microsatellite	50.1

Number of compound class I microsatellites	183 (1.2)

Number of compound interrupting class I microsatellites	137 (74.9)

Number of compound non-interrupting class I microsatellites	46 (25.1)

Number of primer pairs designed for compound class I microsatellites	151 (82.5)

*The number in the bracket is the proportion expressed in percentage

^a^Mononucleotides to hexanucleotides repeated up to 100 times without any interruption at a locus

**Figure 1:**
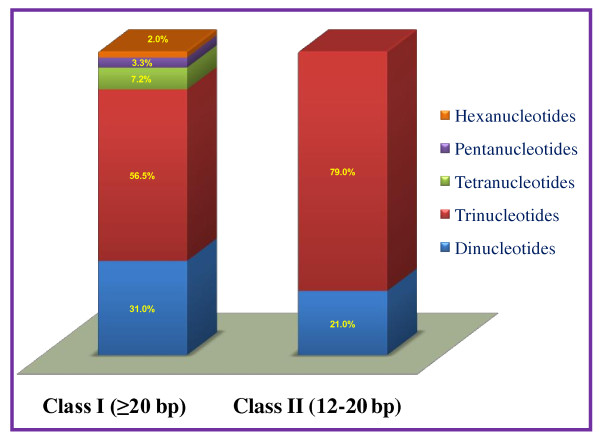
**Frequency and relative distribution of class I and class II microsatellite repeats in sugarcane unigenes**. Comparative distribution of long hypervariable class I and potentially variable class II microsatellite repeats in the unigenes of sugarcane. Trinucleotide was the most abundant repeat-motif in both class I (56.5%) and class II (79%) category, which was followed by dinucleotide motifs.

Out of 841 perfect microsatellites identified in sugarcane, 587 (69.8%) were found in the ORFs and the remaining were present either in the 3'UTRs (102, 12.1%) or in the 5'UTRs (152, 18.1%). The trinucleotide repeat motifs were significantly more frequent (about 86%) in the ORFs. In contrast, the GA-rich dinucleotide repeat motifs were more in the 5' (49%) and 3' (32%) UTRs. The density of longer motif containing perfect class I microsatellites was one in every 44.3 kb sequences, which accounted for 24.6% (207) of the total 841 microsatellites identified (Table 1, Figure 1).

### Development of unigene derived microsatellite (UGMS) markers and evaluation of their polymorphic potential

The primer pairs could be designed for 810 perfect microsatellites that was 96.3% of the total microsatellites (841) identified in the present investigation. The primer sequences flanking all the perfect UGMS including 207 class I microsatellites with their Tm values and product sizes are given in the Additional file 3. Besides, the primer sequences for 151 compound class I UGMS were designed and provided in the Additional file 4. To validate the UGMS markers, 176 primer-pairs designed from different microsatellite containing unigenes were used in PCR amplification (see Additional file 3). One hundred sixty seven (94.9%) of these produced clear and reproducible amplicons, whereas remaining nine (5.1%) did not give amplification in the *S. officinarum *from which sequence the primers were designed. To verify that the primers did amplify the expected microsatellite repeat-motifs, the amplified products obtained with 19 of the primers in all the *Saccharum *species and related genera as well as cereals were sequenced and the presence of the target microsatellite motifs as well the flanking sequences was observed in all the cases (see Additional file 5).

*In silico *polymorphism analysis was confined to *Saccharum officinarum *and five cereal species namely, rice, wheat, maize, *Sorghum *and barley for which unigene sequences were available in the database. The polymorphism based on variation in microsatellite repeat length was observed for a maximum of 163 primers (46.6%) between sugarcane and barley followed by 161 (42.5%) between sugarcane and wheat and least (92, 17.8%) between sugarcane and *Sorghum *(see Additional file 6). The actual level of polymorphism detected by automated fragment analysis using 47 fluorescent dye labeled primers was much higher than that based on *in silico *analysis although the trend was maintained. *S. officinarum *had maximum polymorphism with barley (92.7%) followed by wheat (90.6%), rice (85.8%), maize (67.7%) and *Sorghum *(61.4%). Forty-three (91.5%) of the 47 primers detected polymorphism (mean PIC of 0.85) among the 41 genotypes belonging to *Saccharum *species, related genera, tropical and sub-tropical Indian sugarcane varieties and five cereal species (Table 2). This included 21 (95.5%, mean PIC of 0.81) amplifying dinucleotide repeats, 16 (84.2%, PIC of 0.73) trinucleotide repeats, two (100%, PIC of 0.87) tetranucleotide repeats, two (100%, PIC of 0.86) pentanucleotide repeats and two (100%, PIC of 0.83) amplifying hexanucleotide repeats. All the 38 (100%, mean PIC of 0.82) primers that targeted amplification of class I repeats and five (55.6%, PIC of 0.55) of the nine that targeted class II repeats showed polymorphism among *Saccharum *species, genera, varieties and cereal species. The microsatellites in the 5' and 3'UTR sequences showed higher potential for polymorphism (15 out of 16, 94%, mean PIC of 0.83) as compared to that from the coding regions (28 out of 31, 90%, mean PIC of 0.77).

**Table 2:** Evaluation of the amplification efficiency and polymorphic potential of 47 fluorescent dye labeled primers

														Polymorphic potential		
														
Sl. No.	Unigene Accession IDs'	Class I UGMS primers^A^	Repeat-motifs	Location	Forward primer sequences (5'-3')	Reverse primer sequences (5'-3')	Putative functions	Actual annealing temperature (°C)	No. of locus	Total no. of alleles amplified	Size (bp) of allele (s) amplified	Type of allele size distribution	No. of heterozygous loci	Among sugarcane species and genera	Among sugarcane varieties	
															
															P/M^B^	PIC
1	CA297715	UGSuM2a	(AT)43	CDS	CTGTGTATATGTTCGTAGTTTG	CACTTAGTCACACTCTCACACAC	Sucrose phosphate synthase	55	2	20	206-302	Step-wise	1	P	P	0.86
														
		UGSuM2b								4	481-512	Mixed				

2	CA278792	UGSuM5	(TA)28	CDS	TCACATCCATCATCCACAGC	TCCAATGCAAGCAAACTCAC	Maize-Cyclin III	55	1	23	80-170	Step-wise	0	P	P	0.84

3	AY596609	UGSuM11	(TA)21	3'UTRs	TGGTAACCCTAGGCAGGTGA	GTGCACCAGATTTGGATGGT	Fructose-bisphosphate aldolase	56	1	23	92-190	Step-wise	0	P	P	0.83

4	CA279221	UGSuM15a	(CCTCGC)6	CDS	GTTTAAGACAAGATGGTGTAGATG	TACATATTTACATTGTTACTCCGC	Hypothetical protein	56	2	17	170-247	Mixed	1	P	P	0.80
														
		UGSuM15b								3	360-378	Step-wise				

5	CA278282	UGSuM16	(AT)18	CDS	GCGTCTTCATCATCTGCAAC	GCGTCTTCATCATCTGCAAC	Pathogenesis-related PRMS protein	55	1	22	150-244	Step-wise	0	P	P	0.82

6	CA253277	UGSuM17	(AG)18	CDS	TTTCCATTCTTCCATTCAACTG	GGCAGGCTGAGAGACTGTTC	Abcisic acid-protein kinase	55	1	10	90-188	Step-wise	1	P	P	0.78

7	CA227482	UGSuM18a	(GA)18	CDS	GGCGAGAGAGAGAGAGAGAGAG	AGGTGGAGATCTTGAGGTAGGC	Glycine decarboxylase	56	2	20	96-193	Mixed	1	P	P	0.81
														
		UGSuM18b								2	326-348	Step-wise				

8	CA126180	UGSuM20a	(TCA)12	CDS	ATCCCTTATGCTACAGAAATGT	TTAGCCTAGAGGTTTGATTGAT	Acetyl-CoA synthetase	54	2	16	190-259	Step-wise	1	P	P	0.83
														
		UGSuM20b								4	392-428	Step-wise				

9	CA177414	UGSuM21	(AGGA)9	CDS	CGCTCCCTCACCGTCATT	CTCCGCATCCTCGTCACC	Transcription regulator protein	62	1	20	160-252	Step-wise	0	P	P	0.81

10	CA223153	UGSuM22a	(GCG)12	3'UTRs	CTCCCTCCTCCTCCCGTTG	CTCTTGGGTGTGAACCAG	Polyadenylate-binding protein	64	2	19	96-187	Mixed	1	P	P	0.85
														
		UGSuM22b								3	322-358	Step-wise				

11	CA122659^A^	UGSuM24a	(TTTTC)7	5'UTRs	CTGTACAACAGCAATTATGAATCT	CTCGACTACGAGAGGATATGAT	Hypothetical protein	55	3	12	240-294	Mixed	1	P	P	0.86
														
		UGSuM24b								4	398-428	Step-wise				
														
		UGSuM24c								5	531-571	Step-wise				

12	BU103692^A^	UGSuM26	(CT)17	5'UTRs	CTCGATCCCAGAGAGCTCCACAG	AGTACCGAATTCATTAAACTCCT	Beta-amylase	55	1	21	250-346	Step-wise	0	P	P	0.85

13	CA073284	UGSuM27a	(GGC)11	CDS	CTGCAGTACGGTCCGGAATC	GTACCACCATGGCTCTAGCTTC	30 S ribosomal protein S16	60	2	2	50-54	Mixed	1	P	P	0.84
														
		UGSuM27b								22	154-288	Mixed				

14	AY596606	UGSuM33a	(AGC)10	CDS	CGAGGCACTGAACCCATATC	TGTTTGAACTGGATGGCGTA	Hypothetical protein	58	3	17	92-189	Mixed	1	P	P	0.84
														
		UGSuM33b								2	293-305	Step-wise				
														
		UGSuM33c								2	431-491	Step-wise				

15	CA268640	UGSuM34a	(AAG)10	CDS	TTACAAATGTAGCCTTGCCTTG	ATCTTTCCTTGCTTGCCTCTC	Soluble acid invertase	61	2	19	99-178	Mixed	1	P	P	0.83
														
		UGSuM34b								4	333-351	Step-wise				

16	CA131350	UGSuM41	(CCG)10	CDS	ATCATTCTCCATCATTTCTCA	AGGCTCTTCAACCGTGCT	Unknown protein	54	1	20	120-210	Step-wise	0	P	P	0.80

17	CA133924	UGSuM42a	(CTCTCC)5	5'UTRs	TTCATACAGAAGAACCTCCAC	TCCATCAGAGACAAGCAGA	Auxin-independent growth promoter	54	3	12	130-212	Mixed	1	P	P	0.83
														
		UGSuM42b								8	325-367	Step-wise				
														
		UGSuM42c								2	475-487	Step-wise				

18	CA136599	UGSuM43a	(CCG)10	3'UTRs	CAAAGTGCTGTAGGGCTG	TTCAATGGGTGATAAGTGTGT	Ribose-phosphate pyrophosphokinase 1	55	2	17	90-187	Mixed	1	P	P	0.85
														
		UGSuM43b								2	332-368	Step-wise				

19	CA139800	UGSuM44a	(CT)15	5'UTRs	TCCATCAAGCCGTTCCTC	GCCAAGCAGATAAAGAAGTG	Rudimentary enhancer	55	2	16	220-308	Step-wise	1	P	P	0.84
														
		UGSuM44b								1	419	-				

20	CA171090	UGSuM45a	(AAAAG)6	CDS	ATCTCCTCTTATTCGTTCTGG	AGCAGCGTCTTATCTGGG	PAP fibrillin	56	3	5	91-141	Step-wise	1	P	P	0.79
														
		UGSuM45b								2	267-287	Step-wise				
														
		UGSuM45c								2	368-393	Step-wise				

21	CA196477	UGSuM46a	(GAC)10	CDS	ACTCCTCCCGCCTCCACTAC	CTCACCGAAGCAATCAAG	Hypothetical protein	60	2	15	101-188	Mixed	1	P	P	0.81
														
		UGSuM46b								3	341-377	Step-wise				

22	CA228772	UGSuM47a	(GCC)10	CDS	ATTTATGGAGGAAGAAACGG	ATTACAAACAAGAAGAGCGG	Transport protein particle component	55	2	3	82-97	Step-wise	1	P	P	0.80
														
		UGSuM47b								14	215-270	Mixed				

23	CA161416	UGSuM50	(TC)14	CDS	CTACTGCCGAGGAAAGATCG	GGAAAAGTTTGTGGCAAGGA	Hypothetical protein	58	1	16	94-188	Step-wise	0	P	P	0.80

24	CA228375	UGSuM73a	(CGC)8	5'UTRs	CTTTCAACCTCTACACCTCCAC	ACTAGAAGACTGAGAAGAACCAGT	40 S ribosomal protein S11	55	2	13	101-186	Mixed	1	P	P	0.82
														
		UGSuM73b								5	354-375	Step-wise				

25	CA261182	UGSuM74	(ACA)8	CDS	TCAGCAGCTGTGAAGTTTCATT	CGTCTCTTTTGGGTTTCATCTC	Transcription regulator protein	55	1	18	190-271	Step-wise	0	P	P	0.81

26	CA219230	UGSuM75a	(TA)12	CDS	TTGTGCTGATGTTTCCTGCT	CAAGAGAAGATGCCATTAGCC	Patatin-like protein	55	2	13	94-176	Step-wise	1	P	P	0.83
														
		UGSuM75b								4	333-351	Step-wise				

27	CA093071	UGSuM96a	(AT)11	CDS	TCAAACCAGGATCTAAGCTCAC	GGTAGTGCCATTGAGGTTGC	Putative apyrase	57	2	11	205-290	Mixed	1	P	P	0.81
														
		UGSuM96b								5	408-458	Step-wise				

28	CA229840	UGSuM97a	(GA)11	5'UTRs	GCGAGAGAGATAGAGGGAGAGA	AGGTGCCGTTCATGAGGTAGT	Glycine decarboxylase	56	2	19	240-334	Step-wise	1	P	P	0.85
														
		UGSuM97b								2	453-469	Step-wise				

29	CA112979	UGSuM149a	(AGC)7	CDS	GTTCAATCAAATCCCTCTCCTC	AGCTTGGTCAGCTCCTCATCGTT	Ubiquitin-specific protease 4 (UBP4)	60	2	4	104-141	Mixed	0	P	P	0.78
														
		UGSuM149b								3	276-297	Step-wise				

30	CA116368	UGSuM150a	(GGC)7	CDS	ACACTGACCGATGGATCCTCTT	ATCAACGTGGACCAGATCTTCTT	hAT family dimerisation domain protein	60	2	12	90-167	Mixed	1	P	P	0.76
														
		UGSuM150b								2	279-291	Step-wise				

31	CA280782	UGSuM177a	(TGC)7	CDS	GGTGCTGTCCCTATCACTAC	GCCCTTGTTTCTTTGTCTACT	Hypothetical protein	55	2	3	150-167	Mixed	0	P	P	0.79
														
		UGSuM177b								3	287-314	Step-wise				

32	CA291445	UGSuM178	(GAC)7	5'UTRs	GGACTACTACGACTACTGCGA	ACCTTGCTTACATCTTCCTCT	O-diphenol-O-methyl transferase	54	1	15	100-196	Step-wise	0	P	P	0.80

33	CA244023	UGSuM186	(AG)10	CDS	AACATTTCGGCATTTGAAGC	GGTCTTTCTTGGGGATCTCTC	Ubiquitin C-terminal hydrolase	56	1	4	190-232	Step-wise	0	P	M	0.73

34	CA231668	UGSuM187a	(CT)10	CDS	CAACAATTGTCGAAGCCTCTC	TTTGCTTACCCCCTGTTGAC	ATP synthase	58	2	10	200-264	Step-wise	0	P	M	0.83
														
		UGSuM187b								6	376-418	Step-wise				

35	AY596560	UGSuM188	(GA)10	3'UTRs	CCCAAGCGAGCTAGAGAGAG	TCTTCTTTCCTTCGCACAGC	Hypothetical protein	55	1	19	95-186	Mixed	0	P	P	0.81

36	CA241232	UGSuM189a	(CT)10	CDS	CCGCGACTCTCCTCTCTCT	GTTCTTCTCGGCGTTCCTC	Auxin-regulated protein	55	2	15	106-200	Step-wise	1	P	P	0.80
														
		UGSuM189b								3	345-377	Step-wise				

37	CA133642	UGSuM196a	(AAAG)5	5'UTRs	GCTACTATGGACAACAGGG	ATGAAGAGACGAGACGAAGA	Cinnamoyl CoA reductase	54	2	18	90-181	Mixed	1	P	P	0.81
														
		UGSuM196b								2	373-393	Step-wise				

38	CA134472	UGSuM197	(GA)10	3'UTRs	GAAGGAGCAGCAGCGCCAGT	GATTTGCCGTCCTAGGGTTT	Epsin N-terminal homology domain	56	1	22	200-290	Step-wise	0	P	P	0.84

39	CA297648	UGSuM343a	(CAG)6	5'UTRs	ACTCCTCCTCCTCGCCGT	TCTTGTTGTAGTAGCCCTTGT	SOUL heme-binding family protein	62	2	17	250-320	Mixed	1	P	M	0.87
														
		UGSuM343b								3	438-450	Step-wise				

40	CA300679	UGSuM344	(CTC)6	CDS	CTATCCTCTTGTTGGGTCCT	TCCGCACCTCCGTTCACC	Nucleoside diphosphate kinase protein	55	1	1	260	Step-wise	0	M	M	0.0

41	CA084691	UGSuM345	(TC)8	CDS	TATACAAGAATGAAAGGTGAGAGA	AAGCATACTCCCTCTATCTCTATG	DC1 domain-containing protein	55	1	2	210-220	Step-wise	0	M	P	0.70

42	CA093455	UGSuM346	(AG)8	CDS	TATACGTAGTAGTGATGATGACCG	CTCCTTCGTCCAGTACCAGTAG	DNA-binding protein DF1	60	1	4	150-180	Step-wise	1	P	M	0.71

43	CA110745	UGSuM347a	(CT)8	5'UTRs	TCTGGCTTTATCGTAACTTGTAT	GAGCCTCGTTTGGGTGGCTTTC	Expressed protein	55	2	5	230-254	Step-wise	1	P	M	0.74
														
		UGSuM347b								1	365	-				

44	CA112979	UGSuM348	(CG)8	CDS	CTACCTCCTCGTCTCCTCCCTCTT	AACAAGGAATATGGTCCCTGAG	Unknown protein	61	1	2	240-261	Mixed	0	M	M	0.0

45	CA116458	UGSuM349a	(TC)8	CDS	CAAGATGTACCCGGACATGGCT	TGCTATACTAGCTATCTCCTTCCT	Unknown protein	55	2	3	220-236	Step-wise	0	P	M	0.73
														
		UGSuM349b								2	385-395	Step-wise				

46	CA123971	UGSuM464	(ACA)5	5'UTRs	GGCTACTTCAGACACGCA	TCTACGCATCAACCTCTCA	SNF2-domain-containing protein	55	1	1	220-232	Step-wise	0	M	M	0.0

47	CA125310	UGSuM465	(GCA)5	CDS	GCTAACCAACATCAGCAGT	AGGAGATTGACGAAGAAGAAG	Transducin family protein	53	1	2	260-279	Mixed	0	M	M	0.0

^A^UGSuM stands for unigene derived sugarcane microsatellite primers as indicated in the Additional file 3

^B^Polymorphic (P) and Monomorphic (M)

**Figure 2:**
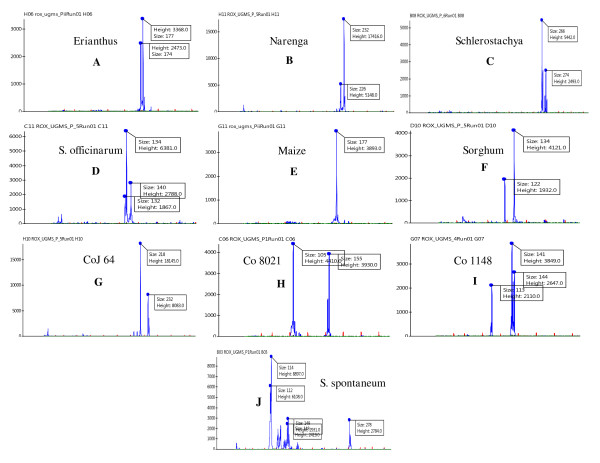
**Different UGMS allele types detected by automated fragment analysis**. Distribution of various allele types detected in *Saccharum *species, genera, commercial varieties and five cereal species using the automated fragment analysis. A, C, D, F and H: Multiple alleles in a single locus showing step-wise distribution, B, G and I: Multiple alleles in a single locus showing mixed distribution, E: Single allele in a unique locus, and J: Multiple loci showing both stepwise and mixed distribution in different loci.

**Figure 3:**
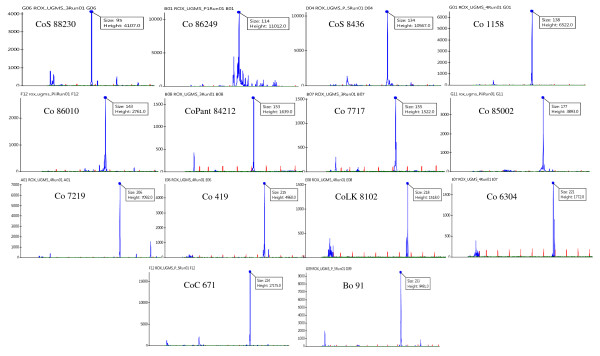
**Fragment length polymorphism detected among Indian sugarcane varieties using fluorescent dye labeled primers**. Allelic variation in a representative set of 14 commercial Indian tropical and sub-tropical sugarcane varieties. Fourteen alleles were amplified by a class I UGMS marker (UGSuM17) designed from the unigene encoding Abcisic acid inducible protein kinase that contained (AG)_18 _repeat-motif. The amplicons generated by the fluorescent dye labeled primers were resolved in MegaBACE automated DNA sequencer and analyzed in Fragment Profiler software. Fragment size (bp) and average peak height for all the amplified alleles are indicated.

Due to high polyploidy and heterozygosity of the *Saccharum *genome, most (28, 65.1%) of the primers amplified two to three different loci (each locus being designated as an UGMS marker) yielding a total of 60 loci with the number of alleles per locus ranging from 2 to 22 and the allele size varying from 82 to 408 bp across sugarcane species, genera and varieties (Figure 2) as well as five cereal species. The remaining 15 (34.9%) primers gave single locus amplification (Table 2) with 1 to 23 alleles each. Overall, the 43 polymorphic primers amplified an average of 7.42 alleles per UGMS marker locus with a total of 722 alleles across 74 loci. Forty-two (73 UGMS marker loci with 720 alleles, 97.7% polymorphic, mean PIC of 0.81) of the 43 informative primers revealed polymorphism between sugarcane species and related genera, whereas 37 (66 UGMS marker loci with 678 alleles, 86% polymorphic, PIC of 0.74) primers detected polymorphism among the Indian commercial sugarcane varieties (Figure 3). Twenty-six (34.7%) of the 75 UGMS marker loci showed heterozygosity in different genotypes used. Maximum heterozygous loci was detected in five sugarcane species (14, 18.7%) followed by commercial varieties (9, 12%) and minimum (4, 5.3%) in the three related genera and five cereal species.

### Molecular basis of UGMS polymorphism and its functional significance

**Figure 4:**
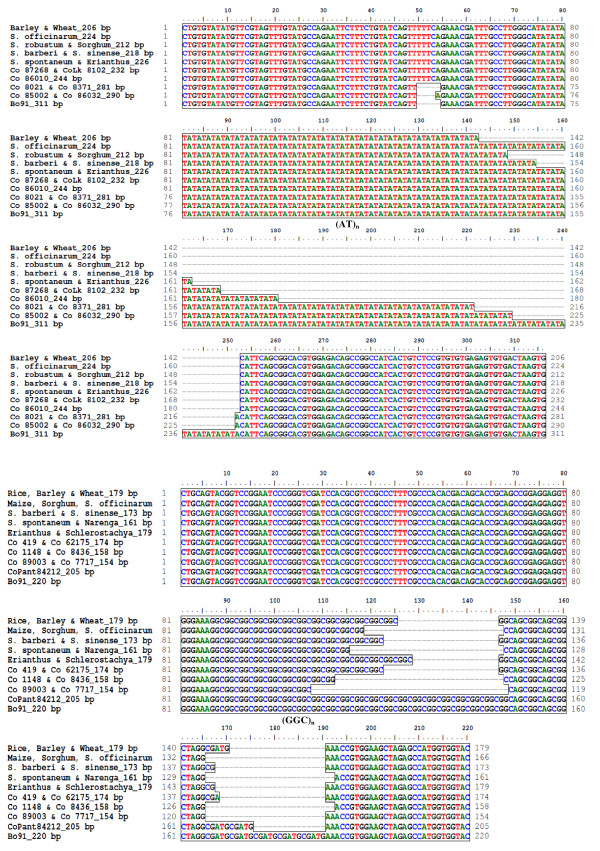
**DNA sequence alignment depicting the molecular basis of UGMS fragment length polymorphism among *Saccharum *complex, varieties and five cereals**. Multiple sequence alignment of 10 size variant alleles showing both stepwise and mixed distributions amplified from sugarcane species, genera, varieties and five cereals for the two UGMS marker loci namely, UGSuM2 (A) and UGSuM27 (B). Variation in the number of repeats of (AT)_n _and (GGC)_n _microsatellite motifs at UGSuM2 and UGSuM27 loci, respectively and additional nucleotide insertions/deletions in the flanking sequences of the repeats are highlighted.

Comparison of fragment size (bp) of variant alleles amplified at 75 polymorphic UGMS loci with changes in number of repeats among *Saccharum *complex, varieties and five cereal species revealed purely step-wise allelic distribution for 51 (68%) loci, while remaining 24 (32%) loci showed mixed type of allele size distribution (Figure 2). To elucidate the distribution pattern of UGMS alleles showing fragment length polymorphism, 10 amplified size variant alleles each of two microsatellite primers namely, UGSuM2 and UGSuM27 showing both step-wise and mixed type of allele distribution were sequenced. High quality sequence alignment revealed that the size variant alleles showing step-wise distribution contained the expected microsatellite repeat sequences with conserved primer binding sites, but corresponded exactly to the step-wise multiples of the number of repeat units. The size variation of sequenced alleles was explained by differences in the number of repeat units. Mixed type of allele distribution resulted from both variation in the number of repeat-units and insertions/deletions in the sequences flanking the UGMS repeats. For instance, UGSuM2 (Figure 4A) and UGSuM27 (Figure 4B) showing both step-wise and mixed type allele size distribution had the expected (AT)_n _and (GGC)_n _UGMS motifs, respectively with varying number of repeat-units in different sugarcane species, genera, varieties and cereals along with a small stretch of nucleotide insertion/deletion in the flanking regions of target microsatellites.

**Figure 5:**
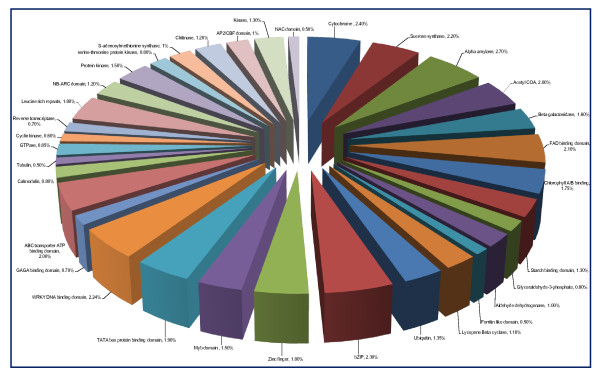
**Functional annotation of unigenes carrying microsatellites in their functional domains**. Functional annotation of 364 unigenes carrying microsatellites in the functional domains of encoded proteins. These unigenes corresponded maximum (47.7%) to the domains responsible for photosynthesis (cytochrome b/c and chlorophyll A/B binding domains) and carbohydrate metabolism (sucrose synthase and alpha amylase domains) followed by transcription factor associated basic leucine zipper, zinc finger, TATA box, Myb and WRKY DNA binding domains (22%), and minimum to the abiotic and biotic stress related leucine rich repeat, protein kinase and chitinase domains (5%).

In order to assess the functional significance of the UGMS, gene ontology classification of 767 unigenes carrying perfect and compound microsatellites was carried out. Six hundred seventy-two (87.6%) of these (see Additional files 3 and 4) could be functionally annotated and were shown to encode enzymes for sugar metabolism (39%), structural proteins (26%), transcription and translation factors (18%), signal transduction pathway proteins (8%), cell growth and development factors (5%) and disease resistance proteins (4%) (see Additional file 7). Three hundred sixty-four (54.2%) of 672 unigenes contained microsatellite repeats in 89 different functional domains of proteins encoded by these unigenes (see Additional file 8) which included cytochrome b/c oxidase, chlorophyll A/B binding, FAD and ubiquitin binding, sucrose synthase, alpha-amylase, acetyl COA, aldehyde dehydrogenase, glyceraldehyde-3-phosphate, lipase, S-adenosylmethionine, leucine rich repeat (LRR), ferritin, protein kinase, chitinase, basic leucine zipper (bZIP), zinc finger, TATA box, Myb and WRKY DNA binding domains (Figure 5). Fifteen primers designed from 15 different unigenes targeting such functional domains (see Additional file 9) that gave single locus amplification and step-wise allele distribution (Table 2) were selected for validation. All of these primers gave fragment length polymorphism among *Saccharum *complex, varieties and five cereals based on variation in the number of UGMS repeat-units within the functional domains.

**Figure 6:**
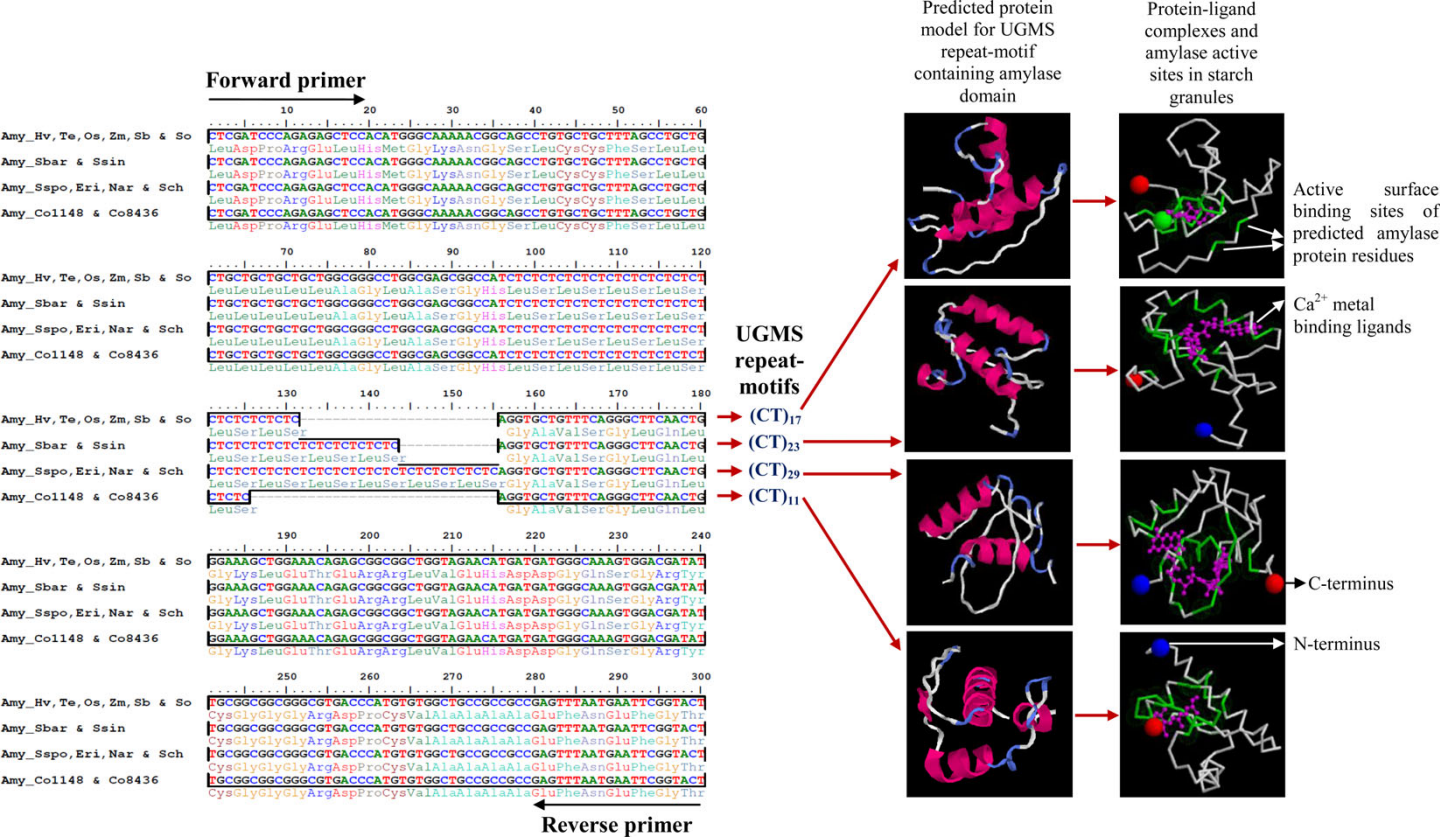
**Alignment, and predicted protein structure and catalytic domain binding sites depicting the functional relevance of microsatellite carrying unigenes**. Multiple sequence alignment of the four size variant alleles showing step-wise distribution in the amylase catalytic domain amplified from sugarcane species, genera, varieties and five cereals using primer (UGSuM26) for β-amylase unigene. Variation in the number of repeat-units of CT and the encoding repeated tracts of Leucine-Serine aminoacid residues at a microsatellite locus predicted different three dimensional protein structures and protein-ligand complex binding sites which are highlighted.

One of these primers (UGSuM26) showing polymorphism targeted the amylase catalytic domain of β-amylase (Figure 6). To understand the possible biological significance of the variable UGMS repeat in the amylase domain, the structure predicted from the aminoacid sequences of the functional domain was analyzed *in silico*. It revealed variation in the active binding site involved in formation of the Ca^2+ ^ligand complexes due to the presence of variable number of repeats encoding Leucine-Serine aminoacid residues (Figure 6). This would most likely alter the function of amylase gene in respect of carbohydrate metabolism in sugarcane. Similarly, the expansion/contraction of microsatellite motifs (AG)_n _encoding Arginine-Glutamine aminoacid residues was observed in the protein kinase functional domain of another sugarcane unigene which was amplified by the primer UGSuM17. *In silico *analysis suggested a novel secondary protein structure and catalytic domain binding site in the variant form that was different from the native form which contained (AG)_18 _microsatellite repeats (see Additional file 10).

### Assessment of functional genetic diversity

The pair-wise similarity among 36 genotypes belonging to *Saccharum *species, related genera and 28 tropical and sub-tropical Indian varieties of sugarcane based on 43 fluorescent dye labeled polymorphic primers revealed a broad range from 0.12 to 0.91 with an average of 0.32 similarity index. The similarity among the sugarcane species varied from 0.24 (IJ-76-3-1-9 and 1151) to 0.88 (IJ-76-3-1-9 and IM-76-256) with an average of 0.36. Among the species, *S. officinarum *showed maximum similarity (0.88) with *S. robustum *followed by *S. barberi *with *S. sinense *(similarity of 0.82). *S. spontaneum *was most divergent from the rest of the *Saccharum *species. The similarity of the three genera of sugarcane, namely, *Erianthus*, *Narenga *and *Sclerostachya *with the sugarcane species varied from 0.22 to 0.90. *Erianthus *had the least similarity with the *Saccharum *species. Similarity among the 28 Indian tropical and sub-tropical sugarcane varieties varied from 0.33 (Bo 91 and Co 8021) to 0.84 (Co 8371 and Co 8021) with an average of 0.40. The pair-wise similarity between the sugarcane species and varieties varied from 0.20 to 0.78 with average of 0.35, while between the three related genera and varieties, it ranged from 0.13 to 0.80 with an average of 0.24. The average similarity between the sub-tropical and tropical varieties (0.40) was slightly lower than the average pair-wise similarity measures within the sub-tropical (0.47) and tropical varietal groups (0.43).

**Figure 7:**
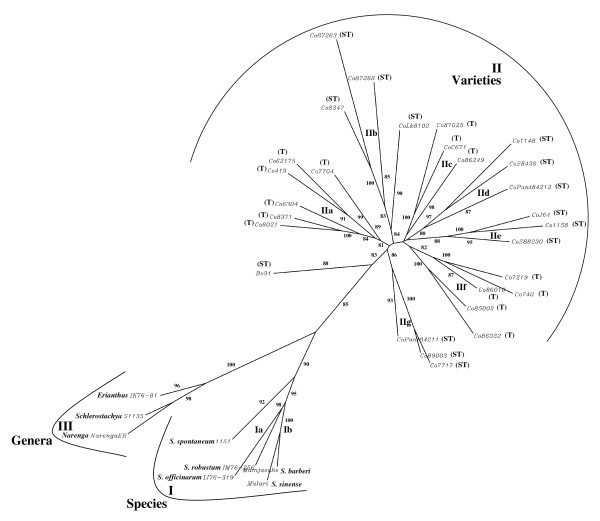
**Phylogenetic tree depicting genetic relationships among *Saccharum *complex and Indian varieties**. Unrooted phylogenetic tree depicting the genetic relationships among the *Saccharum *species, related genera and 28 commercial Indian tropical and sub-tropical sugarcane varieties based on Nei and Li's similarity coefficient using 47 fluorescent dye labeled primers. Bootstrap values are indicated at the corresponding node for each cluster. Molecular classification corresponded to the known evolutionary and pedigree relationship as well as the adaptive environment. T and ST denote the tropical and sub-tropical region of adaptation, respectively.

The genetic relationship among the *Saccharum *species, related genera, and tropical and sub-tropical varieties is depicted in Figure 7. The UGMS markers clearly discriminated all the 36 genotypes from each other and resulted in a definitive grouping among different genera and species of *Saccharum *with high bootstrap values (81 to 100) that corresponded well with their known pedigree relationships. All the clones of five *Saccharum *species were included in the major cluster I in which *S. officinarum *and *S. robustum *clones were sub-clustered (Ia) with 98% occurrence in boot strap analysis; *S. barberi *and *S. sinense *clones grouped together in a separate sub-cluster (Ib) with 100% occurrence and *S. spontaneum *grouped distinctly from these sub-clusters with 92% occurrence. The three related genera of *Saccharum *being highly divergent from the *Saccharum *species and varieties, grouped in a separate cluster (III) with 98% occurrence.

All the tropical and sub-tropical varieties were included in a distinct and separate cluster (II) with seven distinct sub-clusters (Figure 7) with high (88) bootstrap value. The tropical variety Co 62175 grouped in a major sub-cluster IIa with its tropical male parent Co 419 (similarity 0.71) with a bootstrap value of 99%. In this sub-cluster (IIa), the other tropical varieties namely, Co 8021, Co 8371, Co 7704 and Co 6304 were also grouped together with average similarity of 0.77 (supported by 93% occurrences in bootstrap) possibly because of their common ancestry involving Co 419. The two sub-tropical varieties, Co 8347 and Co 87263, and three other sub-tropical varieties, CoPant 84211, Co 89003 and Co 7717, in spite of having a common ancestor Co 775, were included in two distantly placed major sub-clusters; IIb and IIg, respectively. It could be due to inclusion of two diverse sub-tropical varieties, Co 87268 and CoLk 8102 having common parent Black Cheribon within the sub-cluster IIb. Similarly, the clustering of two tropical varieties namely, CoC 671 and Co 87025 in the sub-cluster IIc with average similarity of 0.69 (supported by 100% occurrences) and five tropical varieties, Co 7219, Co 740, Co 86010, Co 86032 and Co 85002 together in another separate large sub-cluster (IIf) with average similarity of 0.64 (supported by 92% occurrences) was observed even though they had one common distant progenitor parent Black Cheribon. The grouping of sub-tropical varieties namely, Co 1148, CoS 8436 and CoPant 84212 in cluster IId with an average similarity of 0.70 (88% occurrences in bootstrap) was clearly influenced by a common ancestry involving Co 1148. The sub-tropical varieties, CoJ 64, Co 1158 and CoS 88230 included in the cluster IIe had common progenitor parent PoJ 2878 that could have influenced their clustering with a high bootstrap value (94.3%).

## Discussion

Unigene resources representing the transcriptome of an organism provide opportunities to understand the sequence organization in the genic regions of complex polylploids and allow design of sequence based robust markers for various genotyping applications. Considering the availability of a large unigene database of sugarcane in public domain, this resource was studied for the presence and functional relevance of different microsatellite repeats. The frequency of microsatellites in sugarcane unigenes (1/10.9 kb) was lower than that obtained in rice, *Sorghum*, barley and maize (1/3.6 kb, 1/5.9 kb, 1/8.9 kb and 1/9 kb, respectively) but similar to wheat (1/10.6 kb). In polyploids, extensive loss of duplicated genes and chromosomal rearrangement after polyploidization possibly have resulted in shortening and loss of microsatellite repeat-motifs from the genic coding sequences leading to dosage compensation, developmental stability and functional plasticity [16,17]. It would be of interest to explore the mechanism that restricts repeat expansion in genic regions of wheat and sugarcane leading to lower microsatellite frequency in these species.

The observed frequency of the mononucleotides (12%) in sugarcane was much less than that reported earlier [18] in the unigenes of maize (75.8%), wheat (71%), barley (42.4%) and rice (41.6%), but similar to *Sorghum *(13%). This suggested a lack of correspondence with genome size and ploidy. The presence of longer (69 bases or more) A/T mononucleotide repeat-motifs in sugarcane is comparable to earlier observations on the nature and the frequency of such repeats in the genomic and EST sequences of cereals [19]. The abundance of trinucleotide microsatellite motifs in the sugarcane unigenes is consistent with the earlier observations based on EST sequences of sugarcane [3,5] and unigene sequences of cereals [18]. The limited expansion of non-triplet microsatellites in the unigenes of sugarcane could be due to selection against frameshift mutations in the coding regions resulting from length changes in non-triplet repeats. We observed that more than 50% of the identified trinucleotide repeat motifs in sugarcane were GC-rich, possibly due to their high GC content [3,5] and consequent usage bias in the coding sequences [20]. The abundance of GC-rich trinucleotide repeat motifs coding for small and hydrophilic aminoacid alanine and GA-rich dinucleotide motifs in sugarcane coding sequences parallels their abundance in cereal exons [18]. The GA-rich dinucleotide UGMS motifs were observed particularly in the 5' and 3' (32%) UTRs with balanced (46 to 52%) GC content and therefore would support better amplification as polymorphic markers [21].

The unigenes being longer in higher quality sequences offer advantages over the EST sequence resources for the development of microsatellite markers. In the present study, 961 (810 perfect and 151 compound) primer-pairs were designed from the 767 different unigene sequences carrying microsatellite repeats in the expressed component of the sugarcane genome. The primers designed from the unigene sequences flanking the microsatellite motifs were highly efficient with amplification success rate of 94.9% that suggested the utility of the unigene database in designing sequence based robust genic markers. The paucity of usable and robust sequence based markers in sugarcane has been a major limitation in genetic analysis in this important sugar crop. The UGMS markers developed by us have been placed in the public domain and thus would be immediately useful in various genotyping applications in sugarcane.

For most (87.6%) of the unigene sequences from which the primers were designed, putative functions have been predicted. For instance, about 39% and 4% of the primers were from sequences related to sugar metabolic enzymes and disease resistance, respectively. Interestingly, 54% of the microsatellite repeats were present in various functional domains of proteins encoded by the unigenes. Correlation between fragment length polymorphism due to variable number of UGMS repeat-units in the functional domains and alteration of the predicted protein structure and active ligand binding sites suggested functional relevance of the genic microsatellites. The evolutionary and adaptive advantages of such variable microsatellite repeats affecting the structure and function of the encoded proteins to generate favorable alleles for relaxation of environmental stress impact under the action of high natural selection pressure through modulation of mutation/recombination in these loci have been reported in many eukaryotes [22]. For instance, in *Saccharomyces cerevisiae*, the expansion and contraction of microsatellite repeats in the coding regions of protein kinase genes have provided greater adaptability to various abiotic and biotic stresses [22]. Sugarcane is a tropical crop. However, varieties adapted to subtropical condition have been developed in India. Variation in microsatellites in the coding regions of these two groups was evident. For example, the tropical sugarcane varieties Co7219 and Co740 contained (AG)_18 _microsatellite repeats whereas varieties Co89003 and Co7717 adapted to sub-tropical condition showed contraction of AG repeats to (AG)_10 _leading to alteration of protein structure and possibly function. Understanding the adaptive significance of such variation is of relevance that needs further experimentation. Designing of genic microsatellite markers targeting functional domains in solanaceous crops like tomato and pepper [23] was reported. Association of such markers with many traits including diseases like neuronal disorders [22] and cancers [24] in humans based on expansion/contraction of repeated tracts of microsatellites encoding aminoacid residues in the functional domains of proteins that changes their secondary structure and function has been demonstrated. The information generated in this study thus would allow selection of candidate gene-based markers for rapidly establishing marker-trait linkages in sugarcane.

The efficiency of sugarcane UGMS markers to detect polymorphism within *Saccharum *complex and among five cereals was evaluated *in silico *that was further validated experimentally. *In silico *polymorphism based on variation in length of the UGMS repeat among *S. officinarum *and each of five cereal species could be due to divergent microsatellite evolution in these lineages [25]. The actual level of polymorphism detected by automated fragment analysis using fluorescent dye labeled primers was much higher than that based on *in silico *analysis. The expected relationship in evolution was reflected by actual polymorphism observed between sugarcane and cereals. With the use of automated fragment analysis system, all the allelic variants could be captured efficiently in a set of 36 sugarcane genotypes and five cereal species, thereby providing a platform for rapid, automated and large-scale genotyping of sugarcane. Further, the allele size information generated would enable multiplexing of the UGMS markers, thus making them useful in various high-throughput genotyping applications. The observed polymorphism (97.7%) among the members of the *Saccharum *species and related genera is higher than that estimated earlier using the fluorescent dye labeled sugarcane genomic (90%, [2]) and EST derived microsatellite markers (81%, [3]). The higher potential of UGMS markers particularly the longer class I di- and tetra-nucleotide repeat-motifs to detect polymorphism as compared to the class I trinucleotide and class II motifs reflected correspondence between the type and length of repeats, and the level of polymorphism as observed earlier in sugarcane [4] and rice [21,26]. Higher polymorphic potential of UGMS markers derived from the UTRs than that from the conserved coding sequences, which are constrained by purifying selection [25,27] suggested the utility of unigenes having such repeat-motifs as a source of polymorphic microsatellite markers.

The level of inter-varietal polymorphism (86%, mean PIC of 0.74) detected by the fluorescent dye labeled primers was higher than the level reported previously with the labeled sugarcane EST derived (38%, PIC of 0.23, [3]) microsatellite markers. The discrepancies could be due to use of different sets of markers and genotypes. However, genotyping using the automated fragment analysis system in this study revealed comparable level of polymorphism with the genic and genomic microsatellite markers in sugarcane. It suggested that the genic microsatellite markers developed in this study would be highly informative and useful in sugarcane genetics, genomics and breeding.

Unigene sequences usually have advantages of unique identity and position in the transcribed regions of the genome. If this is the case then primers designed from the unigene sequences flanking the microsatellite repeats should amplify unique single locus. In contrast, in the present study, multiple loci and thus amplification of multiple sequences of the same gene was observed for 28 (65.1%) of the 43 primers designed from different microsatellite carrying unigenes. This is possibly due to poor representation of unigene sequences in the database that was scanned for microsatellite repeats. Alternatively, all the copies of a microsatellite carrying gene that are PCR amplified might not be transcribed due to dosage compensation leading to silencing of all but one copy in the large polyploid sugarcane genome [16,28]. In spite of high polyploidy and heterozygosity of the sugarcane genome, 15 (34.9%) of the 43 polymorphic UGMS primers amplified a single discrete locus with step-wise allele distribution and showed fragment length polymorphism across the members of *Saccharum *complex and varieties in an automated fragment analysis system. This could be due to polyploidization followed by selective gene loss [29,16,28] resulting in retention of a single copy during evolution of *Saccharum*. Gene ontology analysis of these 14 unigenes showed correspondence with the genes coding for basic metabolic process of energy generation, degradation of cellular building blocks, DNA recombination and repair proteins which played a vital role in plant biology and thus remained as single copy without alteration.

Distribution pattern of size variant alleles amplified at UGMS loci showed higher proportionate (68%) distribution of alleles showing step-wise mutation than that of mixed allele distribution (32%). High-quality sequence alignment of the size variant alleles showing both step-wise and mixed distributions confirmed the presence of variable number of repeat-units in different amplified alleles and additional insertions/deletions in the flanking regions of microsatellite repeat-motifs, which contributed to the UGMS fragment length polymorphism observed in the *Saccharum *complex, varieties and cereal species. Such complex pattern of allele distribution and fragment length polymorphism at microsatellite loci due to variation in the copy number of microsatellite repeats and insertions/deletions in the flanking sequences of microsatellite motifs have been observed earlier in species like maize with a large genome size [30].

It is important to evaluate molecular diversity existing among the members of the *Saccharum *species and related genera, since major varietal improvement in sugarcane was through inter-specific hybridization. Use of *S. spontaneum *and *Erianthus *is vital since they carry many traits of agricultural importance including tolerance to biotic and abiotic stresses [14]. Identification of true inter-specific and inter-generic hybrids is crucial for successful transfer of the target traits. Besides, monitoring of introgression is required to verify the transfer of the target genomic regions from the wild relatives to the cultivated genetic backgrounds. 97.7% of markers revealed inter-specific and inter-generic polymorphism and thus would enable precise identification of inter-specific and inter-generic hybrids, and also provide opportunities to assess transfer of genic regions to desirable genetic backgrounds, thereby offering advantages over the random markers such as RAPD and AFLP.

Evaluation of molecular diversity in a set of 28 commercial Indian tropical and sub-tropical sugarcane varieties using unigene based genic microsatellite markers revealed a wider range (0.33 to 0.84 with an average of 0.40) of genetic similarity than the level detected previously with RAPD (0.59 to 0.81 with average of 0.71, [12]), maize microsatellite (0.40 to 0.73 with average of 0.64, [13]), sugarcane genomic microsatellite (0.39 to 0.82 with average of 0.53, [4]) and AFLP (0.52 to 0.83 with average of 0.62, [14]) markers. Hence, high efficiency of UGMS markers in assaying functional diversity in the transcribed component of the sugarcane genome makes them valuable for understanding diversity pattern, and variety identification. The commercial Indian sugarcane varieties used in this study were bred for either tropical or subtropical agro-climatic conditions with differential contribution of *S. spontaneum*, the most variable *Saccharum *species [14]. The random markers assaying genetic variation largely in different non-genic genomic regions that contribute to large genome size [31] would be of little relevance to phenotypic selection exercised during variety development. In contrast, the genic microsatellite markers assaying variation in the transcribed non-repetitive regions of the genome might be directly related to the phenotypic variation [25]. Our results thus suggested that the genetic base of the Indian sugarcane varieties is not very narrow particularly in the genic regions of the genome, which is most likely due to selection for wider adaptability of these varieties. The adaptive environment as well as parentage of these varieties corresponded well with the clustering pattern obtained using a small set of these markers, that further suggested the usefulness of the designed markers in realistic assessment of genetic diversity.

## Conclusion

The present study identified microsatellites in sugarcane unigenes and assessed their functional relevance. A total of 961 primer-pairs were designed targeting 767 different unigenes carrying microsatellite repeats in the expressed component of the sugarcane genome, which would extend the accessibility of such microsatellite markers to researchers for many genetic studies in sugarcane. Precise allele sizing in automated fragment analysis system has encouraging implications to various high-throughput genotyping applications in sugarcane. Assessment of functional genetic diversity revealed that the genetic base of the Indian sugarcane varieties is not narrow.

## Methods

### Mining of microsatellites in the unigenes of sugarcane and assessment of their functional relevance

Fifteen thousand five hundred ninty-four sugarcane (*Saccharum *sp.) unigenes (Build 13.0, 19^th ^Feb' 2008) comprising of 9.17 million base sequences were acquired in FASTA format from the recent NCBI FTP UniGene repository database of *S. officinarum *[32] in batches and searched for microsatellites using a perl scripting language based program MISA (MIcroSAtellite) [33] considering complementary sequence of repeat-motifs (like AG, GA, TC and CT) in the same class [18]. The identified microsatellites were characterized as perfect (monomers to hexamers repeated up to 100 times without any interruption), compound (non-interrupting) with at least two different repeat-motifs without any interruption, compound (interrupting) with a maximum of 100 nucleotides interposing two microsatellite repeat-motifs, class I (≥20 nucleotides) and class II (12 to 20 nucleotides) types. The microsatellite (excluding monomers) containing unigenes were used in a batch module for designing forward and reverse primers employing the microsatellite primer discovery tool of BatchPrimer3 [34]. All default options except for the optimum and maximum primer sizes of 22 and 24 nucleotides, respectively, were used. The putative functions for these unigenes were determined using NCBI BLASTX search tool [35] as against the nr-protein database. The BLASTX output was annotated into four functional categories *viz*, exact, putative, unknown and hypothetical, and then extracted to Excel sheets. Different structural components namely, coding and untranslated regions (5'UTRs and 3'UTRs) in the unigene sequences was determined using the software tools FGENESH [36] and UTRScan [37], respectively. The aminoacid sequences encoded by the predicted coding nucleotide regions of the microsatellite carrying unigenes were analyzed using the software Pfam [38] to determine the presence of functional domain/protein family within the UGMS. The aminoacid sequences of the functional domain carrying microsatellites was analyzed further using the I-TASSER automated web server [39,40] for prediction of three dimensional protein structure and active binding sites with ligands. Five different protein models and active binding sites were predicted at significant cut-off confidence (C ≥ -1.5) and binding site (BS ≥ 0.5) scores. The high quality protein model of correct topology and protein-ligand complex active binding sites was identified based on high C- and BS- scores.

### Evaluation of polymorphic potential

To assess the amplification success rate of microsatellite markers designed from sugarcane unigenes, 176 primers were used to amplify one genotype of *S. officinarum*. Forty-seven, including thirty-eight class I and nine class II markers of these, labeled with 6-carboxyflourescein (6-FAM) dye phosphoramidites, were used to genotype one accession each belonging to five *Saccharum *species and three related genera, and twenty-eight commercial Indian tropical and sub-tropical sugarcane varieties (Table 3) for evaluating their polymorphic potential and assessment of genetic diversity. Five cereal species namely, rice, wheat, maize, *Sorghum *and barley were included in the experiment for comparison. Standard PCR constituents and cycling conditions except for annealing temperature, which varied from 55°C to 64°C depending on the primers, were used for PCR amplification. The amplified products were purified, mixed with 3.75 μl of MegaBACE formamide loading buffer and 0.25 μl of internal-lane MegaBACE™ET 550-R ROX size standard, denatured, cooled and resolved in automated MegaBACE 1000 DNA sequencer (Amersham Biosciences, Piscataway NJ, USA). The electropherogram containing trace files were analyzed and automated allele calling was carried out using the Binning Peak Post Processor tool of MegaBACE™Fragment Profiler Software Version 1.2 (Amersham Biosciences, Piscataway NJ, USA). The actual allele size (bp) was determined and fragment length polymorphism among the sugarcane genotypes and cereal species was identified. The average peak height and peak quality of alleles generated for all the UGMS loci were graphed and alleles exhibiting average peak height and quality of ≥ 1500 and ≥ 6.0 fluorescence units [41,42] respectively were considered for sizing and base-pair estimation. Allele binning was performed for individual UGMS loci for precise and accurate allele size determination and discrimination of homozygous and heterozygous allele types [42,43] for each genotype.

**Table 3:** List of genotypes belonging to five cereal species and *Saccharum *complex used in the study

Sl. No.	Genus and species	Clones/varieties	Parentage/origin	Region of adaptation
1	*Hordeum vulgare*	AK559	Unknown	-

2	*Triticum aestivum*	Kalyansona	PJ"S" × GB 55	-

3	*Oryza sativa*	IR64	IR5857-33-2-1 × IR-2061-465-1-5-5	-

4	*Zea mays*	KA509	India	-

5	*Sorghum bicolor*	Pusa chari6	India	-

6	*Saccharum officinarum*	IJ-76-3-1-9	Indonesia	-

7	*S. barberi*	Mamjasahe	India	-

8	*S. sinense*	Malari	Unknown	-

9	*S. robustum*	IM-76-256	Indonesia	-

10	*S. spontaneum*	1151	India	-

11	*Narenga*	*Narenga*ER	Unknown	-

12	*Sclerostachya*	S1135	Unknown	-

13	*Erianthus*	IK76-81	Indonesia	-

14	*Saccharum *spp.	Co 1158	Co 421 × Co 419	Sub-tropical

15	*Saccharum *spp.	CoJ 64	Co 976 × Co 617	Sub-tropical

16	*Saccharum *spp.	CoS 88230	Co 1148 × Co 775	Sub-tropical

17	*Saccharum *spp.	Bo 91	Bo 55 × Bo 43	Sub-tropical

18	*Saccharum *spp.	CoS 8436	MS 68/47 × Co 1148	Sub-tropical

19	*Saccharum *spp.	Co 1148	P 4383 × Co 301	Sub-tropical

20	*Saccharum *spp.	CoPant 84212	Co 1148 × Co 775	Sub-tropical

21	*Saccharum *spp.	Co 87268	Bo 91 × Co 62399	Sub-tropical

22	*Saccharum *spp.	CoLk 8102	Co 1158 GC	Sub-tropical

23	*Saccharum *spp.	Co 7717	Co 419 × Co 775	Sub-tropical

24	*Saccharum *spp.	Co 87263	Co 312 × Co 6806	Sub-tropical

25	*Saccharum *spp.	Co 89003	Co 7314 × Co 775	Sub-tropical

26	*Saccharum *spp.	CoPant 84211	Co 6806 × Co 6912	Sub-tropical

27	*Saccharum *spp.	Co 8347	Co 419 × CoC 671	Sub-tropical

28	*Saccharum *spp.	Co 7219	Co 449 × Co 658	Tropical

29	*Saccharum *spp.	Co 740	P 3247 × P 4775	Tropical

30	*Saccharum *spp.	Co 86010	Co 740 × Co 7409	Tropical

31	*Saccharum *spp.	Co 86032	Co 62198 × CoC 671	Tropical

32	*Saccharum *spp.	Co 85002	Co 62198 × (-)	Tropical

33	*Saccharum *spp.	Co 8021	Co 740 × Co 6806	Tropical

34	*Saccharum *spp.	Co 8371	Co 740 × Co 6806	Tropical

35	*Saccharum *spp.	Co 6304	Co 419 × Co 453	Tropical

36	*Saccharum *spp.	Co 419	PoJ 2878 × Co 290	Tropical

37	*Saccharum *spp.	Co 62175	Co 951 × Co 419	Tropical

38	*Saccharum *spp.	Co 7704	Co 740 × Co 6806	Tropical

39	*Saccharum *spp.	Co 86249	CoJ 64 × CoA 7601	Tropical

40	*Saccharum *spp.	CoC 671	Q 63 × Co 775	Tropical

41	*Saccharum *spp.	Co 87025	Co 7704 × Co 62198	Tropical

Variation in the fragment size (bp) of amplified alleles at each polymorphic UGMS marker locus was compared with changes in the number of microsatellite repeat-units at that target locus, and the occurrence of "stepwise" and "mixed" type of allele size distribution was inferred. When the allele size differences strictly corresponded to the variation in the number of repeat-units, it was considered as stepwise distribution. A mixed allele distribution was assumed when the allele size differences could partly be explained by the stepwise model. Multiple amplicons obtained by a primer-pair with peaks ≥ 1500 fluorescence units showing ≥ 100 bp allele size differences were binned into different loci. To confirm that the primers amplified the target microsatellite repeat motifs in different species and genera, the amplified products were purified using Micropon PCR purification kits (Millipore, Bedford, MA, USA) and sequenced two times in both forward and reverse directions using a capillary-based Automated DNA Sequencer (MegaBACE 1000, Amersham Biosciences, Piscataway NJ, USA). The trace files were base called, checked for quality and assembled into contigs [44]. The high quality sequences thus obtained were used for interspecies comparison using CLUSTALW multiple sequence alignment tool employing BIOEDIT software [45]. Ten size variant amplicons each of three microsatellite markers (UGSuM2, UGSuM26 and UGSuM27) showing both step-wise and mixed type of allele size distribution in sugarcane species, genera, varieties and cereals were eluted, purified, cloned in pGEM-T Easy Vector (Promega, USA) and sequenced as described above.

### Assessment of functional genetic diversity

The polymorphic information content (PIC) was calculated using the formula, PIC = 1- ∑P_ij_^2 ^[46], where P_ij _is the frequency of the j^th ^allele for i^th ^locus summed across all alleles for the locus. Cluster analysis among the 36 genotypes of *Saccharum *species, varieties and related genera was based on Nei and Li similarity coefficient [47] using the un-weighted pair group method (UPGMA) in PowerMarker Version 3.0 [48,49] software. The confidence limits of UPGMA based dendrogram was determined by 1000 bootstrap replicates and bootstrap of 50% majority rule consensus unrooted phylogenetic tree was constructed.

## List of abbreviations

Sugarcane, Unigenes, Microsatellites, Functional genetic diversity

## Authors' contributions

SKP conducted mining of UGMS, marker design, large-scale genotyping, polymorphism survey, functional diversity estimation and drafted the manuscript. AP was involved in genotyping and sequencing. KG and TRS participated in microsatellite mining and data analysis. PSS and NKS helped in data analyses, interpretation and drafting of the manuscript. TM designed the study, guided data analysis and interpretation, participated in drafting and correcting the manuscript and gave the final approval of the version to be published. All authors have read and approved the final manuscript.

## Supplementary Material

Additional file 1 **Distribution of codon repeats along with the corresponding aminoacids in the unigenes of sugarcane**. Click here for file

Additional file 2 **Frequency and abundance of microsatellite repeat motifs and distribution of class I and class II motifs in the unigenes of sugarcane**. Click here for file

Additional file 3 **Primers designed from sugarcane unigenes carrying perfect microsatellite repeat-motifs along with the expected amplicon size and putative unigene function**. Click here for file

Additional file 4 **Primers designed from sugarcane unigenes carrying compound class I microsatellite repeat-motifs along with the expected amplicon size and putative unigene function**. Click here for file

Additional file 5 **Alignment showing the presence of class I UGMS repeat-motifs in sugarcane species, related genera and five cereal species**. Click here for file

Additional file 6 ***In silico *polymorphism of sugarcane UGMS loci at sequence level in five cereal species**. Click here for file

Additional file 7 **Functional annotation of 672 unigenes carrying microsatellites of sugarcane**. Click here for file

Additional file 8 **Primers targeting the microsatellite repeats present in the functional domains of proteins encoded by the unigenes**. Click here for file

Additional file 9 **Primers targeting the microsatellite repeats located within the functional domains of proteins encoded by sugarcane unigene sequences which gave single locus and step-wise allele amplification**. Click here for file

Additional file 10 **Alignment depicting the functional significance of sugarcane UGMS markers targeting the microsatellite repeat-motif (AG)_n _located within the protein kinase domain of unigene**. Click here for file
