# Supplementary material for: Functionally relevant microsatellites in sugarcane unigenes
Source: BMC Plant Biol. 2010 Nov 17;10:251. doi: 10.1186/1471-2229-10-251 (PMC3017843; doi:10.1186/1471-2229-10-251)
Supplement: Additional file 2 — Frequency and abundance of microsatellite repeat motifs and distribution of class I and class II motifs in the unigenes of sugarcane. [file 1471-2229-10-251-S2.DOC]

Additional file 2: Frequency and abundance of microsatellite repeat motifs and distribution of class I and class II motifs in the unigenes of sugarcane

| Microsatellite motifs | **Frequency (%)** | **Abundant motifs with frequency (%)** | **Frequency (%) of class I UGMS** | **Frequency (%) of class II UGMS** |
| --- | --- | --- | --- | --- |
| Dinucleotides | 200 (23.8) | GA, 42 (21), CT 36 (18), TA 21 (10.5) | 64 (32) | 136 (68) |
| **Trinucleotides** | 615 (73.1) | GCA/GCC/GCG/GCT, 132 (21.5) | 117 (19) | 498 (81) |
| **Tetranucleotides** | 15 (1.8) | CACG, AGGA, AAAG, 2 (13.3) | 15 (100) | 0 |
| **Pentanucleotides** | 7 (0.83) | CGCCG, CCCTG, CGTGT, 1 (14.3) | 7 (100) | 0 |
| **Hexanucleotides** | 4 (0.47) | CTCTCC, CTGCGG, AGGGAG, 1 (25) | 4 (100) | 0 |
| **Total** | **841** | **-** | **207 (24.6)** | **634 (75.4)** |
