# Supplementary material for: Functionally relevant microsatellites in sugarcane unigenes
Source: BMC Plant Biol. 2010 Nov 17;10:251. doi: 10.1186/1471-2229-10-251 (PMC3017843; doi:10.1186/1471-2229-10-251)
Supplement: Additional file 3 — Primers designed from sugarcane unigenes carrying perfect microsatellite repeat-motifs along with the expected amplicon size and putative unigene function. [file 1471-2229-10-251-S3.DOC]

| **Additional file 3: Primers designed targeting the sugarcane unigenes carrying perfect microsatellite repeat-motifs along with primer sequences, expected product size and putative unigene function**   |  |  |  |  |  |  |  |  |  |  | | | --- | --- | --- | --- | --- | --- | --- | --- | --- | --- | --- | | **Unigene**  **IDs'** | **UGMS primers*** | **Microsatellite repeat-motifs** | **Location** | **Forward primer (5'-3')** | **Reverse primers (5'-3')** | **Left TM** | **Right TM** | **Product Size** | **Putative functions** | | | CA244884^A^ | UGSuM1 | (AT)52 | 3'UTRs | AGCAGAGACACACGCACA | GCTCCCACCACCACCAGA | 57 | 63 | 280 | hypothetical protein, *Oryza* | | | CA297715^A, B^ | UGSuM2 | (AT)43 | CDS | TTCAGAAACGATTTGCCTTG | CACGTGCCGCTGAATGTAT | 55 | 54 | 220 | Sucrose phosphate synthase, *Zea mays* | | | CA127223^A^ | UGSuM3 | (TA)36 | CDS | AAGAAGAGCCGTAGAAACAAC | ATTGAGCGAGGGATGAAC | 55 | 56 | 227 | nonspecific lipid transfer protein, *Hordeum vulgare* | | | CA283973^A^ | UGSuM4 | (AT)30 | CDS | AGTGAAAGGAGCCAGAAAG | CATTGTTATGCGACTTGTGTT | 54 | 56 | 315 | hypothetical protein, *Oryza* | | | CA278792^A, B^ | UGSuM5 | (TA)28 | CDS | TCACATCCATCATCCACAGC | TCCAATGCAAGCAAACTCAC | 55 | 55 | 200 | Cyclin III, *Zea maize* | | | CA226611^A^ | UGSuM6 | (AT)28 | CDS | ACTGACACACACGCACAC | TGGAAGTGAATGAAGCGA | 54 | 56 | 279 | transcriptional coactivator-like protein, *Arabidopsis* | | | CA104636^A^ | UGSuM7 | (CATTA)11 | 5'UTRs | TATACGTAGTAGTGATGATGACCG | CTCCTTCGTCCAGTACCAGTAG | 56 | 58 | 145 | auxin-induced protein, *Arabidopsis* | | | BU103122^A^ | UGSuM8 | (AG)24 | 5'UTRs | GTCCTAGCAGGTCTAGGTCTTG | AAATCTGAGCTAGATCCTCTCC | 55 | 55 | 112 | hypothetical protein, *Oryza* | | | CA134611^A^ | UGSuM9 | (CT)24 | 3'UTRs | GCTCCTGCTTTATCTACACAC | AATCCAGCCCTACTACTGAAC | 54 | 55 | 254 | hypothetical protein, *Oryza* | | | CA206100^A^ | UGSuM10 | (AG)22 | CDS | ACCCTTCCCATTCCCATC | CTCCAGGTTCGCCACCAC | 59 | 62 | 287 | cytochrome b561, *Zea mays* | | | AY596609^A, B^ | UGSuM11 | (TA)21 | 3'UTRs | TGGTAACCCTAGGCAGGTGA | GTGCACCAGATTTGGATGGT | 56 | 55 | 170 | fructose-bisphosphate aldolase, *Zea mays* | | | CA130288^A^ | UGSuM12 | (GA)20 | CDS | TTGACACAGATAAACACCACA | ATCACTTGCCCTCCCTTC | 54 | 57 | 307 | tubulin folding cofactor B, *Arabidopsis* | | | CA272491^A^ | UGSuM13 | (CCA)13 | CDS | GTTCTTAGTCCAGCCGTAGTT | ATCGTTGTTGTCGGTGTC | 55 | 55 | 352 | hypothetical protein, *Vitis vinifera* | | | CA138656^A^ | UGSuM14 | (AC)19 | CDS | AGGTTGGCTTGGTGTCTT | TCACTCACAATCACTGGCTAC | 55 | 56 | 369 | NHL1 (NDR1/HIN1-like 1), *Arabidopsis* | | | CA279221^A, B^ | UGSuM15 | (CCTCGC)6 | CDS | GTTTAAGACAAGATGGTGTAGATG | TACATATTTACATTGTTACTCCGC | 54 | 55 | 200 | hypothetical protein, *Oryza* | | | CA278282^A, B^ | UGSuM16 | (AT)18 | CDS | GCGTCTTCATCATCTGCAAC | TAGAGAGACATGGGGTGCAT | 56 | 55 | 282 | pathogenesis-related protein PRMS, *Oryza* | | | CA253277^A, B^ | UGSuM17 | (AG)18 | CDS | TTTCCATTCTTCCATTCAACTG | GGCAGGCTGAGAGACTGTTC | 55 | 55 | 300 | abcisic acid-inducible protein kinase, *Arabidopsis* | | | CA227482^A, B^ | UGSuM18 | (GA)18 | CDS | GGCGAGAGAGAGAGAGAGAGAG | AGGTGGAGATCTTGAGGTAGGC | 55 | 54 | 200 | glycine decarboxylase, *Arabidopsis* | | | CA106507^A^ | UGSuM19 | (AGGGAG)6 | 3'UTRs | GTTGTCGAGATGATACAGAAGTAA | GTACAATATTACACACACAAAGGG | 55 | 55 | 248 | hypothetical protein, *Phaseolus* | | | CA126180^A, B^ | UGSuM20 | (TCA)12 | CDS | ATCCCTTATGCTACAGAAATGT | TTAGCCTAGAGGTTTGATTGAT | 54 | 54 | 159 | acetyl-CoA synthetase, *Arabidopsis* | | | CA177414^A, B^ | UGSuM21 | (AGGA)9 | CDS | CGCTCCCTCACCGTCATT | CTCCGCATCCTCGTCACC | 62 | 62 | 219 | AtTFIIB2, *Arabidopsis* | | | CA223153^A, B^ | UGSuM22 | (GCG)12 | 3'UTRs | CTCCCTCCTCCTCCCGTTG | CTCTTGGGTGTGAACCAG | 64 | 54 | 227 | Polyadenylate-binding protein, *Arabidopsis* | | | CA227482^A^ | UGSuM23 | (GA)18 | CDS | CAGCAACAAGAGGCACCA | ATTTGGAGGAGAACACGAA | 59 | 55 | 134 | unknown protein, *Oryza* | | | CA122659^A, B^ | UGSuM24 | (TTTTC)7 | 5'UTRs | CTGTACAACAGCAATTATGAATCT | CTCGACTACGAGAGGATATGAT | 55 | 55 | 276 | hypothetical protein, *Oryza* | | | CA122659^A, B^ | UGSuM25 | (TTTGA)7 | 5'UTRs | AGAGAGCGAGCGAGAGAA | CGTCGTGTCGTAGAATAACA | 56 | 54 | 164 | hypothetical protein, *Oryza* | | | BU103692^A, B^ | UGSuM26 | (CT)17 | 5'UTRs | CTCGATCCCAGAGAGCTCCACAG | AGTACCGAATTCATTAAACTCCT | 55 | 63 | 199 | beta-amylase, *Glycine max* | | | CA073284^A, B^ | UGSuM27 | (GGC)11 | CDS | CTGCAGTACGGTCCGGAATC | GTACCACCATGGCTCTAGCTTC | 55 | 57 | 180 | 30S ribosomal protein S16, *Pisum* | | | CA180693^A^ | UGSuM28 | (CTG)11 | CDS | CTGAAACAGCAAACCTGAA | AGAGCCATTGAAAGAGATTG | 54 | 54 | 390 | Zinc finger, C2H2-type, *Medicago* | | | CA248458^A^ | UGSuM29 | (AAAG)8 | CDS | ATTACTTGCCTGTCTTGAATG | GATGTTTGGACCTTGAACC | 54 | 55 | 394 | protein kinase family protein, *Oryza* | | | CA133924^A, B^ | UGSuM30 | (CTCTCC)5 | CDS | CCGAGTGTCCTCATCGCAGAAC | CTCTAGTCTCTTCATAACCTGCTC | 66 | 55 | 344 | auxin-independent growth promoter, *Arabidopsis* | | | CA181871^A^ | UGSuM31 | (AGGAC)6 | 3'UTRs | ATTCCTCAAATCTTATTCAATTCT | GAGATAGTTAACCTTGTGGTGTTT | 54 | 55 | 231 | hypothetical protein, *Oryza* | | | CA228772^A, B^ | UGSuM32 | (GCC)10 | 5'UTRs | CGTTCCCCTCGCGTAGTC | CCAACCCCTTCAAGCTAGTG | 55 | 55 | 213 | SPP30 homolog, *Oryza* | | | AY596606^A, B^ | UGSuM33 | (AGC)10 | CDS | CGAGGCACTGAACCCATATC | TGTTTGAACTGGATGGCGTA | 55 | 55 | 210 | hypothetical protein, *Oryza* | | | CA268640^A, B^ | UGSuM34 | (AAG)10 | CDS | TTACAAATGTAGCCTTGCCTTG | ATCTTTCCTTGCTTGCCTCTC | 63 | 55 | 150 | soluble acid invertase, *Sorghum* | | | AY596606^A^ | UGSuM35 | (AGC)10 | CDS | GTGAGATTCCGATTCCGCTTCC | ACATGAGTATTGTACTGGTGAGAA | 66 | 55 | 389 | MATH, *Medicago* | | | CA064945^A^ | UGSuM36 | (CAG)10 | CDS | CTACAAACACATGAGACGAGAC | TTACATTGTTATTAGTAGCCGTTT | 55 | 55 | 246 | hypothetical protein, *Oryza* | | | CA085095^A^ | UGSuM37 | (CTGCGG)5 | CDS | GAAGAGATTCGTCGCCTCAGTC | AAGGGTAGAGACAGGTAGATCAG | 62 | 55 | 276 | hypothetical protein, *Oryza* | | | CA101287^A^ | UGSuM38 | (GCC)10 | CDS | GTAGTCGCGTGCGCTCTGTCTG | CAGTAATGTTGTCAACTTGAGTCT | 67 | 56 | 347 | o-methyltransferase, *Oryza* | | | CA103857^A^ | UGSuM39 | (TG)15 | CDS | TAAATATGGTGGAGCAAAGTATTA | ACTAGAGCTCTTAAATTCCACAGT | 65 | 54 | 385 | microsomal glutathione S-transferase 3, *Arabidopsis* | | | CA121075^A^ | UGSuM40 | (CT)15 | 3'UTRs | CAATCCCACATCCACATC | ACATACACGAACTGTCCAACT | 55 | 54 | 297 | unknown protein, *Arabidopsis* | | | CA131350^A, B^ | UGSuM41 | (CCG)10 | CDS | ATCATTCTCCATCATTTCTCA | AGGCTCTTCAACCGTGCT | 54 | 58 | 400 | unknown protein, *Arabidopsis* | | | CA133924^A, B^ | UGSuM42 | (CTCTCC)5 | 5'UTRs | TTCATACAGAAGAACCTCCAC | TCCATCAGAGACAAGCAGA | 54 | 55 | 254 | auxin-independent growth promoter, *Arabidopsis* | | | CA136599^A, B^ | UGSuM43 | (CCG)10 | 3'UTRs | CAAAGTGCTGTAGGGCTG | TTCAATGGGTGATAAGTGTGT | 55 | 55 | 352 | Ribose-phosphate pyrophosphokinase 1, *Arabidopsis* | | | CA139800^A, B^ | UGSuM44 | (CT)15 | 5'UTRs | TCCATCAAGCCGTTCCTC | GCCAAGCAGATAAAGAAGTG | 59 | 55 | 400 | rudimentary enhancer, *Glycine max* | | | CA171090^A, B^ | UGSuM45 | (AAAAG)6 | CDS | ATCTCCTCTTATTCGTTCTGG | AGCAGCGTCTTATCTGGG | 55 | 56 | 233 | PAP fibrillin, *Medicago* | | | CA196477^A, B^ | UGSuM46 | (GAC)10 | CDS | ACTCCTCCCGCCTCCACTAC | CTCACCGAAGCAATCAAG | 62 | 55 | 400 | hypothetical protein, *Oryza* | | | CA228772^A, B^ | UGSuM47 | (GCC)10 | CDS | ATTTATGGAGGAAGAAACGG | ATTACAAACAAGAAGAGCGG | 55 | 54 | 223 | transport protein particle (TRAPP), *Arabidopsis* | | | CA272418^A^ | UGSuM48 | (TTGCA)6 | CDS | CCACATACTCTGCGACCA | CTCCTACCACTCCCGCCT | 56 | 60 | 311 | esterase, *Oryza* | | | CA153323^A^ | UGSuM49 | (GGAA)7 | CDS | AAGGTGTACCTGGACCGGCAGAAC | AATTGTCAGGTCAAGTCAGTATAA | 68 | 54 | 277 | hypothetical protein, *Oryza* | | | CA161416^A, B^ | UGSuM50 | (TC)14 | CDS | CTACTGCCGAGGAAAGATCG | GGAAAAGTTTGTGGCAAGGA | 61 | 55 | 200 | hypothetical protein, *Oryza* | | | AY596599^A^ | UGSuM51 | (CAC)9 | CDS | TACTATAATGATAGATCTCCTCCG | GTAATAGGACTGGATTGGAATG | 53 | 55 | 303 | ubiquitin, *Arabidopsis* | | | CA113942^A^ | UGSuM52 | (GGC)9 | CDS | CTTCTTCTTCTTCTTCTTCTTCTG | AAGAATCGAAGGTTAACAACAG | 55 | 55 | 224 | 40S ribosomal protein S19, *Oryza* | | | AY596599^A^ | UGSuM53 | (CAC)9 | 5'UTRs | TACTCCATGGACTTCTCCAA | CAGGAGTTATGTGCTGTGATAC | 55 | 55 | 317 | ubiquitin-associated (UBA)/TS-N domain, *Oryza* | | | CA074283^A^ | UGSuM54 | (GGA)9 | CDS | GAAGGAGAGTTAGGAGAGTCAGT | GACCTCTTCTTGTCTAATACCCT | 55 | 54 | 173 | aminotransferase family protein, *Arabidopsis* | | | CA124417^A^ | UGSuM55 | (GGC)9 | 5'UTRs | CAGACTTGATGTAGGGAACTG | AAGGGAGATGCTGAATAACAC | 54 | 55 | 384 | pr1-like protein, *Oryza* | | | CA200013^A^ | UGSuM56 | (CGG)9 | CDS | TAATACTTTCACCAGCCAA | GGAGCAGCAACGCACAGG | 51 | 64 | 239 | Pyrophosphohydrolase MazG RS21-C6, *Medicago* | | | CA205971^A^ | UGSuM57 | (GTT)9 | CDS | CTACAGACGACGACAGGTATG | TAGGAAGGAACACAGGGCAG | 55 | 60 | 174 | acyl-CoA binding family protein, *Arabidopsis* | | | CA259151^A^ | UGSuM58 | (AGA)9 | CDS | AGAGCCATCAGTCTCCAC | TGAGTCCAAATCTATCAAGCA | 53 | 55 | 370 | expressed protein, *Oryza* | | | CA261837^A^ | UGSuM59 | (TTG)9 | CDS | AGGAGGACTACGAGGAGAAG | AACAAGAACAATCACAAGGAA | 55 | 54 | 334 | zinc finger (C3HC4-type RING finger) family, *Oryza* | | | CA266782^A^ | UGSuM60 | (GCG)9 | CDS | CCCTGAAACAATCTCAAACT | CTTCCAATGAACCAACCTAC | 54 | 54 | 181 | unknown protein, *Arabidopsis* | | | CA276055^A^ | UGSuM61 | (CAC)9 | CDS | ACATCCTTCCCACGCCAG | CCTACTCCTCCTCCTCCTC | 62 | 55 | 100 | Chitin synthase 2, *Arabidopsis* | | | CA279855^A^ | UGSuM62 | (CAA)9 | CDS | ATCTGCCATCAGGCTAAA | TGAGGAATCAGTCTTGTGTAA | 54 | 53 | 176 | oxygenase, *Oryza* | | | CA131357^A^ | UGSuM63 | (GA)13 | CDS | CCGTCCTCTCTTATATATGCAC | GAGCTGCCGTAGTAGTTCTG | 53 | 63 | 400 | Alpha-soluble NSF attachment protein, *Oryza* | | | CA236807^A^ | UGSuM64 | (CT)13 | CDS | TCACCTATCCCACTCTCTACC | CAGATTATTTCCAAGCGTTC | 55 | 55 | 272 | colony-stimulating factor (CSF-1) precursor, *Oryza* | | | CA201095^A^ | UGSuM65 | (AAGGA)5 | 5'UTRs | AATATACTTCTCGATTAATCACCG | CTACTACTACTACCAAGTACGGCG | 55 | 56 | 365 | unknown protein, *Oryza* | | | CA228015^A^ | UGSuM66 | (CTGGG)5 | 5'UTRs | ATACTTCTCGATTAATCACCGAT | GCTCGGTCATCATCACTACTAC | 56 | 56 | 400 | Andropogoneaesin, *Zea mays* | | | CA122274^A^ | UGSuM67 | (CGCCG)5 | 5'UTRs | TCAATCCTCTCACACTTCTCA | TAACCTAACTTGGAACACAGA | 55 | 52 | 182 | AtTLP1 (TUBBY LIKE PROTEIN 1), *Arabidopsis* | | | CA287720^A^ | UGSuM68 | (CGTGT)5 | 5'UTRs | CTCGTCTCGTCTCGTCTC | TCAGTTGCCCTTGTGTCT | 54 | 54 | 279 | lipid transfer protein, *Triticum* | | | CA206415^A^ | UGSuM69 | (TTAT)6 | 3'UTRs | GCTAGCAACAGATCGGAGTGTC | GTGTACCGTGTGTATGTCTGTC | 61 | 55 | 305 | expressed protein, *Arabidopsis* | | | CA196900^A^ | UGSuM70 | (GAG)8 | CDS | TAATATACATCACGACACAACAAA | TTGTGTTATCTAGACGATACCTTT | 55 | 54 | 348 | Avr9 elicitor response protein, *Arabidopsis* | | | CA270879^A^ | UGSuM71 | (CTG)8 | CDS | ATCTCCTCTCCCTCTCTCAAAC | ACTAGACACGGACAGAGAGAGAC | 58 | 56 | 297 | expressed protein, Oryza | | | CA248131^A^ | UGSuM72 | (ACC)8 | CDS | ATCGATCATTTCCTCTTCTTAGTA | AATTTGATTCATAGGATTGTGAG | 55 | 55 | 383 | phosphatase 2C, *Arabidopsis* | | | CA228375^A, B^ | UGSuM73 | (CGC)8 | 5'UTRs | CTTTCAACCTCTACACCTCCAC | ACTAGAAGACTGAGAAGAACCAGT | 57 | 54 | 280 | 40S ribosomal protein S11, *Arabidopsis* | | | CA261182^A, B^ | UGSuM74 | (ACA)8 | CDS | TCAGCAGCTGTGAAGTTTCATT | CGTCTCTTTTGGGTTTCATCTC | 54 | 55 | 390 | trAndropogoneaescription regulator protein, *Oryza* | | | CA219230^A, B^ | UGSuM75 | (TA)12 | CDS | TTGTGCTGATGTTTCCTGCT | CAAGAGAAGATGCCATTAGCC | 55 | 55 | 295 | patatin-like protein, *Oryza* | | | CA071279^A^ | UGSuM76 | (ACG)8 | CDS | GAGTAGCAATGATTAAACGTAATG | TCGAGATATATGTCCTGGTATCTA | 56 | 54 | 284 | hypothetical protein, *Oryza* | | | CA077626^A^ | UGSuM77 | (GCA)8 | CDS | CTGAAGAGATCGTCCAGCATGAG | ATCTATACACACATCGCCTAATC | 63 | 55 | 384 | Glutenin, HMW, *Triticum* | | | CA084157^A^ | UGSuM78 | (CAG)8 | CDS | ACATATACATGTCCTCAGTCCTC | TACTTCTATGGTACAGGTACAGCA | 54 | 55 | 175 | hypothetical protein, *Oryza* | | | CA120241^A^ | UGSuM79 | (AGG)8 | CDS | AACTTGTGGTTCCTAGCTGTTT | AATCCTTACCTTAGAAGATCACC | 55 | 54 | 365 | nucleic acid binding protein, *Arabidopsis* | | | CA130602^A^ | UGSuM80 | (TCC)8 | CDS | TATCAAGAAACAGACAACCCA | AGACTCCAAAGATGGTGAACT | 55 | 55 | 318 | serine carboxypeptidase S28 family protein, *Oryza* | | | CA130848^A^ | UGSuM81 | (AGCT)6 | CDS | GCAATCTTATTCAGGTAGGAGA | CTTTGATATCTTGAAGAGCACC | 60 | 55 | 295 | hypothetical protein, *Oryza* | | | CA139535^A^ | UGSuM82 | (GCA)8 | 5'UTRs | GCACCGCTTCATCATCTAC | GAGGCTTAATGGTTCAAG | 56 | 53 | 400 | serine carboxypeptidase, *Arabidopsis* | | | CA157740^A^ | UGSuM83 | (GA)12 | CDS | GTTCACCACCTCCGACAC | TAGCAGCAACCTCACACTC | 57 | 54 | 351 | CBS domain-containing protein, *Arabidopsis* | | | CA159967^A^ | UGSuM84 | (CAA)8 | 5'UTRs | CTCCGAGAACGTCTGCGTGT | TGTTCTCAAACCTGGTGTAAC | 56 | 54 | 235 | expressed protein, *Oryza* | | | CA162524^A^ | UGSuM85 | (CGC)8 | 5'UTRs | AGAGAGGGAGAAGAACAAGAC | ACCAGAAGGACAGAGATGG | 54 | 54 | 319 | TaCBF5, *Triticum* | | | CA185016^A^ | UGSuM86 | (CGC)8 | CDS | ATGACAGCAGCACAATGA | CACCCAGTTGAATAAGTGA | 54 | 51 | 285 | mitochondrial ribosomal protein L11, *Triticum* | | | CA186729^A^ | UGSuM87 | (CTC)8 | CDS | GAAGCGAATGTGAACTGG | GAGAGCAGCGAGGACAGG | 55 | 59 | 207 | hypothetical protein, *Oryza* | | | CA195435^A^ | UGSuM88 | (GA)12 | CDS | CACTTCCCAGAGACCCAG | GACCTTAGCAATCAAGACAGA | 56 | 54 | 287 | zinc-finger protein [Oryza sativa | | | CA196900^A^ | UGSuM89 | (GAG)8 | CDS | CTCCCAAAGCAAACCCTT | GTTCTTGACCTTCTTCCTGTC | 57 | 55 | 352 | galactosyltransferase family protein, *Arabidopsis* | | | CA222407^A^ | UGSuM90 | (GCC)8 | 3'UTRs | CCGACCCACTCGCCCAAA | AACTTCTTCTTCTTGCTGA | 67 | 50 | 115 | seven in absentia (SINA) family protein, *Arabidopsis* | | | CA228375^A^ | UGSuM91 | (CGC)8 | CDS | GTGTGGAACTGTGGATGG | ACCGAAACAACAAGTAAACAA | 55 | 54 | 348 | 40S ribosomal protein S11, *Arabidopsis* | | | CA251651^A^ | UGSuM92 | (CGC)8 | CDS | TACTATGGAGGCGGGAGG | TAGAAGAGCACAGAGCAAAC | 58 | 53 | 297 | glutathione peroxidase, *Zea mays* | | | CA261154^A^ | UGSuM93 | (TGT)8 | CDS | CAAGTCGTCCTCTTCGTC | ATTAGACAGGTAAATCGTGCC | 54 | 55 | 217 | reverse transcriptase family member, *Glycine max* | | | CA261182^A^ | UGSuM94 | (ACA)8 | CDS | GAGTCAGGGAGAGGAACAG | TAGATACCGAGCACACCAG | 54 | 54 | 238 | transcriptional regulator, *Zea mays* | | | CA267736^A^ | UGSuM95 | (CGC)8 | CDS | AGTGAGTGAAGAAGAGCCAG | AAGAACAACCGAAGGAGATT | 54 | 55 | 366 | hypothetical protein, *Arabidopsis* | | | CA093071^A, B^ | UGSuM96 | (AT)11 | CDS | TCAAACCAGGATCTAAGCTCAC | GGTAGTGCCATTGAGGTTGC | 55 | 54 | 400 | apyrase, *Oryza* | | | CA229840^A, B^ | UGSuM97 | (GA)11 | 5'UTRs | GCGAGAGAGATAGAGGGAGAGA | AGGTGCCGTTCATGAGGTAGT | 57 | 54 | 250 | glycine decarboxylase, *Arabidopsis* | | | CA093071^A^ | UGSuM98 | (AT)11 | CDS | AATATACTTCTCGATTAATCACCG | CTACTACTACTACCAAGTACGGCG | 56 | 56 | 400 | ATAPY2 (APYRASE 2), *Arabidopsis* | | | CA101958^A^ | UGSuM99 | (CT)11 | CDS | ACTAACTCTCTTCAACTTCCTCTG | AGCTGTTCCTCTTTAGCTAGTTC | 60 | 54 | 294 | Lipase, *Oryza* | | | CA117570^A^ | UGSuM100 | (GC)11 | CDS | AGACAGAAAGAAACGCGTGGAG | CATTGTATAGAGTAATCTGCTCGT | 56 | 54 | 137 | hypothetical protein, *Oryza* | | | CA130809^A^ | UGSuM101 | (CCG)7 | CDS | GTATTTGCGTAAATTACAGGAGT | CTCCTGGATATGACACCTTCTT | 54 | 57 | 192 | hypothetical protein, *Oryza* | | | CA209245^A^ | UGSuM102 | (GGC)7 | CDS | TGGTTATGTGTGTAACGGTAATA | TAATTATGCTGAGAATTACAAGGA | 54 | 55 | 142 | hypothetical protein, *Oryza* | | | CA071318^A^ | UGSuM103 | (GAG)7 | CDS | CTCAACAACTTGCTCGGCTATT | CACAGAAACTAACCTTCTTCTTG | 60 | 55 | 273 | hypothetical protein, *Oryza* | | | CA222779^A^ | UGSuM104 | (CTT)7 | CDS | CACCGCAGCCTGACACAGAACC | AGGAACTCAGCATACTCGTGAC | 69 | 58 | 333 | leaf senescence related protein, *Arabidopsis* | | | CA151601^A^ | UGSuM105 | (GCC)7 | CDS | CTTTCTCGTGAGCAGAGCAGGT | ATTTACAGAATCACACGGTAGTAA | 63 | 54 | 379 | hypothetical protein, *Oryza* | | | CA137197^A^ | UGSuM106 | (GAC)7 | CDS | TAAATGATGAAATCTACGACGTTA | ATGAGAAGCTAGTAAGGTAGCACT | 55 | 54 | 126 | hypothetical protein, *Oryza* | | | CA280782^A^ | UGSuM107 | (TGC)7 | CDS | AATCGGCGCTGACCATGGACTC | AGAACACAACTTTCACCTTGTT | 54 | 55 | 276 | S-adenosylmethionine synthetase 1, *Arabidopsis* | | | CA071393^A^ | UGSuM108 | (ACG)7 | CDS | TCTACACATTGCTTTATGAACTTT | CCCAACACCGGCACTTTATGGG | 57 | 55 | 276 | hypothetical protein, *Oryza* | | | CA125878^A^ | UGSuM109 | (CGG)7 | CDS | CTTAAGATTATTGGGAAGTCAAAT | GCCCACATTAGTAAGATTAAAGAT | 55 | 55 | 304 | hypothetical protein, *Oryza* | | | CA065436^A^ | UGSuM110 | (CGC)7 | CDS | GTAGCACTAACAATAGCAGTTCAT | TTTGTGTAGATGTTTAATTCGTTT | 54 | 55 | 365 | hypothetical protein, *Oryza* | | | CA276184^A^ | UGSuM111 | (GTC)7 | CDS | AGAAGGTGATCCTCAAGGACAAG | AACTGATCCCTCTTTCATATATTC | 61 | 54 | 387 | legumin-like protein, *Pisum* | | | CA300125^A^ | UGSuM112 | (CAC)7 | CDS | AAACGATCAGATCTAGCACAAT | CTGCTGTCTTAGGTACAGTCTTC | 55 | 55 | 299 | HAK2 (K+ Andropogoneaesporter), *Arabidopsis* | | | CA179488^A^ | UGSuM113 | (GGC)7 | CDS | TATCTGATCGGTAGCAAATAGC | GTGGTTAAGAAGAGACTAAGTTCG | 56 | 55 | 370 | mitochondrial inner membrane protein, *Arabidopsis* | | | CA164558^A^ | UGSuM114 | (CGC)7 | CDS | ACTTAGTCTCTTCTTAACCACTGC | AAGTAATTATCTATAGTGCCACCC | 55 | 55 | 210 | GR1b gene, *Zea mays* | | | CA159463^A^ | UGSuM115 | (TGC)7 | CDS | ATCTATCGGTCTTCTGGAGATT | CACTTCCTCCTTATTATACCACTT | 56 | 55 | 283 | hypothetical protein, *Oryza* | | | CA161324^A^ | UGSuM116 | (GCG)7 | CDS | ATCTTCAGATTGGTCTAATCCTAC | GAGAGGTAATAGGTTATCACAAGG | 54 | 55 | 265 | hypothetical protein, *Oryza* | | | CA185255^A^ | UGSuM117 | (CAC)7 | CDS | TAATATACATCACGACACAACAAA | TTGTGTTATCTAGACGATACCTTT | 55 | 54 | 348 | hypothetical protein, *Oryza* | | | CA195892^A^ | UGSuM118 | (CGG)7 | CDS | GTACTGAGAGCCAAAGAAACAAC | CTCTTCTTGAGCTGTCTCCAAGC | 57 | 62 | 217 | phosphatidylserine synthase, *Arabidopsis* | | | CA171184^A^ | UGSuM119 | (ACA)7 | CDS | ACATGCCAAGAAGCAGAAGAAG | ACGTAGAAGTAGACCTTCCAGTT | 60 | 55 | 329 | hypothetical protein, *Oryza* | | | CA282680^A^ | UGSuM120 | (ATT)7 | CDS | GTTTCCTTCCTTCTCGTGCCAT | CCTAATAAGTATCGTTGAAGGTG | 63 | 55 | 331 | thaumatin- protein precursor, *Triticum* | | | CA189772^A^ | UGSuM121 | (AGA)7 | CDS | GAGAAGACTGAGAGTTTGACTGTA | CTTCTTCATCCTCTCTAACTTGAT | 54 | 55 | 157 | hypothetical protein, *Oryza* | | | CA204927^A^ | UGSuM122 | (GCC)7 | CDS | ATAGAGATAGAGGGAGTATGCTTG | AGTCTCATTCTCATCAGAAACC | 55 | 55 | 317 | nitrilase 1 like protein, *Arabidopsis* | | | CA086833^A^ | UGSuM123 | (GCT)7 | CDS | TTCAAGGTCAAATATGGTGATA | AGCCTTCAATAGCTGACATTAT | 55 | 55 | 317 | unknown protein, Oryza | | | CA236932^A^ | UGSuM124 | (GCC)7 | CDS | GTGAGATTCCGATTCCGCTTCC | ACATGAGTATTGTACTGGTGAGAA | 66 | 55 | 389 | 60S ribosomal protein L30, *Oryza* | | | CA106018^A^ | UGSuM125 | (GCG)7 | CDS | ACGAGTTCAGGGCGCTGATAGAG | ATCACGACGTCATAGTCCGTAAC | 66 | 60 | 166 | homeodomain leucine zipper, *Arabidopsis* | | | CA157937^A^ | UGSuM126 | (GCA)7 | CDS | GCCGAAGCCTCTCCTCTCCTCC | GTCATCAATGACAGAGATGTAGAC | 69 | 55 | 116 | expressed protein, *Arabidopsis* | | | CA103270^A^ | UGSuM127 | (CGG)7 | CDS | AATACAGCGCAATATTTCCTCC | ATAACCTCAACGCCAAGAGCAAGT | 59 | 64 | 341 | phragmoplastin, *Arabidopsis* | | | CA272322^A^ | UGSuM128 | (ACC)7 | CDS | ATCGATCATTTCCTCTTCTTAGTA | AATTTGATTCATAGGATTGTGAG | 55 | 55 | 383 | silencing group B protein, *Oryza* | | | CA286116^A^ | UGSuM129 | (GGC)7 | CDS | CTGTGCTCCTGCCCTGCCTTTC | AATAATTCTGCCTCTGATAGAAAC | 66 | 56 | 400 | nuclear Andropogoneaesport factor 2 (NTF-2), *Oryza* | | | CA110734^A^ | UGSuM130 | (CGA)7 | CDS | GCGTCTCTGCTCTGCACTCTGC | ATTAACATATTCATAGCCCAATTT | 67 | 55 | 391 | RPB5d protein, *Oryza* | | | CA098361^A^ | UGSuM131 | (GCG)7 | CDS | TAGCAATCTACTCCCTACGTCTAC | GTTGACGTTGATCAGCCCGTTG | 56 | 67 | 378 | hypothetical protein, *Oryza* | | | AY596586^A^ | UGSuM132 | (CGT)7 | CDS | AATCGGCGCTGACCATGGACTC | AGAACACAACTTTCACCTTGTT | 69 | 55 | 354 | andropogoneaekyrin-like protein, *Zea mays* | | | AY596527^A^ | UGSuM133 | (GGC)7 | CDS | GTTTACATCCACCTCCGCCACC | GTACTCCACATTGATCTGTTCGT | 61 | 55 | 320 | expressed protein | | | CA234584^A^ | UGSuM134 | (CGG)7 | CDS | GTGAATCTGCAGGCTGCTGGAAG | GCACTAGTCACTACTACACACGC | 55 | 69 | 232 | hypothetical protein, *Oryza* | | | CA149322^A^ | UGSuM135 | (CGC)7 | CDS | GTCCTAGCAGGTCTAGGTCTTG | AAATCTGAGCTAGATCCTCTCC | 55 | 55 | 112 | ubiquitin/ribosomal protein-maize | | | CA215720^A^ | UGSuM136 | (GCC)7 | CDS | AAGAACATCATGTCGGTGCAGT | GTAAGCTCTTGGTAACATTCTTG | 53 | 56 | 273 | 40S ribosomal protein S7 | | | CA177425^A^ | UGSuM137 | (GCA)7 | CDS | GTTCTTAGTCCAGCCGTAGTTGT | TCAAATTTCTCAGAACCTTCAC | 54 | 56 | 363 | L-ascorbate peroxidase | | | AY093815^A^ | UGSuM138 | (CAC)7 | 5'UTRs | ATCTTCAGATTGGTCTAATCCTAC | GAGAGGTAATAGGTTATCACAAGG | 54 | 55 | 265 | trypsin inhibitor, *Allium* | | | CA067451^A^ | UGSuM139 | (ACG)7 | CDS | CTAGTGGTAGCCTGAGGTACGGT | CCTCTCTAGTTCTTTCTTCTCTTG | 55 | 54 | 372 | subtilisin-like serine protease, *Arabidopsis* | | | CA071318^A^ | UGSuM140 | (GAG)7 | CDS | TTTGTTGACTGATCCCATCTCG | TTTCAACTTGTCAATATTGTTCTT | 54 | 55 | 389 | RALFL23 , *Arabidopsis* | | | CA082679^A^ | UGSuM141 | (GGC)7 | CDS | AAAGACTCCAAGCTCCTGTGTG | CAAGTTTATTAGGGTCTGCAAG | 60 | 55 | 367 | GRF-interacting factor 1, *Zea mays* | | | CA090548^A^ | UGSuM142 | (GCC)7 | CDS | CTCTGTTGTCATAAGAAGAGACAC | CTTTGATTCAGCAGCATAAACT | 54 | 60 | 399 | outer envelope membrane protein, *Arabidopsis* | | | CA094010^A^ | UGSuM143 | (CGC)7 | 5'UTRs | AGTGCTACCTTACTAGCTTCTCAT | ATTTAATTATCCGAACAAGGTAGT | 64 | 54 | 238 | antifreeze glycoprotein, *Arabidopsis* | | | CA094962^A^ | UGSuM144 | (GCC)7 | 5'UTRs | CTCTCCCGTTCCCATCTCACTC | TAACAGTTCTTGTAGTTAACGGAG | 64 | 54 | 238 | aspartic proteinase 2, *Glycine max* | | | CA094967^A^ | UGSuM145 | (CAG)7 | CDS | CCATCTTCATGACAACCTCT | TATCGATCCATCCCTATACATC | 57 | 63 | 114 | calcium-binding EF hand family protein, *Arabidopsis* | | | CA099491^A^ | UGSuM146 | (GCA)7 | CDS | CTGCTGTTCCTCTTATTGCTCC | GTTAGTAGCAGCACTCATCTAGC | 66 | 58 | 400 | NAC domain transcription factor, *Triticum* | | | CA106494^A^ | UGSuM147 | (GCT)7 | 3'UTRs | ACTTCGCCTACACGCTCGAT | CATTTGACTCCTTCAGTTCAG | 55 | 54 | 368 | conserved hypothetical Fe-S oxidoreductase, *Oryza* | | | CA110650^A^ | UGSuM148 | (CCG)7 | 5'UTRs | AACGTATCTTTATTTCCATTCTTC | CTTTCAGTTCAACTTTGGATAAAT | 55 | 54 | 375 | acetylornithine deacetylase, *Brassica* | | | CA112979^A, B^ | UGSuM149 | (AGC)7 | CDS | GTTCAATCAAATCCCTCTCCTC | AGCTTGGTCAGCTCCTCATCGTT | 65 | 55 | 394 | ubiquitin-specific protease 4 (UBP4), *Arabidopsis* | | | CA116368^A, B^ | UGSuM150 | (GGC)7 | CDS | ACACTGACCGATGGATCCTCTT | ATCAACGTGGACCAGATCTTCTT | 65 | 55 | 243 | hAT family dimerisation domain protein, *Arabidopsis* | | | CA129032^A^ | UGSuM151 | (GAG)7 | CDS | TATCGGCAATCATCAAGAG | ATGGTAATGCTCAACTCCAC | 54 | 55 | 182 | hypothetical protein, *Oryza* | | | CA130809^A^ | UGSuM152 | (CCG)7 | CDS | TTATGCTGATGTCTCAAGTCC | CTATTTCAAGCCTCTCTGTTG | 55 | 54 | 348 | hypothetical protein, *Oryza* | | | CA133533^A^ | UGSuM153 | (CAG)7 | 5'UTRs | CAGGCAACCAAACCTTCT | GATAACACAACCAACACCC | 56 | 53 | 252 | hypothetical protein, *Oryza* | | | CA135267^A^ | UGSuM154 | (GCC)7 | 3'UTRs | GTCTGTCATCTTGCTCTGAAA | AGGCTTTGTATTCCCACC | 55 | 54 | 325 | expressed protein, *Oryza* | | | CA137197^A^ | UGSuM155 | (GAC)7 | 3'UTRs | CTCGCTGCTCGGAATCAC | AAATGAAGGCAAGAATACAAG | 60 | 53 | 385 | hypothetical protein, *Oryza* | | | CA139081^A^ | UGSuM156 | (GCT)7 | CDS | TAGAGGAAATAGCAGAACAGG | AGACTGACACCTTTGAGATGA | 54 | 54 | 264 | Anter-specific proline-rich protein, *Arabidopsis* | | | CA155461^A^ | UGSuM157 | (GAG)7 | CDS | GCTTACATACAATCCCAATCA | TCAGAGAAAGATAACGCAAGT | 55 | 54 | 333 | unknown protein, *Arabidopsis* | | | CA159045^A^ | UGSuM158 | (GGC)7 | CDS | AGAGAGGAGAGAGAGAGGAGG | AAACTTGGTGTAGCAGAAGA | 55 | 52 | 400 | pantoate-beta-alanine ligase, *Arabidopsis* | | | CA160203^A^ | UGSuM159 | (GCG)7 | 5'UTRs | CCTCCAACTCTCTTCTTGTCT | AGGGAACCATCCTCAATCT | 55 | 55 | 170 | hypothetical protein, *Oryza* | | | CA162577^A^ | UGSuM160 | (CGG)7 | 5'UTRs | CTGTGAGGAGGCTGTCAA | ATTTGGAAGGCAGGAAAG | 55 | 55 | 262 | holocarboxylase synthetase, *Triticum* | | | CA164308^A^ | UGSuM161 | (CCG)7 | 5'UTRs | ACATAAACTGGGTCACAATCA | CAGCAGGGCAGAACAATC | 55 | 57 | 173 | Protease inhibitor/ /LTP family protein, *Arabidopsis* | | | CA164379^A^ | UGSuM162 | (CAG)7 | 5'UTRs | CAAGAATGAAAGGTGAGAGAA | CCGAGAAAGCCTATGAACT | 54 | 54 | 323 | MYB transcription factor TaMYB1, *Triticum* | | | CA168020^A^ | UGSuM163 | (GGC)7 | CDS | CTGGCAACTTACAGCACC | CCACCTTCAGACAAATACAGA | 55 | 55 | 286 | Zinc finger, RING-type, *Medicago* | | | CA182783^A^ | UGSuM164 | (AAG)7 | CDS | AACAAGGTAGATGATGCCAA | GAGTGGGTTCCGTGGTTC | 55 | 58 | 393 | ubiquitin-onjugating enzyme E2, *Oryza* | | | CA182808^A^ | UGSuM165 | (GCC)7 | CDS | GAACCACGGAACCCACTC | CTACGACCACCAGTCACAC | 58 | 54 | 158 | DNA-dependent RNA polymerase II, *Arabidopsis* | | | CA183660^A^ | UGSuM166 | (AGA)7 | CDS | AATCGGACAATGAGCAGA | GAGGCGAGGTGAGGTGAG | 54 | 59 | 278 | harpin-induced family protein, *Arabidopsis* | | | CA184288^A^ | UGSuM167 | (CGC)7 | CDS | ATCTTTCTCGTCCGCCTC | CCTTGGTTCGGTCTATGTT | 57 | 55 | 296 | Enoyl-CoA hydratase, *Arabidopsis* | | | CA185217^A^ | UGSuM168 | (CGG)7 | CDS | GAAGAGCAAGACGGACCT | GAAAGAAACAGCCGATGA | 55 | 54 | 390 | unknown protein, *Arabidopsis* | | | CA195674^A^ | UGSuM169 | (CCG)7 | CDS | GTCCACGCCTTGCCTAAAC | AAGTTCCTCGCTATGTCTC | 60 | 51 | 260 | 4'-phosphopantetheinyl transferase, *Arabidopsis* | | | CA207386^A^ | UGSuM170 | (GCC)7 | CDS | CAAGGACATTACAAGAATAGGA | GCTGAAGTAGAAAGCACACC | 53 | 55 | 190 | beta-ketothiolase | | | CA213291^A^ | UGSuM171 | (CGG)7 | CDS | GAGGAGAAGAAGGAGGAGG | TATTTCCCACCAACAAGCA | 55 | 57 | 228 | nicotianamine aminotransferase, *Hordeum* | | | CA216941^A^ | UGSuM172 | (GCC)7 | 3'UTRs | TAACACACAAGGCAGCAGT | CCCGAGAAAGCAGAAGGG | 55 | 60 | 328 | unknown protein, *Arabidopsis* | | | CA243256^A^ | UGSuM173 | (GAG)7 | 5'UTRs | TAGTGAGGTGGTGGAGAC | GGAAGAGAGCGAGAGGAC | 55 | 54 | 138 | Expressed protein, *Oryza* | | | CA252244^A^ | UGSuM174 | (CGC)7 | CDS | CCCTTTCTCTTCTGTCCTTG | CTGTGAGTGTCCCGCTTG | 56 | 58 | 295 | leucine-rich repeat protein, *Arabidopsis* | | | CA258475^A^ | UGSuM175 | (CGC)7 | CDS | GGAAATGAACACACCCAGT | AGGAGGGCAAGGACTACC | 55 | 56 | 282 | expressed protein, *Oryza* | | | CA258985^A^ | UGSuM176 | (AGC)7 | CDS | CTCTCTCACATCGCAGTC | GGCAAGGACGACAGGCTC | 52 | 61 | 335 | hypothetical protein, *Solanum* | | | CA280782^A, B^ | UGSuM177 | (TGC)7 | CDS | GGTGCTGTCCCTATCACTAC | GCCCTTGTTTCTTTGTCTACT | 54 | 55 | 226 | hypothetical protein, *Oryza* | | | CA291445^A, B^ | UGSuM178 | (GAC)7 | 5'UTRs | GGACTACTACGACTACTGCGA | ACCTTGCTTACATCTTCCTCT | 54 | 54 | 201 | O-diphenol-O-methyl transferase, *Medicago* | | | CA291864^A^ | UGSuM179 | (CCG)7 | 5'UTRs | ATGATTGCCTGAGCCTTT | CGGAGAGAGAGAGAGAGAGA | 55 | 53 | 331 | actin depolymerizing factor, *Sorghum bicolor* | | | CA128485^A^ | UGSuM180 | (ATAC)5 | 3'UTRs | ATATATAGATGGATGGTGACACAG | CTCTAGTATAGCAGTGTCGTTTGT | 54 | 54 | 249 | hypothetical protein, *Oryza* | | | CA282658^A^ | UGSuM181 | (CACG)5 | 5'UTRs | TTTCTTTGGTTATACTGACTTGAC | GGGACAACTAATGTAACTGATTCT | 54 | 55 | 170 | hypothetical protein, *Oryza* | | | CA105120^A^ | UGSuM182 | (GCCG)5 | 5'UTRs | GTTAAGCTACTATGGACAACAGG | ACTTAACACTATGTCAGGTCTCAA | 55 | 54 | 334 | hypothetical protein, *Oryza* | | | CA284281^A^ | UGSuM183 | (GCAC)5 | 3'UTRs | TACTAACAATCTGACATGTTCTCC | TATTAGTCATCTGATCTGAGTGTG | 55 | 53 | 163 | hypothetical protein, *Oryza* | | | CA252565^A^ | UGSuM184 | (GACC)5 | CDS | GACAGACATACACACGGTACAC | GTGGTGGTCACTCACAATCACT | 55 | 59 | 199 | hypothetical protein, *Oryza* | | | CA240580^A^ | UGSuM185 | (CAAA)5 | CDS | GACAGACATACACACGGTACAC | GAACCCGAGGTAACGTACCGGC | 55 | 67 | 348 | hypothetical protein, *Oryza* | | | CA244023^A, B^ | UGSuM186 | (AG)10 | CDS | AACATTTCGGCATTTGAAGC | GGTCTTTCTTGGGGATCTCTC | 55 | 55 | 160 | ubiquitin C-terminal hydrolase, *Oryza* | | | CA231668^A, B^ | UGSuM187 | (CT)10 | CDS | CAACAATTGTCGAAGCCTCTC | TTTGCTTACCCCCTGTTGAC | 56 | 55 | 500 | ATP synthase delta' chain, *Oryza* | | | AY596560^A, B^ | UGSuM188 | (GA)10 | 3'UTRs | CCCAAGCGAGCTAGAGAGAG | TCTTCTTTCCTTCGCACAGC | 66 | 55 | 470 | hypothetical protein, *Oryza* | | | CA241232^A, B^ | UGSuM189 | (CT)10 | CDS | CCGCGACTCTCCTCTCTCT | GTTCTTCTCGGCGTTCCTC | 55 | 54 | 500 | auxin-regulated protein, *Arabidopsis* | | | AY596593^A^ | UGSuM190 | (CT)10 | 5'UTRs | CTACTAGCTTACATACAATCCCAA | ACAGATTCTTCTTCTTCAGCTAGT | 54 | 55 | 287 | polygalacturonase, *Oryza* | | | CA130851^A^ | UGSuM191 | (CA)10 | 3'UTRs | GTGTAATGATTCTTTGAACAAGAT | ATATAATGGCAATCCAAATGATA | 54 | 54 | 289 | Andropogoneaeine nucleotide-exch protein, *Zea mays* | | | CA271033^A^ | UGSuM192 | (TG)10 | 5'UTRs | CTCCTTCCTTGGTCCTCTCCTAC | AAAGAGCGAACAGCTTACGGGC | 65 | 61 | 353 | beta-D-galactosidase, *Sorghum* | | | CA179066^A^ | UGSuM193 | (GA)10 | 3'UTRs | AAGGTGTGAGCTAAACCTAAAC | GTCCTAAGAAACCGTACTAAATGA | 56 | 54 | 383 | hypothetical protein, *Oryza* | | | CA066249^A^ | UGSuM194 | (TCGG)5 | CDS | ACTTAGTCTCTTCTTAACCACTGC | AAGTAATTATCTATAGTGCCACCC | 55 | 55 | 178 | ferrodoxin NADP oxidoreductase, *Pisum* | | | CA128485^A^ | UGSuM195 | (ATAC)5 | CDS | CTTTCCTTTCTCTTCGGG | ATGCTCACCTTCACATACAG | 54 | 53 | 356 | hypothetical protein, *Oryza* | | | CA133642^A, B^ | UGSuM196 | (AAAG)5 | 5'UTRs | GCTACTATGGACAACAGGG | ATGAAGAGACGAGACGAAGA | 53 | 55 | 120 | cinnamoyl CoA reductase, *Arabidopsis* thaliana | | | CA134472^A, B^ | UGSuM197 | (GA)10 | 3'UTRs | GAAGGAGCAGCAGCGCCAGT | GATTTGCCGTCCTAGGGTTT | 57 | 55 | 289 | epsin N-terminal homology domain, *Arabidopsis* | | | CA143556^A^ | UGSuM198 | (TA)10 | 5'UTRs | GCTCTTCGTCTGGCTCCC | GATGCTCTTCAGTGTCTGTTG | 61 | 55 | 242 | regulatory protein, *Oryza* | | | CA182263^A^ | UGSuM199 | (GCGT)5 | CDS | GGTTGGGTTCTGAGGAGG | CAGGGCTTTATTCTGTCATT | 57 | 54 | 366 | PSBY (photosystem II BY), *Arabidopsis* | | | CA213297^A^ | UGSuM200 | (CTCC)5 | CDS | TTCATCCACAAGGACAAGAC | ACCGTTACCATAGCATACAAA | 55 | 54 | 178 | AtTLP5 (TUBBY LIKE PROTEIN 5); *Arabidopsis* | | | CA231668^A^ | UGSuM201 | (CT)10 | CDS | CGTGGATGTGTGTATTTCTTG | AGGATGATGTGATAAAGTGGA | 56 | 54 | 112 | hypothetical protein, *Vitis vinifera* | | | CA239324^A^ | UGSuM202 | (CGGC)5 | 5'UTRs | ACCTGCTGTAACGGATTTAGT | ATGATTGACCCGAGGATT | 55 | 55 | 299 | unknown protein, *Oryza* | | | CA244023^A^ | UGSuM203 | (AG)10 | 3'UTRs | CACAACGGAAATCATACACA | CAGAAGGTGACAGTGAAAGG | 55 | 55 | 383 | Ubiquitin carboxyl-terminal hydrolase, *Arabidopsis* | | | CA248154^A^ | UGSuM204 | (AGGA)5 | 3'UTRs | GTGAACGAATCCATCGCC | TTGAAACCACCGTAAATAAGA | 60 | 55 | 248 | unknown protein, *Arabidopsis* | | | CA281012^A^ | UGSuM205 | (TTCC)5 | CDS | CCCACACACACAACGGTC | TCAAGGTCGTCTTCTGCC | 59 | 57 | 257 | unknown protein, *Arabidopsis* | | | CA282658^A^ | UGSuM206 | (CACG)5 | CDS | ACCGTCACCAGCAACAAC | ATTAGCAGCCTTCAGCATAG | 57 | 55 | 271 | pectate lyase family protein, *Arabidopsis* | | | AJ969049^A^ | UGSuM207 | (CCTT)5 | CDS | AGAAGGTGATCCTCAAGGACAAG | AACTGATCCCTCTTTCATATATTC | 61 | 54 | 387 | glycosyltransferase, *Saccharum* | | | AY644469 | UGSuM208 | (GGC)6 | CDS | ATCGATCATTTCCTCTTCTTAGTA | AATTTGATTCATAGGATTGTGAG | 55 | 55 | 383 | mitochondrial malate translocator, *Oryza* | | | BQ536978 | UGSuM209 | (GCA)6 | CDS | AATCGGCGCTGACCATGGACTC | AGAACACAACTTTCACCTTGTT | 69 | 55 | 354 | hypothetical protein, *Oryza* | | | BU103565 | UGSuM210 | (CAG)6 | CDS | GTTCTTAGTCCAGCCGTAGTTGT | TCAAATTTCTCAGAACCTTCAC | 54 | 56 | 363 | BAG domain-containing protein, *Oryza* | | | CA065436 | UGSuM211 | (CGC)6 | 5'UTRs | AATTTAGGGTTTGTTCGCCTCT | ATGTGTAAATCTGAACTTCATCCT | 55 | 54 | 313 | hypothetical protein, *Oryza* | | | CA068470 | UGSuM212 | (TAC)6 | 5'UTRs | TTGGTAAGTGGTAACACATAATTT | CTACATCCAAGAACAAATACAAAC | 55 | 54 | 286 | peroxidase2, *Medicago sativa* | | | CA071226 | UGSuM213 | (TCC)6 | 5'UTRs | AGATATAACACACACACACACAAA | GGCCATCGAGGAGGAGTTCAAG | 55 | 55 | 270 | cellulose synthase family protein, *Arabidopsis* | | | CA074021 | UGSuM214 | (GCG)6 | CDS | ACTTCGCCTACACGCTCGATTC | GTCATTTGACTCCTTCAGTTCA | 64 | 56 | 380 | leucine zipper transcription factor, *Arabidopsis* | | | CA076912 | UGSuM215 | (CGC)6 | 5'UTRs | TAATATACATCACGACACAACAAA | TTGTGTTATCTAGACGATACCTTT | 55 | 54 | 348 | hydrolase, *Arabidopsis* | | | CA082494 | UGSuM216 | (CCT)6 | CDS | GTCATCCTGTTCGACATCGGGTT | TTCTAGGTAAGATACCTTTCAAAC | 66 | 53 | 362 | RAB GTPase activator, *Arabidopsis* | | | CA084828 | UGSuM217 | (CGC)6 | CDS | GAGAAGAAGAAGAAGAAGAGGAG | GATGAAGATCAGCTTGTTGGAC | 55 | 58 | 167 | unknown protein, *Arabidopsis thaliana* | | | CA085964 | UGSuM218 | (TGC)6 | CDS | ATTACAGGCTTACACTTACAACAA | ATAGCAGCGTGGAGTCCTACTT | 55 | 59 | 364 | Eukaryotic transcription factor, *Oryza* | | | CA086148 | UGSuM219 | (GA)9 | CDS | ACCTCCACCTCCACCTCAGTTC | CGTTCAGCTTCAGGGTGTCGAT | 64 | 65 | 397 | serine/threonine protein kinase, *Arabidopsis* | | | CA086833 | UGSuM220 | (GCT)6 | CDS | ATAGTTACTGCCTTTAAGTTTGGT | TATTTCATGAGAGAGTTTAGCAAG | 54 | 54 | 363 | unknown protein, *Arabidopsis* | | | CA093362 | UGSuM221 | (GGC)6 | CDS | ATACTTCTCGATTAATCACCGAT | GCTCGGTCATCATCACTACTAC | 56 | 58 | 145 | dynein light chain, *Oryza* | | | CA096260 | UGSuM222 | (GAG)6 | CDS | TAGCAATCTACTCCCTACGTCTAC | GTTGACGTTGATCAGCCCGTTG | 56 | 67 | 378 | small nuclear ribonucleoprotein Prp4p, *Oryza* | | | CA098361 | UGSuM223 | (GCG)6 | CDS | GTATATGACCCTCCTATTACTGCT | AGTGTATGCGTTACCATATAGAGA | 54 | 54 | 276 | expressed protein, *Oryza* | | | CA103033 | UGSuM224 | (CCG)6 | 5'UTRs | GGCAGAGCCTCAAGAAGAAGGG | ATTTATGCTTGCTTCACAAGTC | 55 | 61 | 353 | splicing factor PRP38 protein, *Arabidopsis* | | | CA103708 | UGSuM225 | (CCA)6 | CDS | TTTCTTCGTCTCCTCTTCCTCC | ATTCCAGACGAGCTCCAGAATTT | 66 | 55 | 190 | inorganic pyrophosphatase, *Oryza* | | | CA103873 | UGSuM226 | (GA)9 | CDS | AAGTACTACTGCACTGTCATTGAT | ACTTCCTTCACAATCTCATCATA | 55 | 60 | 200 | myosin heavy chain, *Oryza* | | | CA104473 | UGSuM227 | (TG)9 | CDS | AGACCAACTCGAGAGATGAGCAC | ACAAGCCAATATACCTCTGTAGTC | 55 | 65 | 308 | elicitor inducible beta-1,3-glucanase, *Arabidopsis* | | | CA108086 | UGSuM228 | (TTC)6 | CDS | GAGCTTTGCATGATCTCTCGAT | TACTCCTCCCTATACATTGATACA | 56 | 55 | 230 | homeobox transcription factor GNARLY1, *Zea mays* | | | CA110388 | UGSuM229 | (GCG)6 | CDS | CTGCTACTAGCTTACATACAATCC | ACAGATTCTTCTTCTTCAGCTAGT | 54 | 55 | 307 | hypothetical protein, *Oryza* | | | CA110734 | UGSuM230 | (CGA)6 | 5'UTRs | GTAGTACTGCTTATTATCCATCCC | CACTGCTCCAGAAGGTAAAGAG | 55 | 68 | 366 | eukaryotic rpb5 RNA polymerase family, *Arabidopsis* | | | CA112580 | UGSuM231 | (GGC)6 | CDS | AGTACCATCGCTTAAGTAAAGTCT | ATTAAAGGACCTTACTCAGTTACC | 61 | 55 | 113 | zinc finger family protein, *Oryza* | | | CA115807 | UGSuM232 | (AC)9 | CDS | CCTTGGTTCGTTTATTCTTTACTA | AAGAGGATCCATCGGTCAGTGT | 62 | 61 | 336 | Homeobox protein HD1, *Oryza* | | | CA117569 | UGSuM233 | (CGC)6 | CDS | CTTGCATGAGCATGAGACAG | TTCTTTGTGTTATTCCAAGTCA | 54 | 59 | 226 | seryl-tRNA synthetase, *Zea mays* | | | CA117618 | UGSuM234 | (GCC)6 | CDS | CTGAGGTGAAATTATCGTGTGT | GCAACGTCTAAATATAATTGCTAA | 55 | 54 | 104 | fiber protein Fb2, *Gossypium* | | | CA118738 | UGSuM235 | (CCG)6 | CDS | ACGCCACGCTGGATTAGACTCAG | TCAGGTTCTTTATATCTGTTGAAA | 55 | 54 | 111 | hypothetical protein, *Oryza* | | | CA119630 | UGSuM236 | (CGG)6 | CDS | CTACTAGCTTACATACAATCCCAA | ACAGATTCTTCTTCTTCAGCTAGT | 54 | 55 | 307 | chaperone protein DnaK, *Arabidopsis* | | | CA120436 | UGSuM237 | (AGG)6 | 3'UTRs | ACGGTAAGGCACTAAATCTCT | GTCGTCGTCTGTGGTGTT | 54 | 55 | 355 | ubiquitin-protein ligase, *Arabidopsis* | | | CA121729 | UGSuM238 | (CGC)6 | 3'UTRs | GTCTGATGAACTTGTGGGAA | AATCTTATTTGGACTCGTGGT | 55 | 55 | 195 | p53 binding protein, *Oryza* | | | CA124518 | UGSuM239 | (GGC)6 | CDS | CCATCAGAGAGGAGTAGCAG | ATTCCAAGTTCACAAGTTCA | 55 | 53 | 365 | Gar1 RNA-binding region family protein, *Arabidopsis* | | | CA125127 | UGSuM240 | (TGT)6 | CDS | AGTTGAAGCCGAGAAAGAA | CGTAGAAGAGCGGGATGT | 55 | 56 | 376 | phosphatase 2C, *Arabidopsis* | | | CA126381 | UGSuM241 | (CTG)6 | CDS | ATCGCTAACTCATTCATCATC | GCCAGACGCATTCAAACA | 54 | 59 | 348 | annexin max4, *Arabidopsis* | | | CA128394 | UGSuM242 | (GCA)6 | CDS | CATAGCAAGCACCACCTC | TCTTCTTCTCGTCCACCC | 55 | 56 | 263 | Homeodomain-like; Uridylate kinase, *Medicago* | | | CA129005 | UGSuM243 | (GCC)6 | CDS | GTGTTCGTCTTCCTTGACC | GGCTTTGTAGTTTGCGTATC | 55 | 55 | 324 | nitrate-induced NOI protein, *Zea mays* | | | CA129032 | UGSuM244 | (GAG)6 | CDS | CTTCATACGCCACCTTCTC | CAAATGTTCACTCGCATCA | 55 | 56 | 238 | hypothetical protein, *Oryza* | | | CA129620 | UGSuM245 | (GA)9 | CDS | ACACCTTTACGACCATCAAC | GAATGAGAACCCAATACCAG | 54 | 54 | 375 | unknown protein, *Oryza* |  | | CA131272 | UGSuM246 | (GGC)6 | CDS | CCGCTTCCTTCACCTAAAC | AACACATTTCAGCCTACCC | 57 | 55 | 347 | prephenate dehydratase, *Oryza* | | | CA134573 | UGSuM247 | (CGA)6 | 3'UTRs | CATTTGCTGCTATTCGGT | CTGGTTATGAAACTGCCTTC | 54 | 54 | 234 | ethylene responsive protein, *Oryza* | | | CA135266 | UGSuM248 | (GGC)6 | 3'UTRs | GTTCCAAGTTACAGACCAGAA | GATAAGAAAGATGTCGTCGCT | 54 | 56 | 103 | hydrolase, alpha/beta fold family protein, *Arabidopsis* | | | CA136653 | UGSuM249 | (CGG)6 | 3'UTRs | CTTTGCTTCCTTCGCATC | TTGTGATTTGGTGTCTTGTG | 57 | 55 | 390 | *ATCUL3A (Cullin 3A)*, *Arabidopsis* | | | CA137576 | UGSuM250 | (CGG)6 | CDS | CAGAGCATCACCAGCACC | CTTGAGCAGCGTCTTGTT | 58 | 55 | 135 | ribosomal protein S6 family protein, *Arabidopsis* | | | CA138000 | UGSuM251 | (ACG)6 | CDS | GCTTCCTCGCTCCTCCTC | TACTTCTACCTCGTCTGCTTC | 60 | 54 | 257 | ATPP2-A13, *Arabidopsis* | | | CA139791 | UGSuM252 | (AGC)6 | 5'UTRs | CTGTTTCCTTCCTTCTCGT | CAATCATAGCCCAGACACC | 54 | 56 | 280 | protease inhibitor protein, *Arabidopsis* | | | CA141587 | UGSuM253 | (CT)9 | 5'UTRs | TATTCAGTCATTCGTTTCGTT | GTTCCATACAAGCAGTAGCC | 54 | 54 | 228 | senescence-associated protein, *Arabidopsis* | | | CA142747 | UGSuM254 | (AGG)6 | 5'UTRs | CTCCTTGTTACTGGACCTTTC | AGGCAAACCGACTATTGAG | 55 | 55 | 392 | unknown protein, Oryza | | | CA142982 | UGSuM255 | (GCC)6 | 5'UTRs | ACTCATCCATCCAGTCCA | ATCTTCTTGCCCTCCTTCT | 54 | 55 | 199 | MATE efflux family protein, *Arabidopsis* | | | CA143331 | UGSuM256 | (CCG)6 | 5'UTRs | GTAAAGAGGGTGTTCCGTTCT | TATTTGAAGTTATGGGCTTGA | 57 | 55 | 138 | peptidoglycan-binding domain, *Arabidopsis* | | | CA144976 | UGSuM257 | (CAA)6 | CDS | CTGGCTACATTCACAGGATT | GGTCGGTTGAGTTATGATGAG | 55 | 57 | 338 | C2H2-type zinc finger protein, *Oryza* | | | CA147526 | UGSuM258 | (CCG)6 | CDS | CACACTGACACCTACCAATGA | GCCAAATACAACGAACGA | 56 | 55 | 262 | eukaryotic translation initiation factor3, *Arabidopsis* | | | CA148808 | UGSuM259 | (CAG)6 | CDS | CGTTATGGAAAGCACGAC | CTTGATGCCGTTGAAGAA | 55 | 55 | 271 | unknown protein, *Oryza* | | | CA150692 | UGSuM260 | (CCG)6 | CDS | AATCTGGACTGCTTGGTTC | AGTGTCTTGTTCCTGGTGTC | 55 | 54 | 184 | histone acetyltransferase HAT2, *Triticum* | | | CA152704 | UGSuM261 | (CCG)6 | CDS | AAGATTCCAAACGCTGAA | AGAGATAGACTCAAAGGGCAA | 54 | 55 | 325 | LCV1 (LIKE COV 1), *Arabidopsis* | | | CA152791 | UGSuM262 | (CGT)6 | CDS | GAAGAGGAGGAGAGGAGAAG | TGGGATGGTTGTTGACTG | 54 | 56 | 233 | Tetratricopeptide-like helical, *Medicago* | | | CA157525 | UGSuM263 | (GTG)6 | CDS | TCGTCCAACAAACATAGACA | TGCTACACCATTGAAAGTCAG | 55 | 56 | 258 | mucin 2, *Arabidopsis* | | | CA157937 | UGSuM264 | (GCA)6 | CDS | CGCTCACATCACCTCCTAC | ACGAGAAACGACGACCTG | 56 | 57 | 348 | unknown protein, *Oryza* | | | CA159463 | UGSuM265 | (TGC)6 | CDS | TCTGGCTGTCTACTCTTCATT | CTTCTCCTCTTCAGTCTCCTC | 54 | 54 | 244 | fatty acid desaturase, *Oryza* | | | CA161902 | UGSuM266 | (GCC)6 | 5'UTRs | TACACCCAAGCCCTAAACAC | GAGGAGATCATCTTGTCCAT | 55 | 55 | 400 | E-class P450, *Medicago* | | | CA163234 | UGSuM267 | (CAG)6 | 5'UTRs | CTTCTCCAAATCAACCACC | TACACCAGCACAGCACAG | 55 | 54 | 252 | unknown protein, *Oryza* | | | CA164032 | UGSuM268 | (GCA)6 | 5'UTRs | ACCCTCATAGCACAGATTACA | CATAGTGGCGAGTCTTGG | 54 | 55 | 127 | basic helix-loop-helix family protein, *Arabidopsis* | | | CA164419 | UGSuM269 | (GCC)6 | 5'UTRs | ATTCCATCATTTCGGCAC | CTTCTTCCCAGACACCAAG | 56 | 55 | 251 | CBS domain, *Oryza* | | | CA164558 | UGSuM270 | (CGC)6 | 5'UTRs | GAAAGGGTGCCTGGTAGT | GTTGACAGAAGAGAGAGAGCA | 55 | 54 | 212 | proline-rich 14 kDa protein, *Oryza* | | | CA165184 | UGSuM271 | (CGC)6 | 5'UTRs | GCCAATACTTCACTTCCTG | GCTCTCTGTTGTCAGTCTCC | 53 | 54 | 299 | unknown protein, *Arabidopsis* | | | CA165631 | UGSuM272 | (GAC)6 | CDS | AGGAAAGGAAAGCAAGAG | CACCTCACTTCAGACATACAC | 52 | 52 | 400 | hypothetical protein, *Oryza* | | | CA165670 | UGSuM273 | (CAG)6 | CDS | AACCAATACAATACGAACAGA | TGATGAAACTGAAGGGAGG | 52 | 56 | 383 | Expressed protein, Oryza | | | CA167449 | UGSuM274 | (CAG)6 | CDS | ACTCTAATGACTCTTCTTCCCA | GTGAATGCTGCTTACTTTGTC | 54 | 55 | 308 | Nitrate reductase cytochrome c-type, *Arabidopsis* | | | CA168796 | UGSuM275 | (TCC)6 | CDS | AACTTGACCCTTCTTCTTCC | GCCGATGGACACCTTGAC | 54 | 60 | 351 | Alpha-expansin 13 precursor (At-EXP13), *Arabidopsis* | | | CA168814 | UGSuM276 | (CAT)6 | CDS | GAACATACCAGAGTTGGCAG | CAAGGAGATTAGCATCAGAGA | 55 | 54 | 291 | GAT; ENTH/VHS, *Medicago* | | | CA169369 | UGSuM277 | (CTC)6 | CDS | ATGATGACGAGAACGATG | GCAAGGGTGAGCGTGGAA | 52 | 63 | 296 | 3-methylcrotonyl-CoA carboxylase 1, *Arabidopsis* | | | CA172292 | UGSuM278 | (GCC)6 | CDS | TACTGCTCTCCTTACAGCGAC | CCTCCATCTCTTCCACAAC | 57 | 55 | 169 | Expressed protein, *Arabidopsis* | | | CA174479 | UGSuM279 | (CCT)6 | CDS | GTTTAGGGTTCGTTAGGGTT | ACTATGGGTGCGTGGAAG | 54 | 57 | 226 | unknown protein, *Oryza* | | | CA175191 | UGSuM280 | (GCT)6 | CDS | GATAGAATGACTGGACGGAG | TATGACCTTAGCATCAAGCAC | 54 | 55 | 309 | DEAH (Asp-Glu-Ala-His) box polypeptide, *Arabidopsis* | | | CA175391 | UGSuM281 | (CCT)6 | CDS | TTTACTGGAGAACCACCTGA | GGGAAGACCATCACATCC | 55 | 55 | 362 | *ATBPM5* protein, *Arabidopsis* | | | CA176425 | UGSuM282 | (ATG)6 | CDS | GATTCCGCTTCCGCTTCC | TTGAAACCCTCCAAGAGATAG | 63 | 56 | 338 | hypothetical protein, *Oryza* | | | CA179488 | UGSuM283 | (GGC)6 | CDS | CTTCTTCCACAAACGCAC | CAGCGAACACAGAGATGTAG | 55 | 54 | 341 | Mitochondrial import inner membrane, *Arabidopsis* | | | CA180693 | UGSuM284 | (GCG)6 | CDS | ATCCATTAGGTCTTCCTTCTC | CTACTTCAATCTCCTTGTCCC | 54 | 55 | 338 | Zinc finger, C2H2-type, *Medicago* | | | CA183455 | UGSuM285 | (GGC)6 | CDS | CCTTGATGTTCAGATAGTTGG | CCGATTCAGCCCTTCGTC | 54 | 61 | 379 | unknown protein, *Oryza* | | | CA184172 | UGSuM286 | (GCG)6 | CDS | GAGATTCGTCGCCTCAGT | GGAAGGGTAGAGACAGGTAGA | 56 | 55 | 275 | unknown protein, *Oryza* | | | CA186537 | UGSuM287 | (CGC)6 | CDS | GGGTGTGAAGACAACTGAAA | GGACAACAGGGAGAAGAGG | 56 | 57 | 293 | Zinc finger, C3HC4 type family protein, *Arabidopsis* | | | CA188285 | UGSuM288 | (CCG)6 | CDS | TTAGAACAGGAGAGTGCTTGA | CAGAGTGGGAGTGAGTCGT | 55 | 56 | 132 | Lipase, *Oryza* | | | CA191141 | UGSuM289 | (GCG)6 | CDS | GTGGGTCGTCTTGTCCTC | AAGGTGTTCCATACAGCAA | 56 | 54 | 302 | uroporphyrinogen III methyltransferase, *Zea mays* | | | CA194346 | UGSuM290 | (GCG)6 | CDS | TCCTGGTGGCAGTTGTAG | TTGGGCTTTGTGGAGTCA | 55 | 58 | 368 | histone deacetylase HDA101, *Zea mays* | | | CA195760 | UGSuM291 | (CCT)6 | CDS | ATAAATGTCTTGAGGGTGCT | ACCAAATGCCAGGTGTTC | 53 | 56 | 213 | protein kinase, *Oryza* | | | CA195823 | UGSuM292 | (GAC)6 | CDS | GTTCCGTCCAACCCACCA | GGTCTCTCCTGTCTCCAAA | 62 | 55 | 287 | Expressed protein, *Oryza* | | | CA196460 | UGSuM293 | (AGC)6 | CDS | ACCTCAGTTCCCGTTCTC | GTCCGTGGTGGTGCTTGG | 54 | 63 | 238 | ramosa 2, *Zea mays* | | | CA196495 | UGSuM294 | (CGG)6 | CDS | CCTTGTGCGTGTCTCTCC | TTCCACTACCCTCTTTGTTG | 57 | 55 | 190 | calcium-dependent lipid-binding protein, *Arabidopsis* | | | CA198028 | UGSuM295 | (GCC)6 | CDS | GATTCAACCCTTGTGATTTCT | CACTTACCTTCATCTCCAACA | 55 | 55 | 278 | hypothetical protein, *Oryza* | | | CA206115 | UGSuM296 | (CGC)6 | CDS | ACCTCCACCTACACCTACTG | GCCATACTACACCTCCAAG | 54 | 53 | 386 | putative kinase, *Oryza* | | | CA206236 | UGSuM297 | (GCC)6 | CDS | ATGCTCTCTCTCTTCTGTCAA | CATCAGGTCGTAGTGGGA | 54 | 55 | 323 | Hsp20/alpha crystallin family protein, *Arabidopsis* | | | CA211984 | UGSuM298 | (GCA)6 | CDS | ACCAGTTCCTCTACGCCC | CATCCCATCCCTTGTGTC | 57 | 57 | 184 | FHA domain containing protein, *Oryza* | | | CA212110 | UGSuM299 | (CGC)6 | CDS | CTTCTTCTTCTTCTTCTGGCT | TAGACCCTCCACTTGTTTC | 54 | 52 | 242 | Ornithine carbamoyltransferase, chloroplast, *Arabidopsis* | | | CA213302 | UGSuM300 | (CGG)6 | CDS | ATCTTCACATCCATCATCCAC | ATCTCTCCTTGCTTTGGTTT | 57 | 55 | 201 | SR-rich pre-mRNA splicing activator, *Oryza* | | | CA213570 | UGSuM301 | (TGC)6 | CDS | AACACACACACACACACACAC | ACTAATCTCTCCTTGCTTTGG | 56 | 55 | 160 | peroxidase, *Arabidopsis* | | | CA214701 | UGSuM302 | (CAC)6 | 3'UTRs | CACTCTCGGTGTAGTTGCTC | CGGTCTCATTCTTCCTTTC | 56 | 54 | 331 | pyrophosphate- phosphofructokinase, *Arabidopsis* | | | CA219031 | UGSuM303 | (AAG)6 | 3'UTRs | CTGGAAGATTGGTGGTTG | CCGTAGGACGAGATGTGA | 54 | 55 | 143 | fasciclin-like protein FLA5, *Triticum* | | | CA220974 | UGSuM304 | (GCG)6 | 3'UTRs | CAAGCAGAGCAGACAGGA | CTCCACCGAGAGCACCTT | 56 | 58 | 298 | basic helix-loop-helix family protein, *Arabidopsis* | | | CA221475 | UGSuM305 | (CCG)6 | 3'UTRs | TAGCCCAGGTTTCCAAGT | GATACGCCTTCTTCCTCC | 55 | 54 | 399 | 40S ribosomal protein S24, *Zea mays* | | | CA223280 | UGSuM306 | (TGC)6 | 3'UTRs | CAAGAACCGCCTCCTCTC | TTCCAACCAACAGACACAG | 58 | 55 | 285 | hypothetical protein, *Oryza* | | | CA224596 | UGSuM307 | (GAC)6 | 3'UTRs | CAAGACAATCTCAGCAAGAA | CAGGAATCATAACCAACAGG | 54 | 55 | 400 | EDA4 (embryo sac development arrest 4), *Arabidopsis* | | | CA227502 | UGSuM308 | (CCT)6 | CDS | AGGTCATCTCTCTCTTCTCGT | CTCCTTCTCCTCCTTCTTGT | 54 | 55 | 217 | unknown protein, *Oryza* | | | CA234609 | UGSuM309 | (ATC)6 | CDS | CGTTCGTCTCTCTCTCCTC | ATTTACAGGTCATCCCAAAC | 55 | 53 | 381 | small nuclear ribonucleoprotein, *Arabidopsis* | | | CA234669 | UGSuM310 | (CTC)6 | CDS | ATCGCTTCTACAGTCACCC | AACTTCTCCCTTTCTCCAAC | 55 | 54 | 185 | beta-galactosidase, *Glycine max* | | | CA236932 | UGSuM311 | (GCC)6 | CDS | GCTCTCCTCCTCCTCTCC | GCCACTTTATCATCCTCAGTT | 56 | 55 | 350 | ribosomal protein L30, *Triticum* | | | CA238788 | UGSuM312 | (AGA)6 | 5'UTRs | CTCTTCTACTCTTCAATCTGGTG | CCGTTTCTAACTTTCTATGGG | 55 | 55 | 395 | hydrolase, *Arabidopsis* | | | CA240049 | UGSuM313 | (CCA)6 | 5'UTRs | GACAGGCAGAGGAGAACAC | GTAGCCGAAGACCCACAG | 55 | 56 | 172 | oxysterol-binding protein, *Arabidopsis* | | | CA240507 | UGSuM314 | (CGC)6 | 5'UTRs | TTTGTGTCCTCTCTGTTCATT | GCAAGCATCAGTGTTCATC | 54 | 55 | 384 | microtubule-associated protein, *Nicotiana* | | | CA243480 | UGSuM315 | (CAG)6 | 5'UTRs | CGAGGTGGTGGTCGTCGT | TGTCTGCTTGATGAGGTTCT | 63 | 55 | 275 | auxin-induced protein TGSAUR22, *Arabidopsis* | | | CA244269 | UGSuM316 | (GCT)6 | 3'UTRs | AATACATCCTCAAGACCAACTT | CCGACCGTGAAGATGGAGTC | 55 | 55 | 383 | unknown protein, *Oryza* | | | CA244596 | UGSuM317 | (GCA)6 | 3'UTRs | ATTGAGAAGGTGGAGTTTGAG | GATTCCTCTGGCTTTGGT | 55 | 55 | 224 | calcium-binding protein, *Arabidopsis* | | | CA248168 | UGSuM318 | (GCG)6 | CDS | ATGGCGTCTCGTCTCGTT | ACCTCAGTCTTGTCTTCCTTC | 59 | 55 | 266 | hypothetical protein, *Oryza* | | | CA251749 | UGSuM319 | (GAG)6 | CDS | ACCCTCCTATTACTGCTGACT | AATGCTTCCTGTCCCTTT | 54 | 54 | 192 | hypothetical protein, *Oryza* | | | CA252297 | UGSuM320 | (TGC)6 | CDS | AAAGGGATTTATGATACCAATG | TTTGGAATTAAAGGTAGTGCTT | 56 | 58 | 295 | hypothetical protein, *Oryza* | | | CA252743 | UGSuM321 | (GCA)6 | CDS | TCCACAAACAGAAACAGTCC | CTACCGTGAGAAGCACCA | 56 | 55 | 222 | Expressed protein, *Oryza* | | | CA253068 | UGSuM322 | (AAC)6 | CDS | CTGGCTCTCTATCACCGAC | AGAATCAAATCAACCGCTC | 55 | 55 | 330 | Acyl-CoA synthetase, *Arabidopsis* | | | CA255856 | UGSuM323 | (AGG)6 | CDS | CTTCCCTCCCTCTCCTCT | AGCCTTCTACTAAACTATCTGCT | 55 | 53 | 396 | proline-rich cell wall protein, *Arabidopsis* | | | CA258232 | UGSuM324 | (GCC)6 | CDS | TAGCCGAGCGAGGGAAGG | TTCTTGTGTGGGATGGAC | 63 | 54 | 362 | cold shock protein-1, *Triticum* | | | CA258249 | UGSuM325 | (TC)9 | CDS | CACAACAGGACCAAGATGA | ACTCTCAACGGTATGGCTAA | 55 | 54 | 228 | plastid starch synthase I precursor, *Zea mays* | | | CA260851 | UGSuM326 | (GCG)6 | CDS | CAGGACTACAGGGAACAATAA | GAAATACCAGGCTCACTTCA | 54 | 55 | 342 | endoplasmic reticulum protein, *Arabidopsis* | | | CA261143 | UGSuM327 | (CGC)6 | CDS | TGAGGGAGGCGAAAGCGG | GCAGAGCACCACCGAGAC | 67 | 60 | 237 | Heat shock protein DnaJ, *Medicago* | | | CA267467 | UGSuM328 | (GCC)6 | CDS | GATACTCTTCTATTCGTCGCT | CACTGCTTCACAACATCATC | 53 | 54 | 266 | hypothetical protein, *Oryza* | | | CA270948 | UGSuM329 | (CGC)6 | CDS | CTTCTGATGATGGAGGCA | AGTGTTTCAAGCCAAATCC | 55 | 55 | 381 | thioredoxin, *Bombyx mori* | | | CA272322 | UGSuM330 | (ACC)6 | CDS | CTCTCTTCTCCACAGACCAA | AATCCTTCTATTCAACGCAAC | 55 | 56 | 286 | silencing group B protein, *Zea mays* | | | CA272418 | UGSuM331 | (GGC)6 | CDS | GCAACAGCGACATCTTCA | ACCTCCTCCTCTTTCTTCTTC | 56 | 55 | 238 | esterase, *Oryza* | | | CA272643 | UGSuM332 | (CCA)6 | CDS | CGCATCATCTTCATCTCTC | TGTTTATTTCATTTCATTCATC | 54 | 52 | 210 | hypothetical protein, *Oryza* | | | CA273285 | UGSuM333 | (CTC)6 | CDS | GTGTCTCCGTGCTGTATCTT | GCCTTGTTCTTCATCGTTT | 55 | 55 | 146 | giberellin regulated family protein, *Oryza* | | | CA276184 | UGSuM334 | (GTC)6 | CDS | TCTCAAGGATACACCATCAAG | ATCATCAGCACGACAGACA | 55 | 55 | 177 | legumin-like protein, *Zea mays* | | | CA280844 | UGSuM335 | (GAC)6 | CDS | CGCCAAGGAGGTGGACAA | AGGAAGCCGACGATGATG | 63 | 59 | 222 | leaf senescence protein-like, *Oryza* | | | CA282680 | UGSuM336 | (ATT)6 | CDS | AACAACGGATACAAATGAAAG | CGATTGATGGATGGTAATG | 54 | 54 | 299 | thaumatin-like protein, *Triticum aestivum* | | | CA283420 | UGSuM337 | (GCT)6 | CDS | TCCGTTCAGAGTGATGATG | AAGAAGCCGTGGAGGAAG | 55 | 57 | 288 | MADS-domain transcription factor, *Arabidopsis* | | | CA284697 | UGSuM338 | (GCT)6 | CDS | TAAGCGAGTGTGAGGGAG | GACGAGGATTTGTTCCAG | 54 | 53 | 218 | ATP-binding protein, *Arabidopsis* | | | CA288623 | UGSuM339 | (CGC)6 | 5'UTRs | CAATCACATACCCTCACATTC | CGGTGTTCTACGGGCTGG | 55 | 62 | 310 | hypothetical protein, *Oryza* | | | CA291716 | UGSuM340 | (CAG)6 | 5'UTRs | TATCGTAGCAGTTCGGTATG | GTCACCAGGGATGGAGGT | 54 | 58 | 272 | WRKY DNA binding domain protein, *Arabidopsis* | | | CA295137 | UGSuM341 | (GCC)6 | 5'UTRs | CGCTCTCTCGTAACAGGAC | GTTTAGATGTGTGTATGTGGG | 56 | 52 | 356 | myb family transcription factor, *Arabidopsis* | | | CA296022^B^ | UGSuM342 | (CAC)6 | 5'UTRs | GTTACCATCCCATCCCAC | TGTCCCTCGTTCACAGAC | 55 | 55 | 151 | hypothetical protein, *Arabidopsis* | | | CA297648^B^ | UGSuM343 | (CAG)6 | 5'UTRs | ACTCCTCCTCCTCGCCGT | TCTTGTTGTAGTAGCCCTTGT | 62 | 54 | 368 | SOUL heme-binding family protein, *Arabidopsis* | | | CA300679^B^ | UGSuM344 | (CTC)6 | CDS | CTATCCTCTTGTTGGGTCCT | TCCGCACCTCCGTTCACC | 55 | 65 | 162 | nucleoside diphosphate kinase family protein, *Arabidopsis* | | | CA084691^B^ | UGSuM345 | (TC)8 | CDS | TATACAAGAATGAAAGGTGAGAGA | AAGCATACTCCCTCTATCTCTATG | 55 | 55 | 217 | DC1 domain-containing protein, *Arabidopsis* | | | CA093455^B^ | UGSuM346 | (AG)8 | CDS | TATACGTAGTAGTGATGATGACCG | CTCCTTCGTCCAGTACCAGTAG | 60 | 64 | 341 | DNA-binding protein DF1, *Pisum* | | | CA110745^B^ | UGSuM347 | (CT)8 | 5'UTRs | TCTGGCTTTATCGTAACTTGTAT | GAGCCTCGTTTGGGTGGCTTTC | 55 | 56 | 374 | expressed protein, *Oryza* | | | CA112979^B^ | UGSuM348 | (CG)8 | CDS | CTACCTCCTCGTCTCCTCCCTCTT | AACAAGGAATATGGTCCCTGAG | 61 | 55 | 368 | unknown protein, *Arabidopsis* | | | CA116458^B^ | UGSuM349 | (TC)8 | CDS | CAAGATGTACCCGGACATGGCT | TGCTATACTAGCTATCTCCTTCCT | 55 | 55 | 204 | unknown protein, *Oryza* | | | CA120304 | UGSuM350 | (CA)8 | 3'UTRs | CTGTACTGGTATTACATGTGACCT | TCTACTAATCACAAGAGAAGATGC | 60 | 55 | 175 | unknown protein, *Arabidopsis* | | | CA127000 | UGSuM351 | (GT)8 | CDS | GCATCTATCGGTCTTCTGG | ATCCAATCCTTCATCTTCTTC | 55 | 54 | 314 | 3-alpha-L-fucosyltransferase, *Zea mays* | | | CA130732 | UGSuM352 | (AG)8 | CDS | ACGCGTAGGCCGTACCAAAG | GTTAAACCTCAGCCGTGAGT | 55 | 54 | 348 | hypoxia-responsive family protein, *Arabidopsis* | | | CA131353 | UGSuM353 | (GT)8 | CDS | ACATAGGCTTGAGGACCA | GGTTTGGGCGTTTCAGGG | 53 | 63 | 400 | unknown protein, *Arabidopsis* | | | CA131398 | UGSuM354 | (GA)8 | 5'UTRs | CACAAAGCATAATAAGGATGAA | GGTAGTGCCATTGAGGTTG | 54 | 56 | 302 | nodulin MtN21 family protein, *Arabidopsis* | | | CA148575 | UGSuM355 | (GA)8 | CDS | CTGCTGACTAAATCTGGCAC | TTCTTACTTCTGCTTCCCTCT | 56 | 55 | 359 | xyloglucan-specific glucanase, *Arabidopsis* | | | CA167204 | UGSuM356 | (GT)8 | CDS | AACAATGAGGTTGAAGATGAA | GAATGTAGTAAAGGGAAGGGA | 54 | 55 | 392 | hypothetical protein, *Oryza* | | | CA204826 | UGSuM357 | (CT)8 | CDS | GCTGACTTCGTTCTGACTTAC | TATTGGCTCTGGGATAGACTT | 54 | 55 | 300 | serine/threonine-protein kinase, *Arabidopsis* | | | CA205923 | UGSuM358 | (AC)8 | CDS | ACACATCGCTTTCCCACA | GCATACCTGTCGTCGTCT | 58 | 54 | 148 | APETALA2-protein, *Oryza* | | | CA239728 | UGSuM359 | (CT)8 | 5'UTRs | ACTGCTGCTCACCGACTT | AAGAATAATAACAACCGCACA | 56 | 55 | 267 | Homeobox protein cut-like 1, *Arabidopsis* | | | CA265205 | UGSuM360 | (TC)8 | CDS | AAGATAGGATGGGCAAGG | GAGACGGTGAAGATGCGG | 55 | 60 | 258 | unknown protein, *Arabidopsis* | | | CA280557 | UGSuM361 | (GA)8 | CDS | GACAGAGAACAGGAATCAACA | TAAAGTCCAACAAGTAAGCCA | 55 | 55 | 355 | transcriptional factor B3 family protein, *Arabidopsis* | | | CA286043 | UGSuM362 | (TA)8 | 5'UTRs | GATAGCAAGAAAGCACATCC | CAGCAGAAACAGGAGCAG | 55 | 55 | 242 | gamma-thionin, *Phaseolus vulgaris* | | | AJ231134 | UGSuM363 | (ACT)5 | 5'UTRs | GTAGCACTAACAATAGCAGTTCAT | TTTGTGTAGATGTTTAATTCGTTT | 54 | 55 | 365 | cinnamoyl-CoA reductase, *Saccharum* | | | AJ969049 | UGSuM364 | (CAG)5 | CDS | AAACGATCAGATCTAGCACAAT | CTGCTGTCTTAGGTACAGTCTTC | 55 | 55 | 299 | glycosyltransferase, *Saccharum* | | | AJ969055 | UGSuM365 | (CAC)5 | CDS | TATCTGATCGGTAGCAAATAGC | GTGGTTAAGAAGAGACTAAGTTCG | 56 | 55 | 370 | glycosyltransferase, *Saccharum* | | | AM493723 | UGSuM366 | (GTC)5 | CDS | ACTTAGTCTCTTCTTAACCACTGC | AAGTAATTATCTATAGTGCCACCC | 55 | 55 | 210 | ethylene responsive transcription factor, *Saccharum* | | | AM493723 | UGSuM367 | (TGC)5 | 3'UTRs | ATCTATCGGTCTTCTGGAGATT | CACTTCCTCCTTATTATACCACTT | 56 | 55 | 283 | ethylene responsive transcription factor, *Saccharum* | | | AY596567 | UGSuM368 | (GCC)5 | 3'UTRs | GAGAAGACTGAGAGTTTGACTGTA | CTTCTTCATCCTCTCTAACTTGAT | 54 | 55 | 157 | glycine-rich protein, *Oryza* | | | AY596589 | UGSuM369 | (GGT)5 | 3'UTRs | ATAGAGATAGAGGGAGTATGCTTG | AGTCTCATTCTCATCAGAAACC | 55 | 55 | 317 | protease-associated (PA) domain, *Pisum* | | | AY596612 | UGSuM370 | (TAT)5 | CDS | ACGAGTTCAGGGCGCTGATAGAG | ATCACGACGTCATAGTCCGTAAC | 66 | 60 | 166 | transcriptional co-regulator family protein, *Arabidopsis* | | | AY596613 | UGSuM371 | (TGC)5 | CDS | GCCGAAGCCTCTCCTCTCCTCC | GTCATCAATGACAGAGATGTAGAC | 69 | 55 | 116 | BRI1-KD interacting protein, *Oryza* | | | BQ534085 | UGSuM372 | (TTA)5 | 5'UTRs | GCGTCTCTGCTCTGCACTCTGC | ATTAACATATTCATAGCCCAATTT | 67 | 55 | 391 | RR/LPXTG motif internalin family protein, *Arabidopsis* | | | BQ536356 | UGSuM373 | (GTC)5 | CDS | TAGCAATCTACTCCCTACGTCTAC | GTTGACGTTGATCAGCCCGTTG | 56 | 67 | 378 | zinc finger (AN1-like) family protein, *Arabidopsis* | | | BQ537410 | UGSuM374 | (GAC)5 | CDS | GTTTACATCCACCTCCGCCACC | GTACTCCACATTGATCTGTTCGT | 61 | 55 | 320 | expressed protein, Oryza | | | BU103444 | UGSuM375 | (GAA)5 | CDS | AAGAACATCATGTCGGTGCAGT | GTAAGCTCTTGGTAACATTCTTG | 53 | 56 | 273 | expressed protein, Oryza | | | CA064945 | UGSuM376 | (GCT)5 | 5'UTRs | TCATGTTCTGGAAATAGTTAGTTG | GATCGAGATGCTGAACAAAGAG | 55 | 59 | 327 | hypothetical protein, *Oryza* | | | CA064962 | UGSuM377 | (CCG)5 | CDS | GCGCTCACATCACCTCCTAC | GACGATCTTGAAGAACCTGG | 55 | 54 | 376 | Heavy metal transport/detoxification protein, *Arabidopsis* | | | CA065697 | UGSuM378 | (GGC)5 | 5'UTRs | GACTATATGTACACTGCTGCTGTT | TTATTACAGTTGTTGAGGTGAGTT | 54 | 54 | 159 | expressed protein, *Oryza* | | | CA065736 | UGSuM379 | (CTT)5 | CDS | GTCTATCTTCAAGGAAACCAAA | GGAGAAGTACAATGAGCAAGAT | 65 | 56 | 268 | NAP57, *Arabidopsis* | | | CA066748 | UGSuM380 | (GCT)5 | CDS | GTTCCAAGTTACAGACCAGAAG | TAAGCATAGATAAGAAAGATGTCG | 55 | 55 | 216 | unknown protein, *Arabidopsis* | | | CA067775 | UGSuM381 | (GCG)5 | CDS | ACTAGTTTGCTGTTGCTGACTG | TCTTGAGATATAAACATGGGAGTA | 56 | 55 | 141 | basic helix-loop-helix (bHLH) family protein, *Arabidopsis* | | | CA070210 | UGSuM382 | (TTC)5 | CDS | ACAGATCACAATCAACCAAACTA | AATGTCGAAATGGAACATTAAC | 56 | 55 | 141 | U1 snRNP component, *Arabidopsis* | | | CA071318 | UGSuM383 | (AGA)5 | CDS | TCGAATTCAAAGTTAAATGTCTT | GATTCTGCTCGGATAGAATACG | 55 | 58 | 153 | S25 ribosomal protein, *Medicago* | | | CA073024 | UGSuM384 | (CCG)5 | 5'UTRs | GTTTGTGAGTAGTTCAGTATTTGC | TGTTAGGAGTCTGACTATTGTTCT | 55 | 54 | 355 | transcription factor, *Oryza* | | | CA073274 | UGSuM385 | (CCG)5 | CDS | GTGAAGAAGGAGGAGGTGACGGT | CATCAGAAGATCAGCAGGCAGG | 65 | 64 | 334 | hypothetical protein, *Oryza* | | | CA073445 | UGSuM386 | (GGA)5 | CDS | GGTTTCGAATTACCGGCCCGAC | GAGCCTCTGTCTAGGCTTTAGG | 69 | 58 | 156 | transcription factor [Oryza sativa | | | CA073816 | UGSuM387 | (CGC)5 | 5'UTRs | GGTTTCGAATTACCGGCCCGAC | AAGGCCAGTCACCACTCCACTA | 69 | 63 | 254 | GATA transcription factor 3, *Arabidopsis* | | | CA074644 | UGSuM388 | (CGG)5 | CDS | ATAAGCTCTTTGTAGATAGGAAGC | GAACATCGACATGTACAGTATTTA | 54 | 53 | 300 | leucine zipper protein, *Oryza* | | | CA074905 | UGSuM389 | (CGC)5 | CDS | AAGAACAAGAGATACAACAACAGA | CAGTGTGTTTCTAACACTGAATC | 55 | 54 | 272 | UV-damaged DNA binding protein 2, *Oryza* | | | CA075589 | UGSuM390 | (GCG)5 | CDS | CTAATCGACAGGTCTGAACTTG | CATATTTATATATCACAAGCGACC | 56 | 54 | 389 | hypothetical protein, *Oryza* | | | CA075705 | UGSuM391 | (CGC)5 | CDS | TATCTGATCGGTAGCAAATAGC | GTGGTTAAGAAGAGACTAAGTTCG | 56 | 55 | 370 | inositol polyphosphate, *Arabidopsis* | | | CA076258 | UGSuM392 | (GAG)5 | CDS | CTTCTTTCTGCTTCCAGTTATTAT | CTACAAGGCCTTTGATGAGGTT | 55 | 59 | 324 | zinc finger (DHHC type) family protein, *Arabidopsis* | | | CA078259 | UGSuM393 | (GCG)5 | CDS | CTCCGGGATTAAAGAATCGATGG | CCTCTTCTTAAATTTGGTACTTCT | 64 | 54 | 301 | Auxin-responsive protein IAA11, *Arabidopsis* | | | CA078307 | UGSuM394 | (CCG)5 | 5'UTRs | GACTTGAGAGGAGGAGGAGGAG | CCTCTTCTTAAATTTGGTACTTCT | 60 | 54 | 225 | uracil phosphoribosyl transferase, *Arabidopsis* | | | CA078667 | UGSuM395 | (CCG)5 | CDS | CTATTACAAAGAGTACTCGAGGGT | CATTGCTAGAACTGGTACTCATAG | 55 | 55 | 300 | expressed protein, *Oryza* | | | CA079497 | UGSuM396 | (CCG)5 | 5'UTRs | AATGCTGTCTCTATGAAGTACAAA | GTGTAAGCCTTCTTTCTGAGAC | 55 | 55 | 399 | tubulin folding cofactor A, *Hordeum vulgare* | | | CA080060 | UGSuM397 | (CCA)5 | 5'UTRs | GAGAGCAAGAAGCCGAGGAGGG | TGGACTAGTGTTTAATCCATCTC | 67 | 55 | 195 | auxin-independent growth promoter, *Arabidopsis* | | | CA081110 | UGSuM398 | (GAG)5 | 5'UTRs | CTTGTTGGATAGTACAACCTGTAG | TCTTTGCCTTTCTACTAATGTATG | 54 | 54 | 340 | cellulose synthase-2, *Zea mays* | | | CA083758 | UGSuM399 | (TCG)5 | CDS | GGACAACAAGAAGACCCGCATC | TTGACCATCTCCTACTTTCATT | 65 | 55 | 305 | expressed protein, *Oryza* | | | CA084510 | UGSuM400 | (GGC)5 | CDS | AAGTCTAAGAAGAACAAGAAGGG | ATGTCAAGATCCGAGTTTCTGT | 55 | 57 | 288 | glycosyltransferase, *Sorghum* | | | CA084588 | UGSuM401 | (GAA)5 | CDS | GCGCTCACATCACCTCCTACGC | GGACGATCTTGAAGAACCTGGC | 68 | 64 | 399 | RNA recognition motif (RRM)- protein, *Arabidopsis* | | | CA084621 | UGSuM402 | (GCG)5 | CDS | GGTGAGTACAGGGAAGGAAATC | GTCTCAGTAAGAGAAGGATCAAGT | 58 | 55 | 261 | expressed protein, *Oryza sativa* | | | CA084787 | UGSuM403 | (ACG)5 | CDS | TATACAAGAATGAAAGGTGAGAGA | AAGCATACTCCCTCTATCTCTATG | 55 | 55 | 217 | Helix-loop-helix DNA-binding domain, *Oryza* | | | CA085208 | UGSuM404 | (CGC)5 | CDS | AAGATTACAGAACCAGAGGATTAC | GACTACTCCATGGATTTCTCCC | 54 | 58 | 367 | hypothetical protein, *Oryza* | | | CA086803 | UGSuM405 | (AAG)5 | CDS | ATCGATCATTTCCTCTTCTTAGTA | AATTTGATTCATAGGATTGTGAG | 55 | 55 | 383 | translation initiation factor eIF-2 beta chain, *Oryza* | | | CA086859 | UGSuM406 | (CTC)5 | CDS | GACCATCAGAGACAGCGTGGAG | GTATACTGTGAGGAGGCTGAAG | 64 | 55 | 253 | CLPP2 (Clp protease proteolytic subunit 2), *Pisum* | | | CA087032 | UGSuM407 | (CGC)5 | CDS | GATCTTGCTTCCGTTCTACGTT | TGAGGTAGAACATATTAGCTTGTG | 59 | 55 | 352 | RabGAP/TBC, *Medicago* | | | CA088393 | UGSuM408 | (CCG)5 | 5'UTRs | CTACTCACTCACAAAGTCACAAGT | CTACTACTACTACCAAGTACGGCG | 59 | 64 | 273 | oxidoreductase, Fe oxygenase family protein, *Arabidopsis* | | | CA089088 | UGSuM409 | (CTG)5 | CDS | ATCCACACTACCTGCCTTTCTC | GTGTAGTACCAGTCAGGGTCATA | 69 | 55 | 331 | 3-dehydroquinate synthase, *Arabidopsis* | | | CA089391 | UGSuM410 | (AAG)5 | CDS | GTTTCCTCTCCCACCACCAACAC | CATCAACCTTTGCGTAATAATC | 55 | 55 | 374 | hypothetical protein, *Oryza* | | | CA089404 | UGSuM411 | (CCT)5 | CDS | GTTAGTGACTGTGTCATCAAGGT | TGCAGGAAGTTAAGAATAATAACA | 54 | 56 | 330 | diphosphonucleotide phosphatase1, *Zea mays* | | | CA090918 | UGSuM412 | (GAG)5 | CDS | TGTAGGTCCTAGTAGATCTAGGGT | ATCTTGGACGAGATGAAGCAAG | 67 | 55 | 391 | leucine-rich nuclear phosphoprotein, *Oryza* | | | CA093042 | UGSuM413 | (GAG)5 | CDS | GTTCCTCCTCGAAACCCTATGAG | GAACGTCCGGTAGTAGTGATCC | 55 | 56 | 365 | Protein kinase, *Medicago* | | | CA095367 | UGSuM414 | (AGA)5 | CDS | GTACGCTAGCAATCTACTCCCTAC | GAAGCCGAACAAGAGGAGGTTC | 56 | 67 | 378 | hydrogenase large subunit domain protein, *Arabidopsis* | | | CA098698 | UGSuM415 | (CCG)5 | 5'UTRs | CAATCTGCGGAACCTCTTCTAC | CATAAATATAATTCCAGCAATCAA | 55 | 55 | 176 | acyl carrier protein, *Zea mays* | | | CA098879 | UGSuM416 | (TTC)5 | CDS | GAATGAATCTACACAGGTGAATAA | GTAGCAAGATCATTGTTTCCTT | 60 | 55 | 373 | globulin-like protein, *Oryza* | | | CA100190 | UGSuM417 | (GCA)5 | CDS | CTCAGTCCCAAACGATGCTTAG | GTTGTACTCCACATTGATCTGTT | 67 | 55 | 282 | ATHB-2 (leucine zipper protein HAT4), *Arabidopsis* | | | CA102808 | UGSuM418 | (GGC)5 | CDS | GTACGACACGGTGAAGAACCTT | CTCATCACTATAGTCCACAAGAAC | 65 | 56 | 320 | cyclophilin, *Arabidopsis* | | | CA103142 | UGSuM419 | (GCA)5 | CDS | CAAGTTCACAAGTTCAATACATAA | AAGAAGATTTGCTCAAGTTCATAC | 58 | 56 | 333 | senescence-associated protein, *Arabidopsis* | | | CA103389 | UGSuM420 | (GGT)5 | CDS | CAATCGATGTTTACATCACAAC | GATAGGGAGTAAATCTGGTAGAAA | 69 | 55 | 380 | phospholipase, *Oryza* | | | CA103405 | UGSuM421 | (CGG)5 | CDS | AGCAACTGCAATGGCTGCTCCAC | ATCAGTTGATCTTGAAGCAGTT | 56 | 58 | 285 | hypothetical protein, *Oryza* | | | CA103498 | UGSuM422 | (CGC)5 | CDS | AATTCATGTTTAGATCCTCTCAAT | AATCAGTCTTGTGTAAGTCTCTTG | 55 | 60 | 399 | protein disulfide isomerase, *Zea mays* | | | CA103536 | UGSuM423 | (CGG)5 | CDS | GAGAATTGTCTTGTATATTGGTCC | TTGAGATCTCCAAGGGTACGAG | 61 | 62 | 390 | Protease inhibitor/seed storage/LTP family protein, *Pisum* | | | CA103832 | UGSuM424 | (CCG)5 | CDS | GTTAATCTACCTTTATTACGCAGC | CTCAAGGTCGTCTTCTGCCAGT | 54 | 54 | 159 | fiber protein Fb2, *Gossypium* | | | CA103845 | UGSuM425 | (CGA)5 | CDS | ACTACTTCTAATACAACGGAGAGG | ATGAAGATCATCTACTGCAACC | 55 | 59 | 327 | cellulose synthase catalytic subunit 10, *Zea mays* | | | CA103895 | UGSuM426 | (GCG)5 | CDS | GATAGTAATAGGTGTAGCAGAGGC | ACCATCCTGTTGTTCCTCTCAC | 54 | 63 | 296 | zinc finger protein, *Oryza* | | | CA103926 | UGSuM427 | (CTC)5 | CDS | TACACCTGTAAGTACAACGATCC | ATGTTCTTGTTCGTCATGAAGTT | 54 | 54 | 369 | hypothetical protein, *Oryza* | | | CA104231 | UGSuM428 | (CGC)5 | CDS | GAACATGTACGACAACAACTTC | CCCTACGAGTTTATTCTTCAGTA | 62 | 55 | 158 | Novel plant SNARE 11 (AtNPSN11), *Arabidopsis* | | | CA104607 | UGSuM429 | (GCA)5 | CDS | CGCATATATATAGCTAGAGCGTAA | ATATGCCCGTAGTGCGCTGAGT | 54 | 55 | 371 | DNA polymerase delta subunit 4 family, *Arabidopsis* | | | CA104621 | UGSuM430 | (CTT)5 | 3'UTRs | GTACTACTACTGGTACCTCACCAA | TCAAGTTGATAGATCACTCCATAG | 60 | 56 | 385 | HOX family protein, *Arabidopsis* | | | CA105161 | UGSuM431 | (GCA)5 | 3'UTRs | CTTGCATGAGCATGAGACAGGA | GGTCAAGACATGTGTAGTTGTAAT | 63 | 54 | 134 | lipoprotein, *Arabidopsis* | | | CA105904 | UGSuM432 | (TTA)5 | 3'UTRs | ATTATCTACATTCAGACACGTCAC | ATCTTTGTTAGCAATCCATTAAG | 59 | 65 | 361 | (*ATAURORA1*); histone serine kinase, *Arabidopsis* | | | CA106112 | UGSuM433 | (CGG)5 | 3'UTRs | GTGGGTCTTTCCTTGCAATGCC | GTACTCGTCTAGGTGCTTAGTCAT | 55 | 56 | 284 | unknown protein, *Arabidopsis* | | | CA106322 | UGSuM434 | (CAC)5 | 3'UTRs | GAAGAAGAAGAAGAAGAAGAAGAA | ACTCGTCCTACAACCACGACTAC | 55 | 55 | 295 | leucine-rich repeat family protein, *Arabidopsis* | | | CA106555 | UGSuM435 | (CGG)5 | CDS | AGATCTTAACTCTCACCTGAAATC | TAGAAGATTTAACAGAGCATAACG | 63 | 54 | 183 | homeodomain-leucine zipper transcription factor, *Oryza* | | | CA106603 | UGSuM436 | (GGC)5 | CDS | ATTAGGACACGAGAACTAGCATA | AGGAAATTCGTAGAAGATGTTATC | 56 | 54 | 342 | splicing factor Prp18 family protein, *Arabidopsis* | | | CA107157 | UGSuM437 | (GCG)5 | CDS | GCTGCTATATACCAAACAAGAAAT | GTACTTCAATGGGTGATAAGTGT | 55 | 54 | 393 | serine-rich protein-related, *Arabidopsis* | | | CA110497 | UGSuM438 | (GCG)5 | CDS | GAAAGTAGAATTTGGGAACTAAAC | TTCTCAAAGAGAACTCAACATAAA | 58 | 55 | 321 | unknown protein, *Arabidopsis* | | | CA110505 | UGSuM439 | (CAG)5 | 5'UTRs | AGAAAGGAAAGGAAAGCAAGAG | CATCATAGATGACAGATCGTAGAG | 57 | 54 | 165 | peroxidase ATP22a, *Arabidopsis* | | | CA111000 | UGSuM440 | (GCG)5 | 5'UTRs | AGTTGAAATTAAGAGAACCATACC | TAAAGCCACTATCATATGCTGAC | 55 | 56 | 374 | calmodulin-binding protein, *Beta vulgaris* | | | CA111332 | UGSuM441 | (CCG)5 | CDS | ACATCCACCCAGATCTCGCC | GTGACGGAGATAGACACCAT | 54 | 61 | 215 | transcription factor, *Arabidopsis* | | | CA112141 | UGSuM442 | (AGA)5 | CDS | AGAAACCGATACAACAAAGACT | GAGAGTGAGCGTATTTCTTCTT | 55 | 54 | 159 | farnesylated protein, *Brassica* | | | CA113255 | UGSuM443 | (CGG)5 | CDS | CTTAGCCCATCTAACCATACAG | AATGGAATAAGTTGTTGCTGTT | 55 | 55 | 380 | hypothetical protein, *Oryza* | | | CA113863 | UGSuM444 | (GCT)5 | CDS | ACCTCTACGACCTCTCCGAC | GGCCTTGATAGTGTACAGATTT | 55 | 55 | 380 | response regulator, *Zea mays* | | | CA114681 | UGSuM445 | (TTC)5 | CDS | CATCTAACCATACAGACCATCA | AATGGAATAAGTTGTTGCTGTT | 67 | 56 | 102 | unknown protein, *Arabidopsis* | | | CA117139 | UGSuM446 | (GAC)5 | CDS | AAACATTTATAACTCATTGGTGTG | AAGCATCTTCTTGTACCTATCAAT | 54 | 59 | 226 | NAC protein, *Arabidopsis* | | | CA117618 | UGSuM447 | (GAG)5 | CDS | ATATGTACCCTTGATGAATCTCTC | GCAACGTCTAAATATAATTGCTAA | 57 | 55 | 250 | unknown protein, *Oryza* | | | CA117674 | UGSuM448 | (AGG)5 | CDS | TCCCTACTTCTATGAATATCCTTC | TTGACAAATTGCTTGATGTAGT | 63 | 62 | 399 | hypothetical protein, *Oryza* | | | CA117696 | UGSuM449 | (GCC)5 | CDS | AGTTATTAAGGCCACCCAGCCTAA | GTTGAGGTTGATGCCAAGGATT | 54 | 55 | 333 | plastocyanin-like domain-containing protein, *Arabidopsis* | | | CA118800 | UGSuM450 | (TCC)5 | CDS | CTACCAATGATACAACAACATTCT | ATTCTTTGAGCTTAGTTGTCATAA | 60 | 54 | 134 | ATX1; metal ion binding protein, *Arabidopsis* | | | CA119252 | UGSuM451 | (GCG)5 | CDS | GATCCGATTACAGAACAGAATTAC | GTGTTGCTAGAATAAAGTTGGTG | 54 | 55 | 223 | hypothetical protein, *Vitis vinifera* | | | CA119580 | UGSuM452 | (CGC)5 | CDS | GTATATGTTCGTAGTTTGTATGCC | ATTCACTTAGTCACACTCTCACAC | 54 | 55 | 310 | epsilon1-COP, *Glycine max* | | | CA119653 | UGSuM453 | (ACC)5 | CDS | TACTGGAGTAGCTTAGACTGAGTG | TATATGCAAGTCCATTAGAGGTAA | 58 | 54 | 252 | hypothetical protein, *Oryza* | | | CA120255 | UGSuM454 | (CCT)5 | CDS | TGATCTCTCTCTCTTTCTCTCTCT | ACCTTAGAATCATTCAATCCTTAC | 55 | 56 | 318 | Acyltransferase 3, *Arabidopsis* | | | CA120375 | UGSuM455 | (GCC)5 | 3'UTRs | ACTCTGAACAAGAGAAGAAAGAAC | GTCCCTCCACTTAGTAATGTTT | 54 | 55 | 292 | 40S ribosomal protein S17 (RPS17D), *Arabidopsis* | | | CA120400 | UGSuM456 | (CGC)5 | 3'UTRs | TACTTCAATCTAACACCTTTACCA | TGTATATTAGAGATTTAAGCCACG | 54 | 55 | 203 | expressed protein, *Oryza* | | | CA120409 | UGSuM457 | (CGG)5 | 3'UTRs | GCCCGTAAGCTGTTCGCTCTTT | GACACCTGGAGGTTGTACCAAA | 62 | 54 | 373 | *LigA*, *Arabidopsis* | | | CA120964 | UGSuM458 | (AAG)5 | 3'UTRs | GAGCAAGAACCAAGAACGA | ATGGAGAGGATGGGCTGG | 56 | 61 | 307 | BAP28-related protein, *Arabidopsis* | | | CA121052 | UGSuM459 | (GCG)5 | 3'UTRs | CGACGGCATCCAAGCGAG | GCCTGTTCCTCTTTCACC | 66 | 55 | 375 | laccase 1, *Zea mays* | | | CA121660 | UGSuM460 | (CCG)5 | 3'UTRs | CCGAGTGATGATGTGATGT | GGGACAACTAATGTAACTGATT | 55 | 52 | 293 | cytochrome C oxidase assembly protein, *Oryza* | | | CA122167 | UGSuM461 | (AAG)5 | 5'UTRs | GTGGTGAGGAAGATAGTTGG | CTACAAGGCGACAGACACTC | 54 | 56 | 248 | NADP-specific glutatamate dehydrogenase, *Arabidopsis* | | | CA122330 | UGSuM462 | (CCG)5 | 5'UTRs | AGCAGTGATTGATGGATAGA | ATGGAGAGGAGGAGATGAAG | 53 | 55 | 268 | zinc finger (Ran-binding) family protein, *Oryza* | | | CA123065 | UGSuM463 | (CGG)5 | 5'UTRs | CGGACAAGAAAGAACAAGAG | TTGAACTGATGACTGGATGA | 55 | 54 | 317 | calcium-binding protein, *Oryza* | | | CA123971^B^ | UGSuM464 | (ACA)5 | 5'UTRs | GGCTACTTCAGACACGCA | TCTACGCATCAACCTCTCA | 55 | 55 | 343 | SNF2- domain-containing protein, *Oryza* | | | CA125310^B^ | UGSuM465 | (GCA)5 | CDS | GCTAACCAACATCAGCAGT | AGGAGATTGACGAAGAAGAAG | 53 | 55 | 342 | transducin family protein, *Arabidopsis* | | | CA125811 | UGSuM466 | (GCT)5 | CDS | TTTATTGAGGTTGAGGGTG | AGAGGAGAGACCATTCCATT | 53 | 55 | 150 | maize protease inhibitor, *Zea mays* | | | CA125929 | UGSuM467 | (CCG)5 | CDS | CTCGGTTGATGATGCTATG | TACAGGAGCGAAATAAAGAAG | 55 | 54 | 393 | hypothetical protein, *Oryza* | | | CA125935 | UGSuM468 | (TCC)5 | CDS | AGCCCTACTGATTGTGCC | TCTCCCACTTCCTTCGTT | 56 | 55 | 382 | Ubiquitin-conjugating enzyme, *Medicago* | | | CA125968 | UGSuM469 | (GAG)5 | CDS | GCTTATTTCCCTGTTCGTC | GCACACACCTCCAACTTC | 54 | 54 | 159 | hypothetical protein, *Oryza* | | | CA126513 | UGSuM470 | (CCG)5 | CDS | AAGAGCACAAACGCAAGTAG | TGAGCCACTGTAGGATGATT | 55 | 55 | 344 | cytosolic factor-like protein, *Oryza* | | | CA127133 | UGSuM471 | (CCA)5 | CDS | GGAGCAGATTCATTAGTTGAG | ATCCACCAGAACAGAAACAG | 54 | 55 | 336 | MYB transcription factor, *Oryza* | | | CA127456 | UGSuM472 | (CTG)5 | CDS | CTGTTGCTGGAGTTGCTG | GATGGTGAGGTTGACGGG | 56 | 59 | 176 | RING zinc finger protein, *Oryza* | | | CA128349 | UGSuM473 | (CTT)5 | CDS | ACTTTGCTTTGATTGACTGG | AGAGGATGGAGATGGTGAG | 55 | 54 | 367 | hypothetical protein, *Oryza* | | | CA128692 | UGSuM474 | (TGC)5 | CDS | AGGCGACGACCACGACAC | CCTTCATTTATCCTGAGCC | 63 | 54 | 399 | hypothetical protein, *Oryza* | | | CA128815 | UGSuM475 | (CGG)5 | CDS | TCTTTCTTATCCACCATCAAC | GGGCTCTTCTACCCAGAC | 54 | 54 | 389 | pirin, *Arabidopsis* | | | CA129032 | UGSuM476 | (GAC)5 | CDS | CCTCTCATTGCGGCTCCT | GCTACCAGACAGTAAGCATTT | 61 | 53 | 254 | hypothetical protein, *Oryza* | | | CA130005 | UGSuM477 | (GGC)5 | CDS | TATTGGTCCTTTCTCCCA | AGAGTGGTGGTGGTTGTTT | 53 | 55 | 188 | hypothetical protein, *Vitis vinifera* | | | CA130339 | UGSuM478 | (GCG)5 | CDS | GCTATTGGCTACAAAGGGT | ACTGGAGGTCATCTTCTTCTT | 54 | 54 | 181 | small nuclear ribonucleoprotein F, *Arabidopsis* | | | CA130542 | UGSuM479 | (GCA)5 | CDS | AAGGACGAGGTGATGAAG | CCGAGTGTCTTGGTGGAG | 53 | 57 | 395 | MATE efflux family protein, *Arabidopsis* | | | CA130708 | UGSuM480 | (TGC)5 | CDS | TGTTACTGCTTCTTGTGCTG | TTCAACCTCATCGCCTTAC | 55 | 56 | 242 | xylose isomerase, *Hordeum vulgare* | | | CA130832 | UGSuM481 | (ATA)5 | CDS | TCAGCCTCATCCTCAACTAC | CAGTCAAACCATCATTCAAAC | 55 | 55 | 175 | DEAD/DEAH box helicase, *Arabidopsis* | | | CA131087 | UGSuM482 | (GGC)5 | CDS | GTGGCGTGCTCTGCTCTC | GTAGATTTGGAAGATGGGCT | 60 | 55 | 295 | GAMMAGLUTAMYL HYDROLASE3, *Arabidopsis* | | | CA131266 | UGSuM483 | (GCG)5 | CDS | TATCACACCAAGCACAGAAGT | CCATTTCGTCAGTTCCTTATT | 55 | 55 | 361 | hypothetical protein, *Oryza* | | | CA131943 | UGSuM484 | (GAT)5 | 5'UTRs | CTTCCTCCGCTTCAACCT | CCCAACAACACATACATCTC | 57 | 53 | 198 | ELF7 (EARLY FLOWERING 7), *Arabidopsis* | | | CA133394 | UGSuM485 | (GTG)5 | 5'UTRs | CAAACTCAGGCTACACACATT | GCTTTATGGAGGAAGTGAAAC | 55 | 55 | 266 | glycine-rich protein, *Arabidopsis* | | | CA133509 | UGSuM486 | (CGG)5 | 5'UTRs | AGAAGAGCAAGTTCCCGT | TAGCAGGAGCAATCCAAA | 54 | 55 | 277 | sin3 associated polypeptide p18, *Arabidopsis* | | | CA133893 | UGSuM487 | (CAG)5 | 5'UTRs | GCTGTTGTTTATTTGTTTGCT | CTCATCTTGGATTCTTGGTT | 54 | 54 | 399 | expressed protein, *Oryza* | | | CA134134 | UGSuM488 | (ATC)5 | 5'UTRs | GTCCAGGCATAACATTCATT | TTCGCTCGGTCTCTCTATT | 54 | 55 | 211 | unknown protein, *Arabidopsis* | | | CA134220 | UGSuM489 | (CCG)5 | 3'UTRs | ACTCCCAGTCCCAACAAA | GCAAAGTAAGCAACCCTAATG | 56 | 56 | 307 | pfkB-type carbohydrate kinase family protein, *Oryza* | | | CA134332 | UGSuM490 | (GCG)5 | 3'UTRs | CTCGTTTCATAGCAGACCTT | GCAACTGGAGGAACTGATG | 54 | 56 | 224 | sorbitol dehydrogenase-like protein, *Arabidopsis* | | | CA134431 | UGSuM491 | (GGC)5 | 3'UTRs | CGCATCAGTTCCTCCAGT | AACATCACCGTCAACCAG | 57 | 55 | 289 | ACR6 (ACT Domain Repeat 6), *Arabidopsis* | | | CA134576 | UGSuM492 | (CGG)5 | 3'UTRs | CCGTCTCTTCTTTCTGCTT | CACTTGCCGCTGGATGGA | 54 | 64 | 217 | Ssu72 RNA polymerase II CTD phosphatase, *Arabidopsis* | | | CA134597 | UGSuM493 | (GAG)5 | 3'UTRs | GGCACCTGAAGAACAATGA | AATCCAGCCCTACTACTGAAC | 57 | 55 | 336 | tobamovirus multiplication 3, *Nicotiana* | | | CA134793 | UGSuM494 | (CGC)5 | 3'UTRs | TTCTTCTCTCCACTTGCTAAA | GTATGCTGCTCTGTCATTCC | 54 | 55 | 383 | expressed protein, *Arabidopsis* | | | CA134873 | UGSuM495 | (CCG)5 | 3'UTRs | GAAGGAAGACTACCCTAACCAC | CCTCTTCTTAATTTCGTCTGTG | 59 | 60 | 173 | RNA recognition motif family protein, *Oryza* | | | CA136213 | UGSuM496 | (GCG)5 | 3'UTRs | GTGGACGAGAAGTGGAAGT | GAGAAACTGACTGCGAGG | 55 | 54 | 380 | ATP-dependent RNA helicase, *Arabidopsis* | | | CA136552 | UGSuM497 | (GTC)5 | 3'UTRs | AAGCAAGAGAAGGATTAGGAC | GGATGAAAGAGGAGTGTATGG | 54 | 56 | 316 | ring-H2 zinc finger protein, *Zea mays* | | | CA137166 | UGSuM498 | (CGC)5 | 3'UTRs | CAGAACTCCATCATTACACAAC | TGGCAGGTTTCGCTTTCC | 54 | 62 | 394 | bZIP transcription factor bZIP43, *Glycine max* | | | CA137576 | UGSuM499 | (GAC)5 | CDS | CCAACTATCCTCATCCAAAG | AAGTCCCTCTCAATCCTGAA | 54 | 56 | 266 | ribosomal protein S6 family protein, *Arabidopsis* | | | CA137800 | UGSuM500 | (CGG)5 | CDS | ATCGTCTCTGGTTGTTGGT | ATCCTCCATTTCCACCTC | 55 | 54 | 316 | unknown protein, *Arabidopsis* | | | CA137888 | UGSuM501 | (CAC)5 | CDS | AAGCACCACCACCACCAC | AAGTAGACCTTCCAGTTGCC | 60 | 55 | 304 | cytochrome c oxidase family protein, *Arabidopsis* | | | CA138041 | UGSuM502 | (GCT)5 | CDS | TGAGAGGAGGAGGAGGAG | TGAGAAATGAATCTTGTAGGG | 55 | 54 | 400 | lipase, *Hordeum vulgare* | | | CA138365 | UGSuM503 | (GCG)5 | CDS | TGCTTACACACAGATGGAC | GCCGCACCACTAACTACTC | 52 | 55 | 321 | Nodulin, *Oryza* | | | CA138973 | UGSuM504 | (CTG)5 | CDS | AGGTTGGCTTGGTGTCTT | CGACTCACAATCACTGGCT | 55 | 54 | 369 | hypothetical protein, *Oryza* | | | CA138994 | UGSuM505 | (AGG)5 | CDS | CTCTCTCCTCTCCACTACTCAC | GTGATGACGCACGTGTAACT | 54 | 54 | 264 | Peptidase S26A, signal peptidase I, *Medicago* | | | CA139172 | UGSuM506 | (CCA)5 | CDS | AAAGAGAACCGAGAGCAAA | ATTCATCACCTTCAGTGGTC | 55 | 54 | 295 | tRNA-binding arm, *Medicago* | | | CA139177 | UGSuM507 | (CGC)5 | CDS | AGTTTGTTCTCTCCCTTTGAC | TACTACTTCCACCACGATGA | 55 | 54 | 241 | unknown protein, *Arabidopsis* | | | CA139213 | UGSuM508 | (CTC)5 | CDS | GAGCAAGAAGCCGAGGAG | TACCAAGCCAGACACGAA | 58 | 56 | 332 | cytochrome P450, *Oryza* | | | CA141204 | UGSuM509 | (CGC)5 | 5'UTRs | CTCTCGTTCGCTCTCGCCT | CTAAGCCCACATCCTGGT | 63 | 55 | 193 | salt tolerance protein 1, *Beta vulgaris* | | | CA141245 | UGSuM510 | (CCG)5 | 5'UTRs | TATTCAGTCATTCGTTTCGTT | CCTTTGTGTATGTGTGTGTGT | 54 | 54 | 160 | methionyl-tRNA synthetase, *Arabidopsis* | | | CA142909 | UGSuM511 | (CGC)5 | 5'UTRs | GCGAGCGACACCTCTATC | ACATCTCTTTCGTCTGCCT | 56 | 54 | 195 | Rapid Alkalinization Factor, *Medicago* | | | CA145451 | UGSuM512 | (CAC)5 | CDS | TATTCAAGAATGGAGGAGAAAG | GAAGAAGGGTAGAGTGGCAG | 55 | 57 | 338 | geranylgeranyl diphosphate synthase, *Arabidopsis* | | | CA145715 | UGSuM513 | (CTG)5 | CDS | ACTTGGTGTAAATGAATCTTG | CAGGCGATGACGGGCTTT | 51 | 64 | 259 | zinc finger (C3HC4-type RING finger) family, *Oryza* | | | CA145879 | UGSuM514 | (CCG)5 | CDS | GTGGTGGTCGTGGAAGTG | TTCTCCTCCTCCTCTGTCTC | 58 | 56 | 134 | unknown protein, *Oryza* | | | CA146953 | UGSuM515 | (CGC)5 | CDS | AGCAACCAGACGAGGAGT | GGTCCAGAGCAGTTTGTT | 55 | 53 | 135 | Glycosyl transferase, *Medicago* | | | CA146985 | UGSuM516 | (CGG)5 | CDS | TATGGAAGCCAAAGAGAATAG | CATCAAAGGAAGAGAGTGGA | 53 | 55 | 343 | Cyclin-related protein, *Medicago* | | | CA148468 | UGSuM517 | (GGC)5 | CDS | CCCTCTCGCTCGCTACTC | CCAACACCATCTTTCTTAGTG | 58 | 54 | 292 | class III peroxidase 138 precursor, *Oryza* | | | CA148954 | UGSuM518 | (CCG)5 | CDS | AACCCTCTGCTCCTCGCA | GCTTGTGCTTGTTCATCTTAC | 62 | 55 | 363 | metacaspase 3, *Arabidopsis* | | | CA149330 | UGSuM519 | (GGA)5 | CDS | ATTGTTCTAAGGCACACTCT | GAAGCAGGGTCGGTGATG | 51 | 60 | 254 | EREBP transcription factor, *Triticum* | | | CA149596 | UGSuM520 | (CAG)5 | CDS | AGAGGAAAGCAAAGACAAGTAG | ATGAAGTGGAACAGAAGATGA | 54 | 54 | 149 | transcription factor bZIP63, *Glycine max* | | | CA150865 | UGSuM521 | (GGC)5 | CDS | CTCGTCCAAAGACTCCAAG | ACGGGCAAGTTTATTAGGG | 55 | 56 | 379 | expressed protein, *Oryza* | | | CA152440 | UGSuM522 | (TAC)5 | CDS | GAGGGACAACAAGAAGACC | TAGGAACCAACCCAAGAAA | 54 | 55 | 332 | hypothetical protein, *Oryza* | | | CA152466 | UGSuM523 | (GCG)5 | CDS | ATGTCCTCAGTCCTCCTCA | ACCCTCACACCCAAGAAG | 55 | 55 | 302 | expressed protein, *Oryza* | | | CA152821 | UGSuM524 | (GAC)5 | CDS | GGCAACAAACACAAACACT | GGCTCCGTATCTTACTTTCT | 54 | 52 | 250 | hypothetical protein, *Oryza* | | | CA153229 | UGSuM525 | (CGC)5 | CDS | TAACTGGATTCTCCTAAACC | ACACATCGCCTCCTCCTC | 51 | 58 | 187 | Indole-3-acetic acid, *Arabidopsis* | | | CA154198 | UGSuM526 | (GCA)5 | CDS | ATACCCTGCACAAGGTTACTAC | AGAAGGTGCAAGGAATAGAGTA | 51 | 58 | 187 | phosphatidate cytidylyl transferase protein, *Arabidopsis* | | | CA154741 | UGSuM527 | (CTC)5 | CDS | CACTCCTCCGCCTTTACC | ACCCTCCATCTCCTTTCTC | 58 | 55 | 169 | cystatin, *Zea mays* | | | CA155274 | UGSuM528 | (CGA)5 | CDS | AGATGGAGGGTGCGAGAC | TTTCTTTGGTCGTGCCTG | 58 | 58 | 375 | F-box family protein, *Oryza* | | | CA155811 | UGSuM529 | (CCG)5 | CDS | ATACACCAAATCCAAACACAG | GTTGAGAGCTTGTGTTCCTC | 55 | 54 | 333 | unknown protein, *Arabidopsis* | | | CA157186 | UGSuM530 | (GCC)5 | CDS | CACCTCTCTCCTCTTCTTCTC | ACAACGCCATCTCTACCA | 54 | 54 | 384 | ring-H2 zinc finger protein, *Zea mays* | | | CA157270 | UGSuM531 | (GCC)5 | CDS | AAACGACACGAAGCACGA | ATAAATGACGAGCCCAAG | 58 | 53 | 336 | Acetohydroxy acid isomeroreductase, *Medicago* | | | CA157996 | UGSuM532 | (GCG)5 | CDS | ATAATGACTGAACCTCTCCC | CTTCCTGTGCTTCCTGGT | 53 | 56 | 276 | FAD dependent oxidoreductase, *Arabidopsis* | | | CA158158 | UGSuM533 | (AGC)5 | CDS | AGAACTGAGCGAGGAGATT | GCCTGGAACTTGGTCTTG | 54 | 56 | 182 | SYNC1 protein, *Medicago* | | | CA158777 | UGSuM534 | (GCC)5 | CDS | GATGACCCTTTGGATGTAGTT | ACAGAGTTTCCTTGACCCA | 55 | 55 | 379 | cellulose synthase-2, *Zea mays* | | | CA159034 | UGSuM535 | (TCG)5 | CDS | ACAGAGAGGGAGAGAGAAAGA | GGGACATCGTGCTGAGAG | 54 | 57 | 378 | histone H2A, *Arabidopsis* | | | CA159070 | UGSuM536 | (TGG)5 | CDS | GTGAACCAACCCATTTACAAC | GCCAACTATCTGAACATCAAG | 56 | 54 | 353 | unknown protein, *Oryza* | | | CA159542 | UGSuM537 | (GCC)5 | CDS | GCTGGAGGAGGAGATGAA | AACATACACACTGCGACAAG | 56 | 54 | 235 | expressed protein, *Arabidopsis* | | | CA160387 | UGSuM538 | (GCG)5 | 5'UTRs | CTCCCTTGCTTTCCGTGT | ATGGTGTTGTTCGTCTTCTC | 58 | 55 | 161 | Glycoside hydrolase, family 28, *Medicago* | | | CA161712 | UGSuM539 | (GGC)5 | 5'UTRs | GTGTTGGAGAGGTGGTTGT | CTTGAGAGTGGTGGTTGGT | 55 | 55 | 400 | lipase, *Oryza* | | | CA162470 | UGSuM540 | (GCA)5 | 5'UTRs | GATGATGCGTGTGATGGT | GGAAGAAAGAGGAAGGGAG | 56 | 55 | 294 | DNA binding / transcription factor, *Arabidopsis* | | | CA163320 | UGSuM541 | (TCG)5 | 5'UTRs | TCTCTCTCTTTCTCTCTCTCTCA | ACCTTTCACACCATCACAAC | 54 | 55 | 321 | NAK-type protein kinase, *Nicotiana* | | | CA163396 | UGSuM542 | (GCG)5 | 5'UTRs | CAGAACCATCACAAACACAA | TTCAAGGCATCCACTGTC | 55 | 55 | 297 | pectinesterase, *Arabidopsis* | | | CA164139 | UGSuM543 | (GAT)5 | 5'UTRs | GGAGAAGAAGGACGAGGT | AGGGTATCTGGTGGCAGG | 54 | 58 | 310 | ABT1-associated protein, *Medicago* | | | CA164193 | UGSuM544 | (CCG)5 | 5'UTRs | AGATGAGGTGGATAACTTGG | CTTCTTTGTTCCTTCCCTG | 54 | 54 | 370 | unknown protein, *Oryza* | | | CA164396 | UGSuM545 | (CGG)5 | 5'UTRs | GTACACGCTGGTGGAGGAGAAC | AGACAAGAACCTCTCAAGAAGA | 54 | 54 | 323 | Protein phosphatase 2C, *Medicago* | | | CA165112 | UGSuM546 | (CAG)5 | 5'UTRs | TTCCTTGTTCCCTCCATC | AGTTCTTTCTTCTCTTGTTCAG | 55 | 52 | 115 | somatic embryogenesis receptor kinase, *Arabidopsis* | | | CA165846 | UGSuM547 | (GCT)5 | CDS | TTCGCTGGAATGAGGAAG | ACCATCAACAGGTAGCAAGA | 57 | 55 | 371 | hypothetical protein, *Oryza* | | | CA166974 | UGSuM548 | (CCG)5 | CDS | AATGCTCCACCAAACTCTC | ATCGTATCCACCTTCGCC | 55 | 58 | 398 | hypothetical protein, *Oryza* | | | CA167733 | UGSuM549 | (CGC)5 | CDS | GTCTTCTTCTCCGCCTCC | GTTTCTCCCTTCCTTGCC | 57 | 57 | 208 | uroporphyrin-III C-methyltransferase, *Arabidopsis* | | | CA167921 | UGSuM550 | (CGC)5 | CDS | GGAGCCTTTGGTAGGTTAG | CGTGAAGAGAGAATGAAGAAG | 54 | 54 | 304 | bZIP family transcription factor, *Arabidopsis* | | | CA174283 | UGSuM551 | (TGC)5 | CDS | TAGTTGTGGAAGAGATGGAGG | GATTGAAGAGCAGATGAAGTG | 56 | 55 | 191 | serine/threonine-protein kinase NAK, *Arabidopsis* | | | CA175472 | UGSuM552 | (CCG)5 | CDS | CAGGATGAAATCGTTGGA | GATAGACCGTGGAAGTGAAGT | 55 | 55 | 387 | Zinc finger, C3HC4 type family protein, *Oryza* | | | CA177332 | UGSuM553 | (CGC)5 | CDS | CACGGGCTCATCTCCAAC | CCAATAAACGCAAGGAAAC | 60 | 55 | 252 | Pentaxin, *Medicago* | | | CA177925 | UGSuM554 | (CGG)5 | CDS | TTACACATTCAACCAAATCAG | GCTCTTCTTCTCCAGCATC | 53 | 55 | 288 | oxidoreductase, 2OG-Fe(II) oxygenase family, *Oryza* | | | CA182684 | UGSuM555 | (AAG)5 | CDS | TCCATTCCTTGTTATGTGAGA | CAGAGTTATTCCATTGTGGG | 55 | 55 | 229 | fasciclin-like protein FLA17, *Triticum* | | | CA183299 | UGSuM556 | (GGA)5 | CDS | AAAGGAATGTATGGACGCT | AGATGTTTGGAGAAGGTTGA | 54 | 54 | 385 | Ran-binding protein, *Triticum* | | | CA183376 | UGSuM557 | (CGT)5 | CDS | GTCCAACCTCACCTCCTC | GAGAAAGAGCCACGACCT | 55 | 55 | 146 | leucine-rich repeat protein kinase, *Arabidopsis* | | | CA183646 | UGSuM558 | (GGC)5 | CDS | GGTTCTCGTTCTCTCTAACATC | CAAGGCTATGCTGTCTTCA | 54 | 54 | 375 | transcription factor MYB31, *Zea mays* | | | CA183651 | UGSuM559 | (GCG)5 | CDS | TGTAGGAGAGCAGGATAGGA | CTTTCACAACCACCATAATCT | 55 | 54 | 349 | phosphatidic acid phosphatase beta amylase, *Oryza* | | | CA184085 | UGSuM560 | (CGC)5 | CDS | AGCACCAGGGACCAGTTT | GGAGGGAAGTTGTTGACC | 58 | 55 | 358 | mRNA capping enzyme family protein, *Arabidopsis* | | | CA185192 | UGSuM561 | (GTC)5 | CDS | AGAGAGAGGCAGAGGATAAGA | ACTGAAGGAGCCAGGATT | 55 | 54 | 272 | chloroplast nucleoid DNA-binding protein cnd4, *Oryza* | | | CA185225 | UGSuM562 | (TCC)5 | CDS | ACACAGGAAACAGAGGGAG | CAACCTACAAGACGGCAC | 55 | 55 | 348 | hypothetical protein, *Oryza* | | | CA186291 | UGSuM563 | (CCG)5 | CDS | CAAGGAGGGATGGATAGG | CGGGAGAATAGTGTGATGTT | 55 | 55 | 229 | P-glycoprotein 1, *Sorghum bicolor* | | | CA187542 | UGSuM564 | (CGC)5 | CDS | GCTTGGTCAGCAGGATGG | GGTGGTTGAGAGGAAGGA | 60 | 55 | 307 | Expressed protein, *Oryza* | | | CA189542 | UGSuM565 | (ACC)5 | CDS | CAGCGGGTCGTCGTTATG | CCCATTGCTTGAGTGTTC | 61 | 54 | 365 | hypothetical protein, *Oryza* | | | CA189996 | UGSuM566 | (TCG)5 | CDS | TGATACAGCATTGGAAATAG | AGGGTTTAGGGATTGAAGAG | 51 | 54 | 377 | hypothetical protein, *Oryza* | | | CA190789 | UGSuM567 | (GCG)5 | CDS | ACAAACAGTCCAGAAAGCAA | GTGGCGAAGAGAGAGAGG | 55 | 55 | 313 | heme oxygenase 1, *Lycopersicon* | | | CA195244 | UGSuM568 | (GCT)5 | CDS | CTGCTATTTGTCTCGTGTTG | GTGTGCTAAAGATGCTGATGT | 54 | 55 | 180 | hypothetical protein, *Oryza* | | | CA195245 | UGSuM569 | (GGC)5 | CDS | CTCACGCCTCGCAGTCATC | CCTTAGCCAGCATAGCATAGT | 63 | 56 | 298 | ankyrin repeat family protein, *Arabidopsis* | | | CA195254 | UGSuM570 | (GCC)5 | CDS | TAGCCTTCTCTGCCGCTT | AAGATTATTGTGAGGGAGGA | 58 | 53 | 139 | hypothetical protein, *Oryza* | | | CA195823 | UGSuM571 | (AAG)5 | CDS | CTCACCATTACAGGCTTACAC | GACCACCACTACCACGATT | 54 | 55 | 335 | Expressed protein, *Oryza* | | | CA195848 | UGSuM572 | (CTC)5 | CDS | TATTTCCTCCTCTGCCCAC | GATAACCTCAACGCCAAGA | 57 | 56 | 330 | hypothetical protein, *Oryza* | | | CA196032 | UGSuM573 | (CGC)5 | CDS | ACTGGTAGGAGGAGGAGATG | ACTTCTGTTCTGACTGTGGG | 55 | 55 | 205 | calcium-dependent phospholipase, *Arabidopsis* | | | CA196464 | UGSuM574 | (GCC)5 | CDS | CCGACTTGAAACGAGACA | TGGTAATAGGACTGGATTGG | 55 | 55 | 280 | hypothetical protein, *Oryza* | | | CA197130 | UGSuM575 | (CAG)5 | CDS | TAACTCCTAAGTGAACCACCA | CAACTGAAAGAGACGGAAAC | 54 | 54 | 210 | BRI1-KD interacting protein 130, *Oryza* | | | CA201535 | UGSuM576 | (CGG)5 | CDS | AAGGTCGTCTCCATCCTG | TTGGTCTTCCAGTTGTTGTAG | 55 | 55 | 296 | CREB binding protein, *Arabidopsis* | | | CA201770 | UGSuM577 | (CGG)5 | CDS | TTAGGAAACACTCATCAATCC | GTCCAGAATCAACTGTAAGCA | 54 | 55 | 195 | hypothetical protein, *Oryza* | | | CA202200 | UGSuM578 | (CGG)5 | CDS | TAATCCATCAGAAGCACAGAA | CAAATACGGGTGGCATCA | 55 | 58 | 297 | glycosyltransferase, *Oryza* | | | CA202687 | UGSuM579 | (CGA)5 | CDS | CGGGATGGCGACTATGGG | CAATCTCTTTCCTCACAGCA | 64 | 56 | 335 | hypothetical protein, *Oryza* | | | CA202772 | UGSuM580 | (CGG)5 | CDS | TAGAAACCATTCGGAGTATCA | GTGGAGAGGATGAAGAGGA | 54 | 55 | 358 | myb family transcription factor, *Oryza* | | | CA203292 | UGSuM581 | (GGC)5 | CDS | CATCATCCTCTTCATCCTCTC | GATTGTGAGCAAACCCATT | 56 | 55 | 251 | hypothetical protein, *Oryza* | | | CA203958 | UGSuM582 | (TGC)5 | CDS | GCACAGGACACAAGAGAATAG | GCAGAGTAGACAATGAAGGAG | 55 | 54 | 175 | villin, *Arabidopsis* | | | CA206056 | UGSuM583 | (CGC)5 | CDS | TGCCCTGTGTTCCTTCCT | GGTGCTTGTTGACCATTCT | 59 | 56 | 377 | mitochondrial membrane translocase, *Arabidopsis* | | | CA206398 | UGSuM584 | (TCC)5 | CDS | GTCTACTCTGCTCTGGGTTC | CTGCTGCTTGTGGTTGTG | 53 | 57 | 141 | Clp amino terminal domain- protein, *Arabidopsis* | | | CA206719 | UGSuM585 | (GGA)5 | CDS | GCTACTCGCATCTGTCTGG | GCAATGGGCAACTAATAAAC | 57 | 54 | 211 | MYB transcription factor MYB135, *Glycine max* | | | CA207346 | UGSuM586 | (TCG)5 | CDS | CTCACCCACATACCTCACTC | GGGAAGGAACACAGGAAA | 55 | 55 | 256 | TPR repeat, *Medicago* | | | CA207663 | UGSuM587 | (GCG)5 | CDS | ATGTCTCAACCACCAAGTTC | AGCCCTCAAGTGTTACGAG | 54 | 55 | 358 | hypothetical protein, *Oryza* | | | CA208604 | UGSuM588 | (GGC)5 | CDS | CTCGTCTTCGGTGCTCTC | ACCTTTCCCTTCCGCCAA | 57 | 62 | 374 | hypothetical protein, *Oryza* | | | CA209243 | UGSuM589 | (GCG)5 | CDS | CGTTGCTCTTGTTCCTCTT | GACCTTTCCCTTCCGCCA | 55 | 63 | 191 | protein kinase, *Oryza* | | | CA217688 | UGSuM590 | (ATC)5 | 3'UTRs | CCTCTCCATTCCATACAGAC | CGTTTCTTTCCTCACCAAG | 54 | 55 | 286 | hypothetical protein, *Oryza* | | | CA219081 | UGSuM591 | (AGC)5 | 3'UTRs | ATCAAGGGAAACAACCAC | CATAAAGCATCTCCGCCC | 52 | 59 | 358 | hypothetical protein, *Oryza* | | | CA219145 | UGSuM592 | (GGC)5 | 3'UTRs | GAGGGAGAGAGGGACTCA | GGCACTTCTACTTTGGCTAC | 54 | 54 | 365 | hypothetical protein, *Oryza* | | | CA219729 | UGSuM593 | (CGC)5 | 3'UTRs | GTAGAACCACCCGTCCGCC | CTGTCCCGTTGAAGACTG | 65 | 54 | 374 | ribosomal protein L10 family protein, Arabidopsis | | | CA219937 | UGSuM594 | (GAG)5 | 3'UTRs | GGACGACGAGAACAAGGT | ACACCACACCACACCACA | 56 | 57 | 329 | zinc finger protein-like, *Oryza* | | | CA220664 | UGSuM595 | (TGG)5 | 3'UTRs | CGACGACTACCTCCTCAA | AGCCTCTTTCTGTTATTCCC | 54 | 55 | 352 | Helicase, C-terminal, *Medicago* | | | CA221150 | UGSuM596 | (TCT)5 | 3'UTRs | AGCAGAGCAGGTGGTGAG | ACGGAGAAGAGGTAGACAGAG | 57 | 55 | 174 | hypothetical protein, *Oryza* | | | CA221474 | UGSuM597 | (CGC)5 | 3'UTRs | CTTAGTGATGTGGAGGGACT | ATTTGCTTCATTGTGGAA | 54 | 52 | 273 | DNA repair protein *rad10,* RuvA domain 2, *Arabidopsis* | | | CA223143 | UGSuM598 | (GCC)5 | 3'UTRs | CATCTTTCTCCGTCAACCA | AGTAGCCGTCCTCCGCCAG | 57 | 65 | 121 | hypothetical protein, *Oryza* | | | CA224156 | UGSuM599 | (TGC)5 | 3'UTRs | GCCGAGGATAGAAAGAGATAC | CGATGTAGTGGATGTGGTC | 54 | 54 | 239 | hypothetical protein, *Oryza* | | | CA225244 | UGSuM600 | (GGA)5 | CDS | TTCAATCTAACACCTTTACCAA | TTCCATTCTAACCAGCAAA | 54 | 54 | 331 | myb-like transcription factor, *Zea mays* | | | CA225391 | UGSuM601 | (CAG)5 | CDS | AAGACCCGTGGAGCAGAG | GACGCCGACAGGACAAAC | 58 | 60 | 321 | *ATCDC5* (Arabidopsis homolog of cdc5), *Arabidopsis* | | | CA226040 | UGSuM602 | (CGC)5 | CDS | ATCAAGCACGCCCGCCTC | AAAGAAAGTCCAGAACCC | 67 | 51 | 312 | RNA export 1, *Nicotiana* | | | CA226051 | UGSuM603 | (AGC)5 | CDS | ATCAAGCACGCCCGCCTC | AAAGAAAGTCCAGAACCC | 67 | 51 | 312 | IAA13 (indoleacetic acid-induced protein 13), *Arabidopsis* | | | CA226086 | UGSuM604 | (AGC)5 | CDS | CTTTGGCTCTGTTATCTTCTG | CGTGTGGGTGCCTTCTCC | 54 | 62 | 298 | transcriptional regulator, GntR family, *Oryza* | | | CA226502 | UGSuM605 | (CAG)5 | CDS | TAACGGAGAACGACCTGA | GAGGAGTAAACCGCAGAAG | 55 | 55 | 390 | ABC transporter, *Arabidopsis* | | | CA226506 | UGSuM606 | (CGC)5 | CDS | GGCAAACGCAACACGAAG | AACAGCCAAGATGATTTCC | 61 | 55 | 351 | RNA and export factor binding protein, *Oryza* | | | CA226976 | UGSuM607 | (GGC)5 | CDS | CTGGTGTTGCTGGTGTAGT | GAGAGTTCCTGTCCAAGTGA | 54 | 55 | 381 | RRP41 (Ribosomal RNA Processing 41), *Oryza* | | | CA227013 | UGSuM608 | (GGC)5 | CDS | GGGCTATTACCACACAGACTAC | GCTAACACCACCAGCACTAA | 55 | 56 | 331 | Zinc finger, RING-type, *Medicago* | | | CA227263 | UGSuM609 | (AGC)5 | CDS | AGAACACTAACCACCACCC | CTCGCCGTCCTGCTTTGAG | 54 | 64 | 235 | zinc ion binding factor, *Arabidopsis* | | | CA227455 | UGSuM610 | (CCG)5 | CDS | CGACCTTATCCTCTTCCTCT | GGCTGTCTTCTCCTTCCC | 55 | 57 | 213 | glycine decarboxylase complex H-protein, *Populus* | | | CA227823 | UGSuM611 | (GGC)5 | CDS | AGGTCATCTCTCTCTTCTCGT | CTCCTTCTCCTCCTTCTTGT | 54 | 55 | 217 | prephenate dehydratase family protein, *Arabidopsis* | | | CA228227 | UGSuM612 | (AGG)5 | CDS | ACAAGGATGGAGAACAACAG | ATGAATGAACAAGCGAAGAAC | 55 | 56 | 152 | pyruvate kinase, *Arabidopsis* | | | CA230003 | UGSuM613 | (AAG)5 | CDS | ATACGAACAGGCAGCACTT | GGGAGATGGAGAAGGTCA | 55 | 55 | 233 | hypothetical protein, *Oryza* | | | CA232490 | UGSuM614 | (ACG)5 | CDS | TACACCGTCTTCTCCGCC | TGATACTCCTTTCCCTCTCTG | 59 | 56 | 400 | NPR4 (NPR1-LIKE PROTEIN 4), *Arabidopsis* | | | CA232822 | UGSuM615 | (GCC)5 | CDS | TTGTTAGTTTATTGGAGGGAA | GGCACATCTCTTGCTGTC | 54 | 55 | 279 | AP2/EREBP transcription factor, *Oryza* | | | CA234396 | UGSuM616 | (TGA)5 | CDS | TACTTCTTCGTCCCGTCC | ACACCACACTCATCTTTCATC | 56 | 54 | 279 | unknown protein, *Arabidopsis* | | | CA235607 | UGSuM617 | (GCC)5 | CDS | TGAGAACAAATGCCCAAC | GAAGGGTGGAATCGTCTT | 55 | 54 | 342 | Dcp1-like decapping family, *Oryza* | | | CA236058 | UGSuM618 | (CAC)5 | CDS | CAAGAACGGCTACGGCGA | AGGAAACCAGATACCAGAGAG | 63 | 55 | 327 | Lon family ATP-dependent protease, *Arabidopsis* | | | CA237505 | UGSuM619 | (CCG)5 | 5'UTRs | CTCCTCTCCCTCTCCCTT | GCCACTTTATCATCCTCAGTT | 55 | 55 | 341 | hypothetical protein, *Oryza* | | | CA238108 | UGSuM620 | (TGG)5 | 5'UTRs | CCACACTACCTGCCTTTCT | GTCTTCGCAATGTCAACC | 55 | 54 | 184 | Alcohol dehydrogenase, *Arabidopsis* | | | CA238327 | UGSuM621 | (GCG)5 | 5'UTRs | CTCCTGCCCTGCCTTTCC | TGGTTCTGTTCATCTTAGCAC | 63 | 55 | 363 | hypothetical protein, *Oryza* | | | CA238799 | UGSuM622 | (GGC)5 | 5'UTRs | CTCCCTCGGTTTCCTCTC | TGCGTAATAATCCTTCCAGAC | 57 | 56 | 398 | hypothetical protein, *Oryza* | | | CA239937 | UGSuM623 | (ACG)5 | 5'UTRs | GTATCCTGCGAGAACAAATC | GGGCAACAGAACCCACAG | 54 | 60 | 226 | unknown protein,*Triticum* | | | CA243229 | UGSuM624 | (AGC)5 | 5'UTRs | ACCTCCTCTTCCTTTCCTATC | CAGAGTAAGAGTAACTCCAGCC | 54 | 55 | 384 | beta-1,3 glucanase, *Oryza* | | | CA243647 | UGSuM625 | (GCG)5 | 5'UTRs | GTCTCTTCTCCAGTTCTCCTT | GTCTTCTCCACAACCACCT | 54 | 54 | 260 | AT-rich element binding factor3, *Pisum sativum* | | | CA243926 | UGSuM626 | (CGC)5 | 3'UTRs | TTGCGGCGAGCATCTTAC | CTTGTTGTTCCAGGTATCCA | 61 | 56 | 345 | hypothetical protein, *Oryza* | | | CA243968 | UGSuM627 | (CGA)5 | 3'UTRs | CACACACGGGAATAACCT | CACTGAGCAAGCAATGTC | 54 | 53 | 389 | ring-H2 zinc finger protein, *Oryza sativa* | | | CA244636 | UGSuM628 | (TGC)5 | 3'UTRs | CTCTCCCGTTCCCATCTC | ATGTAGACCATCTCCTCCC | 58 | 53 | 103 | NB-ARC domain containing protein, *Arabidopsis* | | | CA244680 | UGSuM629 | (ACG)5 | 3'UTRs | AGCAGAGACACACGCACA | ACAAGAGGAGGTTCAGGG | 57 | 54 | 161 | hypothetical protein, *Oryza* | | | CA245260 | UGSuM630 | (GCG)5 | 3'UTRs | GAGGTGGTGGTGGTGGAG | AGGGTTTGAGGTTTGATTG | 59 | 55 | 337 | ABC transporter ATPase, *Arabidopsis* | | | CA248113 | UGSuM631 | (CCG)5 | 3'UTRs | TATCCCACTACCAACACCAC | ATGAACACGACACACATTACA | 55 | 54 | 365 | ribosomal protein RL5, *Cicer arietinum* | | | CA248171 | UGSuM632 | (GCC)5 | CDS | CCTCATAACCGAGAAGAACTG | TACCTCCACTGCCACGAC | 56 | 58 | 247 | 3-oxoacyl-[acyl-carrier-protein] reductase, *Arabidopsis* | | | CA248880 | UGSuM633 | (CCG)5 | CDS | GGTGGGTGGATGATAGAAA | CTTCGTCGGTCGGGAATCT | 55 | 62 | 263 | microtubule-associated protein, *Oryza* | | | CA251888 | UGSuM634 | (AAG)5 | CDS | AAAGTTGAGCGTTTCTTGG | TCATAGTTCTGCTGCTGTTG | 55 | 55 | 335 | IAA1 protein, *Triticum* | | | CA251891 | UGSuM635 | (CGG)5 | CDS | GACTACGCCTTCTCCTCC | ATGAGTTCCTTCTCGCACT | 54 | 54 | 359 | GDSL-motif lipase, *Arabidopsis* | | | CA252581 | UGSuM636 | (CGC)5 | CDS | TCGGGAGAGGTATGGTAG | TTCAGCAAGAAATGGGTG | 53 | 55 | 280 | hypothetical protein, *Vitis vinifera* | | | CA252819 | UGSuM637 | (GGC)5 | CDS | CAAACCGAAGAGACCACA | CCTTGAGACTGGAGATAGCA | 55 | 55 | 363 | unknown protein, *Arabidopsis* | | | CA252869 | UGSuM638 | (CAG)5 | CDS | ACAGCACAGGCTCTCTCTT | TCCTTTCAGGCATCCATC | 55 | 56 | 178 | serine/threonine-protein kinase NAK, *Arabidopsis* | | | CA252938 | UGSuM639 | (GTG)5 | CDS | AGGCAGAGCAAGGTCAGT | AAAGAATGAATGGAAGGATTT | 55 | 54 | 309 | Zinc finger, RING-type, *Medicago* | | | CA253365 | UGSuM640 | (GCC)5 | CDS | CGATTGATTATTTCTTGCTGA | CATACGCCTGCTCCAACC | 55 | 60 | 291 | Transcription factor IIA, *Medicago* | | | CA254456 | UGSuM641 | (CTG)5 | CDS | GCCTGCTTCGTCCTCTCC | GACCACTACCCGCCAGTT | 61 | 58 | 272 | hypothetical protein, *Oryza* | | | CA256489 | UGSuM642 | (CGC)5 | CDS | GTTTACATCCACCTCCGCC | GCTCTCCCTTCATCTCCTC | 60 | 56 | 302 | Heat shock protein Hsp20, *Medicago* | | | CA256691 | UGSuM643 | (GCC)5 | CDS | TCCTCCTCCTCCTCAGTC | GAATGGCTGGAACACAAC | 54 | 54 | 211 | ribosomal protein L6, *Oryza* | | | CA257758 | UGSuM644 | (AGC)5 | CDS | TAATAGAGGTGGATTTGGAC | CTTTCTTTCATTGTTGTCGTT | 51 | 54 | 364 | senescence-associated protein DH, *Zea mays* | | | CA258116 | UGSuM645 | (CAG)5 | CDS | CACCCACGCAGCCTCACT | GATTCTCTCCCTAACAGCAA | 63 | 54 | 317 | hypothetical protein, *Oryza* | | | CA259134 | UGSuM646 | (GTG)5 | CDS | ATTGGAGATGGTTTATTTCA | GACACACGCCAGGTAGATT | 52 | 56 | 178 | 40S ribosomal protein S10, *Solanum* | | | CA260566 | UGSuM647 | (CAC)5 | CDS | ACCCAGAAGGAGAAGAAGAG | GGACACCAGTTTGAAGGTC | 55 | 55 | 217 | hypothetical protein, *Oryza* | | | CA261253 | UGSuM648 | (AGC)5 | CDS | ATCCATCAACGCAGGGTC | GTCCTCAGCATCATCCAA | 59 | 55 | 336 | hypothetical protein, *Oryza* | | | CA261687 | UGSuM649 | (AGA)5 | CDS | CGCACTTGACTGTGGTGA | GTGGTGGAGAACTTGGTG | 57 | 54 | 232 | expressed protein, *Oryza* | | | CA261861 | UGSuM650 | (CGG)5 | CDS | CTTACACCATCAGCACCTC | TTCTCTCCCACACACACAC | 53 | 55 | 296 | ATP-dependent DNA helicase, *Arabidopsis* | | | CA262420 | UGSuM651 | (CCG)5 | CDS | TACGCTGTGATACGCTTG | CCTTCTACTTTCGTCGTTCTC | 54 | 55 | 362 | acetyl-CoA carboxylase, carboxyl transferase, *Arabidopsis* | | | CA263669 | UGSuM652 | (GAT)5 | CDS | TCTTCAACTTCCTCTGCCT | GTTCCTGACTGTTCCCTTG | 55 | 55 | 319 | Cytochrome b561 family protein, *Oryza* | | | CA264686 | UGSuM653 | (GGC)5 | CDS | GCTACTACTCTCCGTGTTCCT | GACAATGATGTTCTCGTCCT | 55 | 55 | 244 | peptide ABC transporter ATP-binding protein, *Arabidopsis* | | | CA264842 | UGSuM654 | (CCG)5 | CDS | ATCTCTCTCGTTCGTCGT | GTTGTTCTGCTTCAGGATGT | 52 | 55 | 314 | 4Fe-4S ferredoxin, iron-sulfur bindingprotein, *Medicago* | | | CA265230 | UGSuM655 | (TCG)5 | CDS | CTTCTCCTTGGGTGGCTC | AGGGACCATTTCCAGGTT | 58 | 56 | 386 | expressed protein, *Oryza* | | | CA266038 | UGSuM656 | (GAA)5 | CDS | CGACTCCACACTCCACTC | CCGAACACCACCTTCTTG | 54 | 57 | 347 | expressed protein, *Oryza* | | | CA266676 | UGSuM657 | (GGC)5 | CDS | AGACGACGGCAGAGTGAG | AAGTTTGAGATTGTTTCAGGG | 57 | 55 | 392 | hypothetical protein, *Oryza* | | | CA267701 | UGSuM658 | (AAG)5 | CDS | CCCATTCCTCAAATCTTATTC | ACGACCGCCCGCCTACTCAC | 55 | 68 | 400 | hypothetical protein, *Oryza* | | | CA268992 | UGSuM659 | (TCC)5 | CDS | CGTCGTGAAGGCTGAGGG | GTAACATTCTTGAGGGTCCA | 63 | 55 | 393 | hypothetical protein, *Oryza* | | | CA269918 | UGSuM660 | (GAG)5 | CDS | CTCACCACTGCTCTCCATCT | CCACCAGCACCTTCTCGATG | 55 | 55 | 303 | P-type R2R3 Myb protein, *Sorghum* | | | CA270378 | UGSuM661 | (CGC)5 | CDS | GTCGTCTTCCTCTACTGGTTC | CTCTTTCCTTCTCTACGCAA | 55 | 55 | 375 | AP2 type transcription factor, *Oryza* | | | CA270923 | UGSuM662 | (CTC)5 | CDS | TGGATTTGATTTCGTGACTT | CTACTCTCATTGCTGCCAC | 55 | 54 | 372 | uracil phosphoribosyltransferase, *Arabidopsis* | | | CA271355 | UGSuM663 | (CGC)5 | CDS | GTGTCTTTGTGGCTGAGG | ATTACATCATCTGGTTGTGCT | 54 | 54 | 353 | 60S ribosomal protein L17, *Arabidopsis* | | | CA272222 | UGSuM664 | (GCT)5 | CDS | CCTCTACAAACGGCTCTCT | CAGTAACAACAACCACAGGAC | 54 | 55 | 286 | CD9/CD37/CD63 antigen, *Medicago* | | | CA272409 | UGSuM665 | (AGG)5 | CDS | TTCATCACTGTCGGAATCTAC | ACGCTCTCAACACCTTTATTT | 55 | 55 | 342 | hypothetical protein, *Vitis vinifera* | | | CA272642 | UGSuM666 | (CGG)5 | CDS | AGGAGAAGCCTGAAGAGAAG | ACACCTCGTTGGAGTTAGAG | 55 | 54 | 315 | hypothetical protein, *Oryza* | | | CA272711 | UGSuM667 | (CCA)5 | CDS | CTCACATTTCTTCCCTTCTCT | ATAGCCTCGGTTTCATTCC | 55 | 56 | 231 | signal transduction histidine kinase, *Arabidopsis* | | | CA274721 | UGSuM668 | (GGC)5 | CDS | ACTTGCCCTTGCTTGATT | ATTCTTTCGTTAGTTGCGG | 55 | 55 | 119 | hypothetical protein, *Oryza* | | | CA275058 | UGSuM669 | (GCT)5 | CDS | TACCAATCAGCAATCAAGAC | CAACATCCAGAACAAGCAC | 53 | 54 | 329 | calmodulin-like protein, *Arabidopsis* | | | CA275224 | UGSuM670 | (GGC)5 | CDS | TTTCCATTCACTGTCTCACTC | ACATTACCATACTGCCCAAAC | 55 | 56 | 214 | Expressed protein, *Oryza* | | | CA275285 | UGSuM671 | (CTC)5 | CDS | TTTACATCACAACCTTCCGT | AACCCTTCTCCTGTCACTCT | 55 | 55 | 384 | zinc finger protein, *Oryza* | | | CA275856 | UGSuM672 | (CGG)5 | CDS | GATGCGATTTCTAAGGATGAC | AGGACAGAGGATTTACCACTT | 56 | 54 | 317 | hypothetical protein, *Vitis vinifera* | | | CA276123 | UGSuM673 | (TCC)5 | CDS | CTATCTCTATCTGCTTCCTGCT | TTGTGAACTGCCTTCTTCTT | 54 | 55 | 177 | acid phosphatase, *Arabidopsis* | | | CA276157 | UGSuM674 | (CCG)5 | CDS | ATTGTTGATTTGCTTCAGGT | AAAGGTTACGGGAGAGGAG | 54 | 55 | 289 | Calcineurin B subunit, *Oryza* | | | CA279093 | UGSuM675 | (GGC)5 | CDS | AGAACAACCCTTCCGTGT | TCATCAACATCAACTTTCAG | 55 | 51 | 318 | purple acid phosphatase 2, *Solanum tuberosum* | | | CA279108 | UGSuM676 | (CCG)5 | CDS | GATAGGTTAGCGACACATT | GCCTTGCTTTACTACGGTT | 50 | 54 | 193 | hypothetical protein, *Oryza* | | | CA279291 | UGSuM677 | (GCG)5 | CDS | CCTCTCCTCTCTCTCTCTCTC | GCTGTTCCTTGTGCTTGT | 54 | 54 | 341 | peroxidase, *Oryza* | | | CA279499 | UGSuM678 | (GGC)5 | CDS | GTGGACGAGAAGTGGAAGT | ATAGGAGGGCAGGACAAG | 55 | 55 | 370 | transmembrane CLPTM1 family protein, *Arabidopsis* | | | CA279564 | UGSuM679 | (TCG)5 | CDS | TAGAGCCATAGAGGCGATAGA | CTACGAGGAAGAACGGCGGG | 56 | 66 | 395 | BTI1 (VIRB2-INTERACTING PROTEIN 1), *Arabidopsis* | | | CA279822 | UGSuM680 | (CCG)5 | CDS | TTCTTGCGGTTCTTCTTG | CATCTCCTTCCCACCTTC | 54 | 55 | 170 | Heat shock protein Hsp20, *Medicago* | | | CA279920 | UGSuM681 | (CCG)5 | CDS | GTGTGCCTGTGTCTGTGTT | GTCCAGTTTCCATTCGGT | 55 | 55 | 307 | Ubiquitin-conjugating enzyme, *Oryza* | | | CA280184 | UGSuM682 | (GCT)5 | CDS | AATCTCTTTCTCCCTCTTATCC | TTCTACCCTAACCTTCCAGAC | 54 | 55 | 278 | myb transcription factor, *Arabidopsis* | | | CA280497 | UGSuM683 | (CGG)5 | CDS | ATGCCTTGAAATCTGCTC | CTCCGTTCCTCTCCATCC | 53 | 58 | 259 | cyclophilin, *Triticum* | | | CA280557 | UGSuM684 | (GCC)5 | CDS | TATTTACAACGGGCAACATC | TGAATGAACAGCAGCAAGT | 56 | 55 | 137 | S-adenosylmethionine synthetase 1, *Triticum* | | | CA280813 | UGSuM685 | (GAA)5 | CDS | TTATTTCGCTTCACTGCC | AACTCCGACTGCCCTCCC | 54 | 62 | 400 | hypothetical protein, *Oryza* | | | CA281868 | UGSuM686 | (CAG)5 | CDS | CCACTCAACCTCTCCAACT | TTCTTCTTCTCAGTCTCCTCA | 55 | 54 | 189 | hypothetical protein, *Oryza* | | | CA282616 | UGSuM687 | (CTT)5 | CDS | AGATGAGGAAGAGGATGATG | GGTAGGTGTGGGAGCACTT | 54 | 57 | 274 | glutaredoxin family protein, *Arabidopsis* | | | CA282642 | UGSuM688 | (CGC)5 | CDS | AGGCGTGGTGGAAGGAGG | GAACTGGCTCGTGATGGT | 63 | 57 | 290 | unknown protein, *Oryza* | | | CA285497 | UGSuM689 | (TGG)5 | 5'UTRs | TCTACTATTGCTTCCAACACTTT | AGAGCGAAATCACATCATC | 55 | 53 | 320 | alpha-galactosidase, *Oryza* | | | CA286227 | UGSuM690 | (GGC)5 | 5'UTRs | GGTGAGGTGAGATGAGGAG | CGCCGAAGTTGAAGATGG | 55 | 60 | 135 | phytosulfokine-alpha 1 precursor, *Triticum* | | | CA286569 | UGSuM691 | (CAG)5 | 5'UTRs | AGAACAATGGACACCTGAAC | GAGAGAACCACAACCGAAC | 54 | 55 | 118 | unknown protein, *Arabidopsis* | | | CA289297 | UGSuM692 | (AGA)5 | 5'UTRs | CTTTCGCTTTCTCCCGTC | GTTGTCACGAGCCGCATT | 58 | 60 | 291 | SAR DNA binding protein, *Oryza* | | | CA289987 | UGSuM693 | (GCG)5 | 5'UTRs | ATCTCCGCCCACCTCTCCT | ACCTTGCTTACATCTTCCTCT | 63 | 54 | 395 | Mak3 protein-like, *Oryza* | | | CA293318 | UGSuM694 | (GGC)5 | 5'UTRs | AGATACCAGAAGCAACACTGA | GATGAACCCTTGACTGAGAA | 55 | 55 | 346 | GTP-binding protein, *Oryza* | | | CA294163 | UGSuM695 | (CCG)5 | 5'UTRs | AAACCAACAACTACCACAGG | CTCCATCAAGAGTCAAACAAC | 55 | 54 | 375 | hypothetical protein, *Oryza* | | | CA297374 | UGSuM696 | (GAT)5 | 5'UTRs | CGTTCATTCATCTCATTCTGT | CAACCTTTGGTATTTCTGCT | 55 | 54 | 310 | hypothetical protein, *Oryza* | | | CA297408 | UGSuM697 | (GCC)5 | 5'UTRs | CCTCCTGTTCCTGGTCGT | TACTTGGTGCCGTGCTTG | 58 | 59 | 381 | mitochondrial transcription termination factor, *Arabidopsis* | | | CA301471 | UGSuM698 | (CGC)5 | CDS | TTTGTTTGCCTTCAGTTCA | GTTGTTCATTGCTCATCCA | 55 | 55 | 241 | hypothetical protein, *Oryza* | | | AY596551 | UGSuM699 | (CT)7 | CDS | ACATGCCAAGAAGCAGAAGAAG | ACGTAGAAGTAGACCTTCCAGTT | 60 | 55 | 329 | hypothetical protein, *Oryza* | | | AY644463 | UGSuM700 | (AG)7 | CDS | AATACAGCGCAATATTTCCTCC | ATAACCTCAACGCCAAGAGCAAGT | 59 | 64 | 341 | mitochondrial uncoupling protein 4, *Saccharum* | | | BQ533822 | UGSuM701 | (GC)7 | CDS | CTGTGCTCCTGCCCTGCCTTTC | AATAATTCTGCCTCTGATAGAAAC | 66 | 56 | 400 | hypothetical protein, *Oryza* | | | CA074021 | UGSuM702 | (AG)7 | 5'UTRs | CCATAAGCAAAGAGGGTGGAAC | AGTTTAACTAGCGCTAAGGGTAT | 62 | 55 | 178 | leucine zipper transcription factor, *Arabidopsis* | | | CA074508 | UGSuM703 | (CT)7 | CDS | CTTTCCCTCTTCCTCTCTCGTAG | CTTCAATGTTTGTATTGGATAAAG | 60 | 54 | 139 | cytochrome c oxidase, cbb3-type, subunit III, *Oryza* | | | CA076102 | UGSuM704 | (TA)7 | CDS | AAATTGGATCTCGTCTTCTCTC | CAGGGAATTGTACATTCTTAGAC | 56 | 54 | 204 | Unknown protein, *Arabidopsis* | | | CA087531 | UGSuM705 | (AC)7 | CDS | ACACCTGGAACCAGTTCCTCTAC | AAGAATCGAAGGTTAACAACAG | 60 | 55 | 292 | Acyl carrier protein (ACP), *Medicago* | | | CA098584 | UGSuM706 | (CT)7 | CDS | AACCCACCAATATACTACCTACAG | CTACAAGATGCCAAATATGGTT | 60 | 55 | 316 | mitochondrial half-ABC transporter, *Arabidopsis* | | | CA105296 | UGSuM707 | (AG)7 | 3'UTRs | ATTACAACTACACATGTCTTGACC | GTACTACATGCCTCTCACTCTGT | 55 | 58 | 255 | PHF1 (PHOSPHATE TRANSPORTER), *Arabidopsis* | | | CA105959 | UGSuM708 | (TC)7 | 3'UTRs | CCAAGAAGGCTGCCTCGTCTAA | AGAAGGTTATGACTGTTGGAAG | 65 | 57 | 355 | alliinase precursor, *Oryza* | | | CA106561 | UGSuM709 | (CT)7 | CDS | TATCTGATCGGTAGCAAATAGC | GTGGTTAAGAAGAGACTAAGTTCG | 55 | 55 | 210 | expressed protein, *Oryza* | | | CA110731 | UGSuM710 | (CT)7 | 5'UTRs | AACATCAAAGTTGAGTCGTACTG | TAAGATAGTCTTGGACAGCATAAG | 61 | 65 | 318 | zinc finger (C3HC4-type RING finger) family, *Oryza* | | | CA111572 | UGSuM711 | (GT)7 | CDS | AAATTCTTCCTTCTGTCCACTT | CTATCACACAGCTATCATCATCA | 54 | 54 | 257 | hypothetical protein, *Vitis vinifera* | | | CA122092 | UGSuM712 | (GA)7 | 5'UTRs | GATTCACAAATACTAACGCAA | CACCTAACTCTGTCATCTGCT | 53 | 54 | 397 | senescence-associated protein, *Arabidopsis* | | | CA128736 | UGSuM713 | (GA)7 | CDS | CTACGGCACAAATACGAAA | ACAAACCACGGAACAAGAC | 54 | 55 | 168 | Gtk16 protein, *Medicago* | | | CA132059 | UGSuM714 | (CG)7 | 5'UTRs | CAGGTGGTGATTGACGAA | ATGGCAAAGTATGGGTTATG | 56 | 55 | 377 | DNA-binding protein, *Oryza* | | | CA134844 | UGSuM715 | (TG)7 | 3'UTRs | GATGTAAGGCGAGGTGGG | GCGGGTTGTTGGAAGACA | 59 | 60 | 173 | oxysterol 7alpha-hydrolase, *Arabidopsis* | | | CA155290 | UGSuM716 | (TC)7 | CDS | TTGGACGAAACAAATACAAG | CAGCCAGACAACACCTACA | 53 | 55 | 252 | unknown protein, *Arabidopsis* | | | CA158818 | UGSuM717 | (GT)7 | CDS | CATGAGCCTTTGGATGTAGTT | TCAGAGTTTCCTTGACCCA | 55 | 55 | 379 | CDPK-related protein kinase, *Oryza* | | | CA159725 | UGSuM718 | (CT)7 | 5'UTRs | AATTTAGGGTTTGTTCGCCT | TGTGTAAATCTGAACTTCATCC | 56 | 54 | 235 | expressed protein, *Arabidopsis* | | | CA165112 | UGSuM719 | (CT)7 | 5'UTRs | ATCTCAGTCAGTCTCCCACA | ATAGGGTTTGCCGTTGTTC | 55 | 57 | 303 | somatic embryogenesis receptor kinase, *Arabidopsis* | | | CA167796 | UGSuM720 | (CA)7 | CDS | GCCAGACAGCGACCAGAG | CCCAGAGGGAGGGTAAGG | 60 | 59 | 294 | 40S ribosomal protein S26, *Oryza* | | | CA183663 | UGSuM721 | (CA)7 | CDS | ATAACACACACACACACAAA | TTCCGAGGGCAAATAATC | 50 | 56 | 174 | ABC transporter, ATP-binding protein, *Arabidopsis* | | | CA184137 | UGSuM722 | (GA)7 | CDS | GCAATCAGCCTTACAGCA | GAACATCGCACTTTGGAG | 55 | 55 | 310 | Phospholipase A2, *Medicago* | | | CA184950 | UGSuM723 | (GA)7 | CDS | GTCTGAACAAGAGCGACAA | TAGGATTACACACCACACCAC | 54 | 55 | 398 | transmembrane protein-like protein,*Triticum* | | | CA203271 | UGSuM724 | (GA)7 | CDS | GTCATCGTCATCCTCCTCT | GATTGTGAGCAAACCCATT | 54 | 55 | 331 | integral membrane family protein, *Arabidopsis* | | | CA206091 | UGSuM725 | (AG)7 | CDS | GTCATCTCCATCGCCTCCC | CGAAGTTTGGGTCGTTGA | 62 | 58 | 334 | cytochrome P450 monooxygenase CYP77B5, *Medicago* | | | CA212706 | UGSuM726 | (GA)7 | CDS | AAACAATCCAAACCAACAAC | GCTACAAGAACTCGCCCT | 54 | 55 | 297 | CCAAT-binding transcription factor, *Oryza* | | | CA213608 | UGSuM727 | (GT)7 | CDS | CCGCTTTCAACCTCTACAC | GGCTTGGTGATTCTTCTCT | 55 | 54 | 285 | 2-oxoisovalerate dehydrogenase alpha subunit, *Oryza* | | | CA215850 | UGSuM728 | (GA)7 | 3'UTRs | GTGCTGAGGCTGTCGTGT | GCATAGGACTTGGGCATT | 58 | 56 | 231 | Zinc finger, RING-type; TRAF, *Medicago* | | | CA234016 | UGSuM729 | (TC)7 | CDS | GAGTGGAACAATGAAACCAA | TAATGTGGAATAATGCTCTG | 55 | 51 | 103 | hypothetical protein, *Vitis vinifera* | | | CA254514 | UGSuM730 | (CG)7 | CDS | CTGCTGTTCCTCTTATTGCT | CGGAGACTGAAACCTCGT | 54 | 56 | 123 | calcium-dependent protein kinase, *Zea mays* | | | CA261827 | UGSuM731 | (CT)7 | CDS | ACTCGTCAGCCAGCAGTT | ATGAGGGTGTGATACGGTT | 56 | 55 | 309 | hypothetical protein, *Oryza* | | | CA264205 | UGSuM732 | (TG)7 | CDS | GAGAGCAGACGACGGAGG | ATAGTCCACAAGAACGAACA | 59 | 53 | 235 | high-molecular-weight glutenin subunit, *Triticum* | | | CA270150 | UGSuM733 | (TC)7 | CDS | TCTGTTTCAGTCGTTTCCTAC | CGATTTCTTTCTTTCTTCTCC | 54 | 55 | 128 | acetyl-CoA C-acetyltransferase, *Arabidopsis* | | | CA270995 | UGSuM734 | (AT)7 | CDS | AGATAACAGACTTGGGCAGA | ATTGGTTGGAAATGATGCT | 54 | 55 | 307 | hypothetical protein, *Oryza* | | | CA272719 | UGSuM735 | (TA)7 | CDS | TAGTGATGCTCTCGCAACC | ATCTCAGTCAGTCTCCCACA | 57 | 55 | 330 | ORMDL family protein, *Arabidopsis* | | | CA279539 | UGSuM736 | (GA)7 | CDS | AGCACGAAGCAATACCAG | AACGGACACCACCACTAC | 54 | 53 | 341 | C2-H2 zinc finger protein, *Arabidopsis* | | | CA282704 | UGSuM737 | (TA)7 | CDS | AATTGGATCTCGTCTTCTCTC | ACCAGGGAATTGTACATTCTTA | 54 | 55 | 379 | unknown protein, *Arabidopsis* | | | CA285973 | UGSuM738 | (TC)7 | 5'UTRs | CTACTGAGAGAGCGAAGCAC | GGAACACGAAACCACCTT | 55 | 55 | 148 | phosphatase, *Glycine max* | | | AY521566 | UGSuM739 | (AG)6 | CDS | TAATATACATCACGACACAACAAA | TTGTGTTATCTAGACGATACCTTT | 55 | 54 | 348 | 1-aminocyclopropane-1-carboxylate oxidase, *Saccharum* | | | AY596526 | UGSuM740 | (GA)6 | 5'UTRs | GTACTGAGAGCCAAAGAAACAAC | CTCTTCTTGAGCTGTCTCCAAGC | 57 | 62 | 217 | SM10, *Nicotiana* | | | AY596561 | UGSuM741 | (GA)6 | 3'UTRs | GTTTCCTTCCTTCTCGTGCCAT | CCTAATAAGTATCGTTGAAGGTG | 63 | 54 | 331 | protein phosphatase, *Arabidopsis* | | | BU102605 | UGSuM742 | (AC)6 | 5'UTRs | GTGAATCTGCAGGCTGCTGGAAG | GCACTAGTCACTACTACACACGC | 55 | 69 | 232 | glycosyltransferase, *Saccharum* | | | BU103681 | UGSuM743 | (GA)6 | CDS | AGTAGCCAGGATGGTACTTGTC | GACCCTAATTGTAGTTCTCTCAA | 55 | 54 | 399 | G-box binding factor1, *Arabidopsis* | | | CA065380 | UGSuM744 | (CG)6 | CDS | AAGGCGAGCAGAGCACATCAAT | GAGGTGTAGTTGAAGTAGTCGTAG | 54 | 55 | 278 | catalytic/ methionine gamma-lyase, *Arabidopsis* | | | CA067783 | UGSuM745 | (TC)6 | 5'UTRs | ACAGATCACAATCAACCAAACTA | AATGTCGAAATGGAACATTAAC | 54 | 66 | 346 | Unknown protein, *Arabidopsis* | | | CA072593 | UGSuM746 | (GA)6 | CDS | TGAGAATTTACTGATTTACAGTGC | GAGTTTGACCTCAATGAAACTG | 55 | 56 | 141 | VIP1 protein, *Avena* | | | CA074584 | UGSuM747 | (CT)6 | CDS | ACTTCATCTCTCGACGCCCATCT | CTCGTACTCCTTGACCTTGAGT | 65 | 57 | 364 | Zinc finger CCCH domain protein, *Arabidopsis* | | | CA085169 | UGSuM748 | (CT)6 | CDS | ATCTTTCTCGTCCGCCTCCGCT | GTCATCAATGACAGAGATGTAGAC | 69 | 55 | 163 | beta-glucosidase aggregating factor | | | CA086761 | UGSuM749 | (TA)6 | CDS | CAAATCCTTGTGCTTCAGATCG | CACTCTCAAATCTTGGAGAATAAT | 62 | 55 | 396 | endo-1, 4-beta-glucanase, *Arabidopsis* | | | CA087984 | UGSuM750 | (GA)6 | 5'UTRs | CCCGACCTTATCCTCTTCCTCT | GGAGAAGGAAACTCTGGAGCAG | 62 | 61 | 270 | Ubiquinol-cytochrome c reductase, *Oryza* | | | CA088533 | UGSuM751 | (CG)6 | 5'UTRs | CTCTCCTCACTTTCTCCCATTC | ATCTTGGCGGTGTCGATCTTCT | 59 | 55 | 234 | Protein kinase, *Medicago* | | | CA091702 | UGSuM752 | (GC)6 | CDS | GCGTCTCTGCTCTGCACTCTGC | ATTAACATATTCATAGCCCAATTT | 62 | 59 | 120 | GTP binding protein, *Arabidopsis* | | | CA093469 | UGSuM753 | (CA)6 | CDS | TACACTCACACTCACACACGTCTC | ATGTTGACGCTCTTGACGCTGT | 65 | 54 | 390 | hypothetical protein, *Oryza* | | | CA097999 | UGSuM754 | (CT)6 | 5'UTRs | CCCAGAACTGGAACCTCATC | ACGTGCCAGTGGTTCTTGAC | 69 | 55 | 354 | homogentisate phytyltransferase VTE2-1, *Triticum* | | | CA103036 | UGSuM755 | (TA)6 | CDS | ATTCATACTCGAAGTTTGTATGC | ACTGCACCGACATGATGTTCTT | 61 | 55 | 185 | pre-mRNA splicing factor PRP38 protein, *Arabidopsis* | | | CA103254 | UGSuM756 | (GC)6 | 5'UTRs | GAGATCTCATCGAATAAGAAGC | ATTTGTGATTTCATCATCTTTCTT | 55 | 55 | 293 | alkaline alpha galactosidase2, *Zea mays* | | | CA103405 | UGSuM757 | (GA)6 | CDS | AGTGGAAGTTCTCCAAGAAGAG | AGTCCTTACAGCATACGAGCAT | 57 | 54 | 266 | hypothetical protein, *Oryza* | | | CA104763 | UGSuM758 | (AC)6 | 3'UTRs | GACTATATGTACACTGCTGCTGTT | TTATTACAGTTGTTGAGGTGAGTT | 55 | 54 | 313 | F-box family protein, *Oryza* | | | CA104898 | UGSuM759 | (CT)6 | 3'UTRs | AACTCACCTCAACAACTGTAATAA | CTCCAACATGTGGAAGTAGTTA | 55 | 55 | 115 | F-box family protein, *Oryza* | | | CA105897 | UGSuM760 | (AG)6 | 3'UTRs | ATGATGATGATAGCTGTGTGATAG | GAGAGTGAGCGTATTTCTTCTT | 55 | 54 | 264 | heat shock factor binding protein 2, *Zea mays* | | | CA106068 | UGSuM761 | (TC)6 | 3'UTRs | TACTCTGCAGTACGGTCCGGAATC | CAACTGACAGCCAATACTTCAC | 64 | 59 | 282 | phosphoglycerate mutase family protein, *Arabidopsis* | | | CA106128 | UGSuM762 | (AT)6 | 3'UTRs | CAGTAGACAAATCCAATGAAACT | TCTTGTTGAATACCTCTAGTTCTG | 56 | 62 | 245 | hypothetical protein, *Oryza* | | | CA111537 | UGSuM763 | (CT)6 | CDS | GGAAGCAGAGAAAGAGAGATATAC | GTTGGTGATGAAGTTGAGGCAC | 55 | 55 | 357 | TGACG-motif-binding factor, *Glycine max* | | | CA125488 | UGSuM764 | (CG)6 | CDS | CTAAGACAAGCAAGGCATCT | GTAGTTCCAGTAGCATCAGCA | 54 | 55 | 337 | Heat shock protein Hsp70, *Medicago* | | | CA126513 | UGSuM765 | (CT)6 | CDS | TGCTTGCTCTCCTATTACATT | GGGACTCGTTTGTTTCTTTAC | 54 | 55 | 382 | cytosolic factor-like protein, *Oryza* | | | CA130646 | UGSuM766 | (GC)6 | CDS | GCGGTGGTATTAGGAGATTAG | TATGGGTTATGGAAGCAGA | 55 | 54 | 349 | hypothetical protein, *Oryza* | | | CA130809 | UGSuM767 | (AT)6 | CDS | TGAGAGAACATCAACAGCAC | AGGAAAGCCCAGAAGAGA | 54 | 54 | 311 | hypothetical protein, *Oryza* | | | CA131374 | UGSuM768 | (CT)6 | 5'UTRs | AATCCATCTAAACATCACTGG | ACAAGAAAGGAGAGTTCCATC | 54 | 55 | 214 | hypothetical protein, *Oryza* | | | CA135123 | UGSuM769 | (TA)6 | 3'UTRs | CCTGACCAACAGCGGCAC | CCCAACAAGACATCACCA | 63 | 56 | 170 | small nuclear ribonucleoprotein, *Arabidopsis* | | | CA136602 | UGSuM770 | (CG)6 | 3'UTRs | CAGCAAATGATGTTCCACTC | ACCAGTTGCTCCTTGAGACT | 55 | 55 | 352 | CCAAT-binding transcription factor, *Oryza* | | | CA139255 | UGSuM771 | (TG)6 | 5'UTRs | GCACCGCTTCATCATCTAC | AAGGCTCAATGGTTCAAG | 56 | 53 | 400 | APETALA2 protein, *Arabidopsis* | | | CA140145 | UGSuM772 | (AG)6 | 5'UTRs | ACAACCTGTAGATGAACGAAA | TTACCTGAAGAGTGAAGACC | 54 | 51 | 299 | CBL-interacting protein kinase 1, *Populus* | | | CA140804 | UGSuM773 | (CG)6 | 5'UTRs | CGCCTCTCGTTCGCTCTC | CTAAGCCCACATCCTGGT | 62 | 55 | 196 | expressed protein, *Oryza* | | | CA150430 | UGSuM774 | (CT)6 | CDS | GAACCTGGCGATTTATGAG | TATGATTGAAAGACGGAACAC | 55 | 55 | 368 | leucine-rich repeat protein kinase, *Arabidopsis* | | | CA150625 | UGSuM775 | (GT)6 | CDS | GACGACTGACAAGGCGAG | GTAGAAGACCCAGAACCACA | 57 | 55 | 158 | serine/threonine protein kinase, *Arabidopsis* | | | CA157160 | UGSuM776 | (TA)6 | CDS | AAGACACTTAATCCCTTGAAGA | TTAAGAAGAGACTAAGTTCGCC | 55 | 54 | 333 | cyclophilin type peptidyl-prolyl, *Arabidopsis* | | | CA158542 | UGSuM777 | (CG)6 | CDS | TGCGTGCTCCACAACATC | GTGTCGGTTTCGTTCACC | 60 | 57 | 370 | phosphoenol pyruvate translocator, *Arabidopsis* | | | CA160082 | UGSuM778 | (AG)6 | 5'UTRs | CTGTCTCTTCCTCTCCTGGT | ACTAACCTTCTTCTTGGCGT | 55 | 55 | 309 | myb family transcription factor, *Oryza* | | | CA165870 | UGSuM779 | (CG)6 | CDS | AATGTCAGGCAGAGGGAG | GTAAAGTGATGAAAGTGCGG | 56 | 55 | 231 | thioredoxin h-like protein, *Hordeum vulgare* | | | CA166936 | UGSuM780 | (CG)6 | CDS | TTGTTTCAGAGCCGTCTACTAT | GTAATAGGACTGGATTGGAATG | 56 | 55 | 231 | ATCEN2 (CENTRIN2); calcium ion binding, *Arabidopsis* | | | CA168607 | UGSuM781 | (CT)6 | CDS | ATTTCTCGTGTTAGCGATGT | ACTTTCAGCCTCGTGTCC | 54 | 56 | 344 | Expressed protein, *Oryza* | | | CA184623 | UGSuM782 | (CT)6 | CDS | AAGCCTCTCCTCTCCTCC | CCTTGGTTCGGTCTATGTT | 55 | 55 | 245 | electron carrier/ iron ion binding, *Arabidopsis* | | | CA204853 | UGSuM783 | (AG)6 | CDS | GACAAGCAAGGGAAGGAA | GGAACAGAGCCAAGAGATG | 56 | 55 | 127 | glutamate synthase [NADPH], *Arabidopsis* | | | CA206057 | UGSuM784 | (GA)6 | CDS | AAGGACTACAAGGAGAAGCAC | GCAATCTAAGGAAAGACCAGA | 55 | 56 | 392 | hypothetical protein, *Oryza* | | | CA206551 | UGSuM785 | (CT)6 | CDS | AAACAAAGGAAGTAAAGAGAAA | CGGAGCGACGACAACCAG | 52 | 64 | 165 | Expressed protein, *Oryza* | | | CA206584 | UGSuM786 | (AG)6 | CDS | ATGATAACGGGTCCAACCA | AACGACAGCATCTCCCAC | 58 | 57 | 192 | retroelement, *Oryza* | | | CA209258 | UGSuM787 | (CT)6 | CDS | TCCTTCTCACCTACCTAACTTG | GGTTGCTTCATCAGTAATCAG | 55 | 54 | 375 | kelch repeat-containing F-box family protein, *Arabidopsis* | | | CA210236 | UGSuM788 | (GA)6 | CDS | GCAACAGCATTTCTTCCTATC | TGTGGTAATCGGGTGTATG | 56 | 55 | 279 | unknown protein, *Arabidopsis* | | | CA210322 | UGSuM789 | (CA)6 | CDS | ATCAGAGACAGCGTGGAG | CGTAGAACGGAAGCAAGA | 54 | 54 | 286 | thaumatin, *Nicotiana* | | | CA211805 | UGSuM790 | (CG)6 | CDS | CAGGAGCAGAGGAGACAC | AAATGGCACGATGAGGTAG | 53 | 56 | 281 | Tetratricopeptide-like helical, *Medicago* | | | CA221474 | UGSuM791 | (AT)6 | 3'UTRs | CGCACGCAACGCAAGCAC | ACTGGAAACCTGTGTATCATC | 68 | 54 | 228 | RuvA domain 2-like protein, *Arabidopsis* | | | CA223621 | UGSuM792 | (AC)6 | 3'UTRs | CACGACCATCACAACCTATT | GACTTCCACAACAGCAAGAC | 55 | 55 | 333 | Protease inhibitor/LTP family protein, Arabidopsis | | | CA235197 | UGSuM793 | (GA)6 | CDS | ATGCCTACACCCTCTTACAC | GTTCTGGAGCGATTCCCA | 54 | 59 | 331 | Expressed protein, *Oryza* | | | CA245990 | UGSuM794 | (AT)6 | 3'UTRs | ATCTGGTGGTGGTGGGAG | AGGGTTTGAGGTTTGATTG | 59 | 55 | 306 | 40S ribosomal protein S29 (RPS29A), *Arabidopsis* | | | CA252023 | UGSuM795 | (TA)6 | CDS | ATTCATAGGTTGAGGTTCTGG | ATAAACAAGACAGCATTTGGA | 55 | 54 | 160 | Heat shock factor protein HSF30, *Arabidopsis* | | | CA252563 | UGSuM796 | (CA)6 | CDS | ATTGAGGGAAAGGGAATG | GTCCTGGTGGTAACGAAG | 54 | 53 | 389 | lipid transfer protein, *Hordeum vulgare* | | | CA252945 | UGSuM797 | (CT)6 | CDS | CTCTCACCTTCTTCAACCC | ATAGATTGCCTCAGCCGT | 54 | 55 | 370 | hypothetical protein, *Oryza* | | | CA253276 | UGSuM798 | (TA)6 | CDS | CTCTCTCTCTCTCTCCCTCTG | TCACTTCTTCCTTCTTCTCCT | 55 | 54 | 348 | GTP-binding protein, *Triticum* | | | CA254328 | UGSuM799 | (CG)6 | CDS | CTTCCTTGGTCCTCTCCTAC | CGCTCCTCCATCATCACC | 55 | 60 | 101 | hypothetical protein, *Vitis vinifera* | | | CA261155 | UGSuM800 | (GA)6 | CDS | GATAGGCACTGAACAAGTCAA | AGACGGACAAGAGAGGTCA | 55 | 55 | 341 | tousled-like kinase 2, *Zea mays* | | | CA269306 | UGSuM801 | (CG)6 | CDS | CAGAGCAGCGTCGTCACC | GTAACATTCTTGAGGGTCCA | 61 | 55 | 279 | blue copper binding protein, *Oryza* | | | CA269905 | UGSuM802 | (TG)6 | CDS | CACATCTATTACAAACCGCAC | TGCTCAAGTTCATACAACAA | 55 | 52 | 306 | Protease inhibitor/seed storage/LTP family protein, *Pisum* | | | CA269916 | UGSuM803 | (CT)6 | CDS | GTCGTCTTCCTCTACTGGTTC | CGATTTCTTTCTTTCTTCTCC | 55 | 55 | 303 | Ser/Thr kinase, *Arabidopsis* | | | CA278861 | UGSuM804 | (GA)6 | CDS | CTCTCGCTTGCTTGTGTC | TAGTGGAAGGGTTCTGTTTG | 55 | 55 | 379 | mitochondrial peroxiredoxin, *Pisum sativum* | | | CA280822 | UGSuM805 | (AT)6 | CDS | GAGCACCTCACAGCATTT | CTCTCACGCCACCGCAAC | 54 | 64 | 299 | F-box family protein, *Arabidopsis* | | | CA283708 | UGSuM806 | (TG)6 | CDS | TAAGTGAAAGGAGCCAGAAA | GTCGTCGTGTCCAATAATAAC | 55 | 54 | 270 | 60S ribosomal protein, *Oryza* | | | CA284697 | UGSuM807 | (CT)6 | 5'UTRs | AGATGTCGTTGATGATTGATT | TGAAGAGAGAGGTTAGAGATGA | 54 | 53 | 307 | ATP-binding protein, *Arabidopsis* | | | CA288410 | UGSuM808 | (CG)6 | 5'UTRs | CAATCACATACCCTCACATTC | CGGTGTTCTACGGGCTGG | 55 | 62 | 310 | hypothetical protein, *Oryza* | | | CA289015 | UGSuM809 | (TC)6 | 5'UTRs | CTTTGGAATGGGCTGAGT | CTGGGAGACAATAGGTTTCA | 56 | 55 | 383 | unknown protein, *Arabidopsis* | | | CA295721 | UGSuM810 | (AT)6 | 5'UTRs | TGACACAGAACAAACAAAGTC | AGTGAGGAGGTGGCGATG | 53 | 59 | 295 | Acyltransferase family protein, *Oryza* | | |  |  |  |  |  |  |  |  |  |  |
| --- | --- | --- | --- | --- | --- | --- | --- | --- | --- | --- | --- | --- | --- | --- | --- | --- | --- | --- | --- | --- | --- | --- | --- | --- | --- | --- | --- | --- | --- | --- | --- | --- | --- | --- | --- | --- | --- | --- | --- | --- | --- | --- | --- | --- | --- | --- | --- | --- | --- | --- | --- | --- | --- | --- | --- | --- | --- | --- | --- | --- | --- | --- | --- | --- | --- | --- | --- | --- | --- | --- | --- | --- | --- | --- | --- | --- | --- | --- | --- | --- | --- | --- | --- | --- | --- | --- | --- | --- | --- | --- | --- | --- | --- | --- | --- | --- | --- | --- | --- | --- | --- | --- | --- | --- | --- | --- | --- | --- | --- | --- | --- | --- | --- | --- | --- | --- | --- | --- | --- | --- | --- | --- | --- | --- | --- | --- | --- | --- | --- | --- | --- | --- | --- | --- | --- | --- | --- | --- | --- | --- | --- | --- | --- | --- | --- | --- | --- | --- | --- | --- | --- | --- | --- | --- | --- | --- | --- | --- | --- | --- | --- | --- | --- | --- | --- | --- | --- | --- | --- | --- | --- | --- | --- | --- | --- | --- | --- | --- | --- | --- | --- | --- | --- | --- | --- | --- | --- | --- | --- | --- | --- | --- | --- | --- | --- | --- | --- | --- | --- | --- | --- | --- | --- | --- | --- | --- | --- | --- | --- | --- | --- | --- | --- | --- | --- | --- | --- | --- | --- | --- | --- | --- | --- | --- | --- | --- | --- | --- | --- | --- | --- | --- | --- | --- | --- | --- | --- | --- | --- | --- | --- | --- | --- | --- | --- | --- | --- | --- | --- | --- | --- | --- | --- | --- | --- | --- | --- | --- | --- | --- | --- | --- | --- | --- | --- | --- | --- | --- | --- | --- | --- | --- | --- | --- | --- | --- | --- | --- | --- | --- | --- | --- | --- | --- | --- | --- | --- | --- | --- | --- | --- | --- | --- | --- | --- | --- | --- | --- | --- | --- | --- | --- | --- | --- | --- | --- | --- | --- | --- | --- | --- | --- | --- | --- | --- | --- | --- | --- | --- | --- | --- | --- | --- | --- | --- | --- | --- | --- | --- | --- | --- | --- | --- | --- | --- | --- | --- | --- | --- | --- | --- | --- | --- | --- | --- | --- | --- | --- | --- | --- | --- | --- | --- | --- | --- | --- | --- | --- | --- | --- | --- | --- | --- | --- | --- | --- | --- | --- | --- | --- | --- | --- | --- | --- | --- | --- | --- | --- | --- | --- | --- | --- | --- | --- | --- | --- | --- | --- | --- | --- | --- | --- | --- | --- | --- | --- | --- | --- | --- | --- | --- | --- | --- | --- | --- | --- | --- | --- | --- | --- | --- | --- | --- | --- | --- | --- | --- | --- | --- | --- | --- | --- | --- | --- | --- | --- | --- | --- | --- | --- | --- | --- | --- | --- | --- | --- | --- | --- | --- | --- | --- | --- | --- | --- | --- | --- | --- | --- | --- | --- | --- | --- | --- | --- | --- | --- | --- | --- | --- | --- | --- | --- | --- | --- | --- | --- | --- | --- | --- | --- | --- | --- | --- | --- | --- | --- | --- | --- | --- | --- | --- | --- | --- | --- | --- | --- | --- | --- | --- | --- | --- | --- | --- | --- | --- | --- | --- | --- | --- | --- | --- | --- | --- | --- | --- | --- | --- | --- | --- | --- | --- | --- | --- | --- | --- | --- | --- | --- | --- | --- | --- | --- | --- | --- | --- | --- | --- | --- | --- | --- | --- | --- | --- | --- | --- | --- | --- | --- | --- | --- | --- | --- | --- | --- | --- | --- | --- | --- | --- | --- | --- | --- | --- | --- | --- | --- | --- | --- | --- | --- | --- | --- | --- | --- | --- | --- | --- | --- | --- | --- | --- | --- | --- | --- | --- | --- | --- | --- | --- | --- | --- | --- | --- | --- | --- | --- | --- | --- | --- | --- | --- | --- | --- | --- | --- | --- | --- | --- | --- | --- | --- | --- | --- | --- | --- | --- | --- | --- | --- | --- | --- | --- | --- | --- | --- | --- | --- | --- | --- | --- | --- | --- | --- | --- | --- | --- | --- | --- | --- | --- | --- | --- | --- | --- | --- | --- | --- | --- | --- | --- | --- | --- | --- | --- | --- | --- | --- | --- | --- | --- | --- | --- | --- | --- | --- | --- | --- | --- | --- | --- | --- | --- | --- | --- | --- | --- | --- | --- | --- | --- | --- | --- | --- | --- | --- | --- | --- | --- | --- | --- | --- | --- | --- | --- | --- | --- | --- | --- | --- | --- | --- | --- | --- | --- | --- | --- | --- | --- | --- | --- | --- | --- | --- | --- | --- | --- | --- | --- | --- | --- | --- | --- | --- | --- | --- | --- | --- | --- | --- | --- | --- | --- | --- | --- | --- | --- | --- | --- | --- | --- | --- | --- | --- | --- | --- | --- | --- | --- | --- | --- | --- | --- | --- | --- | --- | --- | --- | --- | --- | --- | --- | --- | --- | --- | --- | --- | --- | --- | --- | --- | --- | --- | --- | --- | --- | --- | --- | --- | --- | --- | --- | --- | --- | --- | --- | --- | --- | --- | --- | --- | --- | --- | --- | --- | --- | --- | --- | --- | --- | --- | --- | --- | --- | --- | --- | --- | --- | --- | --- | --- | --- | --- | --- | --- | --- | --- | --- | --- | --- | --- | --- | --- | --- | --- | --- | --- | --- | --- | --- | --- | --- | --- | --- | --- | --- | --- | --- | --- | --- | --- | --- | --- | --- | --- | --- | --- | --- | --- | --- | --- | --- | --- | --- | --- | --- | --- | --- | --- | --- | --- | --- | --- | --- | --- | --- | --- | --- | --- | --- | --- | --- | --- | --- | --- | --- | --- | --- | --- | --- | --- | --- | --- | --- | --- | --- | --- | --- | --- | --- | --- | --- | --- | --- | --- | --- | --- | --- | --- | --- | --- | --- | --- | --- | --- | --- | --- | --- | --- | --- | --- | --- | --- | --- | --- | --- | --- | --- | --- | --- | --- | --- | --- | --- | --- | --- | --- | --- | --- | --- | --- | --- | --- | --- | --- | --- | --- | --- | --- | --- | --- | --- | --- | --- | --- | --- | --- | --- | --- | --- | --- | --- | --- | --- | --- | --- | --- | --- | --- | --- | --- | --- | --- | --- | --- | --- | --- | --- | --- | --- | --- | --- | --- | --- | --- | --- | --- | --- | --- | --- | --- | --- | --- | --- | --- | --- | --- | --- | --- | --- | --- | --- | --- | --- | --- | --- | --- | --- | --- | --- | --- | --- | --- | --- | --- | --- | --- | --- | --- | --- | --- | --- | --- | --- | --- | --- | --- | --- | --- | --- | --- | --- | --- | --- | --- | --- | --- | --- | --- | --- | --- | --- | --- | --- | --- | --- | --- | --- | --- | --- | --- | --- | --- | --- | --- | --- | --- | --- | --- | --- | --- | --- | --- | --- | --- | --- | --- | --- | --- | --- | --- | --- | --- | --- | --- | --- | --- | --- | --- | --- | --- | --- | --- | --- | --- | --- | --- | --- | --- | --- | --- | --- | --- | --- | --- | --- | --- | --- | --- | --- | --- | --- | --- | --- | --- | --- | --- | --- | --- | --- | --- | --- | --- | --- | --- | --- | --- | --- | --- | --- | --- | --- | --- | --- | --- | --- | --- | --- | --- | --- | --- | --- | --- | --- | --- | --- | --- | --- | --- | --- | --- | --- | --- | --- | --- | --- | --- | --- | --- | --- | --- | --- | --- | --- | --- | --- | --- | --- | --- | --- | --- | --- | --- | --- | --- | --- | --- | --- | --- | --- | --- | --- | --- | --- | --- | --- | --- | --- | --- | --- | --- | --- | --- | --- | --- | --- | --- | --- | --- | --- | --- | --- | --- | --- | --- | --- | --- | --- | --- | --- | --- | --- | --- | --- | --- | --- | --- | --- | --- | --- | --- | --- | --- | --- | --- | --- | --- | --- | --- | --- | --- | --- | --- | --- | --- | --- | --- | --- | --- | --- | --- | --- | --- | --- | --- | --- | --- | --- | --- | --- | --- | --- | --- | --- | --- | --- | --- | --- | --- | --- | --- | --- | --- | --- | --- | --- | --- | --- | --- | --- | --- | --- | --- | --- | --- | --- | --- | --- | --- | --- | --- | --- | --- | --- | --- | --- | --- | --- | --- | --- | --- | --- | --- | --- | --- | --- | --- | --- | --- | --- | --- | --- | --- | --- | --- | --- | --- | --- | --- | --- | --- | --- | --- | --- | --- | --- | --- | --- | --- | --- | --- | --- | --- | --- | --- | --- | --- | --- | --- | --- | --- | --- | --- | --- | --- | --- | --- | --- | --- | --- | --- | --- | --- | --- | --- | --- | --- | --- | --- | --- | --- | --- | --- | --- | --- | --- | --- | --- | --- | --- | --- | --- | --- | --- | --- | --- | --- | --- | --- | --- | --- | --- | --- | --- | --- | --- | --- | --- | --- | --- | --- | --- | --- | --- | --- | --- | --- | --- | --- | --- | --- | --- | --- | --- | --- | --- | --- | --- | --- | --- | --- | --- | --- | --- | --- | --- | --- | --- | --- | --- | --- | --- | --- | --- | --- | --- | --- | --- | --- | --- | --- | --- | --- | --- | --- | --- | --- | --- | --- | --- | --- | --- | --- | --- | --- | --- | --- | --- | --- | --- | --- | --- | --- | --- | --- | --- | --- | --- | --- | --- | --- | --- | --- | --- | --- | --- | --- | --- | --- | --- | --- | --- | --- | --- | --- | --- | --- | --- | --- | --- | --- | --- | --- | --- | --- | --- | --- | --- | --- | --- | --- | --- | --- | --- | --- | --- | --- | --- | --- | --- | --- | --- | --- | --- | --- | --- | --- | --- | --- | --- | --- | --- | --- | --- | --- | --- | --- | --- | --- | --- | --- | --- | --- | --- | --- | --- | --- | --- | --- | --- | --- | --- | --- | --- | --- | --- | --- | --- | --- | --- | --- | --- | --- | --- | --- | --- | --- | --- | --- | --- | --- | --- | --- | --- | --- | --- | --- | --- | --- | --- | --- | --- | --- | --- | --- | --- | --- | --- | --- | --- | --- | --- | --- | --- | --- | --- | --- | --- | --- | --- | --- | --- | --- | --- | --- | --- | --- | --- | --- | --- | --- | --- | --- | --- | --- | --- | --- | --- | --- | --- | --- | --- | --- | --- | --- | --- | --- | --- | --- | --- | --- | --- | --- | --- | --- | --- | --- | --- | --- | --- | --- | --- | --- | --- | --- | --- | --- | --- | --- | --- | --- | --- | --- | --- | --- | --- | --- | --- | --- | --- | --- | --- | --- | --- | --- | --- | --- | --- | --- | --- | --- | --- | --- | --- | --- | --- | --- | --- | --- | --- | --- | --- | --- | --- | --- | --- | --- | --- | --- | --- | --- | --- | --- | --- | --- | --- | --- | --- | --- | --- | --- | --- | --- | --- | --- | --- | --- | --- | --- | --- | --- | --- | --- | --- | --- | --- | --- | --- | --- | --- | --- | --- | --- | --- | --- | --- | --- | --- | --- | --- | --- | --- | --- | --- | --- | --- | --- | --- | --- | --- | --- | --- | --- | --- | --- | --- | --- | --- | --- | --- | --- | --- | --- | --- | --- | --- | --- | --- | --- | --- | --- | --- | --- | --- | --- | --- | --- | --- | --- | --- | --- | --- | --- | --- | --- | --- | --- | --- | --- | --- | --- | --- | --- | --- | --- | --- | --- | --- | --- | --- | --- | --- | --- | --- | --- | --- | --- | --- | --- | --- | --- | --- | --- | --- | --- | --- | --- | --- | --- | --- | --- | --- | --- | --- | --- | --- | --- | --- | --- | --- | --- | --- | --- | --- | --- | --- | --- | --- | --- | --- | --- | --- | --- | --- | --- | --- | --- | --- | --- | --- | --- | --- | --- | --- | --- | --- | --- | --- | --- | --- | --- | --- | --- | --- | --- | --- | --- | --- | --- | --- | --- | --- | --- | --- | --- | --- | --- | --- | --- | --- | --- | --- | --- | --- | --- | --- | --- | --- | --- | --- | --- | --- | --- | --- | --- | --- | --- | --- | --- | --- | --- | --- | --- | --- | --- | --- | --- | --- | --- | --- | --- | --- | --- | --- | --- | --- | --- | --- | --- | --- | --- | --- | --- | --- | --- | --- | --- | --- | --- | --- | --- | --- | --- | --- | --- | --- | --- | --- | --- | --- | --- | --- | --- | --- | --- | --- | --- | --- | --- | --- | --- | --- | --- | --- | --- | --- | --- | --- | --- | --- | --- | --- | --- | --- | --- | --- | --- | --- | --- | --- | --- | --- | --- | --- | --- | --- | --- | --- | --- | --- | --- | --- | --- | --- | --- | --- | --- | --- | --- | --- | --- | --- | --- | --- | --- | --- | --- | --- | --- | --- | --- | --- | --- | --- | --- | --- | --- | --- | --- | --- | --- | --- | --- | --- | --- | --- | --- | --- | --- | --- | --- | --- | --- | --- | --- | --- | --- | --- | --- | --- | --- | --- | --- | --- | --- | --- | --- | --- | --- | --- | --- | --- | --- | --- | --- | --- | --- | --- | --- | --- | --- | --- | --- | --- | --- | --- | --- | --- | --- | --- | --- | --- | --- | --- | --- | --- | --- | --- | --- | --- | --- | --- | --- | --- | --- | --- | --- | --- | --- | --- | --- | --- | --- | --- | --- | --- | --- | --- | --- | --- | --- | --- | --- | --- | --- | --- | --- | --- | --- | --- | --- | --- | --- | --- | --- | --- | --- | --- | --- | --- | --- | --- | --- | --- | --- | --- | --- | --- | --- | --- | --- | --- | --- | --- | --- | --- | --- | --- | --- | --- | --- | --- | --- | --- | --- | --- | --- | --- | --- | --- | --- | --- | --- | --- | --- | --- | --- | --- | --- | --- | --- | --- | --- | --- | --- | --- | --- | --- | --- | --- | --- | --- | --- | --- | --- | --- | --- | --- | --- | --- | --- | --- | --- | --- | --- | --- | --- | --- | --- | --- | --- | --- | --- | --- | --- | --- | --- | --- | --- | --- | --- | --- | --- | --- | --- | --- | --- | --- | --- | --- | --- | --- | --- | --- | --- | --- | --- | --- | --- | --- | --- | --- | --- | --- | --- | --- | --- | --- | --- | --- | --- | --- | --- | --- | --- | --- | --- | --- | --- | --- | --- | --- | --- | --- | --- | --- | --- | --- | --- | --- | --- | --- | --- | --- | --- | --- | --- | --- | --- | --- | --- | --- | --- | --- | --- | --- | --- | --- | --- | --- | --- | --- | --- | --- | --- | --- | --- | --- | --- | --- | --- | --- | --- | --- | --- | --- | --- | --- | --- | --- | --- | --- | --- | --- | --- | --- | --- | --- | --- | --- | --- | --- | --- | --- | --- | --- | --- | --- | --- | --- | --- | --- | --- | --- | --- | --- | --- | --- | --- | --- | --- | --- | --- | --- | --- | --- | --- | --- | --- | --- | --- | --- | --- | --- | --- | --- | --- | --- | --- | --- | --- | --- | --- | --- | --- | --- | --- | --- | --- | --- | --- | --- | --- | --- | --- | --- | --- | --- | --- | --- | --- | --- | --- | --- | --- | --- | --- | --- | --- | --- | --- | --- | --- | --- | --- | --- | --- | --- | --- | --- | --- | --- | --- | --- | --- | --- | --- | --- | --- | --- | --- | --- | --- | --- | --- | --- | --- | --- | --- | --- | --- | --- | --- | --- | --- | --- | --- | --- | --- | --- | --- | --- | --- | --- | --- | --- | --- | --- | --- | --- | --- | --- | --- | --- | --- | --- | --- | --- | --- | --- | --- | --- | --- | --- | --- | --- | --- | --- | --- | --- | --- | --- | --- | --- | --- | --- | --- | --- | --- | --- | --- | --- | --- | --- | --- | --- | --- | --- | --- | --- | --- | --- | --- | --- | --- | --- | --- | --- | --- | --- | --- | --- | --- | --- | --- | --- | --- | --- | --- | --- | --- | --- | --- | --- | --- | --- | --- | --- | --- | --- | --- | --- | --- | --- | --- | --- | --- | --- | --- | --- | --- | --- | --- | --- | --- | --- | --- | --- | --- | --- | --- | --- | --- | --- | --- | --- | --- | --- | --- | --- | --- | --- | --- | --- | --- | --- | --- | --- | --- | --- | --- | --- | --- | --- | --- | --- | --- | --- | --- | --- | --- | --- | --- | --- | --- | --- | --- | --- | --- | --- | --- | --- | --- | --- | --- | --- | --- | --- | --- | --- | --- | --- | --- | --- | --- | --- | --- | --- | --- | --- | --- | --- | --- | --- | --- | --- | --- | --- | --- | --- | --- | --- | --- | --- | --- | --- | --- | --- | --- | --- | --- | --- | --- | --- | --- | --- | --- | --- | --- | --- | --- | --- | --- | --- | --- | --- | --- | --- | --- | --- | --- | --- | --- | --- | --- | --- | --- | --- | --- | --- | --- | --- | --- | --- | --- | --- | --- | --- | --- | --- | --- | --- | --- | --- | --- | --- | --- | --- | --- | --- | --- | --- | --- | --- | --- | --- | --- | --- | --- | --- | --- | --- | --- | --- | --- | --- | --- | --- | --- | --- | --- | --- | --- | --- | --- | --- | --- | --- | --- | --- | --- | --- | --- | --- | --- | --- | --- | --- | --- | --- | --- | --- | --- | --- | --- | --- | --- | --- | --- | --- | --- | --- | --- | --- | --- | --- | --- | --- | --- | --- | --- | --- | --- | --- | --- | --- | --- | --- | --- | --- | --- | --- | --- | --- | --- | --- | --- | --- | --- | --- | --- | --- | --- | --- | --- | --- | --- | --- | --- | --- | --- | --- | --- | --- | --- | --- | --- | --- | --- | --- | --- | --- | --- | --- | --- | --- | --- | --- | --- | --- | --- | --- | --- | --- | --- | --- | --- | --- | --- | --- | --- | --- | --- | --- | --- | --- | --- | --- | --- | --- | --- | --- | --- | --- | --- | --- | --- | --- | --- | --- | --- | --- | --- | --- | --- | --- | --- | --- | --- | --- | --- | --- | --- | --- | --- | --- | --- | --- | --- | --- | --- | --- | --- | --- | --- | --- | --- | --- | --- | --- | --- | --- | --- | --- | --- | --- | --- | --- | --- | --- | --- | --- | --- | --- | --- | --- | --- | --- | --- | --- | --- | --- | --- | --- | --- | --- | --- | --- | --- | --- | --- | --- | --- | --- | --- | --- | --- | --- | --- | --- | --- | --- | --- | --- | --- | --- | --- | --- | --- | --- | --- | --- | --- | --- | --- | --- | --- | --- | --- | --- | --- | --- | --- | --- | --- | --- | --- | --- | --- | --- | --- | --- | --- | --- | --- | --- | --- | --- | --- | --- | --- | --- | --- | --- | --- | --- | --- | --- | --- | --- | --- | --- | --- | --- | --- | --- | --- | --- | --- | --- | --- | --- | --- | --- | --- | --- | --- | --- | --- | --- | --- | --- | --- | --- | --- | --- | --- | --- | --- | --- | --- | --- | --- | --- | --- | --- | --- | --- | --- | --- | --- | --- | --- | --- | --- | --- | --- | --- | --- | --- | --- | --- | --- | --- | --- | --- | --- | --- | --- | --- | --- | --- | --- | --- | --- | --- | --- | --- | --- | --- | --- | --- | --- | --- | --- | --- | --- | --- | --- | --- | --- | --- | --- | --- | --- | --- | --- | --- | --- | --- | --- | --- | --- | --- | --- | --- | --- | --- | --- | --- | --- | --- | --- | --- | --- | --- | --- | --- | --- | --- | --- | --- | --- | --- | --- | --- | --- | --- | --- | --- | --- | --- | --- | --- | --- | --- | --- | --- | --- | --- | --- | --- | --- | --- | --- | --- | --- | --- | --- | --- | --- | --- | --- | --- | --- | --- | --- | --- | --- | --- | --- | --- | --- | --- | --- | --- | --- | --- | --- | --- | --- | --- | --- | --- | --- | --- | --- | --- | --- | --- | --- | --- | --- | --- | --- | --- | --- | --- | --- | --- | --- | --- | --- | --- | --- | --- | --- | --- | --- | --- | --- | --- | --- | --- | --- | --- | --- | --- | --- | --- | --- | --- | --- | --- | --- | --- | --- | --- | --- | --- | --- | --- | --- | --- | --- | --- | --- | --- | --- | --- | --- | --- | --- | --- | --- | --- | --- | --- | --- | --- | --- | --- | --- | --- | --- | --- | --- | --- | --- | --- | --- | --- | --- | --- | --- | --- | --- | --- | --- | --- | --- | --- | --- | --- | --- | --- | --- | --- | --- | --- | --- | --- | --- | --- | --- | --- | --- | --- | --- | --- | --- | --- | --- | --- | --- | --- | --- | --- | --- | --- | --- | --- | --- | --- | --- | --- | --- | --- | --- | --- | --- | --- | --- | --- | --- | --- | --- | --- | --- | --- | --- | --- | --- | --- | --- | --- | --- | --- | --- | --- | --- | --- | --- | --- | --- | --- | --- | --- | --- | --- | --- | --- | --- | --- | --- | --- | --- | --- | --- | --- | --- | --- | --- | --- | --- | --- | --- | --- | --- | --- | --- | --- | --- | --- | --- | --- | --- | --- | --- | --- | --- | --- | --- | --- | --- | --- | --- | --- | --- | --- | --- | --- | --- | --- | --- | --- | --- | --- | --- | --- | --- | --- | --- | --- | --- | --- | --- | --- | --- | --- | --- | --- | --- | --- | --- | --- | --- | --- | --- | --- | --- | --- | --- | --- | --- | --- | --- | --- | --- | --- | --- | --- | --- | --- | --- | --- | --- | --- | --- | --- | --- | --- | --- | --- | --- | --- | --- | --- | --- | --- | --- | --- | --- | --- | --- | --- | --- | --- | --- | --- | --- | --- | --- | --- | --- | --- | --- | --- | --- | --- | --- | --- | --- | --- | --- | --- | --- | --- | --- | --- | --- | --- | --- | --- | --- | --- | --- | --- | --- | --- | --- | --- | --- | --- | --- | --- | --- | --- | --- | --- | --- | --- | --- | --- | --- | --- | --- | --- | --- | --- | --- | --- | --- | --- | --- | --- | --- | --- | --- | --- | --- | --- | --- | --- | --- | --- | --- | --- | --- | --- | --- | --- | --- | --- | --- | --- | --- | --- | --- | --- | --- | --- | --- | --- | --- | --- | --- | --- | --- | --- | --- | --- | --- | --- | --- | --- | --- | --- | --- | --- | --- | --- | --- | --- | --- | --- | --- | --- | --- | --- | --- | --- | --- | --- | --- | --- | --- | --- | --- | --- | --- | --- | --- | --- | --- | --- | --- | --- | --- | --- | --- | --- | --- | --- | --- | --- | --- | --- | --- | --- | --- | --- | --- | --- | --- | --- | --- | --- | --- | --- | --- | --- | --- | --- | --- | --- | --- | --- | --- | --- | --- | --- | --- | --- | --- | --- | --- | --- | --- | --- | --- | --- | --- | --- | --- | --- | --- | --- | --- | --- | --- | --- | --- | --- | --- | --- | --- | --- | --- | --- | --- | --- | --- | --- | --- | --- | --- | --- | --- | --- | --- | --- | --- | --- | --- | --- | --- | --- | --- | --- | --- | --- | --- | --- | --- | --- | --- | --- | --- | --- | --- | --- | --- | --- | --- | --- | --- | --- | --- | --- | --- | --- | --- | --- | --- | --- | --- | --- | --- | --- | --- | --- | --- | --- | --- | --- | --- | --- | --- | --- | --- | --- | --- | --- | --- | --- | --- | --- | --- | --- | --- | --- | --- | --- | --- | --- | --- | --- | --- | --- | --- | --- | --- | --- | --- | --- | --- | --- | --- | --- | --- | --- | --- | --- | --- | --- | --- | --- | --- | --- | --- | --- | --- | --- | --- | --- | --- | --- | --- | --- | --- | --- | --- | --- | --- | --- | --- | --- | --- | --- | --- | --- | --- | --- | --- | --- | --- | --- | --- | --- | --- | --- | --- | --- | --- | --- | --- | --- | --- | --- | --- | --- | --- | --- | --- | --- | --- | --- | --- | --- | --- | --- | --- | --- | --- | --- | --- | --- | --- | --- | --- | --- | --- | --- | --- | --- | --- | --- | --- | --- | --- | --- | --- | --- | --- | --- | --- | --- | --- | --- | --- | --- | --- | --- | --- | --- | --- | --- | --- | --- | --- | --- | --- | --- | --- | --- | --- | --- | --- | --- | --- | --- | --- | --- | --- | --- | --- | --- | --- | --- | --- | --- | --- | --- | --- | --- | --- | --- | --- | --- | --- | --- | --- | --- | --- | --- | --- | --- | --- | --- | --- | --- | --- | --- | --- | --- | --- | --- | --- | --- | --- | --- | --- | --- | --- | --- | --- | --- | --- | --- | --- | --- | --- | --- | --- | --- | --- | --- | --- | --- | --- | --- | --- | --- | --- | --- | --- | --- | --- | --- | --- | --- | --- | --- | --- | --- | --- | --- | --- | --- | --- | --- | --- | --- | --- | --- | --- | --- | --- | --- | --- | --- | --- | --- | --- | --- | --- | --- | --- | --- | --- | --- | --- | --- | --- | --- | --- | --- | --- | --- | --- | --- | --- | --- | --- | --- | --- | --- | --- | --- | --- | --- | --- | --- | --- | --- | --- | --- | --- | --- | --- | --- | --- | --- | --- | --- | --- | --- | --- | --- | --- | --- | --- | --- | --- | --- | --- | --- | --- | --- | --- | --- | --- | --- | --- | --- | --- | --- | --- | --- | --- | --- | --- | --- | --- | --- | --- | --- | --- | --- | --- | --- | --- | --- | --- | --- | --- | --- | --- | --- | --- | --- | --- | --- | --- | --- | --- | --- | --- | --- | --- | --- | --- | --- | --- | --- | --- | --- | --- | --- | --- | --- | --- | --- | --- | --- | --- | --- | --- | --- | --- | --- | --- | --- | --- | --- | --- | --- | --- | --- | --- | --- | --- | --- | --- | --- | --- | --- | --- | --- | --- | --- | --- | --- | --- | --- | --- | --- | --- | --- | --- | --- | --- | --- | --- | --- | --- | --- | --- | --- | --- | --- | --- | --- | --- | --- | --- | --- | --- | --- | --- | --- | --- | --- | --- | --- | --- | --- | --- | --- | --- | --- | --- | --- | --- | --- | --- | --- | --- | --- | --- | --- | --- | --- | --- | --- | --- | --- | --- | --- | --- | --- | --- | --- | --- | --- | --- | --- | --- | --- | --- | --- | --- | --- | --- | --- | --- | --- | --- | --- | --- | --- | --- | --- | --- | --- | --- | --- | --- | --- | --- | --- | --- | --- | --- | --- | --- | --- | --- | --- | --- | --- | --- | --- | --- | --- | --- | --- | --- | --- | --- | --- | --- | --- | --- | --- | --- | --- | --- | --- | --- | --- | --- | --- | --- | --- | --- | --- | --- | --- | --- | --- | --- | --- | --- | --- | --- | --- | --- | --- | --- | --- | --- | --- | --- | --- | --- | --- | --- | --- | --- | --- | --- | --- | --- | --- | --- | --- | --- | --- | --- | --- | --- | --- | --- | --- | --- | --- | --- | --- | --- | --- | --- | --- | --- | --- | --- | --- | --- | --- | --- | --- | --- | --- | --- | --- | --- | --- | --- | --- | --- | --- | --- | --- | --- | --- | --- | --- | --- | --- | --- | --- | --- | --- | --- | --- | --- | --- | --- | --- | --- | --- | --- | --- | --- | --- | --- | --- | --- | --- | --- | --- | --- | --- | --- | --- | --- | --- | --- | --- | --- | --- | --- | --- | --- | --- | --- | --- | --- | --- | --- | --- | --- | --- | --- | --- | --- | --- | --- | --- | --- | --- | --- | --- | --- | --- | --- | --- | --- | --- | --- | --- | --- | --- | --- | --- | --- | --- | --- | --- | --- | --- | --- | --- | --- | --- | --- | --- | --- | --- | --- | --- | --- | --- | --- | --- | --- | --- | --- | --- | --- | --- | --- | --- | --- | --- | --- | --- | --- | --- | --- | --- | --- | --- | --- | --- | --- | --- | --- | --- | --- | --- | --- | --- | --- | --- | --- | --- | --- | --- | --- | --- | --- | --- | --- | --- | --- | --- | --- | --- | --- | --- | --- | --- | --- | --- | --- | --- | --- | --- | --- | --- | --- | --- | --- | --- | --- | --- | --- | --- | --- | --- | --- | --- | --- | --- | --- | --- | --- | --- | --- | --- | --- | --- | --- | --- | --- | --- | --- | --- | --- | --- | --- | --- | --- | --- | --- | --- | --- | --- | --- | --- | --- | --- | --- | --- | --- | --- | --- | --- | --- | --- | --- | --- | --- | --- | --- | --- | --- | --- | --- | --- | --- | --- | --- | --- | --- | --- | --- | --- | --- | --- | --- | --- | --- | --- | --- | --- | --- | --- | --- | --- | --- | --- | --- | --- | --- | --- | --- | --- | --- | --- | --- | --- | --- | --- | --- | --- | --- | --- | --- | --- | --- | --- | --- | --- | --- | --- | --- | --- | --- | --- | --- | --- | --- | --- | --- | --- | --- | --- | --- | --- | --- | --- | --- | --- | --- | --- | --- | --- | --- | --- | --- | --- | --- | --- | --- | --- | --- | --- | --- | --- | --- | --- | --- | --- | --- | --- | --- | --- | --- | --- | --- | --- | --- | --- | --- | --- | --- | --- | --- | --- | --- | --- | --- | --- | --- | --- | --- | --- | --- | --- | --- | --- | --- | --- | --- | --- | --- | --- | --- | --- | --- | --- | --- | --- | --- | --- | --- | --- | --- | --- | --- | --- | --- | --- | --- | --- | --- | --- | --- | --- | --- | --- | --- | --- | --- | --- | --- | --- | --- | --- | --- | --- | --- | --- | --- | --- | --- | --- | --- | --- | --- | --- | --- | --- | --- | --- | --- | --- | --- | --- | --- | --- | --- | --- | --- | --- | --- | --- | --- | --- | --- | --- | --- | --- | --- | --- | --- | --- | --- | --- | --- | --- | --- | --- | --- | --- | --- | --- | --- | --- | --- | --- | --- | --- | --- | --- | --- | --- | --- | --- | --- | --- | --- | --- | --- | --- | --- | --- | --- | --- | --- | --- | --- | --- | --- | --- | --- | --- | --- | --- | --- | --- | --- | --- | --- | --- | --- | --- | --- | --- | --- | --- | --- | --- | --- | --- | --- | --- | --- | --- | --- | --- | --- | --- | --- | --- | --- | --- | --- | --- | --- | --- | --- | --- | --- | --- | --- | --- | --- | --- | --- | --- | --- | --- | --- | --- | --- | --- | --- | --- | --- | --- | --- | --- | --- | --- | --- | --- | --- | --- | --- | --- | --- | --- | --- | --- | --- | --- | --- | --- | --- | --- | --- | --- | --- | --- | --- | --- | --- | --- | --- | --- | --- | --- | --- | --- | --- | --- | --- | --- | --- | --- | --- | --- | --- | --- | --- | --- | --- | --- | --- | --- | --- | --- | --- | --- | --- | --- | --- | --- | --- | --- | --- | --- | --- | --- | --- | --- | --- | --- | --- | --- | --- | --- | --- | --- | --- | --- | --- | --- | --- | --- | --- | --- | --- | --- | --- | --- | --- | --- | --- | --- | --- | --- | --- | --- | --- | --- | --- | --- | --- | --- | --- | --- | --- | --- | --- | --- | --- | --- | --- | --- | --- | --- | --- | --- | --- | --- | --- | --- | --- | --- | --- | --- | --- | --- | --- | --- | --- | --- | --- | --- | --- | --- | --- | --- | --- | --- | --- | --- | --- | --- | --- | --- | --- | --- | --- | --- | --- | --- | --- | --- | --- | --- | --- | --- | --- | --- | --- | --- | --- | --- | --- | --- | --- | --- | --- | --- | --- | --- | --- | --- | --- | --- | --- | --- | --- | --- | --- | --- | --- | --- | --- | --- | --- | --- | --- | --- | --- | --- | --- | --- | --- | --- | --- | --- | --- | --- | --- | --- | --- | --- | --- | --- | --- | --- | --- | --- | --- | --- | --- | --- | --- | --- | --- | --- | --- | --- | --- | --- | --- | --- | --- | --- | --- | --- | --- | --- | --- | --- | --- | --- | --- | --- | --- | --- | --- | --- | --- | --- | --- | --- | --- | --- | --- | --- | --- | --- | --- | --- | --- | --- | --- | --- | --- | --- | --- | --- | --- | --- | --- | --- | --- | --- | --- | --- | --- | --- | --- | --- | --- | --- | --- | --- | --- | --- | --- | --- | --- | --- | --- | --- | --- | --- | --- | --- | --- | --- | --- | --- | --- | --- | --- | --- | --- | --- | --- | --- | --- | --- | --- | --- | --- | --- | --- | --- | --- | --- | --- | --- | --- | --- | --- | --- | --- | --- | --- | --- | --- | --- | --- | --- | --- | --- | --- | --- | --- | --- | --- | --- | --- | --- | --- | --- | --- | --- | --- | --- | --- | --- | --- | --- | --- | --- | --- | --- | --- | --- | --- | --- | --- | --- | --- | --- | --- | --- | --- | --- | --- | --- | --- | --- | --- | --- | --- | --- | --- | --- | --- | --- | --- | --- | --- | --- | --- | --- | --- | --- | --- | --- | --- | --- | --- | --- | --- | --- | --- | --- | --- | --- | --- | --- | --- | --- | --- | --- | --- | --- | --- | --- | --- | --- | --- | --- | --- | --- | --- | --- | --- | --- | --- | --- | --- | --- | --- | --- | --- | --- | --- | --- | --- | --- | --- | --- | --- | --- | --- | --- | --- | --- | --- | --- | --- | --- | --- | --- | --- | --- | --- | --- | --- | --- | --- | --- | --- | --- | --- | --- | --- | --- | --- | --- | --- | --- | --- | --- | --- | --- | --- | --- | --- | --- | --- | --- | --- | --- | --- | --- | --- | --- | --- | --- | --- | --- | --- | --- | --- | --- | --- | --- | --- | --- | --- | --- | --- | --- | --- | --- | --- | --- | --- | --- | --- | --- | --- | --- | --- | --- | --- | --- | --- | --- | --- | --- | --- | --- | --- | --- | --- | --- | --- | --- | --- | --- | --- | --- | --- | --- | --- | --- | --- | --- | --- | --- | --- | --- | --- | --- | --- | --- | --- | --- | --- | --- | --- | --- | --- | --- | --- | --- | --- | --- | --- | --- | --- | --- | --- | --- | --- | --- | --- | --- | --- | --- | --- | --- | --- | --- | --- | --- | --- | --- | --- | --- | --- | --- | --- | --- | --- | --- | --- | --- | --- | --- | --- | --- | --- | --- | --- | --- | --- | --- | --- | --- | --- | --- | --- | --- | --- | --- | --- | --- | --- | --- | --- | --- | --- | --- | --- | --- | --- | --- | --- | --- | --- | --- | --- | --- | --- | --- | --- | --- | --- | --- | --- | --- | --- | --- | --- | --- | --- | --- | --- | --- | --- | --- | --- | --- | --- | --- | --- | --- | --- | --- | --- | --- | --- | --- | --- | --- | --- | --- | --- | --- | --- | --- | --- | --- | --- | --- | --- | --- | --- | --- | --- | --- | --- | --- | --- | --- | --- | --- | --- | --- | --- | --- | --- | --- | --- | --- | --- | --- | --- | --- | --- | --- | --- | --- | --- | --- | --- | --- | --- | --- | --- | --- | --- | --- | --- | --- | --- | --- | --- | --- | --- | --- | --- | --- | --- | --- | --- | --- | --- | --- | --- | --- | --- | --- | --- | --- | --- | --- | --- | --- | --- | --- | --- | --- | --- | --- | --- | --- | --- | --- | --- | --- | --- | --- | --- | --- | --- | --- | --- | --- | --- | --- | --- | --- | --- | --- | --- | --- | --- | --- | --- | --- | --- | --- | --- | --- | --- | --- | --- | --- | --- | --- | --- | --- | --- | --- | --- | --- | --- | --- | --- | --- | --- | --- | --- | --- | --- | --- | --- | --- | --- | --- | --- | --- | --- | --- | --- | --- | --- | --- | --- | --- | --- | --- | --- | --- | --- | --- | --- | --- | --- | --- | --- | --- | --- | --- | --- | --- | --- | --- | --- | --- | --- | --- | --- | --- | --- | --- | --- | --- | --- | --- | --- | --- | --- | --- | --- | --- | --- | --- | --- | --- | --- | --- | --- | --- | --- | --- | --- | --- | --- | --- | --- | --- | --- | --- | --- | --- | --- | --- | --- | --- | --- | --- | --- | --- | --- | --- | --- | --- | --- | --- | --- | --- | --- | --- | --- | --- | --- | --- | --- | --- | --- | --- | --- | --- | --- | --- | --- | --- | --- | --- | --- | --- | --- | --- | --- | --- | --- | --- | --- | --- | --- | --- | --- | --- | --- | --- | --- | --- | --- | --- | --- | --- | --- | --- | --- | --- | --- | --- | --- | --- | --- | --- | --- | --- | --- | --- | --- | --- | --- | --- | --- | --- | --- | --- | --- | --- | --- | --- | --- | --- | --- | --- | --- | --- | --- | --- | --- | --- | --- | --- | --- | --- | --- | --- | --- | --- | --- | --- | --- | --- | --- | --- | --- | --- | --- | --- | --- | --- | --- | --- | --- | --- | --- | --- | --- | --- | --- | --- | --- | --- | --- | --- | --- | --- | --- | --- | --- | --- | --- | --- | --- | --- | --- | --- | --- | --- | --- | --- | --- | --- | --- | --- | --- | --- | --- | --- | --- | --- | --- | --- | --- | --- | --- | --- | --- | --- | --- | --- | --- | --- | --- | --- | --- | --- | --- | --- | --- | --- | --- | --- | --- | --- | --- | --- | --- | --- | --- | --- | --- | --- | --- | --- | --- | --- | --- | --- | --- | --- | --- | --- | --- | --- | --- | --- | --- | --- | --- | --- | --- | --- | --- | --- | --- | --- | --- | --- | --- | --- | --- | --- | --- | --- | --- | --- | --- | --- | --- | --- | --- | --- | --- | --- | --- | --- | --- | --- | --- | --- | --- | --- | --- | --- | --- | --- | --- | --- | --- | --- | --- | --- | --- | --- | --- | --- | --- | --- | --- | --- | --- | --- | --- | --- | --- | --- | --- | --- | --- | --- | --- | --- | --- | --- | --- | --- | --- | --- | --- | --- | --- | --- | --- | --- | --- | --- | --- | --- | --- | --- | --- | --- | --- | --- | --- | --- | --- | --- | --- | --- | --- | --- | --- | --- | --- | --- | --- | --- | --- | --- | --- | --- | --- | --- | --- | --- | --- | --- | --- | --- | --- | --- | --- | --- | --- | --- | --- | --- | --- | --- | --- | --- | --- | --- | --- | --- | --- | --- | --- | --- | --- | --- | --- | --- | --- | --- | --- | --- | --- | --- | --- | --- | --- | --- | --- | --- | --- | --- | --- | --- | --- | --- | --- | --- | --- | --- | --- | --- | --- | --- | --- | --- | --- | --- | --- | --- | --- | --- | --- | --- | --- | --- | --- | --- | --- | --- | --- | --- | --- | --- | --- | --- | --- | --- | --- | --- | --- | --- | --- | --- | --- | --- | --- | --- | --- | --- | --- | --- | --- | --- | --- | --- | --- | --- | --- | --- | --- | --- | --- | --- | --- | --- | --- | --- | --- | --- | --- | --- | --- | --- | --- | --- | --- | --- | --- | --- | --- | --- | --- | --- | --- | --- | --- | --- | --- | --- | --- | --- | --- | --- | --- | --- | --- | --- | --- | --- | --- | --- | --- | --- | --- | --- | --- | --- | --- | --- | --- | --- | --- | --- | --- | --- | --- | --- | --- | --- | --- | --- | --- | --- | --- | --- | --- | --- | --- | --- | --- | --- | --- | --- | --- | --- | --- | --- | --- | --- | --- | --- | --- | --- | --- | --- | --- | --- | --- | --- | --- | --- | --- | --- | --- | --- | --- | --- | --- | --- | --- | --- | --- | --- | --- | --- | --- | --- | --- | --- | --- | --- | --- | --- | --- | --- | --- | --- | --- | --- | --- | --- | --- | --- | --- | --- | --- | --- | --- | --- | --- | --- | --- | --- | --- | --- | --- | --- | --- | --- | --- | --- | --- | --- | --- | --- | --- | --- | --- | --- | --- | --- | --- | --- | --- | --- | --- | --- | --- | --- | --- | --- | --- | --- | --- | --- | --- | --- | --- | --- | --- | --- | --- | --- | --- | --- | --- | --- | --- | --- | --- | --- | --- | --- | --- | --- | --- | --- | --- | --- | --- | --- | --- | --- | --- | --- | --- | --- | --- | --- | --- | --- | --- | --- | --- | --- | --- | --- | --- | --- | --- | --- | --- | --- | --- | --- | --- | --- | --- | --- | --- | --- | --- | --- | --- | --- | --- | --- | --- | --- | --- | --- | --- | --- | --- | --- | --- | --- | --- | --- | --- | --- | --- | --- | --- | --- | --- | --- | --- | --- | --- | --- | --- | --- | --- | --- | --- | --- | --- | --- | --- | --- | --- | --- | --- | --- | --- | --- | --- | --- | --- | --- | --- | --- | --- | --- | --- | --- | --- | --- | --- | --- | --- | --- | --- | --- | --- | --- | --- | --- | --- | --- | --- | --- | --- | --- | --- | --- | --- | --- | --- | --- | --- | --- | --- | --- | --- | --- | --- | --- | --- | --- | --- | --- | --- | --- | --- | --- | --- | --- | --- | --- | --- | --- | --- | --- | --- | --- | --- | --- | --- | --- | --- | --- | --- | --- | --- | --- | --- | --- | --- | --- | --- | --- | --- | --- | --- | --- | --- | --- | --- | --- | --- | --- | --- | --- | --- | --- | --- | --- | --- | --- | --- | --- | --- | --- | --- | --- | --- | --- | --- | --- | --- | --- | --- | --- | --- | --- | --- | --- | --- | --- | --- | --- | --- | --- | --- | --- | --- | --- | --- | --- | --- | --- | --- | --- | --- | --- | --- | --- | --- | --- | --- | --- | --- | --- | --- | --- | --- | --- | --- | --- | --- | --- | --- | --- | --- | --- | --- | --- | --- | --- | --- | --- | --- | --- | --- | --- | --- | --- | --- | --- | --- | --- | --- | --- | --- | --- | --- | --- | --- | --- | --- | --- | --- | --- | --- | --- | --- | --- | --- | --- | --- | --- | --- | --- | --- | --- | --- | --- | --- | --- | --- | --- | --- | --- | --- | --- | --- | --- | --- | --- | --- | --- | --- | --- | --- | --- | --- | --- | --- | --- | --- | --- | --- | --- | --- | --- | --- | --- | --- | --- | --- | --- | --- | --- | --- | --- | --- | --- | --- | --- | --- | --- | --- | --- | --- | --- | --- | --- | --- | --- | --- | --- | --- | --- | --- | --- | --- | --- | --- | --- | --- | --- | --- | --- | --- | --- | --- | --- | --- | --- | --- | --- | --- | --- | --- | --- | --- | --- | --- | --- | --- | --- | --- | --- | --- | --- | --- | --- | --- | --- | --- | --- | --- | --- | --- | --- | --- | --- | --- | --- | --- | --- | --- | --- | --- | --- | --- | --- | --- | --- | --- | --- | --- | --- | --- | --- | --- | --- | --- | --- | --- | --- | --- | --- | --- | --- | --- | --- | --- | --- | --- | --- | --- | --- | --- | --- | --- | --- | --- | --- | --- | --- | --- | --- | --- | --- | --- | --- | --- | --- | --- | --- | --- | --- | --- | --- | --- | --- | --- | --- | --- | --- | --- | --- | --- | --- | --- | --- | --- | --- | --- | --- | --- | --- | --- | --- | --- | --- | --- | --- | --- | --- | --- | --- | --- | --- | --- | --- | --- | --- | --- | --- | --- | --- | --- | --- | --- | --- | --- | --- | --- | --- | --- | --- | --- | --- | --- | --- | --- | --- | --- | --- | --- | --- | --- | --- | --- | --- | --- | --- | --- | --- | --- | --- | --- | --- | --- | --- | --- | --- | --- | --- | --- | --- | --- | --- | --- | --- | --- | --- | --- | --- | --- | --- | --- | --- | --- | --- | --- | --- | --- | --- | --- | --- | --- | --- | --- | --- | --- | --- | --- | --- | --- | --- | --- | --- | --- | --- | --- | --- | --- | --- | --- | --- | --- | --- | --- | --- | --- | --- | --- | --- | --- | --- | --- | --- | --- | --- | --- | --- | --- | --- | --- | --- | --- | --- | --- | --- | --- | --- | --- | --- | --- | --- | --- | --- | --- | --- | --- | --- | --- | --- | --- | --- | --- | --- | --- | --- | --- | --- | --- | --- | --- | --- | --- | --- | --- | --- | --- | --- | --- | --- | --- | --- | --- | --- | --- | --- | --- | --- | --- | --- | --- | --- | --- | --- | --- | --- | --- | --- | --- | --- | --- | --- | --- | --- | --- | --- | --- | --- | --- | --- | --- | --- | --- | --- | --- | --- | --- | --- | --- | --- | --- | --- | --- | --- | --- | --- | --- | --- | --- | --- | --- | --- | --- | --- | --- | --- | --- | --- | --- | --- | --- | --- | --- | --- | --- | --- | --- | --- | --- | --- | --- | --- | --- | --- | --- | --- | --- | --- | --- | --- | --- | --- | --- | --- | --- | --- | --- | --- | --- | --- | --- | --- | --- | --- | --- | --- | --- | --- | --- | --- | --- | --- | --- | --- | --- | --- | --- | --- | --- | --- | --- | --- | --- | --- | --- | --- | --- | --- | --- | --- | --- | --- | --- | --- | --- | --- | --- | --- | --- | --- | --- | --- | --- | --- | --- | --- | --- | --- | --- | --- | --- | --- | --- | --- | --- | --- | --- | --- | --- | --- | --- | --- | --- | --- | --- | --- | --- | --- | --- | --- | --- | --- | --- | --- | --- | --- | --- | --- | --- | --- | --- | --- | --- | --- | --- | --- | --- | --- | --- | --- | --- | --- | --- | --- | --- | --- | --- | --- | --- | --- | --- | --- | --- | --- | --- | --- | --- | --- | --- | --- | --- | --- | --- | --- | --- | --- | --- | --- | --- | --- | --- | --- | --- | --- | --- | --- | --- | --- | --- | --- | --- | --- | --- | --- | --- | --- | --- | --- | --- | --- | --- | --- | --- | --- | --- | --- | --- | --- | --- | --- | --- | --- | --- | --- | --- | --- | --- | --- | --- | --- | --- | --- | --- | --- | --- | --- | --- | --- | --- | --- | --- | --- | --- | --- | --- | --- | --- | --- | --- | --- | --- | --- | --- | --- | --- | --- | --- | --- | --- | --- | --- | --- | --- | --- | --- | --- | --- | --- | --- | --- | --- | --- | --- | --- | --- | --- | --- | --- | --- | --- | --- | --- | --- | --- | --- | --- | --- | --- | --- | --- | --- | --- | --- | --- | --- | --- | --- | --- | --- | --- | --- | --- | --- | --- | --- | --- | --- | --- | --- | --- | --- | --- | --- | --- | --- | --- | --- | --- | --- | --- | --- | --- | --- | --- | --- | --- | --- | --- | --- | --- | --- | --- | --- | --- | --- | --- | --- | --- | --- | --- | --- | --- | --- | --- | --- | --- | --- | --- | --- | --- | --- | --- | --- | --- | --- | --- | --- | --- | --- | --- | --- | --- | --- | --- | --- | --- | --- | --- | --- | --- | --- | --- | --- | --- | --- | --- | --- | --- | --- | --- | --- | --- | --- | --- | --- | --- | --- | --- | --- | --- | --- | --- | --- | --- | --- | --- | --- | --- | --- | --- | --- | --- | --- | --- | --- | --- | --- | --- | --- | --- | --- | --- | --- | --- | --- | --- | --- | --- | --- | --- | --- | --- | --- | --- | --- | --- | --- | --- | --- | --- | --- | --- | --- | --- | --- | --- | --- | --- | --- | --- | --- | --- | --- | --- | --- | --- | --- | --- | --- | --- | --- | --- | --- | --- | --- | --- | --- | --- | --- | --- | --- | --- | --- | --- | --- | --- | --- | --- | --- | --- | --- | --- | --- | --- | --- | --- | --- | --- | --- | --- | --- | --- | --- | --- | --- | --- | --- | --- | --- | --- | --- | --- | --- | --- | --- | --- | --- | --- | --- | --- | --- | --- | --- | --- | --- | --- | --- | --- | --- | --- | --- | --- | --- | --- | --- | --- | --- | --- | --- | --- | --- | --- | --- | --- | --- | --- | --- | --- | --- | --- | --- | --- | --- | --- | --- | --- | --- | --- | --- | --- | --- | --- | --- | --- | --- | --- | --- | --- | --- | --- | --- | --- | --- | --- | --- | --- | --- | --- | --- | --- | --- | --- | --- | --- | --- | --- | --- | --- | --- | --- | --- | --- | --- | --- | --- | --- | --- | --- | --- | --- | --- | --- | --- | --- | --- | --- | --- | --- | --- | --- | --- | --- | --- | --- | --- | --- | --- | --- | --- | --- | --- | --- | --- | --- | --- | --- | --- | --- | --- | --- | --- | --- | --- | --- | --- | --- | --- | --- | --- | --- | --- | --- | --- | --- | --- | --- | --- | --- | --- | --- | --- | --- | --- | --- | --- | --- | --- | --- | --- | --- | --- | --- | --- | --- | --- | --- | --- | --- | --- | --- | --- | --- | --- | --- | --- | --- | --- | --- | --- | --- | --- | --- | --- | --- | --- | --- | --- | --- | --- | --- | --- | --- | --- | --- | --- | --- | --- | --- | --- | --- | --- | --- | --- | --- | --- | --- | --- | --- | --- | --- | --- | --- | --- | --- | --- | --- | --- | --- | --- | --- | --- | --- | --- | --- | --- | --- | --- | --- | --- | --- | --- | --- | --- | --- | --- | --- | --- | --- | --- | --- | --- | --- | --- | --- | --- | --- | --- | --- | --- | --- | --- | --- | --- | --- | --- | --- | --- | --- | --- | --- | --- | --- | --- | --- | --- | --- | --- | --- | --- | --- | --- | --- | --- | --- | --- | --- | --- | --- | --- | --- | --- | --- | --- | --- | --- | --- | --- | --- | --- | --- | --- | --- | --- | --- | --- | --- | --- | --- | --- | --- | --- | --- | --- | --- | --- | --- | --- | --- | --- | --- | --- | --- | --- | --- | --- | --- | --- | --- | --- | --- | --- | --- | --- | --- | --- | --- | --- | --- | --- | --- | --- | --- | --- | --- | --- | --- | --- | --- | --- | --- | --- | --- | --- | --- | --- | --- | --- | --- | --- | --- | --- | --- | --- | --- | --- | --- | --- | --- | --- | --- | --- | --- | --- | --- | --- | --- | --- | --- | --- | --- | --- | --- | --- | --- | --- | --- | --- | --- | --- | --- | --- | --- | --- | --- | --- | --- | --- | --- | --- | --- | --- | --- | --- | --- | --- | --- | --- | --- | --- | --- | --- | --- | --- | --- | --- | --- | --- | --- | --- | --- | --- | --- | --- | --- | --- | --- | --- | --- | --- | --- | --- | --- | --- | --- | --- | --- | --- | --- | --- | --- | --- | --- | --- | --- | --- | --- | --- | --- | --- | --- | --- | --- | --- | --- | --- | --- | --- | --- | --- | --- | --- | --- | --- | --- | --- | --- | --- | --- | --- | --- | --- | --- | --- | --- | --- | --- | --- | --- | --- | --- | --- | --- | --- | --- | --- | --- | --- | --- | --- | --- | --- | --- | --- | --- | --- | --- | --- | --- | --- | --- | --- | --- | --- | --- | --- | --- | --- | --- | --- | --- | --- | --- | --- | --- | --- | --- | --- | --- | --- | --- | --- | --- | --- | --- | --- | --- | --- | --- | --- | --- | --- | --- | --- | --- | --- | --- | --- | --- | --- | --- | --- | --- | --- | --- | --- | --- | --- | --- | --- | --- | --- | --- | --- | --- | --- | --- | --- | --- | --- | --- | --- | --- | --- | --- | --- | --- | --- | --- | --- | --- | --- | --- | --- | --- | --- | --- | --- | --- | --- | --- | --- | --- | --- | --- | --- | --- | --- | --- | --- | --- | --- | --- | --- | --- | --- | --- | --- | --- | --- | --- | --- | --- | --- | --- | --- | --- | --- | --- | --- | --- | --- | --- | --- | --- | --- | --- | --- | --- | --- | --- | --- | --- | --- | --- | --- | --- | --- | --- | --- | --- | --- | --- | --- | --- | --- | --- | --- | --- | --- | --- | --- | --- | --- | --- | --- | --- | --- | --- | --- | --- | --- | --- | --- | --- | --- | --- | --- | --- | --- | --- | --- | --- | --- | --- | --- | --- | --- | --- | --- | --- | --- | --- | --- | --- | --- | --- | --- | --- | --- | --- | --- | --- | --- | --- | --- | --- | --- | --- | --- | --- | --- | --- | --- | --- | --- | --- | --- | --- | --- | --- | --- | --- | --- | --- | --- | --- | --- | --- | --- | --- | --- | --- | --- | --- | --- | --- | --- | --- | --- | --- | --- | --- | --- | --- | --- | --- | --- | --- | --- | --- | --- | --- | --- | --- | --- | --- | --- | --- | --- | --- | --- | --- | --- | --- | --- | --- | --- | --- | --- | --- | --- | --- | --- | --- | --- | --- | --- | --- | --- | --- | --- | --- | --- | --- | --- | --- | --- | --- | --- | --- | --- | --- | --- | --- | --- | --- | --- | --- | --- | --- | --- | --- | --- | --- | --- | --- | --- | --- | --- | --- | --- | --- | --- | --- | --- | --- | --- | --- | --- | --- | --- | --- | --- | --- | --- | --- | --- | --- | --- | --- | --- | --- | --- | --- | --- | --- | --- | --- | --- | --- | --- | --- | --- | --- | --- | --- | --- | --- | --- | --- | --- | --- | --- | --- | --- | --- | --- | --- | --- | --- | --- | --- | --- | --- | --- | --- | --- | --- | --- | --- | --- | --- | --- | --- | --- | --- | --- | --- | --- | --- | --- | --- | --- | --- | --- | --- | --- | --- | --- | --- | --- | --- | --- | --- | --- | --- | --- | --- | --- | --- | --- | --- | --- | --- | --- | --- | --- | --- | --- | --- | --- | --- | --- | --- | --- | --- | --- | --- | --- | --- | --- | --- | --- | --- | --- | --- | --- | --- | --- | --- | --- | --- | --- | --- | --- | --- | --- | --- | --- | --- | --- | --- | --- | --- | --- | --- | --- | --- | --- | --- | --- | --- | --- | --- | --- | --- | --- | --- | --- | --- | --- | --- | --- | --- | --- | --- | --- | --- | --- | --- | --- | --- | --- | --- | --- | --- | --- | --- | --- | --- | --- | --- | --- | --- | --- | --- | --- | --- | --- | --- | --- | --- | --- | --- | --- | --- | --- | --- | --- | --- | --- | --- | --- | --- | --- | --- | --- | --- | --- | --- | --- | --- | --- | --- | --- | --- | --- | --- | --- | --- | --- | --- | --- | --- | --- | --- | --- | --- | --- | --- | --- | --- | --- | --- | --- | --- | --- | --- | --- | --- | --- | --- | --- | --- | --- | --- | --- | --- | --- | --- | --- | --- | --- | --- | --- | --- | --- | --- | --- | --- | --- | --- | --- | --- | --- | --- | --- | --- | --- | --- | --- | --- | --- | --- | --- | --- | --- | --- | --- | --- | --- | --- | --- | --- | --- | --- | --- | --- | --- | --- | --- | --- | --- | --- | --- | --- | --- | --- | --- | --- | --- | --- | --- | --- | --- | --- | --- | --- | --- | --- | --- | --- | --- | --- | --- | --- | --- | --- | --- | --- | --- | --- | --- | --- | --- | --- | --- | --- | --- | --- | --- | --- | --- | --- | --- | --- | --- | --- | --- | --- | --- | --- | --- | --- | --- | --- | --- | --- | --- | --- | --- | --- | --- | --- | --- | --- | --- | --- | --- | --- | --- | --- | --- | --- | --- | --- | --- | --- | --- | --- | --- | --- | --- | --- | --- | --- | --- | --- | --- | --- | --- | --- | --- | --- | --- | --- | --- | --- | --- | --- | --- | --- | --- | --- | --- | --- | --- | --- | --- | --- | --- | --- | --- | --- | --- | --- | --- | --- | --- | --- | --- | --- | --- | --- | --- | --- | --- | --- | --- | --- | --- | --- | --- | --- | --- | --- | --- | --- | --- | --- | --- | --- | --- | --- | --- | --- | --- | --- | --- | --- | --- | --- | --- | --- | --- | --- | --- | --- | --- | --- | --- | --- | --- | --- | --- | --- | --- | --- | --- | --- | --- | --- | --- | --- | --- | --- | --- | --- | --- | --- | --- | --- | --- | --- | --- | --- | --- | --- | --- | --- | --- | --- | --- | --- | --- | --- | --- | --- | --- | --- | --- | --- | --- | --- | --- | --- | --- | --- | --- | --- | --- | --- | --- | --- | --- | --- | --- | --- | --- | --- | --- | --- | --- | --- | --- | --- | --- | --- | --- | --- | --- | --- | --- | --- | --- | --- | --- | --- | --- | --- | --- | --- | --- | --- | --- | --- | --- | --- | --- | --- | --- | --- | --- | --- | --- | --- | --- | --- | --- | --- | --- | --- | --- | --- | --- | --- | --- | --- | --- | --- | --- | --- | --- | --- | --- | --- | --- | --- | --- | --- | --- | --- | --- | --- | --- | --- | --- | --- | --- | --- | --- | --- | --- | --- | --- | --- | --- | --- | --- | --- | --- | --- | --- | --- | --- | --- | --- | --- | --- | --- | --- | --- | --- | --- | --- | --- | --- | --- | --- | --- | --- | --- | --- | --- | --- | --- | --- | --- | --- | --- | --- | --- | --- | --- | --- | --- | --- | --- | --- | --- | --- | --- | --- | --- | --- | --- | --- | --- | --- | --- | --- | --- | --- | --- | --- | --- | --- | --- | --- | --- | --- | --- | --- | --- | --- | --- | --- | --- | --- | --- | --- | --- | --- | --- | --- | --- | --- | --- | --- | --- | --- | --- | --- | --- | --- | --- | --- | --- | --- | --- | --- | --- | --- | --- | --- | --- | --- | --- | --- | --- | --- | --- | --- | --- | --- | --- | --- | --- | --- | --- | --- | --- | --- | --- | --- | --- | --- | --- | --- | --- | --- | --- | --- | --- | --- | --- | --- | --- | --- | --- | --- | --- | --- | --- | --- | --- | --- | --- | --- | --- | --- | --- | --- | --- | --- | --- | --- | --- | --- | --- | --- | --- | --- | --- | --- | --- | --- | --- | --- | --- | --- | --- | --- | --- | --- | --- | --- | --- | --- | --- | --- | --- | --- | --- | --- | --- | --- | --- | --- | --- | --- | --- | --- | --- | --- | --- | --- | --- | --- | --- | --- | --- | --- | --- | --- | --- | --- | --- | --- | --- | --- | --- | --- | --- | --- | --- | --- | --- | --- | --- | --- | --- | --- | --- | --- | --- | --- | --- | --- | --- | --- | --- | --- | --- | --- | --- | --- | --- | --- | --- | --- | --- | --- | --- | --- | --- | --- | --- | --- | --- | --- | --- | --- | --- | --- | --- | --- | --- | --- | --- | --- | --- | --- | --- | --- | --- | --- | --- | --- | --- | --- | --- | --- | --- | --- | --- | --- | --- | --- | --- | --- | --- | --- | --- | --- | --- | --- | --- | --- | --- | --- | --- | --- | --- | --- | --- | --- | --- | --- | --- | --- | --- | --- | --- | --- | --- | --- | --- | --- | --- | --- | --- | --- | --- | --- | --- | --- | --- | --- | --- | --- | --- | --- | --- | --- | --- | --- | --- | --- | --- | --- | --- | --- | --- | --- | --- | --- | --- | --- | --- | --- | --- | --- | --- | --- | --- | --- | --- | --- | --- | --- | --- | --- | --- | --- | --- | --- | --- | --- | --- | --- | --- | --- | --- | --- | --- | --- | --- | --- | --- | --- | --- | --- | --- | --- | --- | --- | --- | --- | --- | --- | --- | --- | --- | --- | --- | --- | --- | --- | --- | --- | --- | --- | --- | --- | --- | --- | --- | --- | --- | --- | --- | --- | --- | --- | --- | --- | --- | --- | --- | --- | --- | --- | --- | --- | --- | --- | --- | --- | --- | --- | --- | --- | --- | --- | --- | --- | --- | --- | --- | --- | --- | --- | --- | --- | --- | --- | --- | --- | --- | --- | --- | --- | --- | --- | --- | --- | --- | --- | --- | --- | --- | --- | --- | --- | --- | --- | --- | --- | --- | --- | --- | --- | --- | --- | --- | --- | --- | --- | --- | --- | --- | --- | --- | --- | --- | --- | --- | --- | --- | --- | --- | --- | --- | --- | --- | --- | --- | --- | --- | --- | --- | --- | --- | --- | --- | --- |

^A^Class I UGMS primers

^B^50 class I UGMS primers used for studying cross-transferability and polymorphic potential

*UGSuM stands for unigene derived sugarcane microsatellite primers
