# Supplementary material for: Functionally relevant microsatellites in sugarcane unigenes
Source: BMC Plant Biol. 2010 Nov 17;10:251. doi: 10.1186/1471-2229-10-251 (PMC3017843; doi:10.1186/1471-2229-10-251)
Supplement: Additional file 4 — Primers designed from sugarcane unigenes carrying compound class I microsatellite repeat-motifs along with the expected amplicon size and putative unigene function. [file 1471-2229-10-251-S4.DOC]

| **Additional file 4: Primers designed targeting the sugarcane unigenes carrying compound class I microsatellite repeat-motifs along with primer sequences, expected product size and putative unigene function** |  |  |  |  |  |  |  |  |  |  |
| --- | --- | --- | --- | --- | --- | --- | --- | --- | --- | --- |
| |  |  |  |  |  |  |  |  |  |  | | --- | --- | --- | --- | --- | --- | --- | --- | --- | --- | | **Unigene IDs'** | **Class I UGMS primers*** | **Compound class I microsatellite repeat-motifs** | **Location** | **Forward primer (5'-3')** | **Reverse primers (5'-3')** | **Left**  **TM** | **Right**  **TM** | **Product Size** | **Putative functions** | | CA094806^B^ | UGSuM811 | (TCACAG)10tgtac (CA)10 | CDS | AGAGAGAGAGAGACAAAGGATG | TAATGGAATTGAGAGAATGTGA | 54 | 54 | 313 | AUX1-like permease, *Oryza* | | CA093499^B^ | UGSuM812 | (GA)61ggag...(GA)22t(AG)14 | 5'UTRs | CTGTCATCCTCTCATCCATC | GTCTCCTCCTCGCTCCGCT | 55 | 64 | 192 | auxin and ethylene responsive GH3-like protein, *Arabidopsis* | | CA258125^B^ | UGSuM813 | (CT)21gtat..(AC)28 | CDS | AAACAGAATTGCAGCCTTTAT | GACATGAAACTTTGTTGATCTG | 55 | 54 | 148 | peptide deformylase, *Oryza* | | CA258354^B^ | UGSuM814 | (CT)6atatat..(AG)25 | CDS | AGAAGGAACCAGGATAGAGAAT | AAGTAAGAAAGACGAACTCTGC | 55 | 54 | 196 | protochlorophyllide reductase protein, *Arabidopsis* | | CA146513^B^ | UGSuM815 | (GT)8taat...(AT)26 | CDS | CTTAGTTTCCGCCTTCCA | CCATTGACTGTATTCTCATC | 56 | 50 | 281 | unknown protein, *Oryza* | | CA291516^B^ | UGSuM816 | (AT)7gaact...(AAAT)7 | CDS | AACTAACAATGACAGTTCCTCC | ATCGTAATACTCATCGTACCGT | 54 | 55 | 371 | cyclin-dependent kinases, *Arabidopsis* | | CA208759^B^ | UGSuM817 | (AGAAGC)5tgtag...(CGG)5 | 5'UTRs | ACTAGTTCTAGATCGCGAGCGG | AGATGAGAAGAGAGCTCAGTGT | 62 | 54 | 339 | expressed protein, *Oryza* | | CA256933^B^ | UGSuM818 | (T)22accaa...(CATA)15 | 3'UTRs | TACTCCTCCTCTACCATCAAAG | TGAGAATCTCTATTCGTTGACA | 55 | 55 | 162 | hypothetical protein, *Oryza* | | CA126151^B^ | UGSuM819 | (CCCTG)5cttctc...(GCC)6 | CDS | AGATGGATGAGGGTTTCTTT | CCTACGAGTTTATTCTTCAGT | 55 | 50 | 273 | gibberellin 20-oxidase, *Zea mays* | | CA171064^B^ | UGSuM820 | (AG)17tcgt...(TC)10 | CDS | AGATAACATACAAGCCCACATC | CTGTGGTCAGAAACTCATACAC | 55 | 54 | 310 | PotyvirusVPg-interacting protein, *Nicotiana* | | CA093387^B^ | UGSuM821 | (T)19acaag...(CACG)5 | CDS | GTGCTCTTCCTTTCACTTTATT | TGTAGATCTTGTACGTCTCCAC | 54 | 54 | 347 | expressed protein, Oryza | | CA271033^B^ | UGSuM822 | (GCT)5gccat...(TGGTGC)5 | CDS | TCTTGAAGGGTAAATGGATAAC | CTCTACTCATCTGTTCCAACCT | 55 | 55 | 357 | hypothetical protein, *Oryza* | | CA293543^B^ | UGSuM823 | (CA)7(CACG)10cgc..(CA)36 | CDS | CAGAAACGGAGAACGGTG | TACACAGCACAGGATGGTAA | 57 | 55 | 161 | asparagine synthetase, *Asparagus* | | CA074558^B^ | UGSuM824 | (AT)9cacca...(AC)12 | 5'UTRs | GGAAGGTCACACGCAATCTG | AGCTAGAGGAGAAGTGTCGTAG | 61 | 54 | 312 | hypothetical protein, *Oryza* | | CA089582^B^ | UGSuM825 | (ACG)6actat..(GGCG)5 | 3'UTRs | CGAATCTGGAAAGAGAGTAAAC | GACACAATTTCAAATCTTCCTT | 54 | 55 | 110 | Carbamoyl phosphate synthetase, *Arabidopsis* | | CA121081^B^ | UGSuM826 | (TTAG)6tatac..(AG)16 | CDS | ACAACTCCAAAGAGAGAAGAAA | TAGATCTACCGAGCAGAGAAAT | 55 | 55 | 320 | hypothetical protein, *Oryza* | | CA250619^B^ | UGSuM827 | (GAG)6ctctga..(GTGA)6(GA)11 | CDS | TAGACCTTCAACTCCATCTCTC | GATTCTACGAAAGAGATCCAAC | 55 | 54 | 308 | hypothetical protein, *Oryza* | | CA194621^B^ | UGSuM828 | (AG)7attag..(TG)10 | CDS | ATTGATGTTCTTCTACCTCCAC | TACTCGAAGCAATGAGACATAC | 54 | 54 | 287 | hypothetical protein, *Oryza* | | CA117179^B^ | UGSuM829 | (GGC)5aat..(GGC)8gga..(CAG)5 | 5'UTRs | GCTTGCCATCTCCCTTCT | CATCCTGTTGTTCCTCTCAC | 57 | 55 | 128 | DEAD/DEAH box helicase, *Oryza* | | CA122161^B^ | UGSuM830 | (GCG)6cgc...gct(GC)6 | 5'UTRs | AAACTCTCTTCTTCGCCTC | TGGATGTGTGTATGATTCTTG | 53 | 54 | 395 | plastid envelope DNA binding protein, *Pisum* | | CA207282^B^ | UGSuM831 | (CGG)5gcg...ga(GCG)5 | CDS | CTAAATACAGCACACGCTAAA | ATCTTCCTGGCGGTTATG | 53 | 56 | 186 | transcription factor IIIA, *Oryza* | | CA238990^B^ | UGSuM832 | (ATT)5gttg..(AC)46 | 5'UTRs | AGTTACCAACAAGGAGAGTGTC | TCACAACTCTACAGACTCTTCG | 55 | 54 | 387 | hypothetical protein, *Oryza* | | CA198392^B^ | UGSuM833 | (AT)6gatat...(TA)37 | CDS | GGGTTTACAACAATCAGTTCTT | TTGATATCATCTAAGCTCCACA | 55 | 55 | 361 | GDP-mannose pyrophosphorylase, *Arabidopsis* | | CA207294^B^ | UGSuM834 | (CGG)9cgcc...(GCA)6 | CDS | ACTCCTGTTTGTGCAATTAAA | CTAGGAATTGAAGCTGAGATTT | 55 | 54 | 116 | expressed protein, Oryza | | CA093565^B^ | UGSuM835 | (TG)24aggat...(CT)6 | 3'UTRs | GTAGACGAGGAGAGGGAGTT | GAAGTGAGAGAGGAGACTTTGA | 54 | 55 | 300 | Reverse transcriptase, *Oryza* | | CA175096^A^ | UGSuM836 | (CTC)6(CT)11cccgt...(CGG)7 | CDS | GTTTCAGATCTCTCCTGGTAAA | TTGGAAGTTGTTCTGTTGTAGA | 55 | 55 | 194 | ribosomal protein, *Arabidopsis* | | CA103803^B^ | UGSuM837 | (CGG)6tgg...g(CA)11 | 5'UTRs | ACACCAACACCTACACCAA | GGCTATCAAATACCTTCACCT | 54 | 55 | 400 | Phosphatase and actin regulator 4, *Oryza* | | CA246196^B^ | UGSuM838 | (AT)12actgt..(AC)17 | 3'UTRs | AACATGGCCTATCTCAAGTATC | TGTACTTTGCTCGCTTCTAAC | 55 | 55 | 236 | Serine/threonine-protein kinase ctr1, *Arabidopsis* | | CA258169^A^ | UGSuM839 | (CTC)5(CT)6ccgat...(CCT)5 | CDS | GTCTATTGCTAAACCCTCTCAC | AACACCCATCATGGTTTATTAT | 54 | 55 | 241 | hypothetical protein, *Oryza* | | AY596531^B^ | UGSuM840 | (CT)10taaac..(TTC)5 | CDS | GGGTTTACAACAATCAGTTCTT | TTGATATCATCTAAGCTCCACA | 55 | 55 | 361 | Expressed protein, *Arabidopsis* | | CA171622^B^ | UGSuM841 | (CT)6catct..(AT)10 | CDS | GCCGCGTCGACTTTGATTCT | GTATCTCACGTGCTATCTTCG | 64 | 55 | 390 | Dwarf protein, *Oryza* | | CA258406^B^ | UGSuM842 | (AGG)5ggac...g(AGC)6 | CDS | TCCAACTCACCTCAACAACT | CATTCGGGAGCCACTTCA | 55 | 60 | 290 | Dof domain zinc finger DNA-binding protein, *Picea* | | CA153774^A^ | UGSuM843 | (TTTC)5(TTC)5accat...(AG)13 | CDS | AGATAAGGACACGGTGAATAAG | AGCAGCATAGATGAAAGAAAGT | 55 | 55 | 321 | Ser/Thr phosphatase, *Arabidopsis* | | CA139166^B^ | UGSuM844 | (CT)16(AT)18c(TA)10gga(GT)10 | CDS | ACCACCACAGTTTCAGCA | CTTTCACGACGAGGGAGA | 55 | 57 | 289 | protease inhibitor/LTP family protein, *Arabidopsis* | | CA179066^B^ | UGSuM845 | (TGC)5aagt...tt(TGC)6 | CDS | ACATACACTCCTAAACTACGG | GGCACACATAGACAAGGG | 51 | 54 | 311 | conserved hypothetical protein, *Oryza* | | CA179066^B^ | UGSuM846 | (TGC)5aagt...(TGC)6 | CDS | CTGTGTTCACCAAGTTAATGAG | AAGAAGCAGAAGAGAGTATGGA | 55 | 55 | 373 | Patatin-like protein, *Arabidopsis* | | CA234461^B^ | UGSuM847 | (AT)10ctcaa...(GCG)5 | CDS | AACCCTAAACCCTAAATCAGTT | AAGAAGCAGAAGAGAGTATGGA | 55 | 55 | 373 | Expressed protein, *Arabidopsis* | | CA110569^B^ | UGSuM848 | (TC)8ttgcact..(AC)7 | 5'UTRs | AGTTTCTTGGGAGAGGAAAG | TGAGAAGAAAGTGACCATCATA | 55 | 54 | 378 | Protein phosphatase 2C, *Arabidopsis* | | CA164565^B^ | UGSuM849 | (TC)7tgtgcg..(AG)18 | CDS | ATAGATAACAACGAGGAAGTCG | AACTTTGTACAGATCGCATGTA | 55 | 55 | 177 | Triose phosphate isomerase, *Oryza* | | CA153011^B^ | UGSuM850 | (CGC)6gccg...(GC)11(GA)8 | 3'UTRS | GAGAGCAGAAGCAAGAGAAA | AGTACTTGCAGTGTGTTGAAAG | 55 | 54 | 177 | hypothetical protein, *Oryza* | | CA113963^A^ | UGSuM851 | (ATCT)11(TAT)17 | CDS | ATCGTTACATTCTCCTTCTGAT | CAATAGCACTAGTTCCTCCATC | 54 | 55 | 360 | glycosyl transferase, *Oryza* | | CA185021^B^ | UGSuM852 | (AGC)5cgc…ga(GT)20 | CDS | TCTATCTGCTCCCTGCTTACT | ATGGTAGGTTGATGTTGGACT | 55 | 55 | 306 | expressed protein, *Oryza* | | CA084581^B^ | UGSuM853 | (CTCC)5ctc...tg(TC)10 | CDS | CTCTCTCTCCCTCTCCGT | CACAGACGAACAATCCATC | 54 | 54 | 358 | laccase, *Zea mays* | | CA143026^B^ | UGSuM854 | (TCCCT)5tcca...cc(GA)12 | CDS | AGAGGGATAGGAGGAGGAG | ATTATGGAATACTAAGCACGG | 54 | 53 | 400 | unknown protein, *Arabidopsis* | | CA093399^B^ | UGSuM855 | (AT)6gtatgt..(TG)6 | CDS | GTCTTGACTGCTTCATCTCTCT | CAGTCTAACGAATAAACCTTCC | 55 | 54 | 385 | expressed protein, *Triticum* | | CA106149^B^ | UGSuM856 | (TA)6aatat...tc(TA)10 | 5'UTRs | CTGTTGCTGTTCCTGCTC | GTTCCTAAATGAGATACACGG | 55 | 53 | 351 | hydrolase family protein, *Arabidopsis* | | CA106149^B^ | UGSuM857 | (TA)6aatat...(CT)10 | 3'UTRs | AGGTATGATAGGCTGAGGAGTA | CTGTACCTAAACCTTGGATGTT | 54 | 55 | 226 | hypothetical protein, *Oryza* | | CA141259^B^ | UGSuM858 | (GTAT)7gtacag...(CA)29 | 5'UTRs | ACAGTTTCAAGAAGGTGATTTC | AGTGTATAGTTGAAGGAGGTGG | 55 | 55 | 333 | hypothetical protein, *Oryza* | | CA230213^B^ | UGSuM859 | (GTAT)7gtacag..(AT)29 | CDS | CATCTTCATCTTTCTTCTCCTT | GATTACGGGAGAGCTATACTTG | 54 | 54 | 353 | succinyl-CoA ligase (GDP-forming), *Arabidopsis* | | CA257488^B^ | UGSuM860 | (CT)6gcagc..(GCC)8 | CDS | CATTTCAGTCCCTCATCTAACT | ATCGTAGCTTATGAACTGTCCT | 55 | 54 | 219 | hypothetical protein, *Oryza* | | CA143052^B^ | UGSuM861 | (AG)6acgggt..(CCG)5 | 5'UTRs | TTACTATATCGGATCGCCTATT | TTTAACAAAGGTAAGGAGGAAA | 54 | 55 | 225 | expressed protein, Oryza | | CA246097^B^ | UGSuM862 | (AG)19gcac..c(AG)19 | 3'UTRS | CTGCTGGTGGCTCTTCAC | GGAGATGCTAAGGCTGTTC | 57 | 54 | 358 | hypothetical protein, *Oryza* | | CA231510^B^ | UGSuM863 | (GAA)5gcgctc..(TCC)7 | CDS | ACTCCTGTTTGTGCAATTAAA | CTAGGAATTGAAGCTGAGATTT | 55 | 54 | 116 | ribosomal protein L17-like protein, *Pisum* | | CA279512^B^ | UGSuM864 | (CGT)5cgggc..(GGC)5 | CDS | CATCCATTTCGAATTATTACCT | ACTTTCTGATAAGCCAGTCAAC | 55 | 55 | 378 | PGPD14 protein, *Arabidopsis* | | CA150670^B^ | UGSuM865 | (TGC)7tactg...(AG)17 | CDS | TTCCTCTTCTTCCTTCTAATCA | GACCAGATTGCTGTCTTCTTAG | 55 | 55 | 346 | tubulin beta-7 chain (Beta-7 tubulin), *Oryza* | | CA210983^B^ | UGSuM866 | (AC)6cttc..(TA)17 | CDS | ATCCGACTCTGAGCAGCTTT | AACCAGACTTAACACCACTCTC | 59 | 55 | 127 | expressed protein, *Arabidopsis* | | CA219198^B^ | UGSuM867 | (GGA)5ccg...gtc(CGT)5 | 3'UTRS | ATAACGGCTCCCTACAACTTC | TCCTTGACCATAATAACATCAC | 57 | 54 | 289 | NOI-like protein, *Arabidopsis* | | CA077024^B^ | UGSuM868 | (CG)7agag...ggct(GCG)6 | CDS | ATACTCCATTCAGGTGGCAG | AGGCTTGTTTGCTTTCAG | 57 | 54 | 367 | protein cdc2 kinase, *Oryza* | | CA149973^B^ | UGSuM869 | (AC)12tnaa...ttt(TA)7 | CDS | GAACGACGAGACTGGAAG | GACCTGGAAATAACCCAAAC | 54 | 55 | 360 | hydroxyproline-rich glycoprotein family protein, *Arabidopsis* | | CA206075^B^ | UGSuM870 | (GA)10gca...gg(GGC)7 | CDS | AACTTCTGTGCTTTCTCCTCT | GCTCAACTGGATGCTGAA | 54 | 55 | 145 | transcription initiation protein, *Arabidopsis* | | CA275761^B^ | UGSuM871 | (ACG)5atg...ggt(GGC)5 | CDS | CTGAGGGAGGAGTTTCCA | GCAAGATGCTGAAGGGCA | 56 | 61 | 224 | lipid transfer protein 7a2b, *Hordeum vulgare* | | CA279443^B^ | UGSuM872 | (TAT)5tggatc...(A)27 | CDS | CTACTGTGGAAGGAATAATGGT | CTGTTGCTGATGTCTTTGAAT | 54 | 54 | 139 | delta-aminolevulinic acid dehydratase, *Arabidopsis* | | CA198419^B^ | UGSuM873 | (TGG)6cgac..(TC)7 | 5'UTRS | GAAGTCTTTCAGAGAAGCAAAT | GTCTTCTTGATAGTAACGCCTC | 54 | 55 | 337 | cyclopropane synthase, *Arabidopsis* | | CA218472^B^ | UGSuM874 | (ACG)5ctggct..(CTT)7 | CDS | CTTCCTGAATAATCCTGACC | GTTGCTGTTATTACTGTGATGC | 53 | 55 | 317 | serine protease, *Oryza* | | CA065724^B^ | UGSuM875 | (GAA)5gggca...(GAG)5 | 3'UTRs | TCAATTTGAGTGGTTTGATTTA | GCCTTACATATGGATACCAAGT | 54 | 54 | 392 | hypothetical protein, *Oryza* | | CA176184^B^ | UGSuM876 | (GCG)5acg...(AT)14 | CDS | AAGGAAGACGACGAGGAG | GTCAGCACCACAGGAAAG | 55 | 54 | 367 | ribosomal protein S27, *Saccharum* | | CA099824^B^ | UGSuM877 | (GGC)5gtt...tga(GCG)6 | 5'UTRs | CTATCCGCTTGCTTTATTGA | ATTCTCTCACATCCAACCC | 55 | 54 | 306 | kinase-related, *Arabidopsis* | | CA105091^B^ | UGSuM878 | (ATA)6accg..(CA)42 | 5'UTRs | GACATCTTCATTTCCTTCATTT | GTTCAAGCTGAATTATTTGGA | 54 | 54 | 335 | hypothetical protein, *Oryza* | | CA152008^B^ | UGSuM879 | (TGT)5tga...(AT)28 | 3'UTRs | TTACTATATCGGATCGCCTATT | TTTAACAAAGGTAAGGAGGAAA | 54 | 55 | 225 | fimbrin- protein (Actin binding motif), *Oryza* | | CA070690^B^ | UGSuM880 | (CGC)5cgag..(GCT)7 | CDS | AGTGTCTGTCGAGTTCCTTGT | CTAGTACAATCCCTGGAGAAAT | 55 | 54 | 363 | expressed protein, Oryza | | CA210294^B^ | UGSuM881 | (AC)6ccac..(TGAG)5(CGAG)6 | CDS | GTCTATTGCTAAACCCTCTCAC | AACACCCATCATGGTTTATTAT | 54 | 55 | 241 | hypothetical protein, *Oryza* | | CA257277^B^ | UGSuM882 | (AATC)5aaca..(A)30 | 5'UTRs | CATTTCTTCTCTCTTGGGTCT | CTTCTTCTCGTTTGACTTTACC | 55 | 55 | 194 | RNA-binding protein, *Arabidopsis* | | CA095721^B^ | UGSuM883 | (CT)6cat...gt(GGC)5 | 5'UTRs | ATCTGCTCCCTCCTCTTC | CCGATGCCGTTGTAGGTC | 54 | 60 | 228 | hypothetical protein, membrane protein, *Arabidopsis* | | CA139111^B^ | UGSuM884 | (CAC)5ctc...cg(CGC)5 | CDS | CTCTTCCTCTCCTGCCGT | CTCCTCCTCCTATCCCTCT | 58 | 54 | 211 | hypothetical protein, *Oryza* | | CA065704^B^ | UGSuM885 | (AAG)5gaga..(CAG)5 | CDS | AATCATCATCTTCTACGGTTTC | TGATTGAAGATAATAGAAGCCC | 55 | 55 | 137 | SMC6 protein, *Arabidopsis* | | CA289517^B^ | UGSuM886 | (CGC)5cg...ag(GAC)5 | CDS | TATTATGTCTGGAGCGGGTT | AAGAAGAGGGTTGACTTTCAC | 57 | 55 | 287 | transmembrane efflux protein, *Arabidopsis* | | CA105750^B^ | UGSuM887 | (AC)23tacg...cc(TA)6 | 5'UTRs | CTGTTGCTGTTCCTGCTC | GTTCCTAAATGAGATACACGG | 55 | 53 | 351 | hypothetical protein, *Oryza* | | CA105750^B^ | UGSuM888 | (A)23tacga...(TA)6 | CDS | CTGGATACATGACAGAGAAGTG | CAAACATAAGCACTGAAAGAAA | 54 | 54 | 214 | hypothetical protein, *Oryza* | | CA128269^B^ | UGSuM889 | (CGG)5ctgg..(TTC)5 | CDS | GAAACAAAGTAGACTACCTGCC | AGATCACAAAGCTACATCATCA | 54 | 54 | 209 | serine acetyltransferase, *Arabidopsis* | | CA158306^B^ | UGSuM890 | (CT)6att...ac(GAG)6 | CDS | TTTGAAGGTGAGAAGGGTG | TGATAACTTGCTGCTTGATTT | 56 | 55 | 323 | mitogen activated protein kinase 6, *Zea mays* | | CA135416^B^ | UGSuM891 | (AGC)5agac..(AGAC)6 | 5'UTRs | CAGTCTAACGAATAAACCTTCC | GATTACGGGAGAGCTATACTTG | 54 | 54 | 353 | delta tonoplast intrinsic protein TIP2, *Arabidopsis* | | CA250930^B^ | UGSuM892 | (ACC)5gccgc..(GA)7 | CDS | AGTGTATAGTTGAAGGAGGTGG | AACTTTGTACAGATCGCATGTA | 55 | 55 | 177 | hypothetical protein, *Oryza* | | CA244508^B^ | UGSuM893 | (AC)16tcat(AG)13 | 3'UTRs | CGATACTCTCATCAAAGGAACT | TCCAAAGTGAAAGTGTCAATAG | 55 | 54 | 368 | hypothetical protein, *Oryza* | | CA124277^B^ | UGSuM894 | (GT)6tga...tgt(AC)17 | CDS | ATCCTTTGTCGTCTCCGT | AGTTGGGTGTGTATTTGGTG | 55 | 55 | 286 | unknown protein, *Arabidopsis* | | CA113958^B^ | UGSuM895 | (CGG)5tatcg..(CGG)5 | CDS | CGATACTCTCATCAAAGGAACT | AAACACATTGATTTGCTTACAA | 55 | 55 | 263 | mRNA cap methyltransferase | | CA229576^B^ | UGSuM896 | (AAG)5agga..(AGC)25 | CDS | GGTTAATCATAGGTGGAATCAG | TGTTTGTCACAATTTATGAATGA | 55 | 55 | 312 | expressed protein, Oryza | | CA237454^B^ | UGSuM897 | (CGC)6cgt...ga(GAG)7 | 3'UTRS | GCAACAATAAAGGATACACTGA | TAACAGGGAGGTTCTGCTCT | 54 | 56 | 273 | mitochondrial inner membrane translocase, *Arabidopsis* | | CA238868^B^ | UGSuM898 | (CGT)5cgc...aa(GCG)9 | 3'UTRS | CTGTCTCGTGGGTCCGTG | ACTCTTCTTCCGCTATTGAAC | 61 | 55 | 175 | DNA-binding protein, *Catharanthus roseus* | | CA218312^B^ | UGSuM899 | (TAGGAT)5tag…c(GAG)5 | 3'UTRS | GACAAGAAGATGCTGAAGAAA | TCTCAATGGTCGTCGGAG | 54 | 58 | 399 | unknown protein, *Oryza* | | CA218312^B^ | UGSuM900 | (TAGGAT)5tggag..(GAG)5 | 5'UTRs | TTATTCTCCTCTCCACCAAAC | AGGACTCCTCTATCTTAGGCTT | 55 | 54 | 376 | hypothetical protein, *Oryza* | | CA113534^B^ | UGSuM901 | (TGC)5agctga..(TCG)6 | CDS | AACATGGCCTATCTCAAGTATC | TGTACTTTGCTCGCTTCTAAC | 55 | 55 | 236 | expressed protein, *Arabidopsis* | | CA153249^A^ | UGSuM902 | (CT)12t(TC)9(CTC)5 | CDS | TGTTTGTTCTGTTCTGTTTGAT | AAGATTCATGCCACTTCAAC | 54 | 55 | 296 | hypothetical protein, *Oryza* | | CA084806^A^ | UGSuM903 | (TA)9(AC)37 | CDS | GCCGCGTCGACTTTGATTCT | GTATCTCACGTGCTATCTTCG | 64 | 55 | 390 | minichromosomal maintenance factor, *Arabidopsis* | | CA122624^A^ | UGSuM904 | (GA)16(GAG)8 | CDS | CTGTGTTCACCAAGTTAATGAG | ACTCGATGAGAGAAAGATTGTT | 54 | 54 | 360 | hypothetical protein, *Oryza* | | CA224348^B^ | UGSuM905 | (GCT)7gcctc..(GC)6 | 3'UTRs | TTCTCAATCTGATTCATTCTCC | CAATCAACTGGTAATCAATACG | 55 | 54 | 329 | hypothetical protein, *Oryza* | | CA254519^B^ | UGSuM906 | (AG)16gacagag(CGC)5 | CDS | GAACAAACACACTACCTTCCC | GTGTTTGACATCAGTGGTCTTA | 56 | 55 | 188 | armadillo/beta-catenin repeat protein, *Arabidopsis* | | CA266356^B^ | UGSuM907 | (CTC)6ggc...g(GGC)7 | CDS | CATCCGACATCCTGGTGG | CTAATGCCCTGTTGTGTTG | 60 | 55 | 399 | protein phosphatase 2A catalytic subunit, *Oryza* | | CA300341^B^ | UGSuM908 | (GCA)5tct...gaa(GAG)6 | CDS | ATACACTGCGGATTAGACCTG | CACCTCTTCCCTTCCTTG | 56 | 55 | 140 | expressed protein, *Oryza* | | CA110600^B^ | UGSuM909 | (TG)6cgcgt..(CA)6 | CDS | CAACGTTGTCTTCTCGGGTT | TAATTTGTTAAAGGCAGGATCT | 60 | 54 | 309 | Transcription initiation factor IIE, *Arabidopsis* | | CA213265^B^ | UGSuM910 | (CT)8gcg...tc(CCG)6 | CDS | CGATGAGACATAAGAGTTCCA | CGGAGGTTGAAGACAAGA | 55 | 54 | 111 | SOH1 family protein, *Arabidopsis* | | CA064962^B^ | UGSuM911 | (GCA)6cat...at(GCA)6 | CDS | CTGATGTATTTGGCTATGGAA | AGCAATCCTTTGACCCAG | 55 | 56 | 121 | CASK-interacting protein, *Oryza* | | CA082696^B^ | UGSuM912 | (GGA)5aca...gtc(CGG)6 | CDS | CTCTCTCTCCCTCTCCGT | CACAGACGAACAATCCATC | 54 | 54 | 358 | AUX1-like permease, *Oryza* | | CA175819^A^ | UGSuM913 | (GAGAG)7(GAG)5 | CDS | TGACTCATCAGAAGACACATTT | AGAACAGAGTTAACTGGGATAGA | 54 | 54 | 148 | 3-deoxy-Darabinoheptulosonate-7-phosphate, *Oryza* | | CA207648^B^ | UGSuM914 | (T)10acaac...(ATT)7 | 5'UTRs | AAAGGGACGTTGAACATAATAG | TGGGAACATACACAGCCAAC | 55 | 59 | 307 | hypothetical protein, *Oryza* | | CA279442^A^ | UGSuM915 | (TCG)5(CGACGG)6 | CDS | AACCAATGGTCTCTCACTTAAC | CCTCATAGATCCCTTGTACTGT | 54 | 54 | 240 | TCP-1/cpn60 chaperonin family protein, *Arabidopsis* | | CA279830^B^ | UGSuM916 | (GGC)5caag...c(CAG)7 | CDS | GAGTGGGCTAATGAAGAGAAG | ATCTGTAGTCGTTGTGTGTGA | 55 | 54 | 393 | auxin-responsive protein IAA14, *Arabidopsis* | | CA279830^B^ | UGSuM917 | (GGC)5caag...(CAG)7 | CDS | CTTGTGGTTTGTTTAACTGTTG | ATATAAGGCAAGAGAGGTCAAA | 55 | 54 | 215 | hypothetical protein, *Oryza* | | CA286535^B^ | UGSuM918 | (CT)7tctcca..(CCG)5 | 5'UTRs | GGGTTTACAACAATCAGTTCTT | TTGATATCATCTAAGCTCCACA | 55 | 55 | 361 | 2-oxoglutarate carrier protein, *Oryza* | | CA150718^B^ | UGSuM919 | (AT)6(TA)7ttt(G)19 | CDS | GAGTTCCAAGAAAGCAAACA | GTAGCATCACACCTCAATCC | 55 | 55 | 364 | F-box protein FBL5, *Oryza* | | CA261119^B^ | UGSuM920 | (GCA)7gctg...cg(GCA)6 | CDS | ATACAAGCAAAGCAACTACCA | GGAGCGTCAGCAACCACT | 55 | 59 | 243 | ribonuclease H1, *Arabidopsis* | | CA084731^B^ | UGSuM921 | (CGC)5cgac...(GAG)8 | CDS | CGATCTCGAGAATCCCAAGT | AGAGAAAGATCAAACCGTACAC | 59 | 55 | 234 | Lipase class 3 protein, *Oryza* | | CA228272^B^ | UGSuM922 | (CGC)5ccc...cg(CGC)5 | 3'UTRS | TGAAAGTGGACAAGCAGATAG | ATGACAATCACGAGGAAGAG | 55 | 55 | 252 | Porin, eukaryotic type, *Medicago truncatula* | | CA103144^A^ | UGSuM923 | (CTC)5(CT)17 | CDS | TCGTCCTCCTTGAGAGCATA | TTCATGAGAGGATCTTTACCTC | 58 | 55 | 164 | delta l pyrroline-5-carboxylate synthase, *Arabidopsis* | | CA265532^A^ | UGSuM924 | (CTC)9(CTCCT)5 | 5'UTRs | GTTCAAGCTGAATTATTTGGA | AACTCACACCAGTAGCATCATA | 54 | 54 | 388 | GTP-binding regulatory protein, *Oryza* | | CA295967^B^ | UGSuM925 | (CT)9gattt(TG)10 | CDS | CTTCTCCCGCTCCTAACC | AACCACTCCCTTCCTCCT | 57 | 55 | 206 | glycosyl hydrolase family 17 protein, *Arabidopsis* | | CA075289^B^ | UGSuM926 | (CCG)5cga...c(AGG)5 | CDS | GCTTACATAGCACCAGCATT | CGTGGCATCTTCTGAGTT | 55 | 54 | 259 | hypothetical protein, *Oryza* | | CA116533^A^ | UGSuM927 | (GAGGA)6(GA)7 | CDS | CCAACAAACTGATTGTGATAGA | TCCAAAGTGAAAGTGTCAATAG | 55 | 54 | 368 | sucrose synthase-2, *Oryza* | | CA134681^B^ | UGSuM928 | (CGG)5actgga..(AGG)5 | CDS | GACAAACCAAACACACATAGAG | GTTTCGTAATCGTACACAGATG | 54 | 54 | 390 | Xyloglucan endotransglucosylase, *Arabidopsis* | | CA279448^A^ | UGSuM929 | (CTC)7(CT)12 | CDS | GCTGTTTCAACAATCTACTCAA | TGCTCATACATGAGATCAAAGT | 54 | 55 | 257 | hypothetical protein, *Oryza* | | CA287173^A^ | UGSuM930 | (CAG)5(CAGCAA)5 | CDS | GAGAGCAAACTAACAATGACAG | ATCGTAATACTCATCGTACCGT | 54 | 55 | 378 | expressed protein, *Oryza* | | CA121070^B^ | UGSuM931 | (CAG)5aggctcag(GCA)6 | 5'UTRs | GAGAGGAACAACAGGATGG | GCACTGGAAGGAGATGTG | 55 | 54 | 318 | oligouridylate binding protein-like protein, *Solanum* | | CA121070^B^ | UGSuM932 | (CAG)5aggct..(GCA)6 | CDS | TATATGCCATGAGAGGACACTA | ATCCTTCACTGTCCAACTTATC | 54 | 54 | 274 | SNF2 domain-containing protein, *Oryza* | | CA266158^A^ | UGSuM933 | (AAG)7(AG)11 | CDS | ACCGACCAGAAGAAATGAAA | TAAATATCGAGGAGTTGAAACC | 57 | 55 | 228 | hypothetical protein, *Oryza* | | CA130851^B^ | UGSuM934 | (CCG)5ctga...cg(CAA)5 | CDS | CATCTCAATCTTCCCTTCACT | AACAACCCTTTAGTTCCAATC | 55 | 54 | 238 | GDA2 protein, *Oryza* | | CA130851^B^ | UGSuM935 | (CCG)5ctgac...(CAA)5 | CDS | CGAATCTGGAAAGAGAGTAAAC | TCTTGCAGCCACTAAATAGTAA | 54 | 54 | 231 | Secretory carrier membrane protein, *Arabidopsis* | | CA088773^B^ | UGSuM936 | (GCG)5gg(GGA)7 | CDS | CAACACAAACCAAAGTTCA | CTATTGCTCCCTTCGCTAC | 52 | 55 | 122 | subtilase family protein, *Arabidopsis* | | CA221686^B^ | UGSuM937 | (GGT)6ggaca(TGG)5 | 3'UTRS | TACATTTCCACAAGCCAAA | GCTCAAGGTCAACAAGACA | 55 | 54 | 294 | unknown protein, *Oryza* | | CA288598^B^ | UGSuM938 | (TC)6ttccaccg(CT)9 | 3'UTRs | AAACAGAATTGCAGCCTTTAT | GACATGAAACTTTGTTGATCTG | 55 | 54 | 148 | expressed protein, Oryza | | AY596572^A^ | UGSuM939 | (GAA)8(AG)7 | CDS | CCGAAGAAGATCGTAGAATTAG | GTTGTTAGCAGCATCTTCTTTC | 55 | 56 | 350 | 60S ribosomal protein L7a, *Arabidopsis* | | CA254910^B^ | UGSuM940 | (CGG)5ctacccg(GGC)5 | 5'UTRs | GCCGCGTCGACTTTGATTCT | GTATCTCACGTGCTATCTTCG | 55 | 57 | 363 | Chlorophyll a/b-binding protein CP24, *Arabidopsis* | | CA279436^A^ | UGSuM941 | (CCT)5(CT)12 | CDS | AGATAACATACAAGCCCACATC | CTGTGGTCAGAAACTCATACAC | 55 | 54 | 310 | mitochondrial L-galactono-1,4-lactone, *Arabidopsis* | | CA248142^A^ | UGSuM942 | (GCA)6(ACA)6 | CDS | CAGCACACAATCAGACAGTAA | GTCGTCGTTCGTCATAGGG | 54 | 59 | 335 | auxin response factor 7a, *Oryza* | | CA267860^A^ | UGSuM943 | (GAGGA)5(GAG)5 | 5'UTRs | GAAACAAAGTAGACTACCTGCC | AGATCACAAAGCTACATCATCA | 54 | 54 | 209 | expressed protein, *Arabidopsis* | | CA158557^A^ | UGSuM944 | (TG)7(TA)10 | CDS | TCACTCGTCAGTTCCATCTC | CATAGTTAGTAGCGTCCCGT | 56 | 54 | 335 | potassium transporter, *Arabidopsis* | | CA066273^A^ | UGSuM945 | (CCA)5(GCA)6 | CDS | CAAATAAAGTAGCGGAAGCA | CAAGACAATCAAGACCAATGT | 55 | 55 | 384 | unknown protein, *Oryza* | | CA085484^B^ | UGSuM946 | (AG)7cga...cc(A)10 | CDS | CAACACAAACCAAAGTTCA | CTATTGCTCCCTTCGCTAC | 52 | 55 | 122 | hypothetical protein, *Oryza* | | CA091719^A^ | UGSuM947 | (CT)6(CGC)7 | CDS | TTGAAGCCTGACCCGAAG | TAAGACCAAATACCACCCTG | 59 | 55 | 392 | ATP-dependent RNA helicase, *Arabidopsis* | | CA171622^A^ | UGSuM948 | (GCC)6(ACC)5 | CDS | ATTTAGGGTTTGTTCGCC | ACTTCATCCTTTGGTTCCTT | 55 | 55 | 370 | hypothetical protein, *Oryza* | | CA181623^A^ | UGSuM949 | (GCC)6(ACC)5 | CDS | GAGAGCAAACTAACAATGACAG | ATCGTAATACTCATCGTACCGT | 54 | 55 | 378 | senescence-associated protein, *Arabidopsis* | | CA209768^A^ | UGSuM950 | (TG)8(AG)8 | CDS | GAGAGCATCCACAACATCA | ATTCCAAGACAAGGGTCGG | 55 | 59 | 364 | cytochrome B561-related protein, *Beta vulgaris* | | CA213390^A^ | UGSuM951 | (GCG)6g(CCT)5 | 3'UTRS | AAGAAGTCATCGCTTGTTCTAA | GAGAGAAGCTAAGAAGTCATCG | 56 | 55 | 397 | Elongation factor 1-gamma, *Oryza* | | CA282602^B^ | UGSuM952 | (GA)9acc(AT)10 | CDS | ATCTGTCACTCAAGGCTAATG | AATCACAAGACTACTACAAGAGA | 54 | 50 | 333 | Thioredoxin fold, *Medicago truncatula* | | CA090532^A^ | UGSuM953 | (GAA)5(GGA)5 | CDS | CGATACTCTCATCAAAGGAACT | AAACACATTGATTTGCTTACAA | 55 | 55 | 263 | hypothetical protein, *Oryza* | | CA115967^A^ | UGSuM954 | (AG)6(GAT)6 | CDS | TCCGTTCGTAGTATCTCTTTCT | CAGGATGGTTGATTAGAGTACC | 55 | 55 | 373 | hypothetical protein, *Oryza* | | BU103688^A^ | UGSuM955 | (CCT)5(CT)7 | CDS | CTCCTTGCTTTCACCCTT | AGACTTCCTTCACAATCTCATC | 55 | 55 | 118 | histone acetyltransferase complex, *Zea mays* | | BU118687^A^ | UGSuM956 | (CCT)5(CT)7 | CDS | CTAATGTCGACTTGGTAATTCA | ATCTGGAGCACCAGGATAAC | 55 | 55 | 361 | unknown protein, *Arabidopsis* | | CA278301^A^ | UGSuM957 | (GTT)5(TA)12 | 3'UTRs | CATCCATTTCGAATTATTACCT | ACTTTCTGATAAGCCAGTCAAC | 55 | 55 | 378 | adhesion of calyx edges protein, *Arabidopsis* | | CA170610^A^ | UGSuM958 | (GAG)5(G)10 | CDS | AAACAGAATTGCAGCCTTTAT | GACATGAAACTTTGTTGATCTG | 55 | 54 | 148 | rreversibly glycosylated polypeptide, *Arabidopsis* | | CA206176^A^ | UGSuM959 | (CT)11(CCA)5 | CDS | AGAAGGAACCAGGATAGAGAAT | AAGTAAGAAAGACGAACTCTGC | 55 | 54 | 196 | CMP-KDO synthetase, *Zea mays* | | CA271179^A^ | UGSuM960 | (AC)11(CT)7 | CDS | GGGTTTACAACAATCAGTTCTT | TTGATATCATCTAAGCTCCACA | 55 | 55 | 361 | homeo domain Leu zipper protien, *Oryza* | | CA179434^A^ | UGSuM961 | (TA)10(TG)6 | 3'UTRs | CATTTCAGTCCCTCATCTAACT | ATCGTAGCTTATGAACTGTCCT | 55 | 54 | 219 | glucose-6-phosphate isomerase, *Arabidopsis* |     ^A^Compound non-interrupting microsatellite motifs  ^B^Compound interrupting microsatellite motifs  ***UGSuM stands for unigene derived sugarcane microsatellite primers**   |  |  |  |  |  |  |  |  |  |  | | --- | --- | --- | --- | --- | --- | --- | --- | --- | --- | |  |  |  |  |  |  |  |  |  |  |
