# Supplementary material for: Functionally relevant microsatellites in sugarcane unigenes
Source: BMC Plant Biol. 2010 Nov 17;10:251. doi: 10.1186/1471-2229-10-251 (PMC3017843; doi:10.1186/1471-2229-10-251)
Supplement: Additional file 5 — Alignment showing the presence of class I UGMS repeat-motifs in sugarcane species, related genera and five cereal species. [file 1471-2229-10-251-S5.DOC]

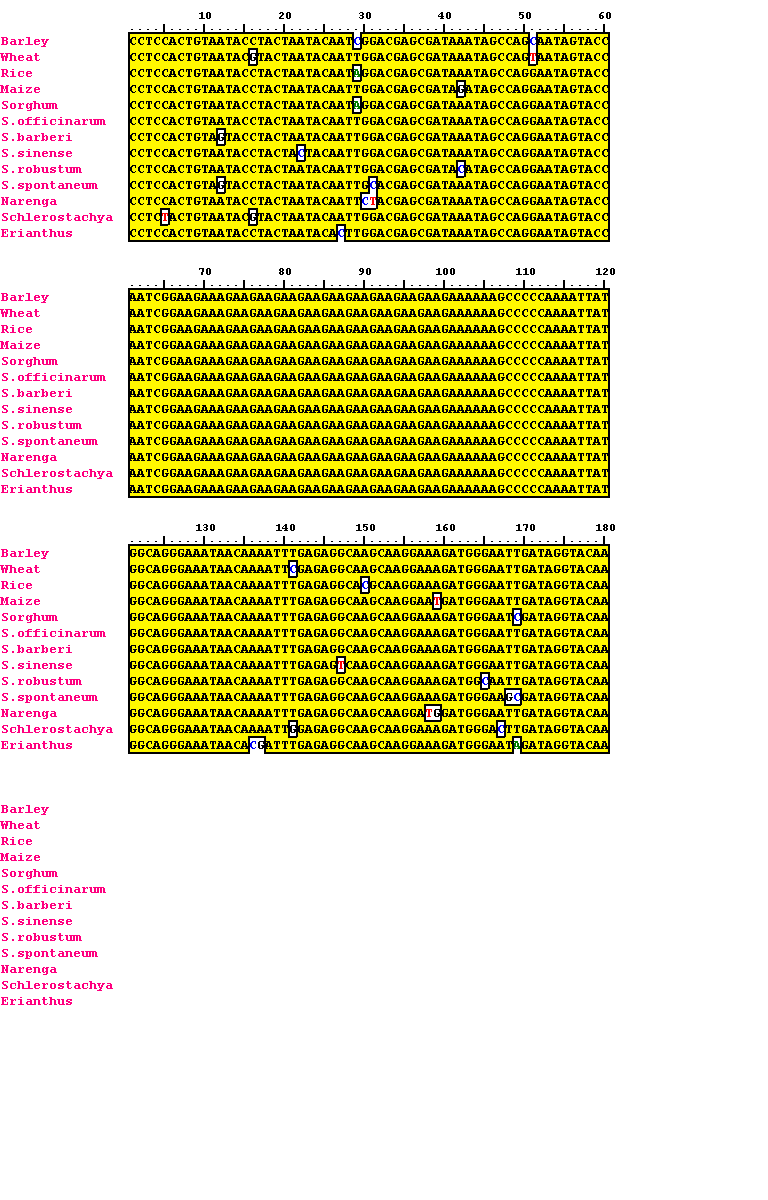


**(AAG)10**

**Additional file 5: Alignment showing the presence of sugarcane class I microsatellite repeat-motif in sugarcane species, related genera and five cereal species. This primer was designed from the unigene for soluble acid invertase containing (AAG)_10_ microsatellite motifs. The unigene sequences flanking the microsatellite repeat motifs are conserved significantly, however the presence of SNPs in the flanking unigene sequences are highlighted.**
