# Supplementary material for: Functionally relevant microsatellites in sugarcane unigenes
Source: BMC Plant Biol. 2010 Nov 17;10:251. doi: 10.1186/1471-2229-10-251 (PMC3017843; doi:10.1186/1471-2229-10-251)
Supplement: Additional file 6 — In silico polymorphism of sugarcane UGMS loci at sequence level in five cereal species. [file 1471-2229-10-251-S6.DOC]

**Additional file 6: *In silico* polymorphism of sugarcane UGMS loci at sequence level in five cereal species**

|  | Sugarcane (810 UGMS primers) | | |
| --- | --- | --- | --- |
| Cereal species | **Putative BLAST-hits (bit score ≥500) of UGMS and showing significant flanking sequence homology (%)** | **Unigenes with variable microsatellite repeat-motifs and showing *in silico* polymorphism (%)** | **Unigenes with similar microsatellite repeat-motifs (%)** |
| *Sorghum* | 517 (63.8) | 92 (17.8) | 425 (82.2) |
| Maize | 495 (61) | 127 (25.6) | 368 (74.4) |
| Rice | 430 (53) | 158 (36.7) | 272 (63.3) |
| Wheat | 379 (46.8%) | 161 (42.5) | 218 (57.5) |
| Barley | 350 (43.2%) | 163 (46.6) | 187 (53.4) |

|  |  |  |  |  |  |  |  |  |  |  |
| --- | --- | --- | --- | --- | --- | --- | --- | --- | --- | --- |
