# Supplementary material for: Functionally relevant microsatellites in sugarcane unigenes
Source: BMC Plant Biol. 2010 Nov 17;10:251. doi: 10.1186/1471-2229-10-251 (PMC3017843; doi:10.1186/1471-2229-10-251)
Supplement: Additional file 7 — Functional annotation of 672 unigenes carrying microsatellites of sugarcane. [file 1471-2229-10-251-S7.DOC]

**Additional file 7: Functional annotation of 672 unigenes carrying microsatellites of sugarcane. These were related to various predicted important cellular and biological functional genes, which corresponded maximum to sugar metabolic enzymes (39%) followed by structural proteins (26%), transcription and translation regulators (18%) and minimum to disease resistance genes (4%).**

**Supplementary Figure 4:** Alignment showing the confirmation of the presence of sugarcane class I microsatellite loci in five cereal species and sugarcane species and related genera designed from the unigenes for auxin-dependent growth promoter containing (CTCTCC)_5_ microsatellite motifs. The sequence information based on auxin-dependent growth promoter showed the presence of varied UGMS motif sequences like that of (CTCTCC)_5_, (CTCTCC)_3_ and (CTCTCC)_2_ particularly in the divergent lineages namely, wheat, barley and three related genera of sugarcane. The unigene sequences flanking the UGMS repeat motifs are conserved significantly, however the presence of SNPs and InDels in the microsatellite repeat motifs and flanking unigene sequences are highlighted in the genotypes. The high quality sequences for all the species were aligned using CLUSTALW multiple alignment tool.
