# Supplementary material for: Functionally relevant microsatellites in sugarcane unigenes
Source: BMC Plant Biol. 2010 Nov 17;10:251. doi: 10.1186/1471-2229-10-251 (PMC3017843; doi:10.1186/1471-2229-10-251)
Supplement: Additional file 8 — Primers targeting the microsatellite repeats present in the functional domains of proteins encoded by the unigenes. [file 1471-2229-10-251-S8.DOC]

|  |  |  |  |  |  |  |  |  |  |  |
| --- | --- | --- | --- | --- | --- | --- | --- | --- | --- | --- |
| **Additional file 8: Sugarcane UGMS primers targeting the microsatellite repeats present in the functional domains of proteins encoded by unigenes**   | **Unigene** | **UGMS primers*** | **Microsatellite**  **repeat-motifs** | **Forward primer (5'-3')** | **Reverse primers (5'-3')** | **TM** | **Product Size** | **Pfam IDs** | **Accession** | **Characteristics** | | --- | --- | --- | --- | --- | --- | --- | --- | --- | --- | | CA127223 | UGSuM3 | (TA)36 | AAGAAGAGCCGTAGAAACAAC | ATTGAGCGAGGGATGAAC | 55 | 227 | Cellulose_synt | PF03552 | Cellulose synthase | | CA278792 | UGSuM5 | (TA)28 | TCACATCCATCATCCACAGC | TCCAATGCAAGCAAACTCAC | 55 | 200 | Cyclin | PF08613 | Cyclin | | CA226611 | UGSuM6 | (AT)28 | ACTGACACACACGCACAC | TGGAAGTGAATGAAGCGA | 54 | 279 | Opi1 | PF08618 | Transcription factor Opi1 | | CA206100 | UGSuM10 | (AG)22 | ACCCTTCCCATTCCCATC | CTCCAGGTTCGCCACCAC | 59 | 287 | Cytochrom_B_C | PF00032 | Cytochrome b(C-terminal) | | CA130288 | UGSuM12 | (GA)20 | TTGACACAGATAAACACCACA | ATCACTTGCCCTCCCTTC | 54 | 307 | TBCA | PF02970 | Tubulin binding cofactor A | | CA278282 | UGSuM16 | (AT)18 | GCGTCTTCATCATCTGCAAC | TAGAGAGACATGGGGTGCAT | 56 | 282 | NB-ARC | PF00931 | NB-ARC domain | | CA253277 | UGSuM17 | (AG)18 | TTTCCATTCTTCCATTCAACTG | GGCAGGCTGAGAGACTGTTC | 55 | 300 | Pkinase | PF00069 | Protein kinase domain | | CA227482 | UGSuM18 | (GA)18 | GGCGAGAGAGAGAGAGAGAGAG | AGGTGGAGATCTTGAGGTAGGC | 55 | 200 | GRP | PF07172 | Glycine rich protein family | | CA126180 | UGSuM20 | (TCA)12 | ATCCCTTATGCTACAGAAATGT | TTAGCCTAGAGGTTTGATTGAT | 54 | 159 | ACBP | PF00887 | Acyl CoA binding protein | | CA177414 | UGSuM21 | (AGGA)9 | CGCTCCCTCACCGTCATT | CTCCGCATCCTCGTCACC | 62 | 219 | TBP-binding | PF09247 | TATA box-binding protein binding | | CA073284 | UGSuM27 | (GGC)11 | CTGCAGTACGGTCCGGAATC | GTACCACCATGGCTCTAGCTTC | 55 | 180 | RBFA | PF02033 | Ribosome-binding factor A | | CA180693 | UGSuM28 | (CTG)11 | CTGAAACAGCAAACCTGAA | AGAGCCATTGAAAGAGATTG | 54 | 390 | zf-C2H2 | PF00096 | Zinc finger, C2H2 type | | CA248458 | UGSuM29 | (AAAG)8 | ATTACTTGCCTGTCTTGAATG | GATGTTTGGACCTTGAACC | 54 | 394 | Pkinase | PF00069 | Protein kinase domain | | CA268640 | UGSuM34 | (AAG)10 | TTACAAATGTAGCCTTGCCTTG | ATCTTTCCTTGCTTGCCTCTC | 63 | 150 | CBM_20 | PF00686 | Starch binding domain | | CA101287 | UGSuM38 | (GCC)10 | GTAGTCGCGTGCGCTCTGTCTG | CAGTAATGTTGTCAACTTGAGTCT | 67 | 347 | Methyltransf_11 | PF08241 | Methyltransferase domain | | CA103857 | UGSuM39 | (TG)15 | TAAATATGGTGGAGCAAAGTATTA | ACTAGAGCTCTTAAATTCCACAGT | 65 | 385 | GST_C | PF00043 | Glutathione S-transferase | | CA228772 | UGSuM47 | (GCC)10 | ATTTATGGAGGAAGAAACGG | ATTACAAACAAGAAGAGCGG | 55 | 223 | Trs120 | PF08626 | Transport protein Trs120 | | AY596599 | UGSuM51 | (CAC)9 | TACTATAATGATAGATCTCCTCCG | GTAATAGGACTGGATTGGAATG | 53 | 303 | ubiquitin | PF00240 | Ubiquitin family | | CA113942 | UGSuM52 | (GGC)9 | CTTCTTCTTCTTCTTCTTCTTCTG | AAGAATCGAAGGTTAACAACAG | 55 | 224 | Ribosomal_60s | PF00428 | 60s Acidic ribosomal protein | | CA074283 | UGSuM54 | (GGA)9 | GAAGGAGAGTTAGGAGAGTCAGT | GACCTCTTCTTGTCTAATACCCT | 55 | 173 | Aminotran_1_2 | PF00155 | Aminotransferase class I and II | | CA200013 | UGSuM56 | (CGG)9 | TAATACTTTCACCAGCCAA | GGAGCAGCAACGCACAGG | 51 | 239 | QRPTase_N | PF02749 | Quinolinate phosphoribosyl transferase | | CA205971 | UGSuM57 | (GTT)9 | CTACAGACGACGACAGGTATG | TAGGAAGGAACACAGGGCAG | 55 | 174 | AcetylCoA_hydro | PF02550 | Acetyl-CoA hydrolase/transferase | | CA261837 | UGSuM59 | (TTG)9 | AGGAGGACTACGAGGAGAAG | AACAAGAACAATCACAAGGAA | 55 | 334 | ADH_zinc_N | PF00107 | Zinc-binding dehydrogenase | | CA276055 | UGSuM61 | (CAC)9 | ACATCCTTCCCACGCCAG | CCTACTCCTCCTCCTCCTC | 62 | 100 | Chitin_synth_1 | PF01644 | Chitin synthase | | CA279855 | UGSuM62 | (CAA)9 | ATCTGCCATCAGGCTAAA | TGAGGAATCAGTCTTGTGTAA | 54 | 176 | GFO_IDH_MocA_C | PF02894 | Oxidoreductase family | | CA131357 | UGSuM63 | (GA)13 | CCGTCCTCTCTTATATATGCAC | GAGCTGCCGTAGTAGTTCTG | 53 | 400 | Alpha-amylase | PF00128 | Alpha amylase catalytic domain | | CA196900 | UGSuM70 | (GAG)8 | TAATATACATCACGACACAACAAA | TTGTGTTATCTAGACGATACCTTT | 55 | 348 | ChiC | PF06483 | Chitinase C | | CA248131 | UGSuM72 | (ACC)8 | ATCGATCATTTCCTCTTCTTAGTA | AATTTGATTCATAGGATTGTGAG | 55 | 383 | Lipoprotein_6 | PF01441 | Lipoprotein | | CA120241 | UGSuM79 | (AGG)8 | AACTTGTGGTTCCTAGCTGTTT | AATCCTTACCTTAGAAGATCACC | 55 | 365 | Nucleoside_tran | PF01733 | Nucleoside transporter | | CA130602 | UGSuM80 | (TCC)8 | TATCAAGAAACAGACAACCCA | AGACTCCAAAGATGGTGAACT | 55 | 318 | FSH1 | PF03959 | Serine hydrolase (FSH1) | | CA157740 | UGSuM83 | (GA)12 | GTTCACCACCTCCGACAC | TAGCAGCAACCTCACACTC | 57 | 351 | CBS | PF00571 | CBS domain | | CA185016 | UGSuM86 | (CGC)8 | ATGACAGCAGCACAATGA | CACCCAGTTGAATAAGTGA | 54 | 285 | MRP-L46 | PF11788 | 39S mitochondrial ribosomal protein L46 | | CA195435 | UGSuM88 | (GA)12 | CACTTCCCAGAGACCCAG | GACCTTAGCAATCAAGACAGA | 56 | 287 | zf-C2H2 | PF00096 | Zinc finger, C2H2 type | | CA196900 | UGSuM89 | (GAG)8 | CTCCCAAAGCAAACCCTT | GTTCTTGACCTTCTTCCTGTC | 57 | 352 | Galactosyl_T | PF01762 | Galactosyltransferase | | CA228375 | UGSuM91 | (CGC)8 | GTGTGGAACTGTGGATGG | ACCGAAACAACAAGTAAACAA | 55 | 348 | Npa1 | PF11707 | Ribosome 60S biogenesis N-terminal | | CA251651 | UGSuM92 | (CGC)8 | TACTATGGAGGCGGGAGG | TAGAAGAGCACAGAGCAAAC | 58 | 297 | GSHPx | PF00255 | Glutathione peroxidase | | CA261154 | UGSuM93 | (TGT)8 | CAAGTCGTCCTCTTCGTC | ATTAGACAGGTAAATCGTGCC | 54 | 217 | RVT_1 | PF00078 | Reverse transcriptase | | CA261182 | UGSuM94 | (ACA)8 | GAGTCAGGGAGAGGAACAG | TAGATACCGAGCACACCAG | 54 | 238 | Rrf2 | PF02082 | Transcriptional regulator | | CA101958 | UGSuM99 | (CT)11 | ACTAACTCTCTTCAACTTCCTCTG | AGCTGTTCCTCTTTAGCTAGTTC | 60 | 294 | Lipase | PF00151 | Lipase | | CA280782 | UGSuM107 | (TGC)7 | AATCGGCGCTGACCATGGACTC | AGAACACAACTTTCACCTTGTT | 54 | 276 | S-AdoMet_synt_C | PF02773 | S-adenosylmethionine synthetase | | CA276184 | UGSuM111 | (GTC)7 | AGAAGGTGATCCTCAAGGACAAG | AACTGATCCCTCTTTCATATATTC | 61 | 387 | Lectin_legB | PF00139 | Legume lectin domain | | CA179488 | UGSuM113 | (GGC)7 | TATCTGATCGGTAGCAAATAGC | GTGGTTAAGAAGAGACTAAGTTCG | 56 | 370 | F1F0-ATPsyn_F | PF10791 | Mitochondrial F1-F0 ATP | | CA282680 | UGSuM120 | (ATT)7 | GTTTCCTTCCTTCTCGTGCCAT | CCTAATAAGTATCGTTGAAGGTG | 63 | 331 | TK | PF00265 | Thymidine kinase | | CA204927 | UGSuM122 | (GCC)7 | ATAGAGATAGAGGGAGTATGCTTG | AGTCTCATTCTCATCAGAAACC | 55 | 317 | NO_synthase | PF02898 | Nitric oxide synthase | | CA236932 | UGSuM124 | (GCC)7 | GTGAGATTCCGATTCCGCTTCC | ACATGAGTATTGTACTGGTGAGAA | 66 | 389 | Ribosomal_60s | PF00428 | 60s Acidic ribosomal protein | | CA106018 | UGSuM125 | (GCG)7 | ACGAGTTCAGGGCGCTGATAGAG | ATCACGACGTCATAGTCCGTAAC | 66 | 166 | bZIP_2 | PF07716 | Basic region leucine zipper | | CA149322 | UGSuM135 | (CGC)7 | GTCCTAGCAGGTCTAGGTCTTG | AAATCTGAGCTAGATCCTCTCC | 55 | 112 | Ufd2P_core | PF10408 | Ubiquitin elongating factor core | | CA215720 | UGSuM136 | (GCC)7 | AAGAACATCATGTCGGTGCAGT | GTAAGCTCTTGGTAACATTCTTG | 53 | 273 | Npa1 | PF11707 | Ribosome 60S biogenesis | | CA177425 | UGSuM137 | (GCA)7 | GTTCTTAGTCCAGCCGTAGTTGT | TCAAATTTCTCAGAACCTTCAC | 54 | 363 | Orn_Arg_deC_N | PF02784 | Pyridoxal-dependent decarboxylase | | CA090548 | UGSuM142 | (GCC)7 | CTCTGTTGTCATAAGAAGAGACAC | CTTTGATTCAGCAGCATAAACT | 54 | 399 | V-ATPase_G | PF03179 | Vacuolar (H+)-ATPase | | CA094967 | UGSuM145 | (CAG)7 | CCATCTTCATGACAACCTCT | TATCGATCCATCCCTATACATC | 57 | 114 | Calmodulin_bind | PF07887 | Calmodulin binding protein-like | | CA099491 | UGSuM146 | (GCA)7 | CTGCTGTTCCTCTTATTGCTCC | GTTAGTAGCAGCACTCATCTAGC | 66 | 400 | NAC | PF01849 | NAC domain | | CA112979 | UGSuM149 | (AGC)7 | GTTCAATCAAATCCCTCTCCTC | AGCTTGGTCAGCTCCTCATCGTT | 65 | 394 | UQ_con | PF00179 | Ubiquitin-conjugating enzyme | | CA116368 | UGSuM150 | (GGC)7 | ACACTGACCGATGGATCCTCTT | ATCAACGTGGACCAGATCTTCTT | 65 | 243 | Acid_phosphat_B | PF03767 | HAD superfamily | | CA168020 | UGSuM163 | (GGC)7 | CTGGCAACTTACAGCACC | CCACCTTCAGACAAATACAGA | 55 | 286 | zf-C2H2 | PF00096 | Zinc finger, C2H2 type | | CA182783 | UGSuM164 | (AAG)7 | AACAAGGTAGATGATGCCAA | GAGTGGGTTCCGTGGTTC | 55 | 393 | UBA_2 | PF08587 | Ubiquitin associated domain | | CA182808 | UGSuM165 | (GCC)7 | GAACCACGGAACCCACTC | CTACGACCACCAGTCACAC | 58 | 158 | RdRP | PF05183 | DNA dependent RNA polymerase | | CA213291 | UGSuM171 | (CGG)7 | GAGGAGAAGAAGGAGGAGG | TATTTCCCACCAACAAGCA | 55 | 228 | NMN_transporter | PF04973 | Nicotinamide mononucleotide transporter | | CA252244 | UGSuM174 | (CGC)7 | CCCTTTCTCTTCTGTCCTTG | CTGTGAGTGTCCCGCTTG | 56 | 295 | Cenp-F_leu_zip | PF10473 | Leucine-rich repeats of kinetochore protein | | CA244023 | UGSuM186 | (AG)10 | AACATTTCGGCATTTGAAGC | GGTCTTTCTTGGGGATCTCTC | 55 | 160 | UBA_2 | PF08587 | Ubiquitin associated domain (UBA) | | CA231668 | UGSuM187 | (CT)10 | CAACAATTGTCGAAGCCTCTC | TTTGCTTACCCCCTGTTGAC | 56 | 500 | OSCP | PF00213 | ATP synthase delta (OSCP) subunit | | CA066249 | UGSuM194 | (TCGG)5 | ACTTAGTCTCTTCTTAACCACTGC | AAGTAATTATCTATAGTGCCACCC | 55 | 178 | FAD_binding_1 | PF00667 | FAD binding domain | | CA213297 | UGSuM200 | (CTCC)5 | TTCATCCACAAGGACAAGAC | ACCGTTACCATAGCATACAAA | 55 | 178 | TBCC | PF07986 | Tubulin binding cofactor C | | CA282658 | UGSuM206 | (CACG)5 | ACCGTCACCAGCAACAAC | ATTAGCAGCCTTCAGCATAG | 57 | 271 | KAP | PF05804 | Kinesin-associated protein (KAP) | | AJ969049 | UGSuM207 | (CCTT)5 | AGAAGGTGATCCTCAAGGACAAG | AACTGATCCCTCTTTCATATATTC | 61 | 387 | GlcNAc | PF11397 | Glycosyltransferase (GlcNAc) | | AY644469 | UGSuM208 | (GGC)6 | ATCGATCATTTCCTCTTCTTAGTA | AATTTGATTCATAGGATTGTGAG | 55 | 383 | IATP | PF04568 | Mitochondrial ATPase inhibitor | | CA074021 | UGSuM214 | (GCG)6 | ACTTCGCCTACACGCTCGATTC | GTCATTTGACTCCTTCAGTTCA | 64 | 380 | bZIP_2 | PF07716 | Basic region leucine zipper | | CA082494 | UGSuM216 | (CCT)6 | GTCATCCTGTTCGACATCGGGTT | TTCTAGGTAAGATACCTTTCAAAC | 66 | 362 | GTPase_binding | PF09027 | GTPase binding | | CA085964 | UGSuM218 | (TGC)6 | ATTACAGGCTTACACTTACAACAA | ATAGCAGCGTGGAGTCCTACTT | 55 | 364 | TAF4 | PF05236 | Transcription initiation factor | | CA086148 | UGSuM219 | (GA)9 | ACCTCCACCTCCACCTCAGTTC | CGTTCAGCTTCAGGGTGTCGAT | 64 | 397 | Stk19 | PF10494 | Serine-threonine protein kinase | | CA093362 | UGSuM221 | (GGC)6 | ATACTTCTCGATTAATCACCGAT | GCTCGGTCATCATCACTACTAC | 56 | 145 | MAP1_LC3 | PF02991 | Microtubule associated protein | | CA096260 | UGSuM222 | (GAG)6 | TAGCAATCTACTCCCTACGTCTAC | GTTGACGTTGATCAGCCCGTTG | 56 | 378 | Img2 | PF05046 | Mitochondrial large subunit ribosomal protein | | CA103708 | UGSuM225 | (CCA)6 | TTTCTTCGTCTCCTCTTCCTCC | ATTCCAGACGAGCTCCAGAATTT | 66 | 190 | Fe-S_biosyn | PF01521 | Iron-sulphur cluster biosynthesis | | CA108086 | UGSuM228 | (TTC)6 | GAGCTTTGCATGATCTCTCGAT | TACTCCTCCCTATACATTGATACA | 56 | 230 | zf-Dof | PF02701 | Dof domain, zinc finger | | CA112580 | UGSuM231 | (GGC)6 | AGTACCATCGCTTAAGTAAAGTCT | ATTAAAGGACCTTACTCAGTTACC | 61 | 113 | zf-C2H2 | PF00096 | Zinc finger, C2H2 type | | CA115807 | UGSuM232 | (AC)9 | CCTTGGTTCGTTTATTCTTTACTA | AAGAGGATCCATCGGTCAGTGT | 62 | 336 | Tbf5 | PF06331 | Transcription factor TFIIH complex | | CA117569 | UGSuM233 | (CGC)6 | CTTGCATGAGCATGAGACAG | TTCTTTGTGTTATTCCAAGTCA | 54 | 226 | SDH_sah | PF01972 | Serine dehydrogenase proteinase | | CA117618 | UGSuM234 | (GCC)6 | CTGAGGTGAAATTATCGTGTGT | GCAACGTCTAAATATAATTGCTAA | 55 | 104 | KIF1B | PF12423 | Kinesin protein 1B | | CA125127 | UGSuM240 | (TGT)6 | AGTTGAAGCCGAGAAAGAA | CGTAGAAGAGCGGGATGT | 55 | 376 | Abhydrolase_2 | PF02230 | Phospholipase/Carboxylesterase | | CA126381 | UGSuM241 | (CTG)6 | ATCGCTAACTCATTCATCATC | GCCAGACGCATTCAAACA | 54 | 348 | START | PF01852 | START domain | | CA128394 | UGSuM242 | (GCA)6 | CATAGCAAGCACCACCTC | TCTTCTTCTCGTCCACCC | 55 | 263 | Ank | PF00023 | Ankyrin repeat | | CA129005 | UGSuM243 | (GCC)6 | GTGTTCGTCTTCCTTGACC | GGCTTTGTAGTTTGCGTATC | 55 | 324 | Nitrate_red_del | PF02613 | Nitrate reductase delta subunit | | CA129620 | UGSuM245 | (GA)9 | ACACCTTTACGACCATCAAC | GAATGAGAACCCAATACCAG | 54 | 375 | NAD_kinase | PF01513 | ATP-NAD kinase | | CA137576 | UGSuM250 | (CGG)6 | CAGAGCATCACCAGCACC | CTTGAGCAGCGTCTTGTT | 58 | 135 | Nop16 | PF09420 | Ribosome biogenesis protein | | CA144976 | UGSuM257 | (CAA)6 | CTGGCTACATTCACAGGATT | GGTCGGTTGAGTTATGATGAG | 55 | 338 | zf-C2H2 | PF00096 | Zinc finger, C2H2 type | | CA147526 | UGSuM258 | (CCG)6 | CACACTGACACCTACCAATGA | GCCAAATACAACGAACGA | 56 | 262 | IF-2 | PF11987 | Translation-initiation factor 2 | | CA150692 | UGSuM260 | (CCG)6 | AATCTGGACTGCTTGGTTC | AGTGTCTTGTTCCTGGTGTC | 55 | 184 | NuA4 | PF09340 | Histone acetyl transferase | | CA167449 | UGSuM274 | (CAG)6 | ACTCTAATGACTCTTCTTCCCA | GTGAATGCTGCTTACTTTGTC | 54 | 308 | NIR_SIR_ferr | PF03460 | Nitrite/Sulfite reductase ferredoxin | | CA179488 | UGSuM283 | (GGC)6 | CTTCTTCCACAAACGCAC | CAGCGAACACAGAGATGTAG | 55 | 341 | MAM33 | PF02330 | Mitochondrial glycoprotein | | CA180693 | UGSuM284 | (GCG)6 | ATCCATTAGGTCTTCCTTCTC | CTACTTCAATCTCCTTGTCCC | 54 | 338 | zf-C2H2 | PF00096 | Zinc finger, C2H2 type | | CA186537 | UGSuM287 | (CGC)6 | GGGTGTGAAGACAACTGAAA | GGACAACAGGGAGAAGAGG | 56 | 293 | zf-C2H2 | PF00096 | Zinc finger, C2H2 type | | CA188285 | UGSuM288 | (CCG)6 | TTAGAACAGGAGAGTGCTTGA | CAGAGTGGGAGTGAGTCGT | 55 | 132 | Lipase | PF00151 | Lipase | | CA191141 | UGSuM289 | (GCG)6 | GTGGGTCGTCTTGTCCTC | AAGGTGTTCCATACAGCAA | 56 | 302 | TRM13 | PF05206 | Methyltransferase | | CA194346 | UGSuM290 | (GCG)6 | TCCTGGTGGCAGTTGTAG | TTGGGCTTTGTGGAGTCA | 55 | 368 | Rxt3 | PF08642 | Histone deacetylation protein | | CA195760 | UGSuM291 | (CCT)6 | ATAAATGTCTTGAGGGTGCT | ACCAAATGCCAGGTGTTC | 53 | 213 | Pkinase | PF00069 | Protein kinase domain | | CA196495 | UGSuM294 | (CGG)6 | CCTTGTGCGTGTCTCTCC | TTCCACTACCCTCTTTGTTG | 57 | 190 | Cad | PF03596 | Cadmium resistance transporter | | CA206115 | UGSuM296 | (CGC)6 | ACCTCCACCTACACCTACTG | GCCATACTACACCTCCAAG | 54 | 386 | KA1 | PF02149 | Kinase associated domain | | CA206236 | UGSuM297 | (GCC)6 | ATGCTCTCTCTCTTCTGTCAA | CATCAGGTCGTAGTGGGA | 54 | 323 | YflT | PF11181 | Heat induced stress protein | | CA213570 | UGSuM301 | (TGC)6 | AACACACACACACACACACAC | ACTAATCTCTCCTTGCTTTGG | 56 | 160 | Nuc_sug_transp | PF04142 | Nucleotide-sugar transporter | | CA234609 | UGSuM309 | (ATC)6 | CGTTCGTCTCTCTCTCCTC | ATTTACAGGTCATCCCAAAC | 55 | 381 | Ribonuclease | PF00545 | ribonuclease | | CA234669 | UGSuM310 | (CTC)6 | ATCGCTTCTACAGTCACCC | AACTTCTCCCTTTCTCCAAC | 55 | 185 | Glyco_hydro_42 | PF02449 | Beta-galactosidase | | CA236932 | UGSuM311 | (GCC)6 | GCTCTCCTCCTCCTCTCC | GCCACTTTATCATCCTCAGTT | 56 | 350 | L31 | PF09784 | Mitochondrial ribosomal protein L31 | | CA253068 | UGSuM322 | (AAC)6 | CTGGCTCTCTATCACCGAC | AGAATCAAATCAACCGCTC | 55 | 330 | LuxC | PF05893 | Acyl-CoA reductase | | CA258249 | UGSuM325 | (TC)9 | CACAACAGGACCAAGATGA | ACTCTCAACGGTATGGCTAA | 55 | 228 | CBM_20 | PF00686 | Starch binding domain | | CA270948 | UGSuM329 | (CGC)6 | CTTCTGATGATGGAGGCA | AGTGTTTCAAGCCAAATCC | 55 | 381 | TF_Zn_Ribbon | PF08271 | TFIIB zinc-binding | | CA276184 | UGSuM334 | (GTC)6 | TCTCAAGGATACACCATCAAG | ATCATCAGCACGACAGACA | 55 | 177 | Lectin_leg-like | PF03388 | Legume-like lectin family | | CA282680 | UGSuM336 | (ATT)6 | AACAACGGATACAAATGAAAG | CGATTGATGGATGGTAATG | 54 | 299 | NB-ARC | PF00931 | NB-ARC domain | | CA283420 | UGSuM337 | (GCT)6 | TCCGTTCAGAGTGATGATG | AAGAAGCCGTGGAGGAAG | 55 | 288 | TAF | PF02969 | TATA box binding protein associated factor | | CA284697 | UGSuM338 | (GCT)6 | TAAGCGAGTGTGAGGGAG | GACGAGGATTTGTTCCAG | 54 | 218 | AAA | PF00004 | ATPase family associated | | CA300679 | UGSuM344 | (CTC)6 | CTATCCTCTTGTTGGGTCCT | TCCGCACCTCCGTTCACC | 55 | 162 | NABP | PF07990 | Nucleic acid binding protein NABP | | CA093455 | UGSuM346 | (AG)8 | TATACGTAGTAGTGATGATGACCG | CTCCTTCGTCCAGTACCAGTAG | 60 | 341 | TMF_DNA_bd | PF12329 | TATA element modulatory factor | | CA204826 | UGSuM357 | (CT)8 | GCTGACTTCGTTCTGACTTAC | TATTGGCTCTGGGATAGACTT | 54 | 300 | Stk19 | PF10494 | Serine-threonine protein kinase 19 | | CA205923 | UGSuM358 | (AC)8 | ACACATCGCTTTCCCACA | GCATACCTGTCGTCGTCT | 58 | 148 | Salt_tol_Pase | PF09506 | Glucosylglycerol-phosphate phosphatase | | CA280557 | UGSuM361 | (GA)8 | GACAGAGAACAGGAATCAACA | TAAAGTCCAACAAGTAAGCCA | 55 | 355 | Tau95 | PF09734 | RNA polymerase III transcription factor | | AJ969049 | UGSuM364 | (CAG)5 | AAACGATCAGATCTAGCACAAT | CTGCTGTCTTAGGTACAGTCTTC | 55 | 299 | Glycos_transf_1 | PF00534 | Glycosyl transferases group 1 | | AM493723 | UGSuM366 | (GTC)5 | ACTTAGTCTCTTCTTAACCACTGC | AAGTAATTATCTATAGTGCCACCC | 55 | 210 | TFIIF_alpha | PF05793 | Transcription initiation factor IIF | | AY596612 | UGSuM370 | (TAT)5 | ACGAGTTCAGGGCGCTGATAGAG | ATCACGACGTCATAGTCCGTAAC | 66 | 166 | OpcA_G6PD_assem | PF10128 | Glucose-6-phosphate dehydrogenase | | AY596613 | UGSuM371 | (TGC)5 | GCCGAAGCCTCTCCTCTCCTCC | GTCATCAATGACAGAGATGTAGAC | 69 | 116 | C6_DPF | PF10170 | Cysteine-rich domain | | BQ536356 | UGSuM373 | (GTC)5 | TAGCAATCTACTCCCTACGTCTAC | GTTGACGTTGATCAGCCCGTTG | 56 | 378 | zf-C2H2 | PF00096 | Zinc finger, C2H2 type | | CA064962 | UGSuM377 | (CCG)5 | GCGCTCACATCACCTCCTAC | GACGATCTTGAAGAACCTGG | 55 | 376 | CorC_HlyC | PF03471 | Transporter associated domain | | CA065736 | UGSuM379 | (CTT)5 | GTCTATCTTCAAGGAAACCAAA | GGAGAAGTACAATGAGCAAGAT | 65 | 268 | Oscp1 | PF10188 | Organic solute transport protein | | CA067775 | UGSuM381 | (GCG)5 | ACTAGTTTGCTGTTGCTGACTG | TCTTGAGATATAAACATGGGAGTA | 56 | 141 | TBP | PF00352 | Transcription factor TFIID | | CA070210 | UGSuM382 | (TTC)5 | ACAGATCACAATCAACCAAACTA | AATGTCGAAATGGAACATTAAC | 56 | 141 | U3_assoc_6 | PF08640 | U3 small nucleolar RNA-associated protein | | CA071318 | UGSuM383 | (AGA)5 | TCGAATTCAAAGTTAAATGTCTT | GATTCTGCTCGGATAGAATACG | 55 | 153 | WRW | PF10206 | Mitochondrial F1F0-ATP synthase | | CA073445 | UGSuM386 | (GGA)5 | GGTTTCGAATTACCGGCCCGAC | GAGCCTCTGTCTAGGCTTTAGG | 69 | 156 | IBD | PF10416 | Transcription-initiator DNA-binding domain | | CA074644 | UGSuM388 | (CGG)5 | ATAAGCTCTTTGTAGATAGGAAGC | GAACATCGACATGTACAGTATTTA | 54 | 300 | GAGA | PF09237 | GAGA factor | | CA074905 | UGSuM389 | (CGC)5 | AAGAACAAGAGATACAACAACAGA | CAGTGTGTTTCTAACACTGAATC | 55 | 272 | AcetDehyd-dimer | PF09290 | Prokaryotic acetaldehyde dehydrogenase | | CA075705 | UGSuM391 | (CGC)5 | TATCTGATCGGTAGCAAATAGC | GTGGTTAAGAAGAGACTAAGTTCG | 56 | 370 | Lact-deh-memb | PF09330 | D-lactate dehydrogenase | | CA076258 | UGSuM392 | (GAG)5 | CTTCTTTCTGCTTCCAGTTATTAT | CTACAAGGCCTTTGATGAGGTT | 55 | 324 | zf-C2H2 | PF00096 | Zinc finger, C2H2 type | | CA078259 | UGSuM393 | (GCG)5 | CTCCGGGATTAAAGAATCGATGG | CCTCTTCTTAAATTTGGTACTTCT | 64 | 301 | NPHI_C | PF08469 | Nucleoside triphosphatase | | CA084510 | UGSuM400 | (GGC)5 | AAGTCTAAGAAGAACAAGAAGGG | ATGTCAAGATCCGAGTTTCTGT | 55 | 288 | Cyclin | PF08613 | Cyclin | | CA084588 | UGSuM401 | (GAA)5 | GCGCTCACATCACCTCCTACGC | GGACGATCTTGAAGAACCTGGC | 68 | 399 | CGI-121 | PF08617 | Kinase binding protein | | CA084787 | UGSuM403 | (ACG)5 | TATACAAGAATGAAAGGTGAGAGA | AAGCATACTCCCTCTATCTCTATG | 55 | 217 | ox_reductase_C | PF08635 | Putative oxidoreductase | | CA086803 | UGSuM405 | (AAG)5 | ATCGATCATTTCCTCTTCTTAGTA | AATTTGATTCATAGGATTGTGAG | 55 | 383 | NCA2 | PF08637 | ATP synthase regulation protein NCA2 | | CA086859 | UGSuM406 | (CTC)5 | GACCATCAGAGACAGCGTGGAG | GTATACTGTGAGGAGGCTGAAG | 64 | 253 | AChE_tetra | PF08674 | Acetylcholinesterase tetramerisation | | CA087032 | UGSuM407 | (CGC)5 | GATCTTGCTTCCGTTCTACGTT | TGAGGTAGAACATATTAGCTTGTG | 59 | 352 | GCD14 | PF08704 | tRNA methyltransferase complex | | CA089088 | UGSuM409 | (CTG)5 | ATCCACACTACCTGCCTTTCTC | GTGTAGTACCAGTCAGGGTCATA | 69 | 331 | zf-RING-like | PF08746 | RING-like domain | | CA089404 | UGSuM411 | (CCT)5 | GTTAGTGACTGTGTCATCAAGGT | TGCAGGAAGTTAAGAATAATAACA | 54 | 330 | Acetone_carb_G | PF08882 | Acetone carboxylase gamma subunit | | CA090918 | UGSuM412 | (GAG)5 | TGTAGGTCCTAGTAGATCTAGGGT | ATCTTGGACGAGATGAAGCAAG | 67 | 391 | GshA | PF08886 | Glutamate-cysteine ligase | | CA093042 | UGSuM413 | (GAG)5 | GTTCCTCCTCGAAACCCTATGAG | GAACGTCCGGTAGTAGTGATCC | 55 | 365 | K-cyclin_vir_C | PF09080 | K cyclin, C terminal | | CA095367 | UGSuM414 | (AGA)5 | GTACGCTAGCAATCTACTCCCTAC | GAAGCCGAACAAGAGGAGGTTC | 56 | 378 | Ubiq-Cytc-red_N | PF09165 | Ubiquinol-cytochrome c reductase | | CA098879 | UGSuM416 | (TTC)5 | GAATGAATCTACACAGGTGAATAA | GTAGCAAGATCATTGTTTCCTT | 60 | 373 | Act-Frag_cataly | PF09192 | Actin-fragmin kinase | | CA100190 | UGSuM417 | (GCA)5 | CTCAGTCCCAAACGATGCTTAG | GTTGTACTCCACATTGATCTGTT | 67 | 282 | bZIP_2 | PF07716 | Basic region leucine zipper | | CA102808 | UGSuM418 | (GGC)5 | GTACGACACGGTGAAGAACCTT | CTCATCACTATAGTCCACAAGAAC | 65 | 320 | Ost4 | PF10215 | Oligosaccaryltransferase | | CA103142 | UGSuM419 | (GCA)5 | CAAGTTCACAAGTTCAATACATAA | AAGAAGATTTGCTCAAGTTCATAC | 58 | 333 | TrmE_N | PF10396 | GTP-binding protein TrmE N-terminus | | CA103389 | UGSuM420 | (GGT)5 | CAATCGATGTTTACATCACAAC | GATAGGGAGTAAATCTGGTAGAAA | 69 | 380 | Xan_ur_permease | PF00860 | Permease family | | CA103498 | UGSuM422 | (CGC)5 | AATTCATGTTTAGATCCTCTCAAT | AATCAGTCTTGTGTAAGTCTCTTG | 55 | 399 | MADF_DNA_bdg | PF10545 | Alcohol dehydrogenase transcription factor | | CA103536 | UGSuM423 | (CGG)5 | GAGAATTGTCTTGTATATTGGTCC | TTGAGATCTCCAAGGGTACGAG | 61 | 390 | Nab6_mRNP_bdg | PF10567 | RNA-recognition motif | | CA103832 | UGSuM424 | (CCG)5 | GTTAATCTACCTTTATTACGCAGC | CTCAAGGTCGTCTTCTGCCAGT | 54 | 159 | NPCBM_assoc | PF10633 | Alpha-galactosidase | | CA103845 | UGSuM425 | (CGA)5 | ACTACTTCTAATACAACGGAGAGG | ATGAAGATCATCTACTGCAACC | 55 | 327 | Iron_transport | PF10634 | Fe2+ transport protein | | CA103895 | UGSuM426 | (GCG)5 | GATAGTAATAGGTGTAGCAGAGGC | ACCATCCTGTTGTTCCTCTCAC | 54 | 296 | zf-C2H2 | PF00096 | Zinc finger, C2H2 type | | CA104231 | UGSuM428 | (CGC)5 | GAACATGTACGACAACAACTTC | CCCTACGAGTTTATTCTTCAGTA | 62 | 158 | NAD-GH | PF10712 | NAD-specific glutamate dehydrogenase | | CA104607 | UGSuM429 | (GCA)5 | CGCATATATATAGCTAGAGCGTAA | ATATGCCCGTAGTGCGCTGAGT | 54 | 371 | NdhL | PF10716 | NADH dehydrogenase transmembrane subunit | | CA106555 | UGSuM435 | (CGG)5 | AGATCTTAACTCTCACCTGAAATC | TAGAAGATTTAACAGAGCATAACG | 63 | 183 | bZIP_2 | PF07716 | Basic region leucine zipper | | CA106603 | UGSuM436 | (GGC)5 | ATTAGGACACGAGAACTAGCATA | AGGAAATTCGTAGAAGATGTTATC | 56 | 342 | NADH-u_ox-rdase | PF10785 | NADH-ubiquinone oxidoreductase | | CA107157 | UGSuM437 | (GCG)5 | GCTGCTATATACCAAACAAGAAAT | GTACTTCAATGGGTGATAAGTGT | 55 | 393 | G6PD_bact | PF10786 | Glucose-6-phosphate 1-dehydrogenase | | CA111332 | UGSuM441 | (CCG)5 | ACATCCACCCAGATCTCGCC | GTGACGGAGATAGACACCAT | 54 | 215 | Tbf5 | PF06331 | Transcription factor TFIIH complex subunit | | CA117139 | UGSuM446 | (GAC)5 | AAACATTTATAACTCATTGGTGTG | AAGCATCTTCTTGTACCTATCAAT | 54 | 226 | NAC | PF01849 | NAC domain | | CA117696 | UGSuM449 | (GCC)5 | AGTTATTAAGGCCACCCAGCCTAA | GTTGAGGTTGATGCCAAGGATT | 54 | 333 | Transport_MerF | PF11431 | Membrane transport protein | | CA118800 | UGSuM450 | (TCC)5 | CTACCAATGATACAACAACATTCT | ATTCTTTGAGCTTAGTTGTCATAA | 60 | 134 | E3_UbLigase_EDD | PF11547 | E3 ubiquitin ligase | | CA119580 | UGSuM452 | (CGC)5 | GTATATGTTCGTAGTTTGTATGCC | ATTCACTTAGTCACACTCTCACAC | 54 | 310 | V-ATPase_H_C | PF11698 | V-ATPase subunit H | | CA120255 | UGSuM454 | (CCT)5 | TGATCTCTCTCTCTTTCTCTCTCT | ACCTTAGAATCATTCAATCCTTAC | 55 | 318 | Git3 | PF11710 | G protein-coupled glucose receptor | | CA125310 | UGSuM465 | (GCA)5 | GCTAACCAACATCAGCAGT | AGGAGATTGACGAAGAAGAAG | 53 | 342 | Trp_DMAT | PF11991 | Tryptophan dimethylallyltransferase | | CA125811 | UGSuM466 | (GCT)5 | TTTATTGAGGTTGAGGGTG | AGAGGAGAGACCATTCCATT | 53 | 150 | OSR1_C | PF12202 | Oxidative-stress-responsive kinase | | CA125935 | UGSuM468 | (TCC)5 | AGCCCTACTGATTGTGCC | TCTCCCACTTCCTTCGTT | 56 | 382 | Se-cys_synth_N | PF12390 | Selenocysteine synthase | | CA126513 | UGSuM470 | (CCG)5 | AAGAGCACAAACGCAAGTAG | TGAGCCACTGTAGGATGATT | 55 | 344 | nlz1 | PF12402 | NocA-like zinc-finger protein 1 | | CA127133 | UGSuM471 | (CCA)5 | GGAGCAGATTCATTAGTTGAG | ATCCACCAGAACAGAAACAG | 54 | 336 | ABA_GPCR | PF12430 | Abscisic acid G-protein coupled receptor | | CA127456 | UGSuM472 | (CTG)5 | CTGTTGCTGGAGTTGCTG | GATGGTGAGGTTGACGGG | 56 | 176 | zf-C2H2 | PF00096 | Zinc finger, C2H2 type | | CA128815 | UGSuM475 | (CGG)5 | TCTTTCTTATCCACCATCAAC | GGGCTCTTCTACCCAGAC | 54 | 389 | Malate_DH | PF12434 | Malate dehydrogenase enzyme | | CA130339 | UGSuM478 | (GCG)5 | GCTATTGGCTACAAAGGGT | ACTGGAGGTCATCTTCTTCTT | 54 | 181 | GDE_N | PF12439 | Glycogen debranching enzyme | | CA130542 | UGSuM479 | (GCA)5 | AAGGACGAGGTGATGAAG | CCGAGTGTCTTGGTGGAG | 53 | 395 | Nucleolin_N | PF12462 | DNA helicase IV / RNA helicase | | CA130708 | UGSuM480 | (TGC)5 | TGTTACTGCTTCTTGTGCTG | TTCAACCTCATCGCCTTAC | 55 | 242 | Mac | PF12464 | Maltose acetyltransferase | | CA130832 | UGSuM481 | (ATA)5 | TCAGCCTCATCCTCAACTAC | CAGTCAAACCATCATTCAAAC | 55 | 175 | GATA | PF00320 | GATA zinc finger | | CA131087 | UGSuM482 | (GGC)5 | GTGGCGTGCTCTGCTCTC | GTAGATTTGGAAGATGGGCT | 60 | 295 | GDH_N | PF12466 | Glutamate dehydrogenase | | CA137576 | UGSuM499 | (GAC)5 | CCAACTATCCTCATCCAAAG | AAGTCCCTCTCAATCCTGAA | 54 | 266 | GIDE | PF12483 | E3 Ubiquitin ligase | | CA137888 | UGSuM501 | (CAC)5 | AAGCACCACCACCACCAC | AAGTAGACCTTCCAGTTGCC | 60 | 304 | CaATP_NAI | PF12515 | Ca2+-ATPase | | CA138041 | UGSuM502 | (GCT)5 | TGAGAGGAGGAGGAGGAG | TGAGAAATGAATCTTGTAGGG | 55 | 400 | Lipase | PF00151 | Lipase | | CA145715 | UGSuM513 | (CTG)5 | ACTTGGTGTAAATGAATCTTG | CAGGCGATGACGGGCTTT | 51 | 259 | zf-C2H2 | PF00096 | Zinc finger, C2H2 type | | CA146953 | UGSuM515 | (CGC)5 | AGCAACCAGACGAGGAGT | GGTCCAGAGCAGTTTGTT | 55 | 135 | CCT | PF06203 | CCT motif | | CA146985 | UGSuM516 | (CGG)5 | TATGGAAGCCAAAGAGAATAG | CATCAAAGGAAGAGAGTGGA | 53 | 343 | NAD4L | PF06235 | NADH dehydrogenase subunit 4L | | CA148468 | UGSuM517 | (GGC)5 | CCCTCTCGCTCGCTACTC | CCAACACCATCTTTCTTAGTG | 58 | 292 | AceK | PF06315 | Isocitrate dehydrogenase kinase/phosphatase | | CA148954 | UGSuM518 | (CCG)5 | AACCCTCTGCTCCTCGCA | GCTTGTGCTTGTTCATCTTAC | 62 | 363 | RCC_reductase | PF06405 | Red chlorophyll catabolite reductase | | CA149330 | UGSuM519 | (GGA)5 | ATTGTTCTAAGGCACACTCT | GAAGCAGGGTCGGTGATG | 51 | 254 | NPIP | PF06409 | Nuclear pore complex interacting protein | | CA149596 | UGSuM520 | (CAG)5 | AGAGGAAAGCAAAGACAAGTAG | ATGAAGTGGAACAGAAGATGA | 54 | 149 | Sec2p | PF06428 | GDP/GTP exchange factor Sec2p | | CA153229 | UGSuM525 | (CGC)5 | TAACTGGATTCTCCTAAACC | ACACATCGCCTCCTCCTC | 51 | 187 | GPI | PF06560 | Glucose-6-phosphate isomerase | | CA154198 | UGSuM526 | (GCA)5 | ATACCCTGCACAAGGTTACTAC | AGAAGGTGCAAGGAATAGAGTA | 51 | 187 | Sugar_transport | PF06800 | Sugar transport protein | | CA154741 | UGSuM527 | (CTC)5 | CACTCCTCCGCCTTTACC | ACCCTCCATCTCCTTTCTC | 58 | 169 | Suc_Fer-like | PF06999 | Sucrase/ferredoxin-like | | CA155274 | UGSuM528 | (CGA)5 | AGATGGAGGGTGCGAGAC | TTTCTTTGGTCGTGCCTG | 58 | 375 | AATase | PF07247 | Alcohol acetyltransferase | | CA157186 | UGSuM530 | (GCC)5 | CACCTCTCTCCTCTTCTTCTC | ACAACGCCATCTCTACCA | 54 | 384 | NAD_Gly3P_dh_C | PF07479 | Glycerol-3-phosphate dehydrogenase | | CA157270 | UGSuM531 | (GCC)5 | AAACGACACGAAGCACGA | ATAAATGACGAGCCCAAG | 58 | 336 | TAT_ubiq | PF07706 | Aminotransferase ubiquitination site | | CA157996 | UGSuM532 | (GCG)5 | ATAATGACTGAACCTCTCCC | CTTCCTGTGCTTCCTGGT | 53 | 276 | MFMR | PF07777 | G-box binding protein MFMR | | CA158158 | UGSuM533 | (AGC)5 | AGAACTGAGCGAGGAGATT | GCCTGGAACTTGGTCTTG | 54 | 182 | GCFC | PF07842 | GC-rich sequence DNA-binding factor | | CA158777 | UGSuM534 | (GCC)5 | GATGACCCTTTGGATGTAGTT | ACAGAGTTTCCTTGACCCA | 55 | 379 | Leader_Thr | PF08254 | Threonine leader peptide | | CA159034 | UGSuM535 | (TCG)5 | ACAGAGAGGGAGAGAGAAAGA | GGGACATCGTGCTGAGAG | 54 | 378 | TF_Zn_Ribbon | PF08271 | TFIIB zinc-binding | | CA167733 | UGSuM549 | (CGC)5 | GTCTTCTTCTCCGCCTCC | GTTTCTCCCTTCCTTGCC | 57 | 208 | U3_snoRNA_assoc | PF08297 | U3 snoRNA associated | | CA167921 | UGSuM550 | (CGC)5 | GGAGCCTTTGGTAGGTTAG | CGTGAAGAGAGAATGAAGAAG | 54 | 304 | bZIP_1 | PF00170 | bZIP transcription factor | | CA174283 | UGSuM551 | (TGC)5 | TAGTTGTGGAAGAGATGGAGG | GATTGAAGAGCAGATGAAGTG | 56 | 191 | Stk19 | PF10494 | Serine-threonine protein kinase 19 | | CA175472 | UGSuM552 | (CCG)5 | CAGGATGAAATCGTTGGA | GATAGACCGTGGAAGTGAAGT | 55 | 387 | zf-C2H2 | PF00096 | Zinc finger, C2H2 type | | CA177332 | UGSuM553 | (CGC)5 | CACGGGCTCATCTCCAAC | CCAATAAACGCAAGGAAAC | 60 | 252 | Xpo1 | PF08389 | Exportin 1-like protein | | CA177925 | UGSuM554 | (CGG)5 | TTACACATTCAACCAAATCAG | GCTCTTCTTCTCCAGCATC | 53 | 288 | Oxidored-like | PF09791 | Oxidoreductase-like protein | | CA183376 | UGSuM557 | (CGT)5 | GTCCAACCTCACCTCCTC | GAGAAAGAGCCACGACCT | 55 | 146 | LRR_1 | PF00560 | Leucine Rich Repeat | | CA183646 | UGSuM558 | (GGC)5 | GGTTCTCGTTCTCTCTAACATC | CAAGGCTATGCTGTCTTCA | 54 | 375 | TAF8_C | PF10406 | Transcription factor TFIID complex | | CA190789 | UGSuM567 | (GCG)5 | ACAAACAGTCCAGAAAGCAA | GTGGCGAAGAGAGAGAGG | 55 | 313 | SufE | PF02657 | Fe-S metabolism associated domain | | CA195245 | UGSuM569 | (GGC)5 | CTCACGCCTCGCAGTCATC | CCTTAGCCAGCATAGCATAGT | 63 | 298 | KdpC | PF02669 | K+-transporting ATPase, c chain | | CA196032 | UGSuM573 | (CGC)5 | ACTGGTAGGAGGAGGAGATG | ACTTCTGTTCTGACTGTGGG | 55 | 205 | TYW3 | PF02676 | Methyltransferase TYW3 | | CA197130 | UGSuM575 | (CAG)5 | TAACTCCTAAGTGAACCACCA | CAACTGAAAGAGACGGAAAC | 54 | 210 | Glucokinase | PF02685 | Glucokinase | | CA201535 | UGSuM576 | (CGG)5 | AAGGTCGTCTCCATCCTG | TTGGTCTTCCAGTTGTTGTAG | 55 | 296 | GASA | PF02704 | Gibberellin regulated protein | | CA202200 | UGSuM578 | (CGG)5 | TAATCCATCAGAAGCACAGAA | CAAATACGGGTGGCATCA | 55 | 297 | TPP_enzyme_N | PF02776 | Thiamine pyrophosphate binding domain | | CA202772 | UGSuM580 | (CGG)5 | TAGAAACCATTCGGAGTATCA | GTGGAGAGGATGAAGAGGA | 54 | 358 | GSH-S_ATP | PF02955 | Prokaryotic glutathione synthetase | | CA206056 | UGSuM583 | (CGC)5 | TGCCCTGTGTTCCTTCCT | GGTGCTTGTTGACCATTCT | 59 | 377 | tRNA_Me_trans | PF03054 | tRNA methyl transferase | | CA206398 | UGSuM584 | (TCC)5 | GTCTACTCTGCTCTGGGTTC | CTGCTGCTTGTGGTTGTG | 53 | 141 | Nucleoplasmin | PF03066 | Nucleoplasmin | | CA206719 | UGSuM585 | (GGA)5 | GCTACTCGCATCTGTCTGG | GCAATGGGCAACTAATAAAC | 57 | 211 | Replicase | PF03090 | Replicase family | | CA207346 | UGSuM586 | (TCG)5 | CTCACCCACATACCTCACTC | GGGAAGGAACACAGGAAA | 55 | 256 | WRKY | PF03106 | WRKY DNA-binding domain | | CA209243 | UGSuM589 | (GCG)5 | CGTTGCTCTTGTTCCTCTT | GACCTTTCCCTTCCGCCA | 55 | 191 | bZIP_Maf | PF03131 | bZIP Maf transcription factor | | CA225244 | UGSuM600 | (GGA)5 | TTCAATCTAACACCTTTACCAA | TTCCATTCTAACCAGCAAA | 54 | 331 | TPT | PF03151 | Triose-phosphate Transporter family | | CA225391 | UGSuM601 | (CAG)5 | AAGACCCGTGGAGCAGAG | GACGCCGACAGGACAAAC | 58 | 321 | UDG | PF03167 | Uracil DNA glycosylase superfamily | | CA226040 | UGSuM602 | (CGC)5 | ATCAAGCACGCCCGCCTC | AAAGAAAGTCCAGAACCC | 67 | 312 | GSH_synthase | PF03199 | Eukaryotic glutathione synthase | | CA226051 | UGSuM603 | (AGC)5 | ATCAAGCACGCCCGCCTC | AAAGAAAGTCCAGAACCC | 67 | 312 | ACCA | PF03255 | Acetyl co-enzyme A carboxylase | | CA226086 | UGSuM604 | (AGC)5 | CTTTGGCTCTGTTATCTTCTG | CGTGTGGGTGCCTTCTCC | 54 | 298 | TF_AP-2 | PF03299 | Transcription factor AP-2 | | CA226502 | UGSuM605 | (CAG)5 | TAACGGAGAACGACCTGA | GAGGAGTAAACCGCAGAAG | 55 | 390 | Trp_dioxygenase | PF03301 | Tryptophan 2,3-dioxygenase | | CA226506 | UGSuM606 | (CGC)5 | GGCAAACGCAACACGAAG | AACAGCCAAGATGATTTCC | 61 | 351 | CBM_19 | PF03427 | Carbohydrate binding domain | | CA226976 | UGSuM607 | (GGC)5 | CTGGTGTTGCTGGTGTAGT | GAGAGTTCCTGTCCAAGTGA | 54 | 381 | GRAS | PF03514 | GRAS family transcription factor | | CA227013 | UGSuM608 | (GGC)5 | GGGCTATTACCACACAGACTAC | GCTAACACCACCAGCACTAA | 55 | 331 | zf-C2H2 | PF00096 | Zinc finger, C2H2 type | | CA227263 | UGSuM609 | (AGC)5 | AGAACACTAACCACCACCC | CTCGCCGTCCTGCTTTGAG | 54 | 235 | Na_H_antiporter | PF03553 | Na+/H+ antiporter family | | CA227455 | UGSuM610 | (CCG)5 | CGACCTTATCCTCTTCCTCT | GGCTGTCTTCTCCTTCCC | 55 | 213 | CdhC | PF03598 | Acetyl-CoA synthase complex beta subunit | | CA227823 | UGSuM611 | (GGC)5 | AGGTCATCTCTCTCTTCTCGT | CTCCTTCTCCTCCTTCTTGT | 54 | 217 | CitMHS | PF03600 | Citrate transporter | | CA228227 | UGSuM612 | (AGG)5 | ACAAGGATGGAGAACAACAG | ATGAATGAACAAGCGAAGAAC | 55 | 152 | LEA_1 | PF03760 | Late embryogenesis abundant (LEA) group | | CA232490 | UGSuM614 | (ACG)5 | TACACCGTCTTCTCCGCC | TGATACTCCTTTCCCTCTCTG | 59 | 400 | KNOX1 | PF03790 | KNOX1 domain | | CA232822 | UGSuM615 | (GCC)5 | TTGTTAGTTTATTGGAGGGAA | GGCACATCTCTTGCTGTC | 54 | 279 | SUN | PF03856 | Beta-glucosidase (SUN family) | | CA235607 | UGSuM617 | (GCC)5 | TGAGAACAAATGCCCAAC | GAAGGGTGGAATCGTCTT | 55 | 342 | NapB | PF03892 | Nitrate reductase cytochrome | | CA236058 | UGSuM618 | (CAC)5 | CAAGAACGGCTACGGCGA | AGGAAACCAGATACCAGAGAG | 63 | 327 | Nop10p | PF04135 | Nucleolar RNA-binding protein | | CA248171 | UGSuM632 | (GCC)5 | CCTCATAACCGAGAAGAACTG | TACCTCCACTGCCACGAC | 56 | 247 | Sugar-bind | PF04198 | Putative sugar-binding domain | | CA248880 | UGSuM633 | (CCG)5 | GGTGGGTGGATGATAGAAA | CTTCGTCGGTCGGGAATCT | 55 | 263 | FMN_bind | PF04205 | FMN-binding domain | | CA251888 | UGSuM634 | (AAG)5 | AAAGTTGAGCGTTTCTTGG | TCATAGTTCTGCTGCTGTTG | 55 | 335 | OAD_gamma | PF04277 | Oxaloacetate decarboxylase | | CA251891 | UGSuM635 | (CGG)5 | GACTACGCCTTCTCCTCC | ATGAGTTCCTTCTCGCACT | 54 | 359 | G3P_antiterm | PF04309 | Glycerol-3-phosphate | | CA252869 | UGSuM638 | (CAG)5 | ACAGCACAGGCTCTCTCTT | TCCTTTCAGGCATCCATC | 55 | 178 | tRNA_synt_1c_R1 | PF04558 | Glutaminyl-tRNA synthetase | | CA252938 | UGSuM639 | (GTG)5 | AGGCAGAGCAAGGTCAGT | AAAGAATGAATGGAAGGATTT | 55 | 309 | zf-C2H2 | PF00096 | Zinc finger, C2H2 type | | CA253365 | UGSuM640 | (GCC)5 | CGATTGATTATTTCTTGCTGA | CATACGCCTGCTCCAACC | 55 | 291 | Mt_ATP-synt_B | PF05405 | Mitochondrial ATP synthase | | CA256489 | UGSuM642 | (CGC)5 | GTTTACATCCACCTCCGCC | GCTCTCCCTTCATCTCCTC | 60 | 302 | Glycogen_syn | PF05693 | Glycogen synthase | | CA256691 | UGSuM643 | (GCC)5 | TCCTCCTCCTCCTCAGTC | GAATGGCTGGAACACAAC | 54 | 211 | MRP-L27 | PF09809 | Mitochondrial ribosomal protein L27 | | CA257758 | UGSuM644 | (AGC)5 | TAATAGAGGTGGATTTGGAC | CTTTCTTTCATTGTTGTCGTT | 51 | 364 | RHD3 | PF05879 | Root hair defective 3 GTP-binding protein | | CA259134 | UGSuM646 | (GTG)5 | ATTGGAGATGGTTTATTTCA | GACACACGCCAGGTAGATT | 52 | 178 | MRP-L28 | PF09812 | Mitochondrial ribosomal protein L28 | | CA261861 | UGSuM650 | (CGG)5 | CTTACACCATCAGCACCTC | TTCTCTCCCACACACACAC | 53 | 296 | UvsW | PF11637 | ATP-dependant DNA helicase UvsW | | CA262420 | UGSuM651 | (CCG)5 | TACGCTGTGATACGCTTG | CCTTCTACTTTCGTCGTTCTC | 54 | 362 | ACC_central | PF08326 | Acetyl-CoA carboxylase | | CA263669 | UGSuM652 | (GAT)5 | TCTTCAACTTCCTCTGCCT | GTTCCTGACTGTTCCCTTG | 55 | 319 | COX15-CtaA | PF02628 | Cytochrome oxidase assembly protein | | CA264686 | UGSuM653 | (GGC)5 | GCTACTACTCTCCGTGTTCCT | GACAATGATGTTCTCGTCCT | 55 | 244 | ABC_tran | PF00005 | ABC transporter | | CA264842 | UGSuM654 | (CCG)5 | ATCTCTCTCGTTCGTCGT | GTTGTTCTGCTTCAGGATGT | 52 | 314 | LdpA_C | PF12617 | Iron-Sulfur binding protein | | CA269918 | UGSuM660 | (GAG)5 | CTCACCACTGCTCTCCATCT | CCACCAGCACCTTCTCGATG | 55 | 303 | GDE_C | PF06202 | Amylo-alpha-1,6-glucosidase | | CA270378 | UGSuM661 | (CGC)5 | GTCGTCTTCCTCTACTGGTTC | CTCTTTCCTTCTCTACGCAA | 55 | 375 | TF_AP-2 | PF03299 | Transcription factor AP-2 | | CA270923 | UGSuM662 | (CTC)5 | TGGATTTGATTTCGTGACTT | CTACTCTCATTGCTGCCAC | 55 | 372 | UDP-g_GGTase | PF06427 | UDP-glucose:Glycoprotein Glucosyltransferase | | CA271355 | UGSuM663 | (CGC)5 | GTGTCTTTGTGGCTGAGG | ATTACATCATCTGGTTGTGCT | 54 | 353 | MRP-L28 | PF09812 | Mitochondrial ribosomal protein L28 | | CA272222 | UGSuM664 | (GCT)5 | CCTCTACAAACGGCTCTCT | CAGTAACAACAACCACAGGAC | 54 | 286 | MCD | PF05292 | Malonyl-CoA decarboxylase | | CA272711 | UGSuM667 | (CCA)5 | CTCACATTTCTTCCCTTCTCT | ATAGCCTCGGTTTCATTCC | 55 | 231 | GATA-N | PF05349 | GATA-type transcription activator | | CA275058 | UGSuM669 | (GCT)5 | TACCAATCAGCAATCAAGAC | CAACATCCAGAACAAGCAC | 53 | 329 | CaKB | PF03185 | Calcium-activated potassium channel | | CA275285 | UGSuM671 | (CTC)5 | TTTACATCACAACCTTCCGT | AACCCTTCTCCTGTCACTCT | 55 | 384 | zf-C2H2 | PF00096 | Zinc finger, C2H2 type | | CA276123 | UGSuM673 | (TCC)5 | CTATCTCTATCTGCTTCCTGCT | TTGTGAACTGCCTTCTTCTT | 54 | 177 | Gb3_synth | PF04572 | Alpha 1,4-glycosyltransferase | | CA276157 | UGSuM674 | (CCG)5 | ATTGTTGATTTGCTTCAGGT | AAAGGTTACGGGAGAGGAG | 54 | 289 | GRDA | PF04723 | Glycine reductase complex selenoprotein A | | CA279093 | UGSuM675 | (GGC)5 | AGAACAACCCTTCCGTGT | TCATCAACATCAACTTTCAG | 55 | 318 | NDUFA12 | PF05071 | NADH ubiquinone oxidoreductase | | CA279291 | UGSuM677 | (GCG)5 | CCTCTCCTCTCTCTCTCTCTC | GCTGTTCCTTGTGCTTGT | 54 | 341 | S6PP | PF05116 | Sucrose-6F-phosphate phosphohydrolase | | CA279499 | UGSuM678 | (GGC)5 | GTGGACGAGAAGTGGAAGT | ATAGGAGGGCAGGACAAG | 55 | 370 | Macoilin | PF09726 | Transmembrane protein | | CA279564 | UGSuM679 | (TCG)5 | TAGAGCCATAGAGGCGATAGA | CTACGAGGAAGAACGGCGGG | 56 | 395 | G-alpha | PF00503 | G-protein alpha subunit | | CA279822 | UGSuM680 | (CCG)5 | TTCTTGCGGTTCTTCTTG | CATCTCCTTCCCACCTTC | 54 | 170 | V-ATPase_C | PF03223 | V-ATPase subunit C | | CA279920 | UGSuM681 | (CCG)5 | GTGTGCCTGTGTCTGTGTT | GTCCAGTTTCCATTCGGT | 55 | 307 | U-box | PF04564 | U-box domain | | CA280184 | UGSuM682 | (GCT)5 | AATCTCTTTCTCCCTCTTATCC | TTCTACCCTAACCTTCCAGAC | 54 | 278 | YABBY | PF04690 | YABBY protein | | CA280497 | UGSuM683 | (CGG)5 | ATGCCTTGAAATCTGCTC | CTCCGTTCCTCTCCATCC | 53 | 259 | QCR10 | PF09796 | Ubiquinol-cytochrome-c reductase complex | | CA280557 | UGSuM684 | (GCC)5 | TATTTACAACGGGCAACATC | TGAATGAACAGCAGCAAGT | 56 | 137 | O-FucT | PF10250 | GDP-fucose protein O-fucosyltransferase | | CA282616 | UGSuM687 | (CTT)5 | AGATGAGGAAGAGGATGATG | GGTAGGTGTGGGAGCACTT | 54 | 274 | GTP_EFTU | PF00009 | Elongation factor Tu GTP binding domain | | AY644463 | UGSuM700 | (AG)7 | AATACAGCGCAATATTTCCTCC | ATAACCTCAACGCCAAGAGCAAGT | 59 | 341 | KH_1 | PF00013 | KH domain | | CA074508 | UGSuM703 | (CT)7 | CTTTCCCTCTTCCTCTCTCGTAG | CTTCAATGTTTGTATTGGATAAAG | 60 | 139 | cNMP_binding | PF00027 | Cyclic nucleotide-binding domain | | CA087531 | UGSuM705 | (AC)7 | ACACCTGGAACCAGTTCCTCTAC | AAGAATCGAAGGTTAACAACAG | 60 | 292 | Gp_dh_N | PF00044 | Glyceraldehyde 3-phosphate dehydrogenase | | CA098584 | UGSuM706 | (CT)7 | AACCCACCAATATACTACCTACAG | CTACAAGATGCCAAATATGGTT | 60 | 316 | Ldh_1_N | PF00056 | lactate/malate dehydrogenase | | CA158818 | UGSuM717 | (GT)7 | CATGAGCCTTTGGATGTAGTT | TCAGAGTTTCCTTGACCCA | 55 | 379 | Sugar_tr | PF00083 | Sugar (and other) transporter | | CA167796 | UGSuM720 | (CA)7 | GCCAGACAGCGACCAGAG | CCCAGAGGGAGGGTAAGG | 60 | 294 | RuBisCO_small | PF00101 | Ribulose bisphosphate carboxylase | | CA183663 | UGSuM721 | (CA)7 | ATAACACACACACACACAAA | TTCCGAGGGCAAATAATC | 50 | 174 | COX1 | PF00115 | Cytochrome C and Quinol oxidase polypeptide I | | CA184137 | UGSuM722 | (GA)7 | GCAATCAGCCTTACAGCA | GAACATCGCACTTTGGAG | 55 | 310 | Gln-synt_C | PF00120 | Glutamine synthetase | | CA184950 | UGSuM723 | (GA)7 | GTCTGAACAAGAGCGACAA | TAGGATTACACACCACACCAC | 54 | 398 | Metallothio | PF00131 | Metallothionein | | CA203271 | UGSuM724 | (GA)7 | GTCATCGTCATCCTCCTCT | GATTGTGAGCAAACCCATT | 54 | 331 | tRNA-synt_1 | PF00133 | tRNA synthetases class I | | CA206091 | UGSuM725 | (AG)7 | GTCATCTCCATCGCCTCCC | CGAAGTTTGGGTCGTTGA | 62 | 334 | NADHdh | PF00146 | NADH dehydrogenase | | CA212706 | UGSuM726 | (GA)7 | AAACAATCCAAACCAACAAC | GCTACAAGAACTCGCCCT | 54 | 297 | Aldedh | PF00171 | Aldehyde dehydrogenase family | | CA213608 | UGSuM727 | (GT)7 | CCGCTTTCAACCTCTACAC | GGCTTGGTGATTCTTCTCT | 55 | 285 | Oxidored_molyb | PF00174 | Oxidoreductase molybdopterin binding domain | | CA254514 | UGSuM730 | (CG)7 | CTGCTGTTCCTCTTATTGCT | CGGAGACTGAAACCTCGT | 54 | 123 | Glyco_hydro_19 | PF00182 | Chitinase | | CA264205 | UGSuM732 | (TG)7 | GAGAGCAGACGACGGAGG | ATAGTCCACAAGAACGAACA | 59 | 235 | Catalase | PF00199 | Catalase | | CA270150 | UGSuM733 | (TC)7 | TCTGTTTCAGTCGTTTCCTAC | CGATTTCTTTCTTTCTTCTCC | 54 | 128 | Ferritin | PF00210 | Ferritin-like domain | | CA272719 | UGSuM735 | (TA)7 | TAGTGATGCTCTCGCAACC | ATCTCAGTCAGTCTCCCACA | 57 | 330 | ATP-synt | PF00231 | ATP synthase | | CA279539 | UGSuM736 | (GA)7 | AGCACGAAGCAATACCAG | AACGGACACCACCACTAC | 54 | 341 | Myb_DNA-binding | PF00249 | Myb-like DNA-binding domain | | AY521566 | UGSuM739 | (AG)6 | TAATATACATCACGACACAACAAA | TTGTGTTATCTAGACGATACCTTT | 55 | 348 | Glycolytic | PF00274 | Fructose-bisphosphate aldolase | | BU103681 | UGSuM743 | (GA)6 | AGTAGCCAGGATGGTACTTGTC | GACCCTAATTGTAGTTCTCTCAA | 55 | 399 | Citrate_synt | PF00285 | Citrate synthase | | CA065380 | UGSuM744 | (CG)6 | AAGGCGAGCAGAGCACATCAAT | GAGGTGTAGTTGAAGTAGTCGTAG | 54 | 278 | Na_K-ATPase | PF00287 | Sodium/potassium ATPase beta chain | | CA072593 | UGSuM746 | (GA)6 | TGAGAATTTACTGATTTACAGTGC | GAGTTTGACCTCAATGAAACTG | 55 | 141 | CAT | PF00302 | Chloramphenicol acetyltransferase | | CA074584 | UGSuM747 | (CT)6 | ACTTCATCTCTCGACGCCCATCT | CTCGTACTCCTTGACCTTGAGT | 65 | 364 | FBPase | PF00316 | Fructose-1-6-bisphosphatase | | CA085169 | UGSuM748 | (CT)6 | ATCTTTCTCGTCCGCCTCCGCT | GTCATCAATGACAGAGATGTAGAC | 69 | 163 | AA_permease | PF00324 | Amino acid permease | | CA086761 | UGSuM749 | (TA)6 | CAAATCCTTGTGCTTCAGATCG | CACTCTCAAATCTTGGAGAATAAT | 62 | 396 | Acid_phosphat_A | PF00328 | Histidine acid phosphatase | | CA091702 | UGSuM752 | (GC)6 | GCGTCTCTGCTCTGCACTCTGC | ATTAACATATTCATAGCCCAATTT | 62 | 120 | Aconitase | PF00330 | Aconitase family | | CA093469 | UGSuM753 | (CA)6 | TACACTCACACTCACACACGTCTC | ATGTTGACGCTCTTGACGCTGT | 65 | 390 | NDK | PF00334 | Nucleoside diphosphate kinase | | CA103036 | UGSuM755 | (TA)6 | ATTCATACTCGAAGTTTGTATGC | ACTGCACCGACATGATGTTCTT | 61 | 185 | Oxidored_q1 | PF00361 | NADH-Ubiquinone/plastoquinone | | CA103405 | UGSuM757 | (GA)6 | AGTGGAAGTTCTCCAAGAAGAG | AGTCCTTACAGCATACGAGCAT | 57 | 266 | Molybdopterin | PF00384 | Molybdopterin oxidoreductase | | CA111537 | UGSuM763 | (CT)6 | GGAAGCAGAGAAAGAGAGATATAC | GTTGGTGATGAAGTTGAGGCAC | 55 | 357 | UCH | PF00443 | Ubiquitin carboxyl-terminal hydrolase | | CA125488 | UGSuM764 | (CG)6 | CTAAGACAAGCAAGGCATCT | GTAGTTCCAGTAGCATCAGCA | 54 | 337 | ICL | PF00463 | Isocitrate lyase family | | CA126513 | UGSuM765 | (CT)6 | TGCTTGCTCTCCTATTACATT | GGGACTCGTTTGTTTCTTTAC | 54 | 382 | Fe-ADH | PF00465 | Iron-containing alcohol dehydrogenase | | CA150430 | UGSuM774 | (CT)6 | GAACCTGGCGATTTATGAG | TATGATTGAAAGACGGAACAC | 55 | 368 | Chloroa_b-bind | PF00504 | Chlorophyll A-B binding protein | | CA150625 | UGSuM775 | (GT)6 | GACGACTGACAAGGCGAG | GTAGAAGACCCAGAACCACA | 57 | 158 | BPD_transp_1 | PF00528 | Binding-protein-dependent transport system | | CA157160 | UGSuM776 | (TA)6 | AAGACACTTAATCCCTTGAAGA | TTAAGAAGAGACTAAGTTCGCC | 55 | 333 | RasGAP | PF00616 | GTPase-activator protein for Ras-like GTPase | | CA158542 | UGSuM777 | (CG)6 | TGCGTGCTCCACAACATC | GTGTCGGTTTCGTTCACC | 60 | 370 | Oxidored_q1_N | PF00662 | NADH-Ubiquinone oxidoreductase | | CA165870 | UGSuM779 | (CG)6 | AATGTCAGGCAGAGGGAG | GTAAAGTGATGAAAGTGCGG | 56 | 231 | AA_kinase | PF00696 | Amino acid kinase family | | CA166936 | UGSuM780 | (CG)6 | TTGTTTCAGAGCCGTCTACTAT | GTAATAGGACTGGATTGGAATG | 56 | 231 | Acyl_transf_1 | PF00698 | Acyl transferase domain | | CA184623 | UGSuM782 | (CT)6 | AAGCCTCTCCTCTCCTCC | CCTTGGTTCGGTCTATGTT | 55 | 245 | Oxidored_FMN | PF00724 | NADH:flavin oxidoreductase | | CA204853 | UGSuM783 | (AG)6 | GACAAGCAAGGGAAGGAA | GGAACAGAGCCAAGAGATG | 56 | 127 | Acetyltransf_2 | PF00797 | N-acetyltransferase | | CA209258 | UGSuM787 | (CT)6 | TCCTTCTCACCTACCTAACTTG | GGTTGCTTCATCAGTAATCAG | 55 | 375 | Ribul_P_3_epim | PF00834 | Ribulose-phosphate 3 epimerase family | | CA210322 | UGSuM789 | (CA)6 | ATCAGAGACAGCGTGGAG | CGTAGAACGGAAGCAAGA | 54 | 286 | Acetate_kinase | PF00871 | Acetokinase family | | CA211805 | UGSuM790 | (CG)6 | CAGGAGCAGAGGAGACAC | AAATGGCACGATGAGGTAG | 53 | 281 | A_deaminase | PF00962 | Adenosine/AMP deaminase | | CA252023 | UGSuM795 | (TA)6 | ATTCATAGGTTGAGGTTCTGG | ATAAACAAGACAGCATTTGGA | 55 | 160 | UDPG_MGDP_dh | PF00984 | UDP-glucose dehydrogenase family | | CA252563 | UGSuM796 | (CA)6 | ATTGAGGGAAAGGGAATG | GTCCTGGTGGTAACGAAG | 54 | 389 | Oxidored_q1_C | PF01010 | NADH-Ubiquinone oxidoreductase | | CA253276 | UGSuM798 | (TA)6 | CTCTCTCTCTCTCTCCCTCTG | TCACTTCTTCCTTCTTCTCCT | 55 | 348 | Carboxyl_trans | PF01039 | Carboxyl transferase domain | | CA261155 | UGSuM800 | (GA)6 | GATAGGCACTGAACAAGTCAA | AGACGGACAAGAGAGGTCA | 55 | 341 | FMN_dh | PF01070 | FMN-dependent dehydrogenase | | CA269306 | UGSuM801 | (CG)6 | CAGAGCAGCGTCGTCACC | GTAACATTCTTGAGGGTCCA | 61 | 279 | NIR_SIR | PF01077 | Nitrite and sulphite reductase 4Fe-4S domain | | CA269905 | UGSuM802 | (TG)6 | CACATCTATTACAAACCGCAC | TGCTCAAGTTCATACAACAA | 55 | 306 | F_bP_aldolase | PF01116 | Fructose-bisphosphate aldolase | | CA269916 | UGSuM803 | (CT)6 | GTCGTCTTCCTCTACTGGTTC | CGATTTCTTTCTTTCTTCTCC | 55 | 303 | Sdh_cyt | PF01127 | Succinate dehydrogenase/Fumarate reductase | | CA278861 | UGSuM804 | (GA)6 | CTCTCGCTTGCTTGTGTC | TAGTGGAAGGGTTCTGTTTG | 55 | 379 | Malate_synthase | PF01274 | Malate synthase | | CA280822 | UGSuM805 | (AT)6 | GAGCACCTCACAGCATTT | CTCTCACGCCACCGCAAC | 54 | 299 | A_amylase_inhib | PF01356 | Alpha amylase inhibitor | | CA283708 | UGSuM806 | (TG)6 | TAAGTGAAAGGAGCCAGAAA | GTCGTCGTGTCCAATAATAAC | 55 | 270 | Transposase_1 | PF01359 | Transposase | | CA094806 | UGSuM811 | (TCACAG)10tgtac (CA)10 | AGAGAGAGAGAGACAAAGGATG | TAATGGAATTGAGAGAATGTGA | 54 | 313 | R3H | PF01424 | R3H domain | | CA258125 | UGSuM813 | (CT)21gtat..(AC)28 | AAACAGAATTGCAGCCTTTAT | GACATGAAACTTTGTTGATCTG | 55 | 148 | B_lectin | PF01453 | D-mannose binding lectin | | CA258354 | UGSuM814 | (CT)6atatat..(AG)25 | AGAAGGAACCAGGATAGAGAAT | AAGTAAGAAAGACGAACTCTGC | 55 | 196 | Zein | PF01559 | Zein seed storage protein | | CA291516 | UGSuM816 | (AT)7gaact...(AAAT)7 | AACTAACAATGACAGTTCCTCC | ATCGTAATACTCATCGTACCGT | 54 | 371 | IF4E | PF01652 | Eukaryotic initiation factor 4E | | CA126151 | UGSuM819 | (CCCTG)5cttctc...(GCC)6 | AGATGGATGAGGGTTTCTTT | CCTACGAGTTTATTCTTCAGT | 55 | 273 | UDPGP | PF01704 | UTP--glucose-1-phosphate uridylyltransferase | | CA171064 | UGSuM820 | (AG)17tcgt...(TC)10 | AGATAACATACAAGCCCACATC | CTGTGGTCAGAAACTCATACAC | 55 | 310 | Nop | PF01798 | Putative snoRNA binding domain | | CA293543 | UGSuM823 | (CA)7(CACG)10cgc..(CA)36 | CAGAAACGGAGAACGGTG | TACACAGCACAGGATGGTAA | 57 | 161 | MNHE | PF01899 | Na+/H+ ion antiporter subunit | | CA207282 | UGSuM831 | (CGG)5gcg...ga(GCG)5 | CTAAATACAGCACACGCTAAA | ATCTTCCTGGCGGTTATG | 53 | 186 | NTP_transf_2 | PF01909 | Nucleotidyltransferase domain | | CA198392 | UGSuM833 | (AT)6gatat...(TA)37 | GGGTTTACAACAATCAGTTCTT | TTGATATCATCTAAGCTCCACA | 55 | 361 | TrmB | PF01978 | Sugar-specific transcriptional regulator | | CA175096 | UGSuM836 | (CTC)6(CT)11cccgt...(CGG)7 | GTTTCAGATCTCTCCTGGTAAA | TTGGAAGTTGTTCTGTTGTAGA | 55 | 194 | vATP-synt_AC39 | PF01992 | ATP synthase | | CA171622 | UGSuM841 | (CT)6catct..(AT)10 | GCCGCGTCGACTTTGATTCT | GTATCTCACGTGCTATCTTCG | 64 | 390 | Trm56 | PF01994 | tRNA ribose 2'-O-methyltransferase, aTrm56 | | CA258406 | UGSuM842 | (AGG)5ggac...g(AGC)6 | TCCAACTCACCTCAACAACT | CATTCGGGAGCCACTTCA | 55 | 290 | tRNA-synt_2e | PF02091 | Glycyl-tRNA synthetase alpha subunit | | CA153774 | UGSuM843 | (TTTC)5(TTC)5accat...(AG)13 | AGATAAGGACACGGTGAATAAG | AGCAGCATAGATGAAAGAAAGT | 55 | 321 | tRNA_synt_2f | PF02092 | Glycyl-tRNA synthetase beta subunit | | CA139166 | UGSuM844 | (CT)16(AT)18c(TA)10gga(GT)10 | ACCACCACAGTTTCAGCA | CTTTCACGACGAGGGAGA | 55 | 289 | NTF2 | PF02136 | Nuclear transport factor 2 domain | | CA179066 | UGSuM845 | (TGC)5aagt...tt(TGC)6 | ACATACACTCCTAAACTACGG | GGCACACATAGACAAGGG | 51 | 311 | CDI | PF02234 | Cyclin-dependent kinase inhibitor | | CA179066 | UGSuM846 | (TGC)5aagt...(TGC)6 | CTGTGTTCACCAAGTTAATGAG | AAGAAGCAGAAGAGAGTATGGA | 55 | 373 | Rib_hydrolayse | PF02267 | ADP-ribosyl cyclase | | CA164565 | UGSuM849 | (TC)7tgtgcg..(AG)18 | ATAGATAACAACGAGGAAGTCG | AACTTTGTACAGATCGCATGTA | 55 | 177 | UCR_14kD | PF02271 | Ubiquinol-cytochrome C reductase complex | | CA113963 | UGSuM851 | (ATCT)11(TAT)17 | ATCGTTACATTCTCCTTCTGAT | CAATAGCACTAGTTCCTCCATC | 54 | 360 | Trehalose_PPase | PF02358 | Trehalose-phosphatase | | CA084581 | UGSuM853 | (CTCC)5ctc...tg(TC)10 | CTCTCTCTCCCTCTCCGT | CACAGACGAACAATCCATC | 54 | 358 | C_tripleX | PF02363 | Cysteine rich repeat | | CA230213 | UGSuM859 | (GTAT)7gtacag..(AT)29 | CATCTTCATCTTTCTTCTCCTT | GATTACGGGAGAGCTATACTTG | 54 | 353 | Glucan_synthase | PF02364 | 1,3-beta-glucan synthase component | | CA231510 | UGSuM863 | (GAA)5gcgctc..(TCC)7 | ACTCCTGTTTGTGCAATTAAA | CTAGGAATTGAAGCTGAGATTT | 55 | 116 | NadA | PF02445 | Quinolinate synthetase A protein | | CA279512 | UGSuM864 | (CGT)5cgggc..(GGC)5 | CATCCATTTCGAATTATTACCT | ACTTTCTGATAAGCCAGTCAAC | 55 | 378 | Nodulin | PF02451 | Nodulin | | CA150670 | UGSuM865 | (TGC)7tactg...(AG)17 | TTCCTCTTCTTCCTTCTAATCA | GACCAGATTGCTGTCTTCTTAG | 55 | 346 | HATPase_c | PF02518 | Histidine kinase | | CA077024 | UGSuM868 | (CG)7agag...ggct(GCG)6 | ATACTCCATTCAGGTGGCAG | AGGCTTGTTTGCTTTCAG | 57 | 367 | GBP_repeat | PF02526 | Glycophorin-binding protein | | CA149973 | UGSuM869 | (AC)12tnaa...ttt(TA)7 | GAACGACGAGACTGGAAG | GACCTGGAAATAACCCAAAC | 54 | 360 | Ldh_2 | PF02615 | Malate/L-lactate dehydrogenase | | CA206075 | UGSuM870 | (GA)10gca...gg(GGC)7 | AACTTCTGTGCTTTCTCCTCT | GCTCAACTGGATGCTGAA | 54 | 145 | Rib_5-P_isom_A | PF06026 | Ribose 5-phosphate isomerase A | | CA218472 | UGSuM874 | (ACG)5ctggct..(CTT)7 | CTTCCTGAATAATCCTGACC | GTTGCTGTTATTACTGTGATGC | 53 | 317 | Macoilin | PF09726 | Transmembrane protein | | CA176184 | UGSuM876 | (GCG)5acg...(AT)14 | AAGGAAGACGACGAGGAG | GTCAGCACCACAGGAAAG | 55 | 367 | Acetyltransf_1 | PF00583 | Acetyltransferase (GNAT) family | | CA289517 | UGSuM886 | (CGC)5cg...ag(GAC)5 | TATTATGTCTGGAGCGGGTT | AAGAAGAGGGTTGACTTTCAC | 57 | 287 | MAPKK1_Int | PF08923 | Mitogen-activated protein kinase kinase 1 | | CA128269 | UGSuM889 | (CGG)5ctgg..(TTC)5 | GAAACAAAGTAGACTACCTGCC | AGATCACAAAGCTACATCATCA | 54 | 209 | Sec31 | PF11549 | Protein transport protein SEC31 | | CA158306 | UGSuM890 | (CT)6att...ac(GAG)6 | TTTGAAGGTGAGAAGGGTG | TGATAACTTGCTGCTTGATTT | 56 | 323 | TMF_DNA_bd | PF12329 | TATA element modulatory factor 1 | | CA266356 | UGSuM907 | (CTC)6ggc...g(GGC)7 | CATCCGACATCCTGGTGG | CTAATGCCCTGTTGTGTTG | 60 | 399 | F-box | PF00646 | F-box domain | | CA110600 | UGSuM909 | (TG)6cgcgt..(CA)6 | CAACGTTGTCTTCTCGGGTT | TAATTTGTTAAAGGCAGGATCT | 60 | 309 | Tudor-knot | PF11717 | RNA binding activity-knot of a chromodomain | | CA150718 | UGSuM919 | (AT)6(TA)7ttt(G)19 | GAGTTCCAAGAAAGCAAACA | GTAGCATCACACCTCAATCC | 55 | 364 | Lipase | PF00151 | Lipase | | CA261119 | UGSuM920 | (GCA)7gctg...cg(GCA)6 | ATACAAGCAAAGCAACTACCA | GGAGCGTCAGCAACCACT | 55 | 243 | Glyco_hydro_1 | PF00232 | Glycosyl hydrolase family 1 | | CA084731 | UGSuM921 | (CGC)5cgac...(GAG)8 | CGATCTCGAGAATCCCAAGT | AGAGAAAGATCAAACCGTACAC | 59 | 234 | Sucrose_synth | PF00862 | Sucrose synthase | | CA295967 | UGSuM925 | (CT)9gattt(TG)10 | CTTCTCCCGCTCCTAACC | AACCACTCCCTTCCTCCT | 57 | 206 | Na_Ca_ex | PF01699 | Sodium/calcium exchanger protein | | CA116533 | UGSuM927 | (GAGGA)6(GA)7 | CCAACAAACTGATTGTGATAGA | TCCAAAGTGAAAGTGTCAATAG | 55 | 368 | Ribosomal_60s | PF00428 | 60s Acidic ribosomal protein | | CA130851 | UGSuM935 | (CCG)5ctgac...(CAA)5 | CGAATCTGGAAAGAGAGTAAAC | TCTTGCAGCCACTAAATAGTAA | 54 | 231 | K_trans | PF02705 | K+ potassium transporter | | AY596572 | UGSuM939 | (GAA)8(AG)7 | CCGAAGAAGATCGTAGAATTAG | GTTGTTAGCAGCATCTTCTTTC | 55 | 350 | Synthase_beta | PF11421 | ATP synthase F1 beta subunit | | CA158557 | UGSuM944 | (TG)7(TA)10 | TCACTCGTCAGTTCCATCTC | CATAGTTAGTAGCGTCCCGT | 56 | 335 | Cytochrom_B_C | PF00032 | Cytochrome b | | CA091719 | UGSuM947 | (CT)6(CGC)7 | TTGAAGCCTGACCCGAAG | TAAGACCAAATACCACCCTG | 59 | 392 | NAD_binding_6 | PF08030 | Ferric reductase NAD binding domain | | CA209768 | UGSuM950 | (TG)8(AG)8 | GAGAGCATCCACAACATCA | ATTCCAAGACAAGGGTCGG | 55 | 364 | KAT11 | PF08214 | Histone acetylation protein | | CA282602 | UGSuM952 | (GA)9acc(AT)10 | ATCTGTCACTCAAGGCTAATG | AATCACAAGACTACTACAAGAGA | 54 | 333 | bZIP_2 | PF07716 | Basic region leucine zipper |   *UGSuM stands for unigene derived sugarcane microsatellite primers |  |  |  |  |  |  |  |  |  |  |
