# Supplementary material for: Functionally relevant microsatellites in sugarcane unigenes
Source: BMC Plant Biol. 2010 Nov 17;10:251. doi: 10.1186/1471-2229-10-251 (PMC3017843; doi:10.1186/1471-2229-10-251)
Supplement: Additional file 9 — Primers targeting the microsatellite repeats located within the functional domains of proteins encoded by sugarcane unigene sequences which gave single locus and step-wise allele amplification. [file 1471-2229-10-251-S9.DOC]

| Additional file 9: Primers targeting the microsatellite repeats located within the functional domains of proteins encoded by sugarcane unigene sequences which gave single locus and step-wise allele amplification   | **Sl. No.** | **Class I UGMS primers^A^** | **Unigene Accession IDs** | **Putative functions** | **Functional domains/protein families** | **Target UGMS region (bp) containing functional domains** | **Allele size distribution** | **Functional significance** | | --- | --- | --- | --- | --- | --- | --- | --- | | 1 | UGSuM5 | CA278792 | Cyclin III | Cyclin | 90-150 | Step-wise | Control nuclear cell division cycles and regulate cyclin dependent kinases (CDKs) | | 2 | UGSuM11 | AY596609 | Fructose biphosphate aldolase | Fructose biphosphate aldolase | 120-160 | Step-wise | Glycolytic enzyme that regulate carbohydrate metabolism (Glycolysis) | | 3 | UGSuM16 | CA278282 | Pathogenesis related proteins | Cysteine-rich secretory protein family | 160-230 | Step-wise | Conserved structural organization regulate disease resistance | | 4 | UGSuM17 | CA253277 | Protein kinase | PKinase family | 100-170 | Step-wise | Role in a multitude of cellular processes, including division, proliferation, apoptosis, and differentiation | | 5 | UGSuM21 | CA177414 | Transcription regulator proteins | Transcription factor | 170-240 | Step-wise | Mediate sequence specific DNA binding | | 6 | UGSuM26^B^ | BU103692 | Beta-amylase | Amylase catalytic domain | 260-310 | Step-wise | Regulate carbohydrate metabolism | | 7 | UGSuM41 | CA131350 | Unknown protein | - | - | Step-wise | - | | 8 | UGSuM50 | CA161416 | Hypothetical protein | - | - | Step-wise | - | | 9 | UGSuM74 | CA261182 | Transcription regulator protein | Transcription factor | 180-260 | Step-wise | Mediate sequence specific DNA binding | | 10 | UGSuM178 | CA291445 | O-diphenol-O-methyl transferase | Methyl transferase | 120-170 | Step-wise | Role in DNA methylation and number of cellular process including gene regulation and differentiation | | 11 | UGSuM186 | CA244023 | Ubiquitin C-terminal hydrolase | Ubiquitin | 200-230 | Step-wise | Significant biological role in transport of signaling proteins | | 12 | UGSuM197 | CA134472 | Epsin | ENTH (Epsin N-terminal homology) domain | 220-270 | Step-wise | Involved in structural organization | | 13 | UGSuM345 | CA084691 | DC1 | C1 domain | 210- 220 | Step-wise | Required for lipid metabolism | | 14 | UGSuM346 | CA093455 | DF1 | *recA* bacterial DNA recombination | 155-175 | Step-wise | Role in homologous recombination, DNA repair and introduction of the SOS response |   ^A^UGSuM stands for unigene derived sugarcane microsatellite primers  ^B^UGMS primers validated through sequencing |  |  |  |  |  |  |  |  |  |  |
| --- | --- | --- | --- | --- | --- | --- | --- | --- | --- | --- | --- | --- | --- | --- | --- | --- | --- | --- | --- | --- | --- | --- | --- | --- | --- | --- | --- | --- | --- | --- | --- | --- | --- | --- | --- | --- | --- | --- | --- | --- | --- | --- | --- | --- | --- | --- | --- | --- | --- | --- | --- | --- | --- | --- | --- | --- | --- | --- | --- | --- | --- | --- | --- | --- | --- | --- | --- | --- | --- | --- | --- | --- | --- | --- | --- | --- | --- | --- | --- | --- | --- | --- | --- | --- | --- | --- | --- | --- | --- | --- | --- | --- | --- | --- | --- | --- | --- | --- | --- | --- | --- | --- | --- | --- | --- | --- | --- | --- | --- | --- | --- | --- | --- | --- | --- | --- | --- | --- | --- | --- | --- | --- | --- | --- | --- | --- | --- | --- | --- | --- |
