# Supplementary material for: Functionally relevant microsatellites in sugarcane unigenes
Source: BMC Plant Biol. 2010 Nov 17;10:251. doi: 10.1186/1471-2229-10-251 (PMC3017843; doi:10.1186/1471-2229-10-251)
Supplement: Additional file 10 — Alignment depicting the functional significance of sugarcane UGMS markers targeting the microsatellite repeat-motif (AG)n located within the protein kinase domain of unigene. [file 1471-2229-10-251-S10.DOC]

**Additional file 10:** **Multiple sequence alignment of the four size variant alleles showing step-wise distribution in the protein kinase catalytic domain amplified from sugarcane species, genera, varieties and five cereals using primer (UGSuM17) for protein kinase unigene. Variation in the number of repeat-units of AG and the encoding repeated tracts of Arginine-Glutamine aminoacid residues at a microsatellite locus predicted different three dimensional protein structures which are highlighted.**

**UGMS repeat-motifs**

**Forward primer**

**Reverse primer**

**(AG)_22_**

**(AG)_18_**

**(AG)_10_**

**(AG)_6_**

**Predicted protein model for UGMS repeat-motif containing protein kinase domain**
